# Effects of intraventricular methotrexate administration on Cuprizone-induced demyelination in mice

**DOI:** 10.3389/fnmol.2013.00034

**Published:** 2013-10-16

**Authors:** Andre M. Mueller, Adam Nassery, Hana Conlon, Xinhe Liu, Esther Jun, Bo Hyung Yoon, Massimiliano Cristofanilli, Saud A. Sadiq

**Affiliations:** ^1^Tisch MS Research Center of New YorkNew York, NY, USA; ^2^Albert Einstein College of MedicineNew York, NY, USA; ^3^Columbia University School of NursingNY, USA; ^4^University of CaliforniaIrvine, CA, USA

**Keywords:** methotrexate, multiple sclerosis, cuprizone, astrocyte, IGF1, EAE

## Abstract

We previously showed that intrathecal administration of methotrexate slowed disability progression in multiple sclerosis (MS) patients with progressive disease. In general MS patients with progressive disease respond poorly to anti-inflammatory therapies. In order to better understand the mechanism by which methotrexate is protective in progressive MS, we analyzed its impact on the non-inflammatory cuprizone-induced demyelination model. When low-dose methotrexate was administered intracerebroventricularly it reduced demyelination and accumulation of GFAP+ reactive astrocytes in the corpus callosum. Administration of methotrexate after the withdrawal of cuprizone neither delayed remyelination nor influenced the number of astrocytes in the corpus callosum suggesting that methotrexate does not interfere with repair processes in the CNS. Moreover, methotrexate increased the expression of IGF1 *in vitro* and *in vivo*, a factor known to protect oligodendrocytes and limit the activation of astrocytes. Our studies show that methotrexate has an impact on pathogenic process in a demyelination model whose pathophysiological basis is not primarily related to inflammatory mechanisms, similar to neurodegenerative mechanisms associated with progressive MS. The pronounced inhibitory influence of methotrexate on the accumulation of astrocytes in the corpus callosum suggests that intrathecal methotrexate modulates astroglial activation in progressive MS possibly by promoting CNS production of IGF1.

## Introduction

Multiple sclerosis (MS) is characterized pathologically by demyelination, axonal loss and glial scar formation. Clinically, most patients have a relapsing-remitting course (RRMS) that over time may become secondarily progressive (SPMS). About 15% of patients have a progressive course from onset (PPMS). In the past decade, several treatments have been approved by the Food and Drug Administration (FDA) for use in RRMS and SPMS. However, these therapies are not effective for PPMS and become increasingly ineffective for SPMS especially in the degenerative phase of the disease. Thus, more effective therapies need to be developed for the progressive forms of MS (Hawker, 2011).

Methotrexate (MTX, amethopterin) is a folate analog that was introduced in clinical practice more than 50 years ago. It competitively inhibits dihydrofolate reductase (DHFR), an enzyme that participates in folate metabolism and purine synthesis and thereby MTX therapy results in inhibition of synthesis of ribonucleic acids.

MTX is an established chemotherapeutic agent and is also widely used in the treatment of patients with psoriasis, rheumatoid arthritis and other autoimmune diseases. In MS, low dose oral MTX (7.5 mg weekly) is effective, although minimally, in slowing deterioration in patients with SPMS (Goodkin et al., 1995). Our center conducted a study using intrathecal methotrexate administration (ITMTX) in 121 treatment unresponsive progressive MS patients with an initial EDSS score of more than 5.0 (Sadiq et al., 2010). ITMTX stabilized the disease course in 87% of secondary-progressive and in 82% of primary progressive MS patients within a two study period.

The mechanism of action of ITMTX in MS remains to be elucidated. Chemotherapeutic immunosuppressive agents have been extensively investigated in MS, but have limited efficacy in progressive forms of MS (Kieseier and Jeffery, 2010). Profound peripheral depletion of lymphocytes as a therapeutic strategy, while highly effective in RRMS and inflammatory (relapsing) forms of SPMS, has little or no benefit in late stage SPMS or PPMS (Bradl and Lassmann, 2009). This may be because these progressive forms of MS are pathologically in a degenerative and non-inflammatory phase of the disease and are, therefore, unresponsive to lymphocyte depletion (Bradl and Lassmann, 2009). One of the pathologic hallmarks of the chronic stage of disease is glial proliferation and scarring (Moore et al., 2011), and it is possible that ITMTX attenuates these aspects of the disease. An alternate explanation for the efficacy trends seen with ITMTX may be that some of the immune abnormalities seen in MS are primarily CNS in origin and are, therefore, more responsive to intrathecal therapy (Magliozzi et al., 2007). There is accumulating evidence that the inflammation seen in progressive MS is trapped behind a closed or repaired blood brain barrier in progressive MS (Meinl et al., 2008). This view is also supported by the observation that in progressive MS lymph follicle-like structures are formed with the connective tissue compartments within the CNS, meninges, and large perivascular spaces (Bradl and Lassmann, 2009). These CNS restricted immune responses may be unaffected by systemic immunosuppression or by agents such as natalizumab that stop lymphocyte trafficking across the blood brain barrier. It is possible that ITMTX acts by limiting inflammatory reactions in the CNS because of its route of administration circumventing an intact blood brain barrier.

Feeding of cuprizone [bis(cyclohexanone)-oxaldihydrazone] to mice induces a consistent, synchronous and anatomically reproducible demyelination (Kipp et al., 2009). Importantly, the pathology of this model is independent of adaptive immune responses (Arnett et al., 2001). Furthermore, removal of cuprizone from the mice's diet leads to remyelination (reviewed in Matsushima and Morell, 2001). Cuprizone chelates copper, an essential component of metalloenzymes, like the mitochondrial cytochrome oxidase and monoamine oxidase (Acs and Komoly, 2012). It has been assumed although not proven that copper deficiency results in specific damage to oligodendrocytes in the CNS and subsequent demyelination. The specific susceptibility of oligodendrocytes has been attributed to the high metabolic demand of these glial cells required to maintain a vast expanse of myelin and the resulting vulnerability to a disturbed energy metabolism. This model allows analysis of demyelination in the CNS accompanied by strong astrogliosis, without the interference of the peripheral immune system (Matsushima and Morell, 2001).

To better understand the mechanisms of action of ITMTX on progressive MS, we examined the effects of this therapy in the cuprizone induced demyelination model.

## Materials and methods

### Animals

C57Bl/6 mice, either 8 weeks or 1 year old, were purchased from the Jackson Laboratory (Bar Harbor, ME). Animals were housed in the animal facility of Roosevelt Hospital (New York, NY) where our research center conducts its animal research. Food and water were available *ad libitum*. All procedures were conducted according to protocols approved by the Institutional Animal Care and Use Committee (IACUC) of St. Luke's Roosevelt Institute of Health Sciences (New York City, NY).

### Induction of demyelination

Demyelination was induced by feeding C57Bl/6 mice a diet of 0.2% cuprizone (bis-cyclohexanone oxaldihydrazone, Sigma-Aldrich, St.Louis, MO) mixed into ground standard chow. The ground chow supplemented with cuprizone was replaced every other day.

### EAE induction

Active EAE in female C57/B6 mice was induced by s.c. immunization with 200 μg MOG-peptide (aa 35–55: MEVGWYRSPFSRVVHLYRNGK, Pepceuticals, UK) emulsified 1:1 in CFA containing 450 μg H37Ra and i.p. injection of 200 ng pertussis toxin (Merck KGaA Germany) on days 0 and 2.

### Administration of methotrexate

The Alzet Brain Infusion Kit 3 (DURECT Corp., Cupertino, CA) and the Alzet osmotic pump (Alzet 1004, DURECT Corp.) were used to deliver 0.5 mg/mL methotrexate (MTX) dissolved in a 10 mg/mL BrdU/PBS dilution or 10 mg/mL BrdU/PBS (vehicle) directly into the third ventricle. The osmotic pumps were connected to the brain infusion cannula by catheter tubing 1 day before surgery. The filled infusion assembly with attached osmotic pump was incubated overnight in sterile saline at 37°C. A group size of eight animals was examined in all experiments.

For pump installation, mice were anesthetized with a mixture of Ketamine (77 mg/kg)/Xylazine (15 mg/kg) in saline. The animals were fixed in a stereotactic frame with non-traumatic ear-bars to hold the skull in place. A small hole was created in the skull with a drill bit. The coordinates of the hole for the cannula were 0.94 mm posterior to the bregma, 0 mm lateral, and 2.5 mm ventral to the skull surface. The stereotactic arm holding the cannula was lowered into the third ventricle and glued to the skull surface using veterinary skin adhesive (3M Vetbond, 3M Animal Care Products, St. Paul, MN). Thereafter, the cannula was covered by orthodontic powder mixed with acrylic resin liquid (Lang Dental Manufacturing Inc., Wheeling, IL). The mini-pump was inserted beneath the skin at the right scapula and the skin was closed using surgical adhesive. The volume and the rate of delivery of PBS or MTX by mini-pump were ~100 nL per h continuously for 28 days. Mice received approximately 50 μg MTX per kg body weight and day mirroring the low dose intrathecal MTX administration in progressive MS patients (Sadiq et al., 2010). Mice were housed individually to avoid possible damage to the mini-pump.

### Quantification of demyelination, glial activation and astroglial processes length

The amount of demyelination and accumulation of reactive astrocytes and macrophages and microglial cells in the corpus callosum were quantified by immunohistofluorescent analyses.

At the end of the experiment, mice were transcardially perfused with 4% PFA. The brains were removed, postfixed overnight in PFA and embedded in paraffin. 5 μm serial paraffin sections representing the corpus callosum were cut, dried at 37°C, de-waxed, rehydrated and boiled for 5 min in 10 mM citrate buffer (pH 6.0). Sections were quenched with H_2_O_2_, blocked with 1% goat serum and incubated with primary antibodies for the detection of myelin, astrocytes and macrophages/ microglial cells. Following primary antibodies were used: anti-BrdU (clone G3G4, Development Studies Hybridoma-Bank, IA), polyclonal rat anti-MBP (Millipore, MA), mouse anti-GFAP (clone G-A-5, Sigma Aldrich) and rabbit anti-Iba1 (Thermo Scientific). Isotype control antibodies and staining without primary antibodies were used as controls. Following secondary antibodies were used: Alexa Fluor 555 goat anti-rabbit IgG, Alexa Fluor 488 goat anti-mouse IgG1, Alexa Fluor 647 goat anti-mouse IgG2 and Alexa Fluor 647 goat anti-rat (all from Abcam).

In order to quantify the number of reactive astrocytes and of macrophages/microglial cells in the corpus callosum, GFAP+ cells as well as Iba1+ positive cells were counted by a blinded investigator. At least twelve brain slices per mouse were analyzed. The myelination of the corpus callosum was determined by calculating the ratio of the demyelinated area in the corpus callosum and the total area of the corpus callosum.

The length of the astroglial processes was determined using the Fiji software (Schindelin et al., 2012) based on GFAP staining of corpus callosum containing brain sections. For every slide the length of 20 representative astroglial processes was determined by a blinded investigator. Six slides per mouse were analyzed.

### CSF samples and microarray procedure

Cerebrospinal fluid (CSF) was collected with informed consent and approval (protocol number 05–178) from the St. Luke's-Roosevelt Hospital Center Institute for Health Sciences Institutional Review Board (New York, NY) either via standard lumbar puncture or by access port aspiration of an implanted baclofen pump for the treatment of spasticity. CSF samples were centrifuged and the cell-free supernatant was stored frozen at −80°C. RNA was isolated from pelleted CSF cells using the RNeasy Micro Kit (Qiagen, Hilden, Germany).

RNA was processed and analyzed by microarray hybridization by the Centre of Excellence for Fluorescent Bioanalytics (KFB, Regensburg, Germany) as described before (Muller et al., 2012).

Probe set intensities; background correction and signal normalization were done using the MAS 5.0 algorithm (Affymetrix). Analyses were performed using BRB Array Tools version 4.1.0 (Simon et al., 2007). Signal intensities were normalized using the array with the median overall signal intensities as reference. All gene expression intensities below 10 were set to the value of 10. Probe sets with less than 10% of expression data values having at least 1.5-fold change in either direction from the gene's median value were excluded from further evaluations.

### Quantification of IGF1 protein in human CSF

IGF1 concentrations in cell-free CSF were measured using the Human IGF1 ELISA development Kit, DuoSet (R&D Systems, Minneapolis, MN). Measurements were performed in duplicates.

### Quantification of IGF1 expression in corpus callosum

After 4 weeks of cuprizone feeding and icv MTX administration, mice were sacrificed and perfused via left cardiac ventricle with RNAse-free phosphate buffered saline (PBS) for gene expression analysis. A group size of eight animals was investigated. For gene expression analysis the brains were removed and immediately embedded in O.C.T. TISSUE-Tek Compound (Sacura, CA) and stored at −80°C until use. Serial coronal sections with a thickness of 10 μm were cut at −20°C. The sections were mounted on PEN membrane slides (LEICA GmbH, Germany) and prepared for microdissection using the Histogene LCM Frozen Section Staining Kit (Life Technologies). The LMD6000 laser microdissection microscope (Leica GmbH, Germany) was used to excise the medial part of the corpus callosum. The RNeasy Micro plus kit (Qiagen, Germany) was used to isolate RNA immediately after microdissection. cDNA was generated using the Sensiscript RT kit (Qiagen). The IGF1 expression was quantified using murine IGF1 specific Taqman probes (Mm00439560_m1, Applied Biosystems) and normalized to respective 18S rRNA quantities also determined by using Taqman probes.

### Methotrexate treatment of mouse embryonic-derived oligodendrocytes precursor cells (ES-OPCs) and quantification of IGF1 expression

We studied a highly pure batch of ES-OPCs, previously established and characterized in our lab. To address the impact of methotrexate on the ability of oligodendroglial cell to produce IGF1, ES-OPCs (100,000 per well in a 24 well plate) were incubated for 10 days in 2 mL of N2 media without growth factors, a condition known to promote terminal differentiation of these cells into oligodendrocytes (Brustle et al., 1999), supplemented with varying amounts of methotrexate. 25% of the medium was changed every other day. The IGF1 expression was quantified using murine IGF1 specific Taqman probes as described above for microdissected tissue analysis.

### Statistical analyses

Statistical analyses were performed using SigmaStat 3.0 (IBM, Somers, NY). Comparisons of groups of normal-distributed data were done by Student's *t*-test or an ANOVA analysis.

## Results

### Influence of methotrexate on cuprizone-induced pathology in the corpus callosum

**Figure 1:**
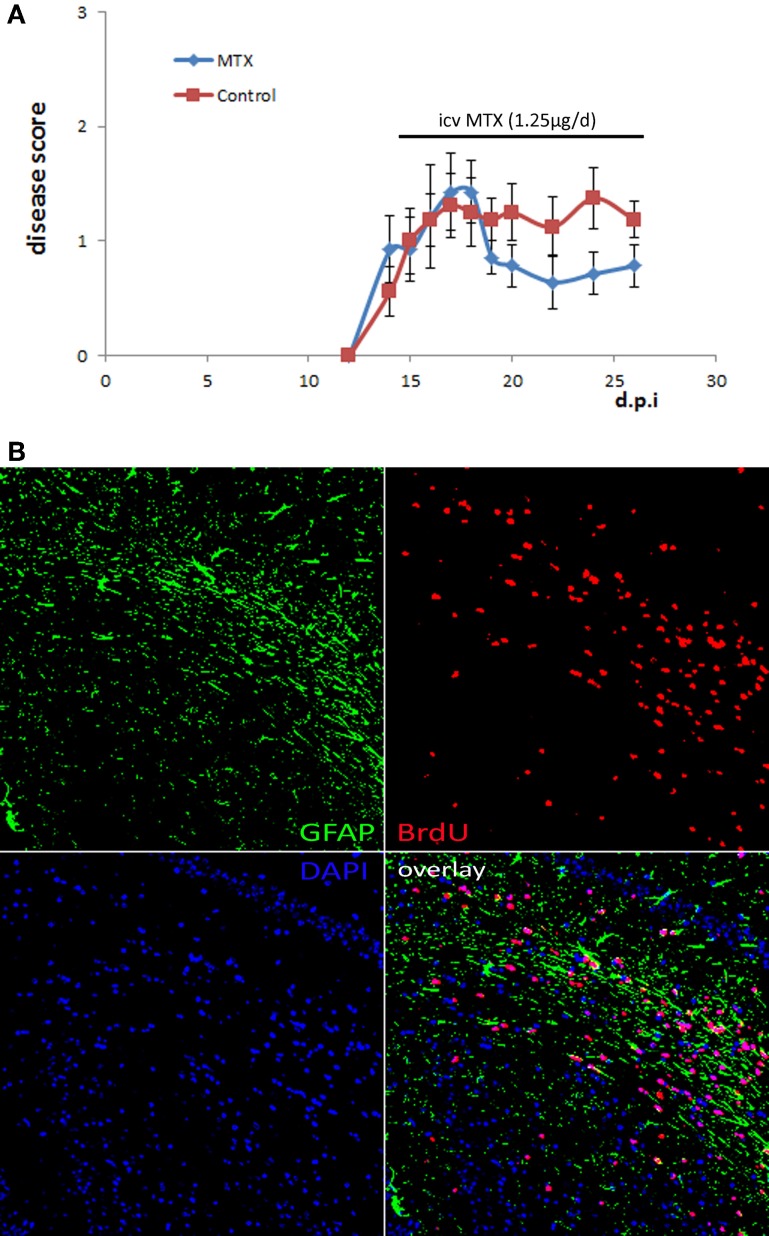
**Osmotic pumps delivering MTX/PBS were functional. (A)** Active EAE was induced in 16 female C57Bl/6 mice. Half of the mice received icv MTX (1.25 μg per day) starting at day 14 after disease induction, control mice received PBS instead. Error bars represent the standard error of the mean. **(B)** Representative BrdU staining of brain sections of a mouse that was treated with MTX/BrdU delivered by osmotic pumps for 4 weeks showing that BrdU was delivered into the brain implying that the respective animal also received methotrexate (200 × magnification).

We aimed to investigate the effect of methotrexate administration on the cuprizone-induced demyelination mouse model that is characterized by a highly reproducible demyelination of distinct brain regions independent of an immune attack on the myelin sheet (Kipp et al., 2009). Mice received methotrexate intracerebroventricularly by osmotic pumps at a dosage of approximately 1.25 μg per day. Methotrexate was reported before to be protective in the context of the inflammatory MS animal model experimental autoimmune encephalomyelitis (EAE) (Rosenthale et al., 1969). In order to confirm that the selected dosage of 1.25 μg per day had an *in vivo* effect, we tested it within the context of EAE. The administration started at the beginning of EAE disease symptoms (day 14). This protocol demonstrated an *in vivo* effect in EAE by decreasing the disease symptoms (Figure 1A).

**Figure 2:**
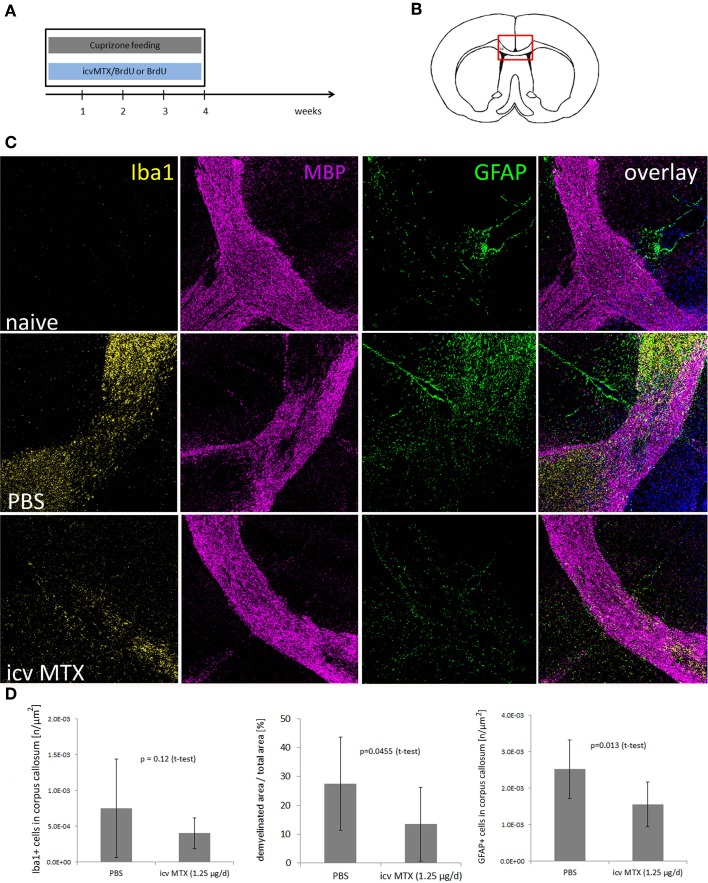
**icv MTX administration modulates Cuprizone-induced demyelination and astroglial activation. (A,B)** Eight mice per group were fed with cuprizone and received either 1.25 μg/day methotrexate dissolved in 10 mg/mL BrdU/PBS intracerebroventricularly or just 10 mg/mL BrdU in PBS. After 4 weeks animals were sacrificed and the midline of the corpus callosum was analyzed for pathological differences. **(C)** Representative myelin (MBP), macrophage/microglial cell (IBA1), and astrocyte (GFAP) staining of brain sections containing the corpus callosum (200× magnification). **(D)** Quantitative analyses of demyelination and numbers of macrophages/microglial cells and astrocytes in corpus callosum. Twelve brains slices per mouse were analyzed. Error bars represent standard deviations.

Next, we determined the impact of icv MTX on non-inflammatory demyelination, 16 mice were fed with cuprizone for 4 weeks. Half of the animals received methotrexate intracerebroventricularly (icv) dissolved in BrdU/PBS delivered by osmotic pumps during the whole 4 weeks of cuprizone feeding (Figures 2A,B). Control mice received BrdU dissolved in PBS. Immunohistofluorescent analyses focused on the corpus callosum were setup aiming to determine potential alterations caused by methotrexate and to confirm the function of the osmotic pumps by BrdU staining (shown in Figure 1B). After 4 weeks of cuprizone feeding, mice were characterized by a reproducible demyelination and an increase in GFAP+ astrocytes and Iba1+ monocytic cells within the medial part of the corpus callosum as compared to treatment naive mice. Icv MTX significantly decreased the demyelination and accumulation of astrocytes in the corpus callosum, while the number of Iba1+ monocytic cells in the corpus callosum was not significantly altered by methotrexate (Figures 2C,D).

High-dosage of methotrexate is known to cause leukoencephalopathy, brain demyelination and astrocytosis (Gregorios et al., 1989; Surtees et al., 1998). In order to exclude that the dosage administered to cuprizone-fed mice had significant detrimental effects, we treated naïve mice with icv MTX as described before for 4 weeks without concomitant cuprizone feeding. Immunohistofluorescent analyses of demyelination and astrocyte accumulation in corpus callosum showed no demyelination and very low number of GFAP+ astrocytes. In this experimental setup, icv MTX-treated mice were indistinguishable from PBS-treated control mice (Supplementary Figure 1) suggesting that the chosen dosage of methotrexate did not have significant side effects in murine brains.

**Figure 3:**
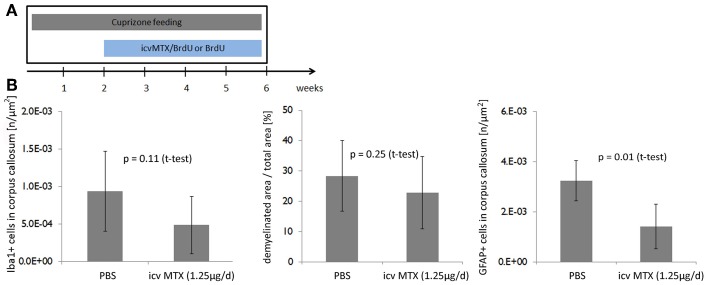
**Delayed onset of icv MTX administration does not prevent demyelination but reduces astroglial activation. (A)** Eight mice per group were fed with cuprizone for a total of 6 weeks. For the last 4 weeks the mice received additionally either 1.25 μg/day methotrexate dissolved in 10 mg/mL BrdU/PBS intracerebroventricularly or just 10 mg/mL BrdU in PBS. **(B)** Quantitative analyses of demyelination and numbers of macrophages/ microglial cells and astrocytes in corpus callosum. Twelve brains slices per mouse were analyzed. Error bars represent standard deviations.

We next showed that the effects of icv MTX on demyelination and astroglial activation are dependent on the timing of MTX administration. We analyzed mice fed with cuprizone for 6 weeks that also received either methotrexate or PBS intracerebroventricularly for the last 4 weeks (Figure 3A). In this setting, methotrexate administration resulted in neither a significant decrease of demyelination seen in the corpus callosum nor an alteration of monocytic cell accumulation, but the number of astrocytes accumulated in the corpus callosum was significantly lower in icv MTX treated mice than in control mice (Figure 3B).

By using confocal microscopy, the influence of icv MTX on astrocytes' morphology was investigated. The majority of astrocytes found in the corpus callosum of PBS- and icv MTX treated mice had a protoplasmic shape with only a very limited number of processes (Supplementary Figure 2A) The average length of astrocytic processes in PBS- and icv MTX-treated mice did not differ significantly from each other (Supplementary Figure 2B). Thus, icv MTX does not seem to have an obvious effect on the morphology of astrocytes accumulating in the corpus callosum during cuprizone-induced demyelination.

### Methotrexate does not interfere with remyelination of the corpus callosum

**Figure 4:**
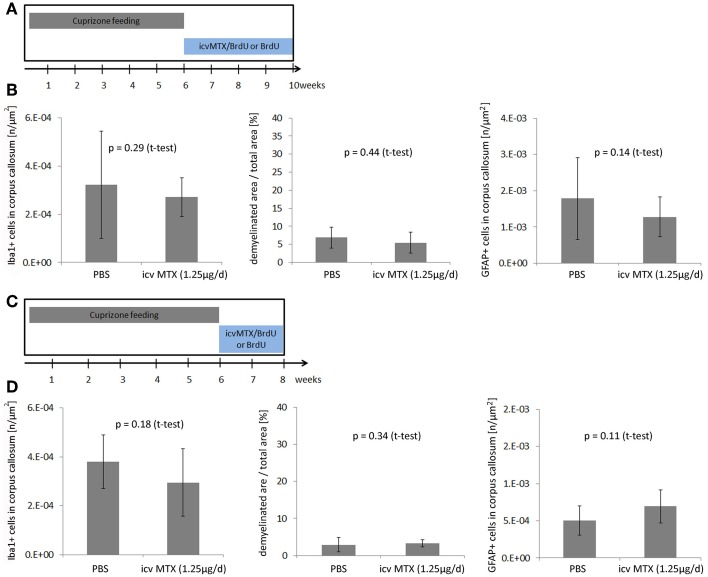
**icv MTX administration does not interfere with repair processes after Cuprizone withdrawal. (A)** Eight mice per group were fed with cuprizone for a total of 6 weeks. Afterwards mice received either 1.25 μg/day methotrexate dissolved in 10 mg/mL BrdU/PBS intracerebroventricularly or just 10 mg/mL BrdU in PBS for another 4 weeks. **(B)** Quantitative analyses of demyelination and numbers of macrophages/ microglial cells and astrocytes in corpus callosum. Twelve brains slices per mouse were analyzed. Error bars represent standard deviations. **(C)** Eight 1 year old mice per group were fed with cuprizone for a total of 6 weeks. Afterwards mice received either 1.25 μg/day methotrexate dissolved in 10 mg/mL BrdU/PBS intracerebroventricularly or just 10 mg/mL BrdU in PBS for another 2 weeks. **(D)** Quantitative analyses of demyelination and numbers of macrophages/microglial cells and astrocytes in corpus callosum. Twelve brains slices per mouse were analyzed. Error bars represent standard deviations.

Remyelination is a complex process ensuring cellular integrity after neuronal damage, as well as after primary loss of mature oligodendrocytes. The cuprizone model is a suitable tool to study the molecular mechanisms of remyelination *in vivo* (Armstrong et al., 2006). To determine, if MTX interferes with remyelination, mice received methotrexate intracerebroventricularly after the withdrawal of cuprizone from the diet (Figure 4A). In particular, mice were fed with cuprizone for 6 weeks followed by 4 weeks of feeding with normal chow without cuprizone to allow the generation of oligodendrocytes. Eight mice received icv MTX during these 4 weeks after cuprizone withdrawal; control mice received PBS instead. Quantitative assessment of Iba1−, MBP− and GFAP-stained brain slices revealed that the withdrawal of cuprizone from the diet reduced the number of Iba1+ cells and GFAP+ astrocyte numbers in the corpus callosum as well as the amount of demyelination (data not shown). Mice treated with icv MTX did not differ significantly from the control mice in any of the aforementioned parameters (Figure 4B) suggesting that MTX does not interfere with the spontaneous recovery after the withdrawal of cuprizone.

The processes leading to remyelination of the corpus callosum after 6 weeks of cuprizone feeding were reported to be relatively fast (Stidworthy et al., 2003). To determine the effects of icv MTX on remyelination in less favorable conditions, we used 1 year old mice as remyelination is naturally less robust during aging (Rist and Franklin, 2008). The mice were sacrificed 2 weeks after cuprizone withdrawal (Figure 4C). We found no significant differences in the number of Iba1+ and GFAP cells and demyelination of the corpus callosum between icv MTX and control treated mice (Figure 4D), corroborating our hypothesis that MTX is not interfering with repair processes in the cuprizone-induced demyelination model.

### Possible mechanism underlying the effect of icv MTX

**Figure 5:**
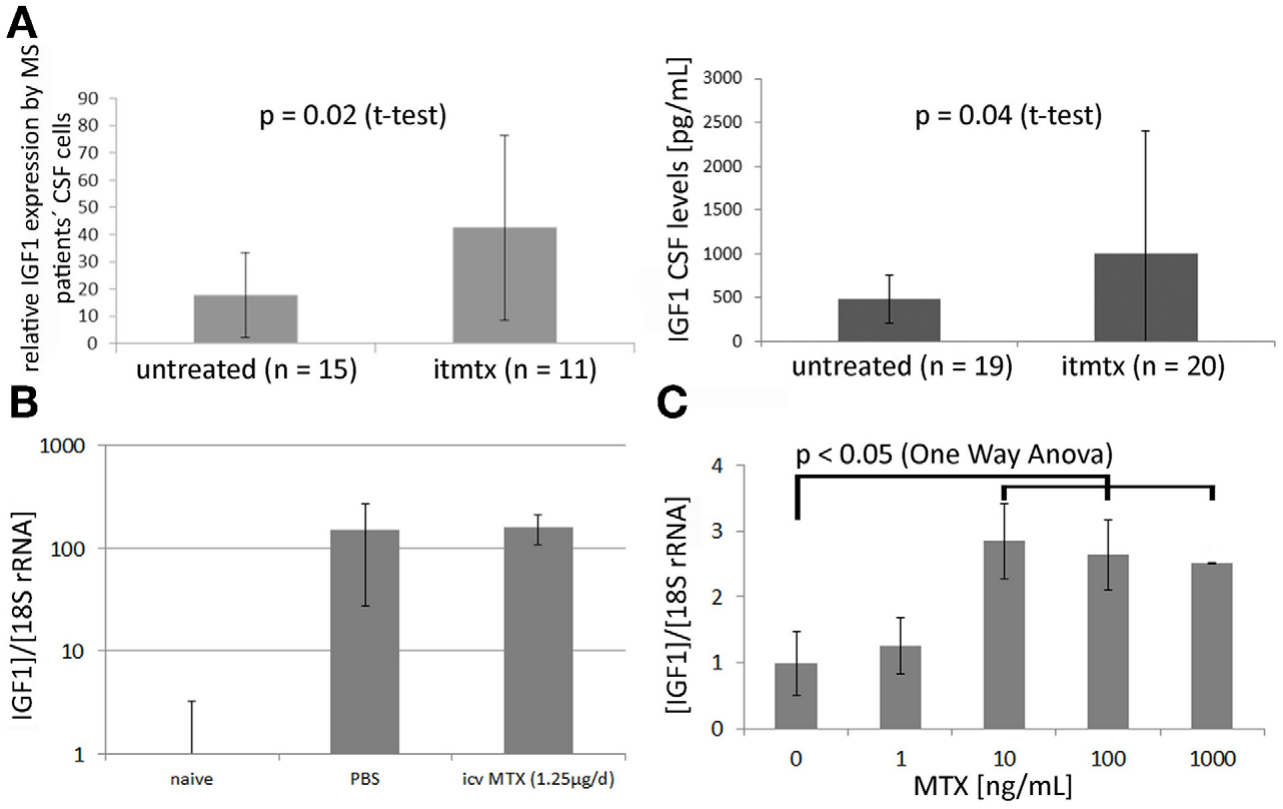
**Methotrexate modulates IGF1 expression in CNS and by embryonic stem cells. (A)** Left: The CSF cell transcriptome of 15 untreated progressive MS and 11 ITMTX treated patients was analyzed by microarray hybridization. Right: IGF1 levels were quantified in cell-free CSF samples obtained from 19 control patients and 20 MS patients using a commercially available IGF1 ELISA (RandD Systems). **(B)** Mice were fed with cuprizone for 4 weeks. Additionally, they either received icv MTX or PBS. RNA was isolated from the midline section of corpus callosum and IGF1 expression determined by RealTime PCR. Bars represent IGF1 expression in respective mouse group standardized to IGF1 expression in corpus callosum of treatment naïve mice. **(C)** Methotrexate increases the expression of IGF1 by murine embryonic-derived oligodendroglial progenitor cells. 100,000 ES-OPCs cells incubated with different amounts of methotrexate diluted in differentiation medium. After 10d the expression of IGF1 was determined by RealTime-PCR (One-Way ANOVA). Error bars represent Standard Deviations.

In order to elucidate the protective mechanism of methotrexate, we analyzed the CSF cell transcriptome of eleven progressive MS patients treated intrathecally with methotrexate and of fifteen untreated progressive MS patients as described before (Muller et al., 2012). By definition, untreated MS patients were not on any disease modulating treatment for at least 1 year before the CSF sample was drawn. 1596 probe sets were identified as differentially expressed between ITMTX-treated MS patients and untreated MS patients (Supplementary Table 1). The gene encoding for the insulin-growth factor 1 (IGF1) was expressed in significantly increased magnitude by CSF cells of patients treated intrathecally with methotrexate compared to untreated MS patients (Figure 5A, patients' characteristics in Table 1). IGF1 is of high interest, because it is known to protect oligodendrocytes from cell death and prevents demyelination in the cuprizone model (Mason et al., 2001). Additionally, it inhibits the activation of astrocytes *in vivo* and *in vitro* (Fernandez et al., 2007; Bellini et al., 2011). To confirm our finding of the increased expression of IGF1 by CSF cells of ITMTX treated patients, we quantified IGF1 protein in CSF samples derived from 19 untreated and 20-ITMTX-treated progressive MS patients. CSF levels of IGF1 protein were significantly higher in methotrexate-treated progressive MS patients than in untreated MS patients (Figure 5A, patients' characteristics in Table 1).

**Table 1:** **Patients' characteristics**.

	***n***	**Gender [F/M]**	**Age ± *SD* [years]**	**EDSS ± *SD***	**Disease duration ± *SD* [years]**	**PPMS/SPMS**
**MICROARRAY STUDY**						
Untreated	15	12/3	52.6 ± 13	6.1 ± 1.9	18.5 ± 12	6/9
Itmtx treated	11	7/4	57.1 ± 12	6.7 ± 1.5	17.1 ± 9.7	4/7
**IGF1 PROTEIN QUANTIFICATION IN CSF**						
Untreated	19	7/12	55.0 ± 9	3.5 ± 11	19.6 ± 10	4/15
Itmtx treated	20	5/15	51.4 ± 10	6.8 ± 1	21.7 ± 11	3/17

Using a laser microdissection microscope, we isolated RNA from the medial part of the corpus callosum of mice fed with cuprizone for 4 weeks that received either methotrexate or PBS intracerebroventricularly. IGF1 expression was determined by RealTime PCR. We found a more than 150-fold upregulation of IGF1 in cuprizone-fed mice compared to treatment naïve mice, but no difference between icv MTX treated mice and control mice (Figure 5B). Cuprizone feeding is already known to cause a very strong upregulation of IGF1 (Komoly et al., 1992; Voss et al., 2012). Hence, the general induction of IGF1 caused by cuprizone may overshadow potential effects of icv MTX on IGF1 expression in the brain of cuprizone-fed mice.

To corroborate that methotrexate influences IGF1 expression, we incubated murine ES-OPCs in differentiation medium for 10 days with varying amounts of methotrexate and quantified the expression of IGF1 by RealTime PCR. This experimental setup resulted in a significant increase of IGF1 expression in cells treated at a concentration of 10 ng/ml methotrexate or more (Figure 5C). These results indicate that methotrexate increases the production of the growth factor IGF1 *in vivo* (MS patients) and *in vitro* (ES-OPCs). IGF1 upregulation possibly contributes to the effects icv MTX has on demyelination and astroglial activation.

## Discussion

Methotrexate is an antimetabolite that inhibits dihydrofolic acid reductase. Dihydrofolate reduction to tetrahydrofolate is necessary because it acts as a one-carbon group carrier in the synthesis of purine nucleotides and thymidylate. Mechanistically, methotrexate interferes with DNA synthesis and cellular replication, and thus inhibits cellular proliferation such as associated with malignancy or generation of immune responses. Methotrexate has a well-established protective effect in EAE, an inflammatory animal model of MS (Rosenthale et al., 1969; Levine and Sowinski, 1977). This protection relies most likely on its well established immunosuppressive properties. Although immunosuppressive agents are of limited therapeutic value in progressive MS (Kieseier and Jeffery, 2010), intrathecal administration of low doses of methotrexate provided a benefit in progressive MS patients resulting in a halt of the disability progression in 89% of 121 treated progressive MS patients (Sadiq et al., 2010). This suggested that the mechanism of action of ITMTX in progressive MS might not be solely mediated through immune suppression.

To determine if methotrexate has an impact on neurodegenerative processes not primarily caused by inflammation, we investigated its use in the cuprizone-induced demyelination model which is characterized by a primary oligodendrocyte apoptosis independent of adaptive immunity (Arnett et al., 2001). This model is frequently used to study processes leading to de- and remyelination in the CNS in the absence of an autoimmune component. It is debatable to what extent cuprizone-induced demyelination reflects human MS pathology. Primary oligodendrocyte death and activation of astrocytes are hallmarks of the cuprizone animal model. These pathological features are also characteristic for human MS lesion formation. In addition, cuprizone-induced oligodendrocyte-apoptosis may reflect pathological steps of newly forming MS lesions in humans, because human type III and IV MS lesions are partially mimicked by the cuprizone model (Kipp et al., 2009; Zendedel et al., 2013).

We set up a series of experiments in which the icv delivery of methotrexate started either at the onset of cuprizone feeding, after 2 weeks of cuprizone feeding or upon withdrawal of cuprizone. Mice received 1.25 μg methotrexate per day delivered by osmotic pumps, a dosage that was protective in active MOG-induced EAE of C57Bl/6 mice and correlated with the low dose ITMTX administration in progressive MS patients (Sadiq et al., 2010).

We found that MTX significantly reduced demyelination in the corpus callosum only when administrated concurrently with the cuprizone feeding, but not when started after 2 weeks of cuprizone feeding. This suggested that methotrexate influences the onset of demyelination, but does not alter established demyelinating processes. Methotrexate did not modulate the number of Iba1+ macrophages/microglial cells in any of the experiments significantly.

The most noticeable effect of MTX in the cuprizone model was its inhibitory effect on the accumulation of reactive astrocytes within the corpus callosum. Diffuse activation of astrocytes with astrocytic proliferation and increased GFAP expression is a characteristic pathologic feature of the cuprizone model. Methotrexate decreased the number of reactive astrocytes accumulating in the corpus callosum as determined by a significant reduction in the number GFAP+ astrocytes in the corpus callosum. This effect was seen in both, mice receiving icv MTX at onset of cuprizone feeding and in mice treated with MTX 2 weeks after initiation of cuprizone feeding. This data showed that icv MTX inhibits the accumulation of reactive GFAP+ astrocytes in the corpus callosum in this model. Although astroglial activation is a prominent feature of MS, its exact role in the pathogenesis remains to be elucidated: Activated astrocytes contribute to myelin breakdown by phagocytosis and to act as professional antigen presenting cells (Lee et al., 1990), to secrete proinflammatory cyto- and chemokines (Tanuma et al., 2006; De Keyser et al., 2008) and to interfere with myelin repair through astroglial scar formation (Fawcett and Asher, 1999). However, selective ablation of proliferating astrocytes in murine CNS worsened the disease course of EAE due to increased CNS infiltration of pathogenic immune cells (Voskuhl et al., 2009; Toft-Hansen et al., 2011).

The specific role of astrocytes within progressive forms of MS is hardly known: In chronic progressive MS astrocytic proliferation is the hallmark feature of the characteristic sclerotic plaques seen pathologically (Kuhlmann et al., 2008). In secondary progressive MS patients, hyperactive astrocytes were found at the rim of chronic demyelinating CNS lesions. These astrocytes produced a variety of proinflammatory mediators like CCL2 and CXCL10. As these lesions did not include T-cells, it was concluded that astrocytes may be key players driving the development of chronic demyelinating CNS lesions (Tanuma et al., 2006). Based on our findings in this cuprizone model one could speculate that the deceleration of disability progression seen in ITMTX treated progressive MS patients is partially due to an inhibition of astroglial proliferation leading to a suppression of proinflammatory chemokines and due to a reduction of scar formation.

Demyelinated brain areas spontaneously remyelinate once cuprizone is withdrawn from the diet. In the context of the cuprizone model, remyelination is generally assumed to be performed either by surviving oligodendrocytes and by maturation of oligodendroglial progenitor cells into myelin-producing oligodendrocytes (Kipp et al., 2009). Mice in which methotrexate was administered after the withdrawal of cuprizone were indistinguishable from control animals with regard to the degree of myelination and to the number of macrophages/microglial cells and astrocytes in the corpus callosum implying that methotrexate does not affect repair processes in the cuprizone model. There are several possible reasons for the lack of impact of icv MTX on the remyelination of the corpus callosum in our experiments: (1) The processes leading to the remyelination of demyelinated brain regions after the withdrawal of cuprizone from the diet are supposed to be very fast and reliable (Zendedel et al., 2013). We studied methotrexate's influence on remyelination under stringent conditions by using mice with an age of 1 year at start of the experiment and by analyzing them as soon as 2 weeks after cuprizone withdrawal. Nevertheless, it cannot be completely excluded that we did not find any impact of icv MTX on repair processes because the animals were sacrificed at time points at which the repair was basically accomplished. (2) De- and remyelination were quantified in this study by immunohistofluorescent-based analyses of MBP in brain sections. MPB is a myelin component that is re-expressed very early after the withdrawal of cuprizone from the diet (Lindner et al., 2008). It is possible that analyses of MOG whose re-expression is considerably delayed in comparison to MBP expression after cuprizone withdrawal (Lindner et al., 2008) would have uncovered an impact of icv MTX on remyelination of the corpus callosum. (3) Mice received 1.25 μg of methotrexate per day. This low dose could have been not enough to interfere with remyelination mediated by mature oligodendrocytes that have survived the cuprizone treatment and by differentiation of oligodendroglial progenitor cells into mature oligodendrocytes. (4) Neural stem cells growing in a folate-depleted culture or in the presence of methotrexate were reported to differentiate preferentially into glial cells (Sato et al., 2006). Thus, a possible repair-delaying property of methotrexate within the recovery phase of the cuprizone-model could have been counteracted by an enhancement of the differentiation of neural stem cells into oligodendrocytes contributing to remyelination.

Quantification of the CSF cell transcriptome of progressive MS patients treated with intrathecal methotrexate revealed that the expression of the growth factor IGF1 by CSF cells is higher in ITMTX treated than in untreated progressive MS patients. Increased IGF1 protein levels in CSF samples of ITMTX treated patients compared to untreated patients substantiated this finding. Unfortunately, we could not establish a direct link between methotrexate and IGF1 in the cuprizone model as determined by IGF1 expression in the corpus callosum of mice fed with cuprizone. This may be because IGF1 is more than 150-fold more strongly expressed in cuprizone-treated mice than in treatment naïve mice. The strong IGF1 upregulation that is generally seen in the cuprizone model (Komoly et al., 1992) arguably overrides a putative induction of IGF1 by methotrexate. However, IGF1 expression was induced in murine ES-OPCs incubated in differentiation medium supplemented with 10 ng/mL of methotrexate or more. As IGF1 was described as an agent that protects oligodendrocytes in the cuprizone model (Mason et al., 2001) and to inhibit the activation of astrocytes *in vitro* and *in vivo* (Bellini et al., 2011), the increased production of IGF1 in the CNS of ITMTX treated patients plus the induction of IGF1 by methotrexate in oligodendroglial progenitor cell cultures provides indirect evidence for a putative mechanism. Methotrexate administration leads to an increase of IGF1 production in the CNS which may favor the survival of oligodendrocytes and interfere with astroglial activation.

Our studies establish a limited protective effect of methotrexate on demyelination and a more pronounced inhibitory effect on accumulation of reactive GFAP+ astrocytes in a non-inflammatory demyelination model. This corroborates that the beneficial impact of ITMTX in progressive MS patients reported earlier (Sadiq et al., 2010) is not solely mediated by its anti-inflammatory properties. Our work indicates that inhibition of astroglial activation, possibly by increasing the production of the growth factor IGF1 in the CNS may retard the generation of astrocytic scars or sclerosis in MS lesions.

## Author contributions

Andre M. Mueller conceived the design of the study, performed experiments as well as experimental analysis and prepared the manuscript. Adam Nassery performed experiments and supported the design of the study. Xinhe Liu established the installation of osmotic pumps for the icv delivery of methotrexate. Bo Hyung Yoon, Esther Jun, and Hana Conlon provided technical support for the experiments and for data collection and drafted the manuscript. Massimiliano Cristofanilli provided the ES-OPCs. Saud A. Sadiq provided guidance for all aspects of the work. All authors read and approved the final version of the manuscript.

### Conflict of interest statement

The authors declare that the research was conducted in the absence of any commercial or financial relationships that could be construed as a potential conflict of interest.

## Supplementary material

Supplementary Figure 1 **A 4-week icv MTX administration does not have detrimental side effects. (A)** Experimental setup: Eight mice per group were treated with icv MTX. Another eight mice received PBS intraventricularly. **(B)** Quantitative analyses of numbers of GFAP+ astrocytes in corpus callosum. Ten brains slices per mouse were analyzed. **(C)** Quantitative analyses of numbers of demyelination in corpus callosum. Ten brains slices per mouse were analyzed. Error bars represent standard deviations.

Supplementary Figure 2 **Methotrexate does not affect the length of astroglial processes in the corpus callosum. (A)** Top: Representative image of corpus callosum of PBS- treated mouse (600× magnification). Bottom: Representative image of corpus callosum of icv MTX-treated mouse (600× magnification). **(B)** Quantitative analyses of the length of astroglial processes. Six brains slices per mouse were analyzed. Error bars represent standard deviations.

Supplementary Table 1 **Genes differentially expressed by CSF cells of ITMTX treated MS patients compared to untreated MS patients**. 794 probe sets were upregulated in ITMTX-treated MS patients and 802 downregulated (*p* ≤ 0.05).

## Abbreviations

CSF: cerebrospinal fluid
CC: corpus callosum
EAE: experimental autoimmune encephalomyelitis
icv MTX: intracerebroventricular methotrexate
ITMTX: intrathecal methotrexate.
