# Supplementary figures and images for: Effects of intraventricular methotrexate administration on Cuprizone-induced demyelination in mice
Source: Front Mol Neurosci. 2013 Oct 16;6:34. doi: 10.3389/fnmol.2013.00034 (PMC3797440; doi:10.3389/fnmol.2013.00034)

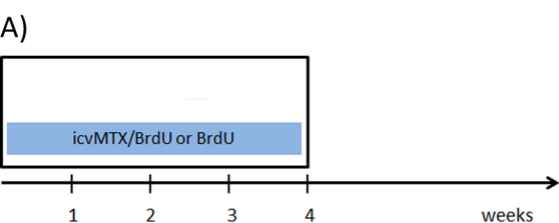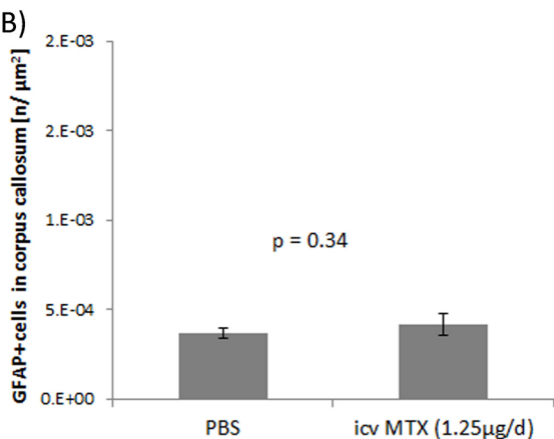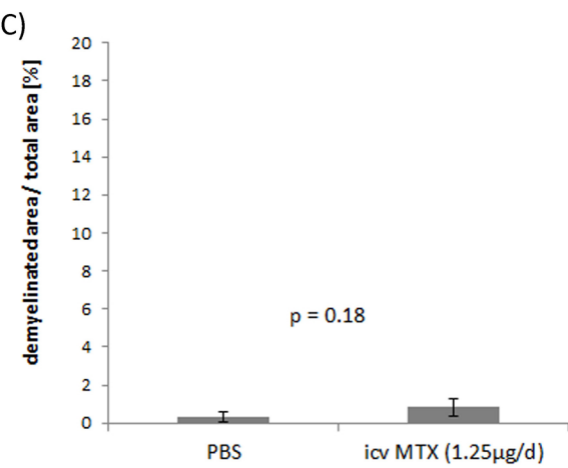

Supplement: Supplementary Figure 1 — A 4-week icv MTX administration does not have detrimental side effects. (A) Experimental setup: Eight mice per group were treated with icv MTX. Another eight mice received PBS intraventricularly. (B) Quantitative analyses of numbers of GFAP+ astrocytes in corpus callosum. Ten brains slices per mouse were analyzed. (C) Quantitative analyses of numbers of demyelination in corpus callosum. Ten brains slices per mouse were analyzed. Error bars represent standard deviations. [file Presentation1.PDF]

A)

GFAP DAPI

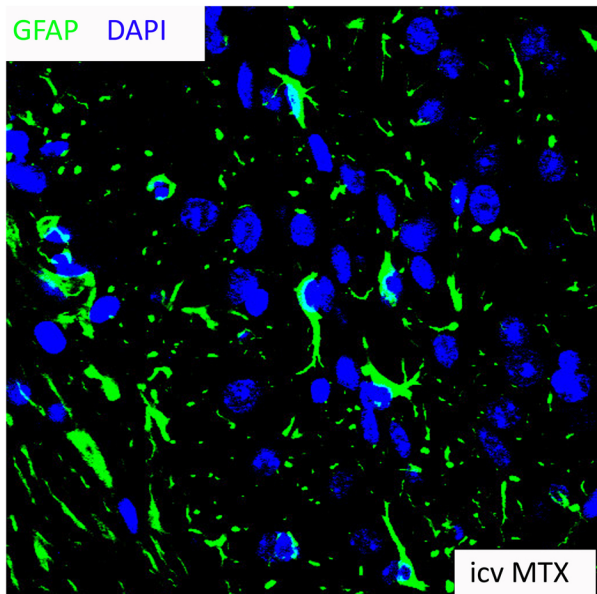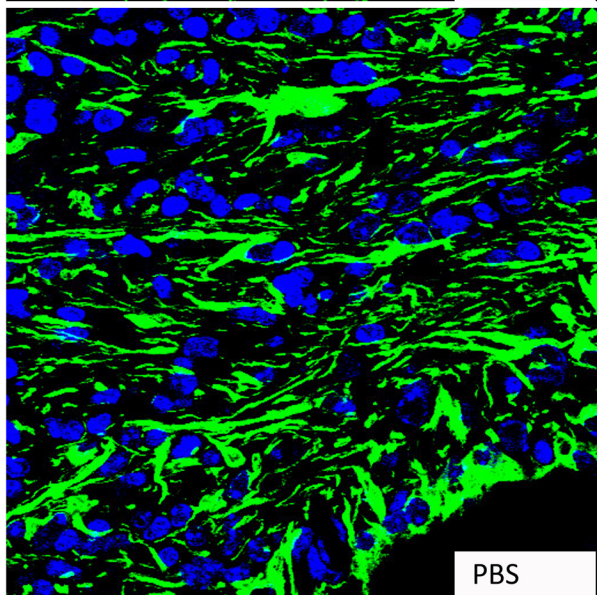

B)

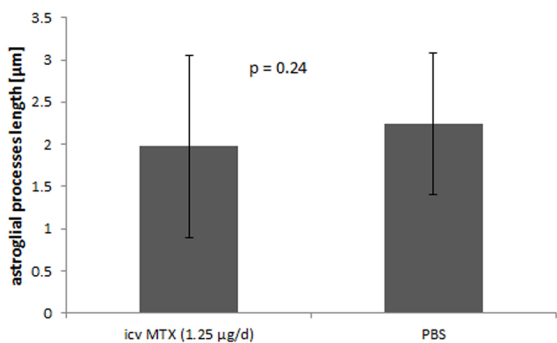

Supplement: Supplementary Figure 2 — Methotrexate does not affect the length of astroglial processes in the corpus callosum. (A) Top: Representative image of corpus callosum of PBS- treated mouse (600× magnification). Bottom: Representative image of corpus callosum of icv MTX-treated mouse (600× magnification). (B) Quantitative analyses of the length of astroglial processes. Six brains slices per mouse were analyzed. Error bars represent standard deviations. [file Presentation2.PDF]
