# Supplementary material for: Effects of intraventricular methotrexate administration on Cuprizone-induced demyelination in mice
Source: Front Mol Neurosci. 2013 Oct 16;6:34. doi: 10.3389/fnmol.2013.00034 (PMC3797440; doi:10.3389/fnmol.2013.00034)
Supplement: Supplementary Table 1 — Genes differentially expressed by CSF cells of ITMTX treated MS patients compared to untreated MS patients. 794 probe sets were upregulated in ITMTX-treated MS patients and 802 downregulated (p ≤ 0.05). [file DataSheet1.DOCX]

| **Affy probe set** | **Symbol** | **Name** | **Fold Change (ITMTX/untreated)** | **AV signal-untreated** | **Stdev** | **AV signal-ITMTX** | **Stdev** | **p-value** |
| --- | --- | --- | --- | --- | --- | --- | --- | --- |
| [236776_at](https://www.affymetrix.com/LinkServlet?probeset=236776_at) | [NA](http://www.ncbi.nlm.nih.gov/entrez/query.fcgi?cmd=search&db=gene&term=NA) | NA | **0.312** | **74.156** | 88.745 | **23.150** | 32.825 | 2.350E-03 |
| [215217_at](https://www.affymetrix.com/LinkServlet?probeset=215217_at) | [IGKC](http://www.ncbi.nlm.nih.gov/entrez/query.fcgi?cmd=search&db=gene&term=IGKC) | immunoglobulin kappa constant | **0.344** | **1390.940** | 1295.267 | **478.759** | 864.554 | 1.306E-02 |
| [217281_x_at](https://www.affymetrix.com/LinkServlet?probeset=217281_x_at) | [IGH@](mailto:IGH@) | immunoglobulin heavy locus | **0.344** | **220.565** | 255.974 | **75.959** | 109.799 | 3.850E-02 |
| [216829_at](https://www.affymetrix.com/LinkServlet?probeset=216829_at) | [IGK@](mailto:IGK@) | immunoglobulin kappa locus | **0.352** | **40.410** | 38.601 | **14.244** | 13.603 | 2.564E-02 |
| [243064_at](https://www.affymetrix.com/LinkServlet?probeset=243064_at) | [NA](http://www.ncbi.nlm.nih.gov/entrez/query.fcgi?cmd=search&db=gene&term=NA) | NA | **0.366** | **50.902** | 44.623 | **18.613** | 12.594 | 6.624E-03 |
| [216401_x_at](https://www.affymetrix.com/LinkServlet?probeset=216401_x_at) | [NA](http://www.ncbi.nlm.nih.gov/entrez/query.fcgi?cmd=search&db=gene&term=NA) | NA | **0.370** | **456.311** | 443.598 | **169.012** | 212.246 | 3.959E-02 |
| [211644_x_at](https://www.affymetrix.com/LinkServlet?probeset=211644_x_at) | [IGK@](mailto:IGK@) | immunoglobulin kappa locus | **0.382** | **1676.012** | 1185.991 | **639.485** | 1078.880 | 7.861E-03 |
| [238620_at](https://www.affymetrix.com/LinkServlet?probeset=238620_at) | [NA](http://www.ncbi.nlm.nih.gov/entrez/query.fcgi?cmd=search&db=gene&term=NA) | NA | **0.389** | **140.817** | 202.641 | **54.719** | 34.428 | 1.377E-02 |
| [236125_at](https://www.affymetrix.com/LinkServlet?probeset=236125_at) | [NA](http://www.ncbi.nlm.nih.gov/entrez/query.fcgi?cmd=search&db=gene&term=NA) | NA | **0.396** | **55.844** | 45.319 | **22.122** | 13.420 | 5.667E-04 |
| [201286_at](https://www.affymetrix.com/LinkServlet?probeset=201286_at) | [SDC1](http://www.ncbi.nlm.nih.gov/entrez/query.fcgi?cmd=search&db=gene&term=SDC1) | syndecan 1 | **0.398** | **410.653** | 331.988 | **163.553** | 268.695 | 6.331E-03 |
| [217036_at](https://www.affymetrix.com/LinkServlet?probeset=217036_at) | [LOC100293679](http://www.ncbi.nlm.nih.gov/entrez/query.fcgi?cmd=search&db=gene&term=LOC100293679) | hypothetical LOC100293679 | **0.411** | **77.656** | 72.155 | **31.922** | 42.139 | 8.053E-03 |
| [228551_at](https://www.affymetrix.com/LinkServlet?probeset=228551_at) | [DENND5B](http://www.ncbi.nlm.nih.gov/entrez/query.fcgi?cmd=search&db=gene&term=DENND5B) | DENN/MADD domain containing 5B | **0.411** | **84.743** | 117.686 | **34.868** | 52.008 | 1.886E-02 |
| [217480_x_at](https://www.affymetrix.com/LinkServlet?probeset=217480_x_at) | [NA](http://www.ncbi.nlm.nih.gov/entrez/query.fcgi?cmd=search&db=gene&term=NA) | NA | **0.414** | **1020.989** | 883.013 | **422.529** | 683.248 | 3.489E-02 |
| [206693_at](https://www.affymetrix.com/LinkServlet?probeset=206693_at) | [IL7](http://www.ncbi.nlm.nih.gov/entrez/query.fcgi?cmd=search&db=gene&term=IL7) | interleukin 7 | **0.418** | **27.503** | 26.091 | **11.500** | 5.479 | 1.100E-02 |
| [224188_s_at](https://www.affymetrix.com/LinkServlet?probeset=224188_s_at) | [XPNPEP3](http://www.ncbi.nlm.nih.gov/entrez/query.fcgi?cmd=search&db=gene&term=XPNPEP3) | X-prolyl aminopeptidase (aminopeptidase P) 3, putative | **0.425** | **43.783** | 24.946 | **18.587** | 10.258 | 8.127E-03 |
| [1570007_at](https://www.affymetrix.com/LinkServlet?probeset=1570007_at) | [LRRC8C](http://www.ncbi.nlm.nih.gov/entrez/query.fcgi?cmd=search&db=gene&term=LRRC8C) | leucine rich repeat containing 8 family, member C | **0.427** | **23.734** | 22.941 | **10.137** | 4.848 | 1.245E-02 |
| [238557_at](https://www.affymetrix.com/LinkServlet?probeset=238557_at) | [LOC100144603](http://www.ncbi.nlm.nih.gov/entrez/query.fcgi?cmd=search&db=gene&term=LOC100144603) | hypothetical transcript | **0.440** | **17.782** | 23.466 | **7.822** | 2.900 | 1.866E-02 |
| [207641_at](https://www.affymetrix.com/LinkServlet?probeset=207641_at) | [TNFRSF13B](http://www.ncbi.nlm.nih.gov/entrez/query.fcgi?cmd=search&db=gene&term=TNFRSF13B) | tumor necrosis factor receptor superfamily, member 13B | **0.441** | **33.145** | 27.776 | **14.602** | 14.468 | 1.254E-02 |
| [205476_at](https://www.affymetrix.com/LinkServlet?probeset=205476_at) | [CCL20](http://www.ncbi.nlm.nih.gov/entrez/query.fcgi?cmd=search&db=gene&term=CCL20) | chemokine (C-C motif) ligand 20 | **0.442** | **35.153** | 24.047 | **15.550** | 13.363 | 1.042E-02 |
| [227181_at](https://www.affymetrix.com/LinkServlet?probeset=227181_at) | [LNP1](http://www.ncbi.nlm.nih.gov/entrez/query.fcgi?cmd=search&db=gene&term=LNP1) | leukemia NUP98 fusion partner 1 | **0.447** | **81.473** | 111.453 | **36.383** | 24.982 | 4.597E-02 |
| [215949_x_at](https://www.affymetrix.com/LinkServlet?probeset=215949_x_at) | [NA](http://www.ncbi.nlm.nih.gov/entrez/query.fcgi?cmd=search&db=gene&term=NA) | NA | **0.448** | **86.441** | 100.799 | **38.753** | 36.390 | 4.702E-02 |
| [1557736_at](https://www.affymetrix.com/LinkServlet?probeset=1557736_at) | [NKTR](http://www.ncbi.nlm.nih.gov/entrez/query.fcgi?cmd=search&db=gene&term=NKTR) | natural killer-tumor recognition sequence | **0.453** | **19.690** | 15.285 | **8.912** | 4.758 | 1.668E-02 |
| [229721_x_at](https://www.affymetrix.com/LinkServlet?probeset=229721_x_at) | [DERL3](http://www.ncbi.nlm.nih.gov/entrez/query.fcgi?cmd=search&db=gene&term=DERL3) | Der1-like domain family, member 3 | **0.455** | **181.942** | 191.731 | **82.786** | 104.466 | 4.535E-02 |
| [1569004_at](https://www.affymetrix.com/LinkServlet?probeset=1569004_at) | [NA](http://www.ncbi.nlm.nih.gov/entrez/query.fcgi?cmd=search&db=gene&term=NA) | NA | **0.465** | **49.283** | 31.672 | **22.917** | 13.747 | 1.941E-02 |
| [217157_x_at](https://www.affymetrix.com/LinkServlet?probeset=217157_x_at) | [NA](http://www.ncbi.nlm.nih.gov/entrez/query.fcgi?cmd=search&db=gene&term=NA) | NA | **0.468** | **636.630** | 664.585 | **297.921** | 539.061 | 3.568E-02 |
| [1569927_at](https://www.affymetrix.com/LinkServlet?probeset=1569927_at) | [NA](http://www.ncbi.nlm.nih.gov/entrez/query.fcgi?cmd=search&db=gene&term=NA) | NA | **0.469** | **21.070** | 16.693 | **9.884** | 7.995 | 2.504E-02 |
| [203755_at](https://www.affymetrix.com/LinkServlet?probeset=203755_at) | [BUB1B](http://www.ncbi.nlm.nih.gov/entrez/query.fcgi?cmd=search&db=gene&term=BUB1B) | budding uninhibited by benzimidazoles 1 homolog beta (yeast) | **0.471** | **63.321** | 50.522 | **29.845** | 33.417 | 7.755E-03 |
| [1555420_a_at](https://www.affymetrix.com/LinkServlet?probeset=1555420_a_at) | [KLF7](http://www.ncbi.nlm.nih.gov/entrez/query.fcgi?cmd=search&db=gene&term=KLF7) | Kruppel-like factor 7 (ubiquitous) | **0.477** | **20.641** | 15.001 | **9.842** | 4.774 | 7.244E-03 |
| [217034_at](https://www.affymetrix.com/LinkServlet?probeset=217034_at) | [NTN3](http://www.ncbi.nlm.nih.gov/entrez/query.fcgi?cmd=search&db=gene&term=NTN3) | netrin 3 | **0.478** | **45.561** | 46.617 | **21.796** | 11.434 | 4.267E-02 |
| [203395_s_at](https://www.affymetrix.com/LinkServlet?probeset=203395_s_at) | [HES1](http://www.ncbi.nlm.nih.gov/entrez/query.fcgi?cmd=search&db=gene&term=HES1) | hairy and enhancer of split 1, (Drosophila) | **0.479** | **19.751** | 16.550 | **9.459** | 7.274 | 3.219E-02 |
| [201287_s_at](https://www.affymetrix.com/LinkServlet?probeset=201287_s_at) | [SDC1](http://www.ncbi.nlm.nih.gov/entrez/query.fcgi?cmd=search&db=gene&term=SDC1) | syndecan 1 | **0.480** | **202.257** | 219.463 | **97.083** | 145.780 | 3.287E-02 |
| [1558208_at](https://www.affymetrix.com/LinkServlet?probeset=1558208_at) | [TARDBP](http://www.ncbi.nlm.nih.gov/entrez/query.fcgi?cmd=search&db=gene&term=TARDBP) | TAR DNA binding protein | **0.480** | **139.107** | 98.278 | **66.799** | 39.723 | 1.682E-02 |
| [206641_at](https://www.affymetrix.com/LinkServlet?probeset=206641_at) | [TNFRSF17](http://www.ncbi.nlm.nih.gov/entrez/query.fcgi?cmd=search&db=gene&term=TNFRSF17) | tumor necrosis factor receptor superfamily, member 17 | **0.481** | **552.090** | 502.439 | **265.408** | 387.681 | 4.836E-02 |
| [228898_s_at](https://www.affymetrix.com/LinkServlet?probeset=228898_s_at) | [NA](http://www.ncbi.nlm.nih.gov/entrez/query.fcgi?cmd=search&db=gene&term=NA) | NA | **0.481** | **288.645** | 271.688 | **138.776** | 167.007 | 4.429E-02 |
| [235092_at](https://www.affymetrix.com/LinkServlet?probeset=235092_at) | [NA](http://www.ncbi.nlm.nih.gov/entrez/query.fcgi?cmd=search&db=gene&term=NA) | NA | **0.484** | **22.960** | 13.406 | **11.114** | 12.212 | 3.146E-03 |
| [237461_at](https://www.affymetrix.com/LinkServlet?probeset=237461_at) | [NLRP7](http://www.ncbi.nlm.nih.gov/entrez/query.fcgi?cmd=search&db=gene&term=NLRP7) | NLR family, pyrin domain containing 7 | **0.486** | **456.018** | 269.260 | **221.570** | 229.502 | 4.735E-02 |
| [203986_at](https://www.affymetrix.com/LinkServlet?probeset=203986_at) | [STBD1](http://www.ncbi.nlm.nih.gov/entrez/query.fcgi?cmd=search&db=gene&term=STBD1) | starch binding domain 1 | **0.487** | **54.883** | 29.865 | **26.732** | 14.723 | 1.455E-03 |
| [207090_x_at](https://www.affymetrix.com/LinkServlet?probeset=207090_x_at) | [ZFP30](http://www.ncbi.nlm.nih.gov/entrez/query.fcgi?cmd=search&db=gene&term=ZFP30) | zinc finger protein 30 homolog (mouse) | **0.489** | **78.516** | 46.744 | **38.365** | 21.005 | 1.533E-03 |
| [1557193_at](https://www.affymetrix.com/LinkServlet?probeset=1557193_at) | [NA](http://www.ncbi.nlm.nih.gov/entrez/query.fcgi?cmd=search&db=gene&term=NA) | NA | **0.489** | **28.699** | 18.683 | **14.025** | 9.905 | 2.775E-02 |
| [215565_at](https://www.affymetrix.com/LinkServlet?probeset=215565_at) | [DTNB](http://www.ncbi.nlm.nih.gov/entrez/query.fcgi?cmd=search&db=gene&term=DTNB) | dystrobrevin, beta | **0.493** | **258.271** | 310.574 | **127.291** | 190.026 | 2.859E-02 |
| [1559232_a_at](https://www.affymetrix.com/LinkServlet?probeset=1559232_a_at) | [NA](http://www.ncbi.nlm.nih.gov/entrez/query.fcgi?cmd=search&db=gene&term=NA) | NA | **0.496** | **18.064** | 9.827 | **8.963** | 3.828 | 4.250E-03 |
| [1560792_at](https://www.affymetrix.com/LinkServlet?probeset=1560792_at) | [NA](http://www.ncbi.nlm.nih.gov/entrez/query.fcgi?cmd=search&db=gene&term=NA) | NA | **0.496** | **50.266** | 27.562 | **24.945** | 10.267 | 1.824E-03 |
| [208651_x_at](https://www.affymetrix.com/LinkServlet?probeset=208651_x_at) | [CD24](http://www.ncbi.nlm.nih.gov/entrez/query.fcgi?cmd=search&db=gene&term=CD24) | CD24 molecule | **0.497** | **158.819** | 140.298 | **78.953** | 55.238 | 3.941E-02 |
| [213391_at](https://www.affymetrix.com/LinkServlet?probeset=213391_at) | [DPY19L4](http://www.ncbi.nlm.nih.gov/entrez/query.fcgi?cmd=search&db=gene&term=DPY19L4) | dpy-19-like 4 (C. elegans) | **0.499** | **35.499** | 25.556 | **17.705** | 10.494 | 2.688E-02 |
| [214669_x_at](https://www.affymetrix.com/LinkServlet?probeset=214669_x_at) | [IGKC](http://www.ncbi.nlm.nih.gov/entrez/query.fcgi?cmd=search&db=gene&term=IGKC) | immunoglobulin kappa constant | **0.499** | **2125.872** | 1087.966 | **1060.998** | 920.774 | 2.640E-02 |
| [219671_at](https://www.affymetrix.com/LinkServlet?probeset=219671_at) | [HPCAL4](http://www.ncbi.nlm.nih.gov/entrez/query.fcgi?cmd=search&db=gene&term=HPCAL4) | hippocalcin like 4 | **0.499** | **33.495** | 19.694 | **16.724** | 10.762 | 1.823E-02 |
| [242741_x_at](https://www.affymetrix.com/LinkServlet?probeset=242741_x_at) | [NA](http://www.ncbi.nlm.nih.gov/entrez/query.fcgi?cmd=search&db=gene&term=NA) | NA | **0.503** | **32.090** | 23.959 | **16.155** | 16.542 | 2.178E-02 |
| [203129_s_at](https://www.affymetrix.com/LinkServlet?probeset=203129_s_at) | [KIF5C](http://www.ncbi.nlm.nih.gov/entrez/query.fcgi?cmd=search&db=gene&term=KIF5C) | kinesin family member 5C | **0.507** | **19.015** | 12.512 | **9.639** | 4.073 | 3.248E-02 |
| [227941_at](https://www.affymetrix.com/LinkServlet?probeset=227941_at) | [LOC339803](http://www.ncbi.nlm.nih.gov/entrez/query.fcgi?cmd=search&db=gene&term=LOC339803) | hypothetical LOC339803 | **0.508** | **44.439** | 35.314 | **22.574** | 32.733 | 2.550E-02 |
| [1558275_at](https://www.affymetrix.com/LinkServlet?probeset=1558275_at) | [NA](http://www.ncbi.nlm.nih.gov/entrez/query.fcgi?cmd=search&db=gene&term=NA) | NA | **0.512** | **23.555** | 13.611 | **12.062** | 8.867 | 1.729E-02 |
| [235505_s_at](https://www.affymetrix.com/LinkServlet?probeset=235505_s_at) | [NA](http://www.ncbi.nlm.nih.gov/entrez/query.fcgi?cmd=search&db=gene&term=NA) | NA | **0.515** | **62.858** | 23.127 | **32.343** | 19.886 | 1.073E-03 |
| [211643_x_at](https://www.affymetrix.com/LinkServlet?probeset=211643_x_at) | [NA](http://www.ncbi.nlm.nih.gov/entrez/query.fcgi?cmd=search&db=gene&term=NA) | NA | **0.515** | **795.340** | 716.411 | **409.642** | 754.832 | 2.036E-02 |
| [1565876_x_at](https://www.affymetrix.com/LinkServlet?probeset=1565876_x_at) | [NA](http://www.ncbi.nlm.nih.gov/entrez/query.fcgi?cmd=search&db=gene&term=NA) | NA | **0.517** | **19.467** | 16.081 | **10.071** | 4.785 | 4.883E-02 |
| [1560916_a_at](https://www.affymetrix.com/LinkServlet?probeset=1560916_a_at) | [DPY19L1](http://www.ncbi.nlm.nih.gov/entrez/query.fcgi?cmd=search&db=gene&term=DPY19L1) | dpy-19-like 1 (C. elegans) | **0.519** | **34.730** | 23.972 | **18.035** | 7.140 | 3.149E-02 |
| [226649_at](https://www.affymetrix.com/LinkServlet?probeset=226649_at) | [PANK1](http://www.ncbi.nlm.nih.gov/entrez/query.fcgi?cmd=search&db=gene&term=PANK1) | pantothenate kinase 1 | **0.520** | **35.493** | 21.878 | **18.459** | 12.272 | 4.231E-02 |
| [1557576_at](https://www.affymetrix.com/LinkServlet?probeset=1557576_at) | [PAQR3](http://www.ncbi.nlm.nih.gov/entrez/query.fcgi?cmd=search&db=gene&term=PAQR3) | progestin and adipoQ receptor family member III | **0.520** | **18.582** | 12.696 | **9.667** | 5.048 | 1.086E-02 |
| [237262_at](https://www.affymetrix.com/LinkServlet?probeset=237262_at) | [NA](http://www.ncbi.nlm.nih.gov/entrez/query.fcgi?cmd=search&db=gene&term=NA) | NA | **0.521** | **12.480** | 11.730 | **6.496** | 1.588 | 2.859E-02 |
| [215286_s_at](https://www.affymetrix.com/LinkServlet?probeset=215286_s_at) | [PHTF2](http://www.ncbi.nlm.nih.gov/entrez/query.fcgi?cmd=search&db=gene&term=PHTF2) | putative homeodomain transcription factor 2 | **0.521** | **37.399** | 31.416 | **19.473** | 10.982 | 4.334E-02 |
| [235826_at](https://www.affymetrix.com/LinkServlet?probeset=235826_at) | [NA](http://www.ncbi.nlm.nih.gov/entrez/query.fcgi?cmd=search&db=gene&term=NA) | NA | **0.523** | **46.687** | 37.612 | **24.428** | 17.546 | 4.233E-02 |
| [223089_at](https://www.affymetrix.com/LinkServlet?probeset=223089_at) | [VEZT](http://www.ncbi.nlm.nih.gov/entrez/query.fcgi?cmd=search&db=gene&term=VEZT) | vezatin, adherens junctions transmembrane protein | **0.526** | **31.390** | 10.704 | **16.510** | 8.308 | 5.578E-04 |
| [1569251_a_at](https://www.affymetrix.com/LinkServlet?probeset=1569251_a_at) | [ZNF333](http://www.ncbi.nlm.nih.gov/entrez/query.fcgi?cmd=search&db=gene&term=ZNF333) | zinc finger protein 333 | **0.528** | **16.943** | 12.220 | **8.943** | 5.972 | 3.972E-02 |
| [244778_x_at](https://www.affymetrix.com/LinkServlet?probeset=244778_x_at) | [NA](http://www.ncbi.nlm.nih.gov/entrez/query.fcgi?cmd=search&db=gene&term=NA) | NA | **0.528** | **49.379** | 28.778 | **26.069** | 19.043 | 3.044E-02 |
| [1565875_at](https://www.affymetrix.com/LinkServlet?probeset=1565875_at) | [NA](http://www.ncbi.nlm.nih.gov/entrez/query.fcgi?cmd=search&db=gene&term=NA) | NA | **0.529** | **22.116** | 18.161 | **11.699** | 6.552 | 3.230E-02 |
| [223565_at](https://www.affymetrix.com/LinkServlet?probeset=223565_at) | [MZB1](http://www.ncbi.nlm.nih.gov/entrez/query.fcgi?cmd=search&db=gene&term=MZB1) | marginal zone B and B1 cell-specific protein | **0.530** | **389.721** | 407.752 | **206.743** | 311.341 | 4.495E-02 |
| [59433_at](https://www.affymetrix.com/LinkServlet?probeset=59433_at) | [LOC389906](http://www.ncbi.nlm.nih.gov/entrez/query.fcgi?cmd=search&db=gene&term=LOC389906) | hypothetical LOC389906 | **0.532** | **13.752** | 8.320 | **7.310** | 1.968 | 1.617E-02 |
| [215845_x_at](https://www.affymetrix.com/LinkServlet?probeset=215845_x_at) | [NA](http://www.ncbi.nlm.nih.gov/entrez/query.fcgi?cmd=search&db=gene&term=NA) | NA | **0.534** | **27.260** | 14.123 | **14.545** | 5.050 | 7.153E-03 |
| [229455_at](https://www.affymetrix.com/LinkServlet?probeset=229455_at) | [NA](http://www.ncbi.nlm.nih.gov/entrez/query.fcgi?cmd=search&db=gene&term=NA) | NA | **0.534** | **24.613** | 11.235 | **13.141** | 9.084 | 2.464E-03 |
| [1554665_at](https://www.affymetrix.com/LinkServlet?probeset=1554665_at) | [ZNF586](http://www.ncbi.nlm.nih.gov/entrez/query.fcgi?cmd=search&db=gene&term=ZNF586) | zinc finger protein 586 | **0.535** | **23.655** | 15.788 | **12.645** | 7.108 | 4.392E-02 |
| [243160_at](https://www.affymetrix.com/LinkServlet?probeset=243160_at) | [NA](http://www.ncbi.nlm.nih.gov/entrez/query.fcgi?cmd=search&db=gene&term=NA) | NA | **0.535** | **13.397** | 8.317 | **7.162** | 2.231 | 2.263E-02 |
| [203187_at](https://www.affymetrix.com/LinkServlet?probeset=203187_at) | [DOCK1](http://www.ncbi.nlm.nih.gov/entrez/query.fcgi?cmd=search&db=gene&term=DOCK1) | dedicator of cytokinesis 1 | **0.535** | **12.751** | 9.158 | **6.824** | 1.815 | 2.087E-02 |
| [37793_r_at](https://www.affymetrix.com/LinkServlet?probeset=37793_r_at) | [RAD51L3](http://www.ncbi.nlm.nih.gov/entrez/query.fcgi?cmd=search&db=gene&term=RAD51L3) | RAD51-like 3 (S. cerevisiae) | **0.536** | **48.408** | 17.957 | **25.924** | 19.673 | 8.896E-04 |
| [221286_s_at](https://www.affymetrix.com/LinkServlet?probeset=221286_s_at) | [MZB1](http://www.ncbi.nlm.nih.gov/entrez/query.fcgi?cmd=search&db=gene&term=MZB1) | marginal zone B and B1 cell-specific protein | **0.536** | **433.928** | 431.940 | **232.385** | 337.481 | 4.095E-02 |
| [215310_at](https://www.affymetrix.com/LinkServlet?probeset=215310_at) | [APC](http://www.ncbi.nlm.nih.gov/entrez/query.fcgi?cmd=search&db=gene&term=APC) | adenomatous polyposis coli | **0.536** | **63.669** | 32.687 | **34.156** | 26.893 | 1.647E-02 |
| [243733_at](https://www.affymetrix.com/LinkServlet?probeset=243733_at) | [NA](http://www.ncbi.nlm.nih.gov/entrez/query.fcgi?cmd=search&db=gene&term=NA) | NA | **0.538** | **12.637** | 10.196 | **6.798** | 1.108 | 1.921E-02 |
| [234937_x_at](https://www.affymetrix.com/LinkServlet?probeset=234937_x_at) | [ZFP28](http://www.ncbi.nlm.nih.gov/entrez/query.fcgi?cmd=search&db=gene&term=ZFP28) | zinc finger protein 28 homolog (mouse) | **0.539** | **21.886** | 14.015 | **11.803** | 11.051 | 3.195E-02 |
| [237625_s_at](https://www.affymetrix.com/LinkServlet?probeset=237625_s_at) | [IGKC](http://www.ncbi.nlm.nih.gov/entrez/query.fcgi?cmd=search&db=gene&term=IGKC) | immunoglobulin kappa constant | **0.546** | **124.343** | 113.048 | **67.853** | 131.047 | 3.395E-02 |
| [213556_at](https://www.affymetrix.com/LinkServlet?probeset=213556_at) | [LOC390940](http://www.ncbi.nlm.nih.gov/entrez/query.fcgi?cmd=search&db=gene&term=LOC390940) | hypothetical protein LOC390940 | **0.547** | **30.646** | 18.036 | **16.763** | 8.597 | 8.290E-03 |
| [205049_s_at](https://www.affymetrix.com/LinkServlet?probeset=205049_s_at) | [CD79A](http://www.ncbi.nlm.nih.gov/entrez/query.fcgi?cmd=search&db=gene&term=CD79A) | CD79a molecule, immunoglobulin-associated alpha | **0.547** | **251.984** | 219.047 | **137.842** | 159.145 | 4.362E-02 |
| [234300_s_at](https://www.affymetrix.com/LinkServlet?probeset=234300_s_at) | [ZFP28](http://www.ncbi.nlm.nih.gov/entrez/query.fcgi?cmd=search&db=gene&term=ZFP28) | zinc finger protein 28 homolog (mouse) | **0.547** | **22.296** | 15.029 | **12.200** | 11.679 | 2.185E-02 |
| [1561959_x_at](https://www.affymetrix.com/LinkServlet?probeset=1561959_x_at) | [NA](http://www.ncbi.nlm.nih.gov/entrez/query.fcgi?cmd=search&db=gene&term=NA) | NA | **0.548** | **13.503** | 8.106 | **7.397** | 2.447 | 2.345E-02 |
| [236439_at](https://www.affymetrix.com/LinkServlet?probeset=236439_at) | [NA](http://www.ncbi.nlm.nih.gov/entrez/query.fcgi?cmd=search&db=gene&term=NA) | NA | **0.548** | **145.007** | 67.690 | **79.496** | 64.354 | 1.591E-02 |
| [226122_at](https://www.affymetrix.com/LinkServlet?probeset=226122_at) | [PLEKHG1](http://www.ncbi.nlm.nih.gov/entrez/query.fcgi?cmd=search&db=gene&term=PLEKHG1) | pleckstrin homology domain containing, family G (with RhoGef domain) member 1 | **0.549** | **56.978** | 26.076 | **31.272** | 22.091 | 8.222E-03 |
| [203820_s_at](https://www.affymetrix.com/LinkServlet?probeset=203820_s_at) | [IGF2BP3](http://www.ncbi.nlm.nih.gov/entrez/query.fcgi?cmd=search&db=gene&term=IGF2BP3) | insulin-like growth factor 2 mRNA binding protein 3 | **0.549** | **22.434** | 12.876 | **12.315** | 10.784 | 1.562E-02 |
| [229090_at](https://www.affymetrix.com/LinkServlet?probeset=229090_at) | [LOC220930](http://www.ncbi.nlm.nih.gov/entrez/query.fcgi?cmd=search&db=gene&term=LOC220930) | hypothetical LOC220930 | **0.550** | **46.879** | 26.590 | **25.765** | 14.412 | 4.220E-02 |
| [207583_at](https://www.affymetrix.com/LinkServlet?probeset=207583_at) | [ABCD2](http://www.ncbi.nlm.nih.gov/entrez/query.fcgi?cmd=search&db=gene&term=ABCD2) | ATP-binding cassette, sub-family D (ALD), member 2 | **0.550** | **54.513** | 27.646 | **29.974** | 25.985 | 6.347E-03 |
| [266_s_at](https://www.affymetrix.com/LinkServlet?probeset=266_s_at) | [CD24](http://www.ncbi.nlm.nih.gov/entrez/query.fcgi?cmd=search&db=gene&term=CD24) | CD24 molecule | **0.552** | **266.299** | 227.328 | **146.989** | 126.503 | 4.246E-02 |
| [222743_s_at](https://www.affymetrix.com/LinkServlet?probeset=222743_s_at) | [C11orf71](http://www.ncbi.nlm.nih.gov/entrez/query.fcgi?cmd=search&db=gene&term=C11orf71) | chromosome 11 open reading frame 71 | **0.553** | **61.520** | 48.576 | **34.013** | 28.508 | 1.431E-02 |
| [209645_s_at](https://www.affymetrix.com/LinkServlet?probeset=209645_s_at) | [ALDH1B1](http://www.ncbi.nlm.nih.gov/entrez/query.fcgi?cmd=search&db=gene&term=ALDH1B1) | aldehyde dehydrogenase 1 family, member B1 | **0.553** | **13.530** | 9.736 | **7.487** | 3.217 | 2.114E-02 |
| [230520_at](https://www.affymetrix.com/LinkServlet?probeset=230520_at) | [AIG1](http://www.ncbi.nlm.nih.gov/entrez/query.fcgi?cmd=search&db=gene&term=AIG1) | androgen-induced 1 | **0.555** | **12.135** | 7.870 | **6.729** | 2.417 | 1.459E-02 |
| [208456_s_at](https://www.affymetrix.com/LinkServlet?probeset=208456_s_at) | [RRAS2](http://www.ncbi.nlm.nih.gov/entrez/query.fcgi?cmd=search&db=gene&term=RRAS2) | related RAS viral (r-ras) oncogene homolog 2 | **0.556** | **34.822** | 23.827 | **19.348** | 10.782 | 3.744E-02 |
| [241858_at](https://www.affymetrix.com/LinkServlet?probeset=241858_at) | [FPGT](http://www.ncbi.nlm.nih.gov/entrez/query.fcgi?cmd=search&db=gene&term=FPGT) | fucose-1-phosphate guanylyltransferase | **0.556** | **26.391** | 10.928 | **14.680** | 5.986 | 5.231E-03 |
| [216491_x_at](https://www.affymetrix.com/LinkServlet?probeset=216491_x_at) | [IGHM](http://www.ncbi.nlm.nih.gov/entrez/query.fcgi?cmd=search&db=gene&term=IGHM) | immunoglobulin heavy constant mu | **0.557** | **211.610** | 209.949 | **117.900** | 202.203 | 4.854E-02 |
| [228655_at](https://www.affymetrix.com/LinkServlet?probeset=228655_at) | [NA](http://www.ncbi.nlm.nih.gov/entrez/query.fcgi?cmd=search&db=gene&term=NA) | NA | **0.560** | **69.075** | 30.942 | **38.660** | 29.000 | 4.129E-03 |
| [222886_at](https://www.affymetrix.com/LinkServlet?probeset=222886_at) | [NSUN3](http://www.ncbi.nlm.nih.gov/entrez/query.fcgi?cmd=search&db=gene&term=NSUN3) | NOP2/Sun domain family, member 3 | **0.561** | **101.916** | 33.584 | **57.196** | 25.590 | 1.224E-03 |
| [243111_at](https://www.affymetrix.com/LinkServlet?probeset=243111_at) | [ENTPD1](http://www.ncbi.nlm.nih.gov/entrez/query.fcgi?cmd=search&db=gene&term=ENTPD1) | ectonucleoside triphosphate diphosphohydrolase 1 | **0.562** | **24.177** | 18.211 | **13.595** | 7.957 | 3.390E-02 |
| [1560209_at](https://www.affymetrix.com/LinkServlet?probeset=1560209_at) | [NA](http://www.ncbi.nlm.nih.gov/entrez/query.fcgi?cmd=search&db=gene&term=NA) | NA | **0.563** | **18.022** | 10.139 | **10.147** | 8.175 | 2.299E-02 |
| [207926_at](https://www.affymetrix.com/LinkServlet?probeset=207926_at) | [GP5](http://www.ncbi.nlm.nih.gov/entrez/query.fcgi?cmd=search&db=gene&term=GP5) | glycoprotein V (platelet) | **0.563** | **24.559** | 12.231 | **13.838** | 11.133 | 2.065E-02 |
| [215859_at](https://www.affymetrix.com/LinkServlet?probeset=215859_at) | [NCLN](http://www.ncbi.nlm.nih.gov/entrez/query.fcgi?cmd=search&db=gene&term=NCLN) | nicalin | **0.564** | **21.636** | 12.942 | **12.210** | 6.820 | 4.477E-02 |
| [1556498_at](https://www.affymetrix.com/LinkServlet?probeset=1556498_at) | [FAM69A](http://www.ncbi.nlm.nih.gov/entrez/query.fcgi?cmd=search&db=gene&term=FAM69A) | family with sequence similarity 69, member A | **0.566** | **25.337** | 13.317 | **14.340** | 7.614 | 1.817E-02 |
| [215374_at](https://www.affymetrix.com/LinkServlet?probeset=215374_at) | [PAPOLA](http://www.ncbi.nlm.nih.gov/entrez/query.fcgi?cmd=search&db=gene&term=PAPOLA) | poly(A) polymerase alpha | **0.567** | **74.884** | 36.580 | **42.446** | 24.243 | 9.858E-03 |
| [224443_at](https://www.affymetrix.com/LinkServlet?probeset=224443_at) | [C1orf97](http://www.ncbi.nlm.nih.gov/entrez/query.fcgi?cmd=search&db=gene&term=C1orf97) | chromosome 1 open reading frame 97 | **0.567** | **31.760** | 19.291 | **18.007** | 10.363 | 2.485E-02 |
| [237418_at](https://www.affymetrix.com/LinkServlet?probeset=237418_at) | [CDK5RAP2](http://www.ncbi.nlm.nih.gov/entrez/query.fcgi?cmd=search&db=gene&term=CDK5RAP2) | CDK5 regulatory subunit associated protein 2 | **0.568** | **17.392** | 6.616 | **9.874** | 5.170 | 1.540E-03 |
| [244640_at](https://www.affymetrix.com/LinkServlet?probeset=244640_at) | [ZNF850](http://www.ncbi.nlm.nih.gov/entrez/query.fcgi?cmd=search&db=gene&term=ZNF850) | zinc finger protein 850 | **0.568** | **43.050** | 13.299 | **24.463** | 19.764 | 1.297E-03 |
| [240294_at](https://www.affymetrix.com/LinkServlet?probeset=240294_at) | [HIPK2](http://www.ncbi.nlm.nih.gov/entrez/query.fcgi?cmd=search&db=gene&term=HIPK2) | homeodomain interacting protein kinase 2 | **0.569** | **24.995** | 13.944 | **14.218** | 9.342 | 7.119E-03 |
| [237411_at](https://www.affymetrix.com/LinkServlet?probeset=237411_at) | [ADAMTS6](http://www.ncbi.nlm.nih.gov/entrez/query.fcgi?cmd=search&db=gene&term=ADAMTS6) | ADAM metallopeptidase with thrombospondin type 1 motif, 6 | **0.569** | **12.861** | 7.726 | **7.323** | 1.686 | 1.868E-02 |
| [229963_at](https://www.affymetrix.com/LinkServlet?probeset=229963_at) | [BEX5](http://www.ncbi.nlm.nih.gov/entrez/query.fcgi?cmd=search&db=gene&term=BEX5) | brain expressed, X-linked 5 | **0.570** | **118.770** | 70.562 | **67.665** | 33.434 | 2.460E-02 |
| [233021_at](https://www.affymetrix.com/LinkServlet?probeset=233021_at) | [LOC100505538](http://www.ncbi.nlm.nih.gov/entrez/query.fcgi?cmd=search&db=gene&term=LOC100505538) | hypothetical LOC100505538 | **0.572** | **40.008** | 35.843 | **22.886** | 15.865 | 4.715E-02 |
| [1555779_a_at](https://www.affymetrix.com/LinkServlet?probeset=1555779_a_at) | [CD79A](http://www.ncbi.nlm.nih.gov/entrez/query.fcgi?cmd=search&db=gene&term=CD79A) | CD79a molecule, immunoglobulin-associated alpha | **0.575** | **332.749** | 260.963 | **191.246** | 217.509 | 4.087E-02 |
| [1553849_at](https://www.affymetrix.com/LinkServlet?probeset=1553849_at) | [CCDC26](http://www.ncbi.nlm.nih.gov/entrez/query.fcgi?cmd=search&db=gene&term=CCDC26) | coiled-coil domain containing 26 | **0.575** | **15.526** | 8.700 | **8.933** | 3.366 | 3.956E-02 |
| [238042_at](https://www.affymetrix.com/LinkServlet?probeset=238042_at) | [NA](http://www.ncbi.nlm.nih.gov/entrez/query.fcgi?cmd=search&db=gene&term=NA) | NA | **0.577** | **30.052** | 13.528 | **17.331** | 10.611 | 9.353E-03 |
| [215946_x_at](https://www.affymetrix.com/LinkServlet?probeset=215946_x_at) | [IGLL3P](http://www.ncbi.nlm.nih.gov/entrez/query.fcgi?cmd=search&db=gene&term=IGLL3P) | immunoglobulin lambda-like polypeptide 3, pseudogene | **0.577** | **527.664** | 351.724 | **304.447** | 333.586 | 3.969E-02 |
| [211947_s_at](https://www.affymetrix.com/LinkServlet?probeset=211947_s_at) | [PRRC2C](http://www.ncbi.nlm.nih.gov/entrez/query.fcgi?cmd=search&db=gene&term=PRRC2C) | proline-rich coiled-coil 2C | **0.577** | **77.786** | 43.130 | **44.883** | 22.949 | 9.444E-03 |
| [238492_at](https://www.affymetrix.com/LinkServlet?probeset=238492_at) | [NA](http://www.ncbi.nlm.nih.gov/entrez/query.fcgi?cmd=search&db=gene&term=NA) | NA | **0.578** | **59.279** | 18.075 | **34.279** | 19.361 | 2.444E-03 |
| [238497_at](https://www.affymetrix.com/LinkServlet?probeset=238497_at) | [TMEM136](http://www.ncbi.nlm.nih.gov/entrez/query.fcgi?cmd=search&db=gene&term=TMEM136) | transmembrane protein 136 | **0.578** | **19.708** | 10.915 | **11.398** | 8.094 | 1.161E-02 |
| [239695_at](https://www.affymetrix.com/LinkServlet?probeset=239695_at) | [JAK1](http://www.ncbi.nlm.nih.gov/entrez/query.fcgi?cmd=search&db=gene&term=JAK1) | Janus kinase 1 | **0.580** | **52.988** | 23.697 | **30.729** | 16.463 | 2.647E-02 |
| [226339_at](https://www.affymetrix.com/LinkServlet?probeset=226339_at) | [TRUB1](http://www.ncbi.nlm.nih.gov/entrez/query.fcgi?cmd=search&db=gene&term=TRUB1) | TruB pseudouridine (psi) synthase homolog 1 (E. coli) | **0.581** | **86.779** | 33.380 | **50.419** | 27.077 | 2.971E-03 |
| [1558942_at](https://www.affymetrix.com/LinkServlet?probeset=1558942_at) | [ZNF765](http://www.ncbi.nlm.nih.gov/entrez/query.fcgi?cmd=search&db=gene&term=ZNF765) | zinc finger protein 765 | **0.582** | **128.412** | 32.449 | **74.758** | 20.159 | 1.990E-05 |
| [226912_at](https://www.affymetrix.com/LinkServlet?probeset=226912_at) | [ZDHHC23](http://www.ncbi.nlm.nih.gov/entrez/query.fcgi?cmd=search&db=gene&term=ZDHHC23) | zinc finger, DHHC-type containing 23 | **0.582** | **39.864** | 20.785 | **23.217** | 16.425 | 3.603E-02 |
| [242656_at](https://www.affymetrix.com/LinkServlet?probeset=242656_at) | [GTF2H1](http://www.ncbi.nlm.nih.gov/entrez/query.fcgi?cmd=search&db=gene&term=GTF2H1) | general transcription factor IIH, polypeptide 1, 62kDa | **0.584** | **31.168** | 27.137 | **18.196** | 8.466 | 4.260E-02 |
| [203990_s_at](https://www.affymetrix.com/LinkServlet?probeset=203990_s_at) | [KDM6A](http://www.ncbi.nlm.nih.gov/entrez/query.fcgi?cmd=search&db=gene&term=KDM6A) | lysine (K)-specific demethylase 6A | **0.584** | **13.555** | 9.643 | **7.922** | 3.498 | 4.309E-02 |
| [218904_s_at](https://www.affymetrix.com/LinkServlet?probeset=218904_s_at) | [C9orf40](http://www.ncbi.nlm.nih.gov/entrez/query.fcgi?cmd=search&db=gene&term=C9orf40) | chromosome 9 open reading frame 40 | **0.585** | **44.406** | 19.023 | **25.994** | 17.189 | 1.060E-02 |
| [210232_at](https://www.affymetrix.com/LinkServlet?probeset=210232_at) | [CDC42](http://www.ncbi.nlm.nih.gov/entrez/query.fcgi?cmd=search&db=gene&term=CDC42) | cell division cycle 42 (GTP binding protein, 25kDa) | **0.586** | **14.072** | 6.004 | **8.249** | 2.566 | 3.217E-03 |
| [229999_at](https://www.affymetrix.com/LinkServlet?probeset=229999_at) | [NA](http://www.ncbi.nlm.nih.gov/entrez/query.fcgi?cmd=search&db=gene&term=NA) | NA | **0.586** | **75.661** | 30.511 | **44.368** | 20.477 | 4.895E-03 |
| [234726_s_at](https://www.affymetrix.com/LinkServlet?probeset=234726_s_at) | [TMEM168](http://www.ncbi.nlm.nih.gov/entrez/query.fcgi?cmd=search&db=gene&term=TMEM168) | transmembrane protein 168 | **0.587** | **48.741** | 29.656 | **28.634** | 12.293 | 3.433E-02 |
| [240557_at](https://www.affymetrix.com/LinkServlet?probeset=240557_at) | [NA](http://www.ncbi.nlm.nih.gov/entrez/query.fcgi?cmd=search&db=gene&term=NA) | NA | **0.588** | **46.635** | 41.646 | **27.403** | 12.629 | 4.809E-02 |
| [231173_at](https://www.affymetrix.com/LinkServlet?probeset=231173_at) | [PYROXD1](http://www.ncbi.nlm.nih.gov/entrez/query.fcgi?cmd=search&db=gene&term=PYROXD1) | pyridine nucleotide-disulphide oxidoreductase domain 1 | **0.588** | **16.381** | 8.954 | **9.638** | 5.819 | 3.418E-02 |
| [215826_x_at](https://www.affymetrix.com/LinkServlet?probeset=215826_x_at) | [ZNF835](http://www.ncbi.nlm.nih.gov/entrez/query.fcgi?cmd=search&db=gene&term=ZNF835) | zinc finger protein 835 | **0.589** | **123.511** | 40.549 | **72.774** | 37.935 | 1.094E-02 |
| [1561502_x_at](https://www.affymetrix.com/LinkServlet?probeset=1561502_x_at) | [CTU2](http://www.ncbi.nlm.nih.gov/entrez/query.fcgi?cmd=search&db=gene&term=CTU2) | cytosolic thiouridylase subunit 2 homolog (S. pombe) | **0.590** | **15.394** | 7.169 | **9.079** | 3.201 | 8.170E-03 |
| [239834_at](https://www.affymetrix.com/LinkServlet?probeset=239834_at) | [NA](http://www.ncbi.nlm.nih.gov/entrez/query.fcgi?cmd=search&db=gene&term=NA) | NA | **0.590** | **11.543** | 6.406 | **6.811** | 1.630 | 2.160E-02 |
| [217693_x_at](https://www.affymetrix.com/LinkServlet?probeset=217693_x_at) | [MAGOH2](http://www.ncbi.nlm.nih.gov/entrez/query.fcgi?cmd=search&db=gene&term=MAGOH2) | mago-nashi homolog 2, proliferation-associated (Drosophila) | **0.591** | **26.725** | 16.474 | **15.792** | 8.929 | 7.146E-03 |
| [225907_at](https://www.affymetrix.com/LinkServlet?probeset=225907_at) | [LOC728743](http://www.ncbi.nlm.nih.gov/entrez/query.fcgi?cmd=search&db=gene&term=LOC728743) | similar to GLI-Kruppel family member HKR1 | **0.591** | **67.142** | 39.782 | **39.675** | 25.055 | 3.406E-02 |
| [1570373_at](https://www.affymetrix.com/LinkServlet?probeset=1570373_at) | [ZNF746](http://www.ncbi.nlm.nih.gov/entrez/query.fcgi?cmd=search&db=gene&term=ZNF746) | zinc finger protein 746 | **0.591** | **13.403** | 7.278 | **7.922** | 3.083 | 2.800E-02 |
| [233076_at](https://www.affymetrix.com/LinkServlet?probeset=233076_at) | [JAKMIP3](http://www.ncbi.nlm.nih.gov/entrez/query.fcgi?cmd=search&db=gene&term=JAKMIP3) | Janus kinase and microtubule interacting protein 3 | **0.592** | **10.912** | 5.434 | **6.457** | 1.384 | 4.923E-03 |
| [232796_at](https://www.affymetrix.com/LinkServlet?probeset=232796_at) | [NA](http://www.ncbi.nlm.nih.gov/entrez/query.fcgi?cmd=search&db=gene&term=NA) | NA | **0.593** | **14.799** | 7.143 | **8.773** | 3.865 | 1.252E-02 |
| [219073_s_at](https://www.affymetrix.com/LinkServlet?probeset=219073_s_at) | [OSBPL10](http://www.ncbi.nlm.nih.gov/entrez/query.fcgi?cmd=search&db=gene&term=OSBPL10) | oxysterol binding protein-like 10 | **0.595** | **144.281** | 115.251 | **85.880** | 101.640 | 2.201E-02 |
| [214959_s_at](https://www.affymetrix.com/LinkServlet?probeset=214959_s_at) | [API5](http://www.ncbi.nlm.nih.gov/entrez/query.fcgi?cmd=search&db=gene&term=API5) | apoptosis inhibitor 5 | **0.596** | **51.956** | 22.574 | **30.950** | 13.496 | 1.373E-02 |
| [1555878_at](https://www.affymetrix.com/LinkServlet?probeset=1555878_at) | [RPS24](http://www.ncbi.nlm.nih.gov/entrez/query.fcgi?cmd=search&db=gene&term=RPS24) | ribosomal protein S24 | **0.597** | **307.992** | 105.052 | **183.756** | 114.355 | 5.406E-03 |
| [219693_at](https://www.affymetrix.com/LinkServlet?probeset=219693_at) | [AGPAT4](http://www.ncbi.nlm.nih.gov/entrez/query.fcgi?cmd=search&db=gene&term=AGPAT4) | 1-acylglycerol-3-phosphate O-acyltransferase 4 (lysophosphatidic acid acyltransferase, delta) | **0.597** | **60.808** | 25.301 | **36.304** | 21.130 | 6.295E-03 |
| [216014_s_at](https://www.affymetrix.com/LinkServlet?probeset=216014_s_at) | [NA](http://www.ncbi.nlm.nih.gov/entrez/query.fcgi?cmd=search&db=gene&term=NA) | NA | **0.598** | **14.818** | 8.848 | **8.858** | 5.526 | 4.222E-02 |
| [1563014_at](https://www.affymetrix.com/LinkServlet?probeset=1563014_at) | [RPS15](http://www.ncbi.nlm.nih.gov/entrez/query.fcgi?cmd=search&db=gene&term=RPS15) | ribosomal protein S15 | **0.599** | **16.555** | 9.891 | **9.910** | 3.732 | 4.369E-02 |
| [1558305_at](https://www.affymetrix.com/LinkServlet?probeset=1558305_at) | [GIGYF2](http://www.ncbi.nlm.nih.gov/entrez/query.fcgi?cmd=search&db=gene&term=GIGYF2) | GRB10 interacting GYF protein 2 | **0.600** | **13.599** | 7.028 | **8.158** | 3.533 | 2.154E-02 |
| [223412_at](https://www.affymetrix.com/LinkServlet?probeset=223412_at) | [KBTBD7](http://www.ncbi.nlm.nih.gov/entrez/query.fcgi?cmd=search&db=gene&term=KBTBD7) | kelch repeat and BTB (POZ) domain containing 7 | **0.601** | **62.906** | 34.368 | **37.785** | 17.350 | 1.518E-02 |
| [1555760_a_at](https://www.affymetrix.com/LinkServlet?probeset=1555760_a_at) | [RBM15](http://www.ncbi.nlm.nih.gov/entrez/query.fcgi?cmd=search&db=gene&term=RBM15) | RNA binding motif protein 15 | **0.601** | **95.880** | 23.099 | **57.650** | 20.319 | 1.055E-04 |
| [237689_at](https://www.affymetrix.com/LinkServlet?probeset=237689_at) | [SARS](http://www.ncbi.nlm.nih.gov/entrez/query.fcgi?cmd=search&db=gene&term=SARS) | seryl-tRNA synthetase | **0.604** | **14.492** | 7.951 | **8.757** | 4.014 | 3.357E-02 |
| [241343_at](https://www.affymetrix.com/LinkServlet?probeset=241343_at) | [RNASEH1](http://www.ncbi.nlm.nih.gov/entrez/query.fcgi?cmd=search&db=gene&term=RNASEH1) | ribonuclease H1 | **0.604** | **30.511** | 14.410 | **18.440** | 10.472 | 3.223E-02 |
| [1554043_a_at](https://www.affymetrix.com/LinkServlet?probeset=1554043_a_at) | [NA](http://www.ncbi.nlm.nih.gov/entrez/query.fcgi?cmd=search&db=gene&term=NA) | NA | **0.605** | **19.651** | 16.329 | **11.884** | 13.213 | 2.993E-02 |
| [202714_s_at](https://www.affymetrix.com/LinkServlet?probeset=202714_s_at) | [KIAA0391](http://www.ncbi.nlm.nih.gov/entrez/query.fcgi?cmd=search&db=gene&term=KIAA0391) | KIAA0391 | **0.605** | **105.193** | 39.030 | **63.651** | 39.262 | 1.258E-02 |
| [212668_at](https://www.affymetrix.com/LinkServlet?probeset=212668_at) | [SMURF1](http://www.ncbi.nlm.nih.gov/entrez/query.fcgi?cmd=search&db=gene&term=SMURF1) | SMAD specific E3 ubiquitin protein ligase 1 | **0.605** | **16.261** | 8.064 | **9.843** | 5.964 | 1.515E-02 |
| [205339_at](https://www.affymetrix.com/LinkServlet?probeset=205339_at) | [STIL](http://www.ncbi.nlm.nih.gov/entrez/query.fcgi?cmd=search&db=gene&term=STIL) | SCL/TAL1 interrupting locus | **0.605** | **112.907** | 75.267 | **68.352** | 59.171 | 1.077E-02 |
| [227503_at](https://www.affymetrix.com/LinkServlet?probeset=227503_at) | [NA](http://www.ncbi.nlm.nih.gov/entrez/query.fcgi?cmd=search&db=gene&term=NA) | NA | **0.606** | **21.200** | 9.832 | **12.838** | 5.677 | 3.098E-02 |
| [222869_s_at](https://www.affymetrix.com/LinkServlet?probeset=222869_s_at) | [ELAC1](http://www.ncbi.nlm.nih.gov/entrez/query.fcgi?cmd=search&db=gene&term=ELAC1) | elaC homolog 1 (E. coli) | **0.606** | **86.448** | 59.888 | **52.361** | 28.229 | 2.697E-02 |
| [225655_at](https://www.affymetrix.com/LinkServlet?probeset=225655_at) | [UHRF1](http://www.ncbi.nlm.nih.gov/entrez/query.fcgi?cmd=search&db=gene&term=UHRF1) | ubiquitin-like with PHD and ring finger domains 1 | **0.606** | **104.415** | 50.839 | **63.265** | 30.280 | 1.430E-02 |
| [227533_at](https://www.affymetrix.com/LinkServlet?probeset=227533_at) | [NA](http://www.ncbi.nlm.nih.gov/entrez/query.fcgi?cmd=search&db=gene&term=NA) | NA | **0.606** | **162.177** | 71.096 | **98.349** | 62.627 | 1.646E-02 |
| [235918_x_at](https://www.affymetrix.com/LinkServlet?probeset=235918_x_at) | [CEP97](http://www.ncbi.nlm.nih.gov/entrez/query.fcgi?cmd=search&db=gene&term=CEP97) | centrosomal protein 97kDa | **0.607** | **229.453** | 58.873 | **139.196** | 51.732 | 1.836E-04 |
| [214787_at](https://www.affymetrix.com/LinkServlet?probeset=214787_at) | [DENND4A](http://www.ncbi.nlm.nih.gov/entrez/query.fcgi?cmd=search&db=gene&term=DENND4A) | DENN/MADD domain containing 4A | **0.607** | **92.940** | 30.730 | **56.432** | 21.545 | 1.927E-03 |
| [240321_at](https://www.affymetrix.com/LinkServlet?probeset=240321_at) | [NA](http://www.ncbi.nlm.nih.gov/entrez/query.fcgi?cmd=search&db=gene&term=NA) | NA | **0.607** | **9.882** | 5.462 | **6.000** | 0.000 | 1.618E-02 |
| [1558943_x_at](https://www.affymetrix.com/LinkServlet?probeset=1558943_x_at) | [ZNF765](http://www.ncbi.nlm.nih.gov/entrez/query.fcgi?cmd=search&db=gene&term=ZNF765) | zinc finger protein 765 | **0.607** | **165.087** | 36.564 | **100.244** | 23.419 | 9.700E-06 |
| [221185_s_at](https://www.affymetrix.com/LinkServlet?probeset=221185_s_at) | [IQCG](http://www.ncbi.nlm.nih.gov/entrez/query.fcgi?cmd=search&db=gene&term=IQCG) | IQ motif containing G | **0.607** | **101.374** | 58.725 | **61.562** | 24.192 | 1.428E-02 |
| [215645_at](https://www.affymetrix.com/LinkServlet?probeset=215645_at) | [FLCN](http://www.ncbi.nlm.nih.gov/entrez/query.fcgi?cmd=search&db=gene&term=FLCN) | folliculin | **0.609** | **19.715** | 11.080 | **12.010** | 7.961 | 2.405E-02 |
| [241933_at](https://www.affymetrix.com/LinkServlet?probeset=241933_at) | [QRSL1](http://www.ncbi.nlm.nih.gov/entrez/query.fcgi?cmd=search&db=gene&term=QRSL1) | glutaminyl-tRNA synthase (glutamine-hydrolyzing)-like 1 | **0.610** | **61.180** | 37.497 | **37.291** | 12.098 | 2.355E-02 |
| [243817_at](https://www.affymetrix.com/LinkServlet?probeset=243817_at) | [NA](http://www.ncbi.nlm.nih.gov/entrez/query.fcgi?cmd=search&db=gene&term=NA) | NA | **0.610** | **35.265** | 14.740 | **21.511** | 9.574 | 7.140E-03 |
| [238607_at](https://www.affymetrix.com/LinkServlet?probeset=238607_at) | [ZNF296](http://www.ncbi.nlm.nih.gov/entrez/query.fcgi?cmd=search&db=gene&term=ZNF296) | zinc finger protein 296 | **0.610** | **36.477** | 19.500 | **22.263** | 8.520 | 1.865E-02 |
| [1552794_a_at](https://www.affymetrix.com/LinkServlet?probeset=1552794_a_at) | [ZNF547](http://www.ncbi.nlm.nih.gov/entrez/query.fcgi?cmd=search&db=gene&term=ZNF547) | zinc finger protein 547 | **0.611** | **52.880** | 19.361 | **32.285** | 17.442 | 4.063E-02 |
| [243508_at](https://www.affymetrix.com/LinkServlet?probeset=243508_at) | [C20orf196](http://www.ncbi.nlm.nih.gov/entrez/query.fcgi?cmd=search&db=gene&term=C20orf196) | chromosome 20 open reading frame 196 | **0.612** | **36.809** | 14.740 | **22.516** | 14.543 | 1.810E-02 |
| [229974_at](https://www.affymetrix.com/LinkServlet?probeset=229974_at) | [EVC2](http://www.ncbi.nlm.nih.gov/entrez/query.fcgi?cmd=search&db=gene&term=EVC2) | Ellis van Creveld syndrome 2 | **0.613** | **10.715** | 7.222 | **6.565** | 1.429 | 2.993E-02 |
| [227331_at](https://www.affymetrix.com/LinkServlet?probeset=227331_at) | [ZNF740](http://www.ncbi.nlm.nih.gov/entrez/query.fcgi?cmd=search&db=gene&term=ZNF740) | zinc finger protein 740 | **0.614** | **52.652** | 29.948 | **32.337** | 14.020 | 9.997E-03 |
| [238504_at](https://www.affymetrix.com/LinkServlet?probeset=238504_at) | [C6orf57](http://www.ncbi.nlm.nih.gov/entrez/query.fcgi?cmd=search&db=gene&term=C6orf57) | chromosome 6 open reading frame 57 | **0.615** | **24.911** | 11.766 | **15.327** | 9.261 | 7.803E-03 |
| [234060_at](https://www.affymetrix.com/LinkServlet?probeset=234060_at) | [NA](http://www.ncbi.nlm.nih.gov/entrez/query.fcgi?cmd=search&db=gene&term=NA) | NA | **0.616** | **15.335** | 9.375 | **9.441** | 3.996 | 3.684E-02 |
| [222820_at](https://www.affymetrix.com/LinkServlet?probeset=222820_at) | [TNRC6C](http://www.ncbi.nlm.nih.gov/entrez/query.fcgi?cmd=search&db=gene&term=TNRC6C) | trinucleotide repeat containing 6C | **0.617** | **149.819** | 84.121 | **92.402** | 47.893 | 3.281E-02 |
| [239937_at](https://www.affymetrix.com/LinkServlet?probeset=239937_at) | [ZNF207](http://www.ncbi.nlm.nih.gov/entrez/query.fcgi?cmd=search&db=gene&term=ZNF207) | zinc finger protein 207 | **0.617** | **89.997** | 27.845 | **55.532** | 25.522 | 2.983E-03 |
| [215629_s_at](https://www.affymetrix.com/LinkServlet?probeset=215629_s_at) | [NA](http://www.ncbi.nlm.nih.gov/entrez/query.fcgi?cmd=search&db=gene&term=NA) | NA | **0.618** | **38.591** | 17.315 | **23.834** | 13.180 | 4.856E-02 |
| [223588_at](https://www.affymetrix.com/LinkServlet?probeset=223588_at) | [THAP2](http://www.ncbi.nlm.nih.gov/entrez/query.fcgi?cmd=search&db=gene&term=THAP2) | THAP domain containing, apoptosis associated protein 2 | **0.619** | **40.604** | 21.909 | **25.114** | 14.983 | 2.983E-02 |
| [216175_at](https://www.affymetrix.com/LinkServlet?probeset=216175_at) | [NA](http://www.ncbi.nlm.nih.gov/entrez/query.fcgi?cmd=search&db=gene&term=NA) | NA | **0.619** | **17.010** | 9.016 | **10.524** | 4.752 | 2.277E-02 |
| [240957_at](https://www.affymetrix.com/LinkServlet?probeset=240957_at) | [NA](http://www.ncbi.nlm.nih.gov/entrez/query.fcgi?cmd=search&db=gene&term=NA) | NA | **0.619** | **21.258** | 13.723 | **13.168** | 8.981 | 3.463E-02 |
| [227340_s_at](https://www.affymetrix.com/LinkServlet?probeset=227340_s_at) | [RGMB](http://www.ncbi.nlm.nih.gov/entrez/query.fcgi?cmd=search&db=gene&term=RGMB) | RGM domain family, member B | **0.620** | **15.058** | 6.922 | **9.334** | 3.127 | 2.538E-02 |
| [223601_at](https://www.affymetrix.com/LinkServlet?probeset=223601_at) | [OLFM2](http://www.ncbi.nlm.nih.gov/entrez/query.fcgi?cmd=search&db=gene&term=OLFM2) | olfactomedin 2 | **0.620** | **22.880** | 11.140 | **14.188** | 7.434 | 3.305E-02 |
| [210540_s_at](https://www.affymetrix.com/LinkServlet?probeset=210540_s_at) | [B4GALT4](http://www.ncbi.nlm.nih.gov/entrez/query.fcgi?cmd=search&db=gene&term=B4GALT4) | UDP-Gal:betaGlcNAc beta 1,4- galactosyltransferase, polypeptide 4 | **0.622** | **94.535** | 40.877 | **58.781** | 29.101 | 1.182E-02 |
| [209374_s_at](https://www.affymetrix.com/LinkServlet?probeset=209374_s_at) | [IGHM](http://www.ncbi.nlm.nih.gov/entrez/query.fcgi?cmd=search&db=gene&term=IGHM) | immunoglobulin heavy constant mu | **0.622** | **955.895** | 805.676 | **594.584** | 962.531 | 1.782E-02 |
| [219715_s_at](https://www.affymetrix.com/LinkServlet?probeset=219715_s_at) | [TDP1](http://www.ncbi.nlm.nih.gov/entrez/query.fcgi?cmd=search&db=gene&term=TDP1) | tyrosyl-DNA phosphodiesterase 1 | **0.622** | **110.900** | 38.844 | **69.009** | 36.670 | 6.014E-03 |
| [215607_x_at](https://www.affymetrix.com/LinkServlet?probeset=215607_x_at) | [NA](http://www.ncbi.nlm.nih.gov/entrez/query.fcgi?cmd=search&db=gene&term=NA) | NA | **0.622** | **143.884** | 47.884 | **89.546** | 38.700 | 8.868E-03 |
| [213449_at](https://www.affymetrix.com/LinkServlet?probeset=213449_at) | [POP1](http://www.ncbi.nlm.nih.gov/entrez/query.fcgi?cmd=search&db=gene&term=POP1) | processing of precursor 1, ribonuclease P/MRP subunit (S. cerevisiae) | **0.624** | **80.291** | 36.841 | **50.123** | 32.372 | 1.876E-02 |
| [202558_s_at](https://www.affymetrix.com/LinkServlet?probeset=202558_s_at) | [HSPA13](http://www.ncbi.nlm.nih.gov/entrez/query.fcgi?cmd=search&db=gene&term=HSPA13) | heat shock protein 70kDa family, member 13 | **0.625** | **49.483** | 37.409 | **30.928** | 19.538 | 3.945E-02 |
| [219716_at](https://www.affymetrix.com/LinkServlet?probeset=219716_at) | [APOL6](http://www.ncbi.nlm.nih.gov/entrez/query.fcgi?cmd=search&db=gene&term=APOL6) | apolipoprotein L, 6 | **0.625** | **122.045** | 39.749 | **76.289** | 33.974 | 3.468E-03 |
| [204024_at](https://www.affymetrix.com/LinkServlet?probeset=204024_at) | [OSGIN2](http://www.ncbi.nlm.nih.gov/entrez/query.fcgi?cmd=search&db=gene&term=OSGIN2) | oxidative stress induced growth inhibitor family member 2 | **0.625** | **42.689** | 25.397 | **26.697** | 15.867 | 1.876E-02 |
| [205558_at](https://www.affymetrix.com/LinkServlet?probeset=205558_at) | [TRAF6](http://www.ncbi.nlm.nih.gov/entrez/query.fcgi?cmd=search&db=gene&term=TRAF6) | TNF receptor-associated factor 6 | **0.626** | **72.360** | 39.310 | **45.281** | 18.828 | 1.543E-02 |
| [240844_at](https://www.affymetrix.com/LinkServlet?probeset=240844_at) | [NA](http://www.ncbi.nlm.nih.gov/entrez/query.fcgi?cmd=search&db=gene&term=NA) | NA | **0.626** | **14.241** | 8.697 | **8.915** | 4.938 | 3.712E-02 |
| [214915_at](https://www.affymetrix.com/LinkServlet?probeset=214915_at) | [ZNF362](http://www.ncbi.nlm.nih.gov/entrez/query.fcgi?cmd=search&db=gene&term=ZNF362) | zinc finger protein 362 | **0.626** | **14.847** | 9.123 | **9.300** | 4.695 | 4.239E-02 |
| [205267_at](https://www.affymetrix.com/LinkServlet?probeset=205267_at) | [POU2AF1](http://www.ncbi.nlm.nih.gov/entrez/query.fcgi?cmd=search&db=gene&term=POU2AF1) | POU class 2 associating factor 1 | **0.627** | **1496.651** | 580.919 | **937.794** | 634.061 | 1.543E-02 |
| [232392_at](https://www.affymetrix.com/LinkServlet?probeset=232392_at) | [SRSF3](http://www.ncbi.nlm.nih.gov/entrez/query.fcgi?cmd=search&db=gene&term=SRSF3) | serine/arginine-rich splicing factor 3 | **0.627** | **341.638** | 204.040 | **214.325** | 97.958 | 4.282E-02 |
| [228027_at](https://www.affymetrix.com/LinkServlet?probeset=228027_at) | [GPRASP2](http://www.ncbi.nlm.nih.gov/entrez/query.fcgi?cmd=search&db=gene&term=GPRASP2) | G protein-coupled receptor associated sorting protein 2 | **0.628** | **63.808** | 24.934 | **40.049** | 27.321 | 8.482E-03 |
| [214006_s_at](https://www.affymetrix.com/LinkServlet?probeset=214006_s_at) | [GGCX](http://www.ncbi.nlm.nih.gov/entrez/query.fcgi?cmd=search&db=gene&term=GGCX) | gamma-glutamyl carboxylase | **0.628** | **77.632** | 25.542 | **48.784** | 18.668 | 1.921E-03 |
| [210385_s_at](https://www.affymetrix.com/LinkServlet?probeset=210385_s_at) | [ERAP1](http://www.ncbi.nlm.nih.gov/entrez/query.fcgi?cmd=search&db=gene&term=ERAP1) | endoplasmic reticulum aminopeptidase 1 | **0.629** | **68.490** | 36.349 | **43.086** | 28.227 | 3.662E-02 |
| [238858_at](https://www.affymetrix.com/LinkServlet?probeset=238858_at) | [TIFA](http://www.ncbi.nlm.nih.gov/entrez/query.fcgi?cmd=search&db=gene&term=TIFA) | TRAF-interacting protein with forkhead-associated domain | **0.630** | **19.057** | 8.374 | **12.010** | 5.596 | 9.121E-03 |
| [221253_s_at](https://www.affymetrix.com/LinkServlet?probeset=221253_s_at) | [NA](http://www.ncbi.nlm.nih.gov/entrez/query.fcgi?cmd=search&db=gene&term=NA) | NA | **0.631** | **1041.940** | 576.657 | **657.263** | 521.613 | 4.064E-02 |
| [215497_s_at](https://www.affymetrix.com/LinkServlet?probeset=215497_s_at) | [WDTC1](http://www.ncbi.nlm.nih.gov/entrez/query.fcgi?cmd=search&db=gene&term=WDTC1) | WD and tetratricopeptide repeats 1 | **0.631** | **64.384** | 47.715 | **40.620** | 15.510 | 3.854E-02 |
| [244011_at](https://www.affymetrix.com/LinkServlet?probeset=244011_at) | [PPM1K](http://www.ncbi.nlm.nih.gov/entrez/query.fcgi?cmd=search&db=gene&term=PPM1K) | protein phosphatase, Mg2+/Mn2+ dependent, 1K | **0.631** | **21.312** | 7.178 | **13.448** | 8.742 | 5.191E-03 |
| [218122_s_at](https://www.affymetrix.com/LinkServlet?probeset=218122_s_at) | [SENP2](http://www.ncbi.nlm.nih.gov/entrez/query.fcgi?cmd=search&db=gene&term=SENP2) | SUMO1/sentrin/SMT3 specific peptidase 2 | **0.631** | **19.596** | 9.368 | **12.367** | 7.437 | 1.322E-02 |
| [200998_s_at](https://www.affymetrix.com/LinkServlet?probeset=200998_s_at) | [CKAP4](http://www.ncbi.nlm.nih.gov/entrez/query.fcgi?cmd=search&db=gene&term=CKAP4) | cytoskeleton-associated protein 4 | **0.631** | **34.230** | 21.586 | **21.614** | 12.184 | 3.566E-02 |
| [1559848_at](https://www.affymetrix.com/LinkServlet?probeset=1559848_at) | [NSUN4](http://www.ncbi.nlm.nih.gov/entrez/query.fcgi?cmd=search&db=gene&term=NSUN4) | NOP2/Sun domain family, member 4 | **0.632** | **10.523** | 4.368 | **6.653** | 1.308 | 5.020E-03 |
| [1569183_a_at](https://www.affymetrix.com/LinkServlet?probeset=1569183_a_at) | [CHM](http://www.ncbi.nlm.nih.gov/entrez/query.fcgi?cmd=search&db=gene&term=CHM) | choroideremia (Rab escort protein 1) | **0.633** | **25.821** | 14.450 | **16.349** | 15.633 | 2.171E-02 |
| [238637_at](https://www.affymetrix.com/LinkServlet?probeset=238637_at) | [NA](http://www.ncbi.nlm.nih.gov/entrez/query.fcgi?cmd=search&db=gene&term=NA) | NA | **0.633** | **25.579** | 8.582 | **16.197** | 9.830 | 5.673E-03 |
| [202085_at](https://www.affymetrix.com/LinkServlet?probeset=202085_at) | [TJP2](http://www.ncbi.nlm.nih.gov/entrez/query.fcgi?cmd=search&db=gene&term=TJP2) | tight junction protein 2 (zona occludens 2) | **0.633** | **75.686** | 37.482 | **47.943** | 28.246 | 3.823E-02 |
| [228583_at](https://www.affymetrix.com/LinkServlet?probeset=228583_at) | [LIN52](http://www.ncbi.nlm.nih.gov/entrez/query.fcgi?cmd=search&db=gene&term=LIN52) | lin-52 homolog (C. elegans) | **0.634** | **66.357** | 28.963 | **42.064** | 19.134 | 1.342E-02 |
| [234462_at](https://www.affymetrix.com/LinkServlet?probeset=234462_at) | [NA](http://www.ncbi.nlm.nih.gov/entrez/query.fcgi?cmd=search&db=gene&term=NA) | NA | **0.634** | **23.167** | 10.909 | **14.688** | 10.137 | 3.830E-02 |
| [1554067_at](https://www.affymetrix.com/LinkServlet?probeset=1554067_at) | [C12orf66](http://www.ncbi.nlm.nih.gov/entrez/query.fcgi?cmd=search&db=gene&term=C12orf66) | chromosome 12 open reading frame 66 | **0.634** | **169.846** | 45.512 | **107.710** | 60.983 | 1.573E-03 |
| [204375_at](https://www.affymetrix.com/LinkServlet?probeset=204375_at) | [CLSTN3](http://www.ncbi.nlm.nih.gov/entrez/query.fcgi?cmd=search&db=gene&term=CLSTN3) | calsyntenin 3 | **0.634** | **33.313** | 17.584 | **21.129** | 7.393 | 3.770E-02 |
| [1554084_a_at](https://www.affymetrix.com/LinkServlet?probeset=1554084_a_at) | [NOL9](http://www.ncbi.nlm.nih.gov/entrez/query.fcgi?cmd=search&db=gene&term=NOL9) | nucleolar protein 9 | **0.635** | **18.066** | 9.581 | **11.465** | 9.472 | 3.277E-02 |
| [222262_s_at](https://www.affymetrix.com/LinkServlet?probeset=222262_s_at) | [ETNK1](http://www.ncbi.nlm.nih.gov/entrez/query.fcgi?cmd=search&db=gene&term=ETNK1) | ethanolamine kinase 1 | **0.635** | **69.378** | 45.162 | **44.035** | 32.280 | 3.474E-02 |
| [212182_at](https://www.affymetrix.com/LinkServlet?probeset=212182_at) | [NA](http://www.ncbi.nlm.nih.gov/entrez/query.fcgi?cmd=search&db=gene&term=NA) | NA | **0.635** | **88.370** | 28.720 | **56.091** | 17.937 | 1.915E-03 |
| [213600_at](https://www.affymetrix.com/LinkServlet?probeset=213600_at) | [SIPA1L3](http://www.ncbi.nlm.nih.gov/entrez/query.fcgi?cmd=search&db=gene&term=SIPA1L3) | signal-induced proliferation-associated 1 like 3 | **0.636** | **246.220** | 104.352 | **156.610** | 93.665 | 1.419E-02 |
| [235675_at](https://www.affymetrix.com/LinkServlet?probeset=235675_at) | [DHFRL1](http://www.ncbi.nlm.nih.gov/entrez/query.fcgi?cmd=search&db=gene&term=DHFRL1) | dihydrofolate reductase-like 1 | **0.636** | **64.049** | 26.467 | **40.761** | 17.473 | 4.217E-02 |
| [209145_s_at](https://www.affymetrix.com/LinkServlet?probeset=209145_s_at) | [CBFA2T2](http://www.ncbi.nlm.nih.gov/entrez/query.fcgi?cmd=search&db=gene&term=CBFA2T2) | core-binding factor, runt domain, alpha subunit 2; translocated to, 2 | **0.637** | **13.657** | 7.551 | **8.698** | 4.459 | 3.423E-02 |
| [237289_at](https://www.affymetrix.com/LinkServlet?probeset=237289_at) | [CREB1](http://www.ncbi.nlm.nih.gov/entrez/query.fcgi?cmd=search&db=gene&term=CREB1) | cAMP responsive element binding protein 1 | **0.639** | **75.682** | 45.202 | **48.340** | 29.413 | 4.041E-02 |
| [205461_at](https://www.affymetrix.com/LinkServlet?probeset=205461_at) | [RAB35](http://www.ncbi.nlm.nih.gov/entrez/query.fcgi?cmd=search&db=gene&term=RAB35) | RAB35, member RAS oncogene family | **0.639** | **11.483** | 6.708 | **7.338** | 1.856 | 3.317E-02 |
| [237107_at](https://www.affymetrix.com/LinkServlet?probeset=237107_at) | [PRKRA](http://www.ncbi.nlm.nih.gov/entrez/query.fcgi?cmd=search&db=gene&term=PRKRA) | protein kinase, interferon-inducible double stranded RNA dependent activator | **0.639** | **37.325** | 13.595 | **23.854** | 18.559 | 8.224E-03 |
| [229382_at](https://www.affymetrix.com/LinkServlet?probeset=229382_at) | [C1orf183](http://www.ncbi.nlm.nih.gov/entrez/query.fcgi?cmd=search&db=gene&term=C1orf183) | chromosome 1 open reading frame 183 | **0.640** | **11.504** | 6.850 | **7.361** | 2.232 | 3.693E-02 |
| [240128_at](https://www.affymetrix.com/LinkServlet?probeset=240128_at) | [NA](http://www.ncbi.nlm.nih.gov/entrez/query.fcgi?cmd=search&db=gene&term=NA) | NA | **0.641** | **184.151** | 92.997 | **118.015** | 69.034 | 1.894E-02 |
| [241808_at](https://www.affymetrix.com/LinkServlet?probeset=241808_at) | [FAM164A](http://www.ncbi.nlm.nih.gov/entrez/query.fcgi?cmd=search&db=gene&term=FAM164A) | family with sequence similarity 164, member A | **0.641** | **86.392** | 29.121 | **55.374** | 22.554 | 7.350E-03 |
| [59705_at](https://www.affymetrix.com/LinkServlet?probeset=59705_at) | [SCLY](http://www.ncbi.nlm.nih.gov/entrez/query.fcgi?cmd=search&db=gene&term=SCLY) | selenocysteine lyase | **0.642** | **14.855** | 5.863 | **9.541** | 4.011 | 1.643E-02 |
| [225616_at](https://www.affymetrix.com/LinkServlet?probeset=225616_at) | [SPRYD4](http://www.ncbi.nlm.nih.gov/entrez/query.fcgi?cmd=search&db=gene&term=SPRYD4) | SPRY domain containing 4 | **0.644** | **25.757** | 10.801 | **16.576** | 8.128 | 3.156E-02 |
| [241621_at](https://www.affymetrix.com/LinkServlet?probeset=241621_at) | [SMCHD1](http://www.ncbi.nlm.nih.gov/entrez/query.fcgi?cmd=search&db=gene&term=SMCHD1) | structural maintenance of chromosomes flexible hinge domain containing 1 | **0.644** | **78.572** | 32.328 | **50.570** | 22.139 | 1.787E-02 |
| [1559134_a_at](https://www.affymetrix.com/LinkServlet?probeset=1559134_a_at) | [NA](http://www.ncbi.nlm.nih.gov/entrez/query.fcgi?cmd=search&db=gene&term=NA) | NA | **0.644** | **10.741** | 5.352 | **6.918** | 1.726 | 1.967E-02 |
| [222642_s_at](https://www.affymetrix.com/LinkServlet?probeset=222642_s_at) | [TMEM33](http://www.ncbi.nlm.nih.gov/entrez/query.fcgi?cmd=search&db=gene&term=TMEM33) | transmembrane protein 33 | **0.644** | **99.284** | 23.756 | **63.947** | 23.977 | 4.195E-03 |
| [235648_at](https://www.affymetrix.com/LinkServlet?probeset=235648_at) | [ZNF567](http://www.ncbi.nlm.nih.gov/entrez/query.fcgi?cmd=search&db=gene&term=ZNF567) | zinc finger protein 567 | **0.644** | **65.281** | 18.737 | **42.051** | 26.206 | 7.380E-03 |
| [1559964_at](https://www.affymetrix.com/LinkServlet?probeset=1559964_at) | [FLJ38717](http://www.ncbi.nlm.nih.gov/entrez/query.fcgi?cmd=search&db=gene&term=FLJ38717) | FLJ38717 protein | **0.644** | **129.510** | 45.927 | **83.452** | 38.090 | 2.271E-02 |
| [201686_x_at](https://www.affymetrix.com/LinkServlet?probeset=201686_x_at) | [API5](http://www.ncbi.nlm.nih.gov/entrez/query.fcgi?cmd=search&db=gene&term=API5) | apoptosis inhibitor 5 | **0.646** | **28.864** | 13.712 | **18.637** | 7.342 | 1.902E-02 |
| [209934_s_at](https://www.affymetrix.com/LinkServlet?probeset=209934_s_at) | [ATP2C1](http://www.ncbi.nlm.nih.gov/entrez/query.fcgi?cmd=search&db=gene&term=ATP2C1) | ATPase, Ca++ transporting, type 2C, member 1 | **0.646** | **174.571** | 81.562 | **112.781** | 26.520 | 3.326E-03 |
| [214118_x_at](https://www.affymetrix.com/LinkServlet?probeset=214118_x_at) | [PCM1](http://www.ncbi.nlm.nih.gov/entrez/query.fcgi?cmd=search&db=gene&term=PCM1) | pericentriolar material 1 | **0.647** | **100.988** | 36.887 | **65.289** | 36.991 | 1.481E-02 |
| [1552632_a_at](https://www.affymetrix.com/LinkServlet?probeset=1552632_a_at) | [ARSG](http://www.ncbi.nlm.nih.gov/entrez/query.fcgi?cmd=search&db=gene&term=ARSG) | arylsulfatase G | **0.647** | **35.313** | 12.608 | **22.862** | 11.134 | 1.455E-02 |
| [204434_at](https://www.affymetrix.com/LinkServlet?probeset=204434_at) | [SPATA2](http://www.ncbi.nlm.nih.gov/entrez/query.fcgi?cmd=search&db=gene&term=SPATA2) | spermatogenesis associated 2 | **0.647** | **47.882** | 27.979 | **31.001** | 25.607 | 4.074E-02 |
| [1557512_at](https://www.affymetrix.com/LinkServlet?probeset=1557512_at) | [NA](http://www.ncbi.nlm.nih.gov/entrez/query.fcgi?cmd=search&db=gene&term=NA) | NA | **0.648** | **25.416** | 15.369 | **16.465** | 13.566 | 4.425E-02 |
| [217542_at](https://www.affymetrix.com/LinkServlet?probeset=217542_at) | [MDM2](http://www.ncbi.nlm.nih.gov/entrez/query.fcgi?cmd=search&db=gene&term=MDM2) | Mdm2 p53 binding protein homolog (mouse) | **0.649** | **84.862** | 33.703 | **55.069** | 30.503 | 3.910E-02 |
| [1559893_at](https://www.affymetrix.com/LinkServlet?probeset=1559893_at) | [CCDC75](http://www.ncbi.nlm.nih.gov/entrez/query.fcgi?cmd=search&db=gene&term=CCDC75) | coiled-coil domain containing 75 | **0.650** | **28.947** | 15.043 | **18.802** | 10.723 | 3.777E-02 |
| [230736_at](https://www.affymetrix.com/LinkServlet?probeset=230736_at) | [LOC387647](http://www.ncbi.nlm.nih.gov/entrez/query.fcgi?cmd=search&db=gene&term=LOC387647) | patched domain containing 3 pseudogene | **0.650** | **47.624** | 20.532 | **30.961** | 19.737 | 2.427E-02 |
| [238933_at](https://www.affymetrix.com/LinkServlet?probeset=238933_at) | [NA](http://www.ncbi.nlm.nih.gov/entrez/query.fcgi?cmd=search&db=gene&term=NA) | NA | **0.650** | **9.981** | 5.413 | **6.490** | 0.886 | 3.309E-02 |
| [1554068_s_at](https://www.affymetrix.com/LinkServlet?probeset=1554068_s_at) | [C12orf66](http://www.ncbi.nlm.nih.gov/entrez/query.fcgi?cmd=search&db=gene&term=C12orf66) | chromosome 12 open reading frame 66 | **0.651** | **54.333** | 28.513 | **35.363** | 16.335 | 3.323E-02 |
| [221777_at](https://www.affymetrix.com/LinkServlet?probeset=221777_at) | [C12orf52](http://www.ncbi.nlm.nih.gov/entrez/query.fcgi?cmd=search&db=gene&term=C12orf52) | chromosome 12 open reading frame 52 | **0.652** | **44.815** | 20.444 | **29.217** | 12.331 | 2.859E-02 |
| [226520_at](https://www.affymetrix.com/LinkServlet?probeset=226520_at) | [LCOR](http://www.ncbi.nlm.nih.gov/entrez/query.fcgi?cmd=search&db=gene&term=LCOR) | ligand dependent nuclear receptor corepressor | **0.652** | **225.955** | 83.742 | **147.322** | 70.813 | 7.341E-03 |
| [219123_at](https://www.affymetrix.com/LinkServlet?probeset=219123_at) | [ZNF232](http://www.ncbi.nlm.nih.gov/entrez/query.fcgi?cmd=search&db=gene&term=ZNF232) | zinc finger protein 232 | **0.653** | **175.636** | 84.332 | **114.672** | 32.887 | 1.402E-02 |
| [216072_at](https://www.affymetrix.com/LinkServlet?probeset=216072_at) | [NA](http://www.ncbi.nlm.nih.gov/entrez/query.fcgi?cmd=search&db=gene&term=NA) | NA | **0.654** | **137.008** | 69.849 | **89.584** | 53.608 | 4.454E-02 |
| [229970_at](https://www.affymetrix.com/LinkServlet?probeset=229970_at) | [NA](http://www.ncbi.nlm.nih.gov/entrez/query.fcgi?cmd=search&db=gene&term=NA) | NA | **0.654** | **66.268** | 27.805 | **43.352** | 22.884 | 3.357E-02 |
| [1554894_a_at](https://www.affymetrix.com/LinkServlet?probeset=1554894_a_at) | [PCBD2](http://www.ncbi.nlm.nih.gov/entrez/query.fcgi?cmd=search&db=gene&term=PCBD2) | pterin-4 alpha-carbinolamine dehydratase/dimerization cofactor of hepatocyte nuclear factor 1 alpha (TCF1) 2 | **0.655** | **26.983** | 11.300 | **17.685** | 12.365 | 2.574E-02 |
| [221248_s_at](https://www.affymetrix.com/LinkServlet?probeset=221248_s_at) | [WHSC1L1](http://www.ncbi.nlm.nih.gov/entrez/query.fcgi?cmd=search&db=gene&term=WHSC1L1) | Wolf-Hirschhorn syndrome candidate 1-like 1 | **0.657** | **65.404** | 27.363 | **42.959** | 24.478 | 2.387E-02 |
| [238155_at](https://www.affymetrix.com/LinkServlet?probeset=238155_at) | [NA](http://www.ncbi.nlm.nih.gov/entrez/query.fcgi?cmd=search&db=gene&term=NA) | NA | **0.658** | **55.752** | 20.086 | **36.658** | 15.850 | 1.109E-02 |
| [221002_s_at](https://www.affymetrix.com/LinkServlet?probeset=221002_s_at) | [TSPAN14](http://www.ncbi.nlm.nih.gov/entrez/query.fcgi?cmd=search&db=gene&term=TSPAN14) | tetraspanin 14 | **0.658** | **292.124** | 52.356 | **192.357** | 87.193 | 1.361E-03 |
| [1563781_at](https://www.affymetrix.com/LinkServlet?probeset=1563781_at) | [LOC285949](http://www.ncbi.nlm.nih.gov/entrez/query.fcgi?cmd=search&db=gene&term=LOC285949) | hypothetical protein LOC285949 | **0.659** | **32.754** | 16.370 | **21.581** | 13.767 | 2.591E-02 |
| [243973_at](https://www.affymetrix.com/LinkServlet?probeset=243973_at) | [NA](http://www.ncbi.nlm.nih.gov/entrez/query.fcgi?cmd=search&db=gene&term=NA) | NA | **0.659** | **125.454** | 55.476 | **82.733** | 55.502 | 3.737E-02 |
| [244105_at](https://www.affymetrix.com/LinkServlet?probeset=244105_at) | [NA](http://www.ncbi.nlm.nih.gov/entrez/query.fcgi?cmd=search&db=gene&term=NA) | NA | **0.660** | **49.841** | 19.891 | **32.876** | 27.301 | 2.322E-02 |
| [203069_at](https://www.affymetrix.com/LinkServlet?probeset=203069_at) | [SV2A](http://www.ncbi.nlm.nih.gov/entrez/query.fcgi?cmd=search&db=gene&term=SV2A) | synaptic vesicle glycoprotein 2A | **0.660** | **16.903** | 7.091 | **11.160** | 4.938 | 2.291E-02 |
| [215114_at](https://www.affymetrix.com/LinkServlet?probeset=215114_at) | [SENP3](http://www.ncbi.nlm.nih.gov/entrez/query.fcgi?cmd=search&db=gene&term=SENP3) | SUMO1/sentrin/SMT3 specific peptidase 3 | **0.660** | **22.978** | 10.925 | **15.176** | 9.577 | 3.433E-02 |
| [222805_at](https://www.affymetrix.com/LinkServlet?probeset=222805_at) | [MANEA](http://www.ncbi.nlm.nih.gov/entrez/query.fcgi?cmd=search&db=gene&term=MANEA) | mannosidase, endo-alpha | **0.661** | **149.459** | 40.815 | **98.769** | 41.452 | 1.846E-03 |
| [1553603_s_at](https://www.affymetrix.com/LinkServlet?probeset=1553603_s_at) | [ATL2](http://www.ncbi.nlm.nih.gov/entrez/query.fcgi?cmd=search&db=gene&term=ATL2) | atlastin GTPase 2 | **0.661** | **38.223** | 17.524 | **25.277** | 13.438 | 2.269E-02 |
| [205136_s_at](https://www.affymetrix.com/LinkServlet?probeset=205136_s_at) | [NUFIP1](http://www.ncbi.nlm.nih.gov/entrez/query.fcgi?cmd=search&db=gene&term=NUFIP1) | nuclear fragile X mental retardation protein interacting protein 1 | **0.661** | **17.543** | 8.516 | **11.603** | 3.171 | 1.523E-02 |
| [239771_at](https://www.affymetrix.com/LinkServlet?probeset=239771_at) | [CAND1](http://www.ncbi.nlm.nih.gov/entrez/query.fcgi?cmd=search&db=gene&term=CAND1) | cullin-associated and neddylation-dissociated 1 | **0.662** | **48.592** | 21.081 | **32.154** | 17.290 | 2.190E-02 |
| [1556154_a_at](https://www.affymetrix.com/LinkServlet?probeset=1556154_a_at) | [MGC23284](http://www.ncbi.nlm.nih.gov/entrez/query.fcgi?cmd=search&db=gene&term=MGC23284) | hypothetical LOC197187 | **0.662** | **213.464** | 94.702 | **141.329** | 68.730 | 4.250E-02 |
| [235295_at](https://www.affymetrix.com/LinkServlet?probeset=235295_at) | [NA](http://www.ncbi.nlm.nih.gov/entrez/query.fcgi?cmd=search&db=gene&term=NA) | NA | **0.663** | **52.372** | 19.914 | **34.708** | 18.114 | 4.370E-02 |
| [232463_at](https://www.affymetrix.com/LinkServlet?probeset=232463_at) | [NCRNA00107](http://www.ncbi.nlm.nih.gov/entrez/query.fcgi?cmd=search&db=gene&term=NCRNA00107) | non-protein coding RNA 107 | **0.663** | **67.358** | 21.883 | **44.652** | 21.876 | 2.885E-02 |
| [238425_at](https://www.affymetrix.com/LinkServlet?probeset=238425_at) | [NA](http://www.ncbi.nlm.nih.gov/entrez/query.fcgi?cmd=search&db=gene&term=NA) | NA | **0.663** | **40.359** | 18.005 | **26.767** | 10.694 | 4.809E-02 |
| [223352_s_at](https://www.affymetrix.com/LinkServlet?probeset=223352_s_at) | [C17orf80](http://www.ncbi.nlm.nih.gov/entrez/query.fcgi?cmd=search&db=gene&term=C17orf80) | chromosome 17 open reading frame 80 | **0.663** | **11.908** | 7.772 | **7.899** | 3.265 | 3.951E-02 |
| [218630_at](https://www.affymetrix.com/LinkServlet?probeset=218630_at) | [MKS1](http://www.ncbi.nlm.nih.gov/entrez/query.fcgi?cmd=search&db=gene&term=MKS1) | Meckel syndrome, type 1 | **0.664** | **28.501** | 13.839 | **18.924** | 11.076 | 2.246E-02 |
| [214194_at](https://www.affymetrix.com/LinkServlet?probeset=214194_at) | [NA](http://www.ncbi.nlm.nih.gov/entrez/query.fcgi?cmd=search&db=gene&term=NA) | NA | **0.664** | **191.062** | 57.268 | **126.905** | 58.869 | 1.073E-02 |
| [213560_at](https://www.affymetrix.com/LinkServlet?probeset=213560_at) | [GADD45B](http://www.ncbi.nlm.nih.gov/entrez/query.fcgi?cmd=search&db=gene&term=GADD45B) | growth arrest and DNA-damage-inducible, beta | **0.664** | **359.882** | 148.640 | **239.113** | 64.416 | 5.880E-03 |
| [221913_at](https://www.affymetrix.com/LinkServlet?probeset=221913_at) | [NA](http://www.ncbi.nlm.nih.gov/entrez/query.fcgi?cmd=search&db=gene&term=NA) | NA | **0.667** | **74.263** | 40.437 | **49.531** | 18.685 | 3.850E-02 |
| [243642_x_at](https://www.affymetrix.com/LinkServlet?probeset=243642_x_at) | [NA](http://www.ncbi.nlm.nih.gov/entrez/query.fcgi?cmd=search&db=gene&term=NA) | NA | **0.667** | **248.881** | 105.220 | **166.052** | 115.583 | 4.781E-02 |
| [231328_s_at](https://www.affymetrix.com/LinkServlet?probeset=231328_s_at) | [NA](http://www.ncbi.nlm.nih.gov/entrez/query.fcgi?cmd=search&db=gene&term=NA) | NA | **0.668** | **34.653** | 17.428 | **23.135** | 13.456 | 3.120E-02 |
| [212533_at](https://www.affymetrix.com/LinkServlet?probeset=212533_at) | [WEE1](http://www.ncbi.nlm.nih.gov/entrez/query.fcgi?cmd=search&db=gene&term=WEE1) | WEE1 homolog (S. pombe) | **0.668** | **391.386** | 151.168 | **261.422** | 107.264 | 8.207E-03 |
| [213199_at](https://www.affymetrix.com/LinkServlet?probeset=213199_at) | [C2CD3](http://www.ncbi.nlm.nih.gov/entrez/query.fcgi?cmd=search&db=gene&term=C2CD3) | C2 calcium-dependent domain containing 3 | **0.669** | **119.820** | 68.289 | **80.218** | 30.487 | 1.613E-02 |
| [44065_at](https://www.affymetrix.com/LinkServlet?probeset=44065_at) | [C12orf52](http://www.ncbi.nlm.nih.gov/entrez/query.fcgi?cmd=search&db=gene&term=C12orf52) | chromosome 12 open reading frame 52 | **0.670** | **62.098** | 35.395 | **41.603** | 22.393 | 4.872E-02 |
| [221974_at](https://www.affymetrix.com/LinkServlet?probeset=221974_at) | [IPW](http://www.ncbi.nlm.nih.gov/entrez/query.fcgi?cmd=search&db=gene&term=IPW) | imprinted in Prader-Willi syndrome (non-protein coding) | **0.670** | **82.179** | 33.513 | **55.081** | 46.046 | 1.163E-02 |
| [214291_at](https://www.affymetrix.com/LinkServlet?probeset=214291_at) | [RPL17](http://www.ncbi.nlm.nih.gov/entrez/query.fcgi?cmd=search&db=gene&term=RPL17) | ribosomal protein L17 | **0.670** | **180.874** | 36.385 | **121.259** | 72.537 | 4.941E-03 |
| [224623_at](https://www.affymetrix.com/LinkServlet?probeset=224623_at) | [NA](http://www.ncbi.nlm.nih.gov/entrez/query.fcgi?cmd=search&db=gene&term=NA) | NA | **0.671** | **353.057** | 118.098 | **236.790** | 71.666 | 3.707E-03 |
| [221540_x_at](https://www.affymetrix.com/LinkServlet?probeset=221540_x_at) | [NA](http://www.ncbi.nlm.nih.gov/entrez/query.fcgi?cmd=search&db=gene&term=NA) | NA | **0.671** | **234.895** | 98.954 | **157.692** | 77.302 | 1.164E-02 |
| [218170_at](https://www.affymetrix.com/LinkServlet?probeset=218170_at) | [ISOC1](http://www.ncbi.nlm.nih.gov/entrez/query.fcgi?cmd=search&db=gene&term=ISOC1) | isochorismatase domain containing 1 | **0.671** | **115.755** | 37.721 | **77.720** | 30.807 | 6.848E-03 |
| [228981_at](https://www.affymetrix.com/LinkServlet?probeset=228981_at) | [TMEM169](http://www.ncbi.nlm.nih.gov/entrez/query.fcgi?cmd=search&db=gene&term=TMEM169) | transmembrane protein 169 | **0.673** | **55.881** | 23.968 | **37.592** | 24.062 | 3.959E-02 |
| [223556_at](https://www.affymetrix.com/LinkServlet?probeset=223556_at) | [HELLS](http://www.ncbi.nlm.nih.gov/entrez/query.fcgi?cmd=search&db=gene&term=HELLS) | helicase, lymphoid-specific | **0.673** | **40.287** | 27.370 | **27.104** | 31.237 | 4.294E-02 |
| [203352_at](https://www.affymetrix.com/LinkServlet?probeset=203352_at) | [ORC4](http://www.ncbi.nlm.nih.gov/entrez/query.fcgi?cmd=search&db=gene&term=ORC4) | origin recognition complex, subunit 4 | **0.673** | **76.180** | 30.043 | **51.297** | 22.807 | 9.187E-03 |
| [1562236_at](https://www.affymetrix.com/LinkServlet?probeset=1562236_at) | [MYST4](http://www.ncbi.nlm.nih.gov/entrez/query.fcgi?cmd=search&db=gene&term=MYST4) | MYST histone acetyltransferase (monocytic leukemia) 4 | **0.674** | **46.393** | 21.090 | **31.283** | 19.654 | 4.717E-02 |
| [204918_s_at](https://www.affymetrix.com/LinkServlet?probeset=204918_s_at) | [MLLT3](http://www.ncbi.nlm.nih.gov/entrez/query.fcgi?cmd=search&db=gene&term=MLLT3) | myeloid/lymphoid or mixed-lineage leukemia (trithorax homolog, Drosophila); translocated to, 3 | **0.675** | **113.968** | 42.828 | **76.941** | 39.292 | 2.277E-02 |
| [213949_s_at](https://www.affymetrix.com/LinkServlet?probeset=213949_s_at) | [DOHH](http://www.ncbi.nlm.nih.gov/entrez/query.fcgi?cmd=search&db=gene&term=DOHH) | deoxyhypusine hydroxylase/monooxygenase | **0.675** | **12.446** | 5.234 | **8.405** | 2.970 | 4.609E-02 |
| [228597_at](https://www.affymetrix.com/LinkServlet?probeset=228597_at) | [MIS18A](http://www.ncbi.nlm.nih.gov/entrez/query.fcgi?cmd=search&db=gene&term=MIS18A) | MIS18 kinetochore protein homolog A (S. pombe) | **0.676** | **210.448** | 70.709 | **142.321** | 58.821 | 3.874E-02 |
| [212966_at](https://www.affymetrix.com/LinkServlet?probeset=212966_at) | [HIC2](http://www.ncbi.nlm.nih.gov/entrez/query.fcgi?cmd=search&db=gene&term=HIC2) | hypermethylated in cancer 2 | **0.677** | **25.493** | 10.457 | **17.247** | 7.571 | 3.381E-02 |
| [231818_x_at](https://www.affymetrix.com/LinkServlet?probeset=231818_x_at) | [NA](http://www.ncbi.nlm.nih.gov/entrez/query.fcgi?cmd=search&db=gene&term=NA) | NA | **0.677** | **36.157** | 15.753 | **24.464** | 13.500 | 3.814E-02 |
| [241445_at](https://www.affymetrix.com/LinkServlet?probeset=241445_at) | [NA](http://www.ncbi.nlm.nih.gov/entrez/query.fcgi?cmd=search&db=gene&term=NA) | NA | **0.677** | **20.609** | 9.352 | **13.945** | 10.010 | 4.068E-02 |
| [240516_at](https://www.affymetrix.com/LinkServlet?probeset=240516_at) | [NA](http://www.ncbi.nlm.nih.gov/entrez/query.fcgi?cmd=search&db=gene&term=NA) | NA | **0.677** | **8.995** | 5.201 | **6.088** | 0.198 | 4.900E-02 |
| [203898_at](https://www.affymetrix.com/LinkServlet?probeset=203898_at) | [CRCP](http://www.ncbi.nlm.nih.gov/entrez/query.fcgi?cmd=search&db=gene&term=CRCP) | CGRP receptor component | **0.677** | **59.805** | 19.990 | **40.486** | 12.345 | 6.588E-03 |
| [45633_at](https://www.affymetrix.com/LinkServlet?probeset=45633_at) | [GINS3](http://www.ncbi.nlm.nih.gov/entrez/query.fcgi?cmd=search&db=gene&term=GINS3) | GINS complex subunit 3 (Psf3 homolog) | **0.678** | **146.378** | 49.797 | **99.198** | 36.538 | 1.794E-02 |
| [209304_x_at](https://www.affymetrix.com/LinkServlet?probeset=209304_x_at) | [GADD45B](http://www.ncbi.nlm.nih.gov/entrez/query.fcgi?cmd=search&db=gene&term=GADD45B) | growth arrest and DNA-damage-inducible, beta | **0.678** | **124.671** | 58.794 | **84.511** | 26.981 | 3.618E-02 |
| [208144_s_at](https://www.affymetrix.com/LinkServlet?probeset=208144_s_at) | [NA](http://www.ncbi.nlm.nih.gov/entrez/query.fcgi?cmd=search&db=gene&term=NA) | NA | **0.678** | **22.674** | 10.306 | **15.375** | 6.663 | 4.381E-02 |
| [53968_at](https://www.affymetrix.com/LinkServlet?probeset=53968_at) | [INTS5](http://www.ncbi.nlm.nih.gov/entrez/query.fcgi?cmd=search&db=gene&term=INTS5) | integrator complex subunit 5 | **0.679** | **138.682** | 70.268 | **94.211** | 36.005 | 4.206E-02 |
| [205822_s_at](https://www.affymetrix.com/LinkServlet?probeset=205822_s_at) | [HMGCS1](http://www.ncbi.nlm.nih.gov/entrez/query.fcgi?cmd=search&db=gene&term=HMGCS1) | 3-hydroxy-3-methylglutaryl-CoA synthase 1 (soluble) | **0.680** | **60.080** | 20.510 | **40.833** | 20.168 | 1.752E-02 |
| [240025_x_at](https://www.affymetrix.com/LinkServlet?probeset=240025_x_at) | [NA](http://www.ncbi.nlm.nih.gov/entrez/query.fcgi?cmd=search&db=gene&term=NA) | NA | **0.680** | **134.724** | 49.925 | **91.574** | 53.884 | 4.157E-02 |
| [233650_at](https://www.affymetrix.com/LinkServlet?probeset=233650_at) | [CEP63](http://www.ncbi.nlm.nih.gov/entrez/query.fcgi?cmd=search&db=gene&term=CEP63) | centrosomal protein 63kDa | **0.680** | **24.669** | 10.004 | **16.777** | 7.106 | 3.021E-02 |
| [36552_at](https://www.affymetrix.com/LinkServlet?probeset=36552_at) | [C2CD3](http://www.ncbi.nlm.nih.gov/entrez/query.fcgi?cmd=search&db=gene&term=C2CD3) | C2 calcium-dependent domain containing 3 | **0.681** | **282.251** | 139.907 | **192.075** | 80.482 | 2.093E-02 |
| [235687_at](https://www.affymetrix.com/LinkServlet?probeset=235687_at) | [ZNF626](http://www.ncbi.nlm.nih.gov/entrez/query.fcgi?cmd=search&db=gene&term=ZNF626) | zinc finger protein 626 | **0.681** | **55.198** | 24.959 | **37.594** | 23.156 | 3.257E-02 |
| [221981_s_at](https://www.affymetrix.com/LinkServlet?probeset=221981_s_at) | [WDR59](http://www.ncbi.nlm.nih.gov/entrez/query.fcgi?cmd=search&db=gene&term=WDR59) | WD repeat domain 59 | **0.681** | **24.905** | 9.669 | **16.967** | 8.835 | 3.954E-02 |
| [236466_at](https://www.affymetrix.com/LinkServlet?probeset=236466_at) | [NA](http://www.ncbi.nlm.nih.gov/entrez/query.fcgi?cmd=search&db=gene&term=NA) | NA | **0.682** | **9.021** | 4.967 | **6.150** | 0.498 | 4.830E-02 |
| [1559524_at](https://www.affymetrix.com/LinkServlet?probeset=1559524_at) | [NA](http://www.ncbi.nlm.nih.gov/entrez/query.fcgi?cmd=search&db=gene&term=NA) | NA | **0.682** | **44.022** | 13.200 | **30.015** | 16.565 | 1.582E-02 |
| [212035_s_at](https://www.affymetrix.com/LinkServlet?probeset=212035_s_at) | [EXOC7](http://www.ncbi.nlm.nih.gov/entrez/query.fcgi?cmd=search&db=gene&term=EXOC7) | exocyst complex component 7 | **0.682** | **29.718** | 14.301 | **20.267** | 15.773 | 2.695E-02 |
| [209441_at](https://www.affymetrix.com/LinkServlet?probeset=209441_at) | [RHOBTB2](http://www.ncbi.nlm.nih.gov/entrez/query.fcgi?cmd=search&db=gene&term=RHOBTB2) | Rho-related BTB domain containing 2 | **0.683** | **17.393** | 6.106 | **11.872** | 3.776 | 1.245E-02 |
| [215501_s_at](https://www.affymetrix.com/LinkServlet?probeset=215501_s_at) | [DUSP10](http://www.ncbi.nlm.nih.gov/entrez/query.fcgi?cmd=search&db=gene&term=DUSP10) | dual specificity phosphatase 10 | **0.683** | **30.929** | 15.486 | **21.126** | 15.665 | 4.765E-02 |
| [1564207_at](https://www.affymetrix.com/LinkServlet?probeset=1564207_at) | [FLJ35390](http://www.ncbi.nlm.nih.gov/entrez/query.fcgi?cmd=search&db=gene&term=FLJ35390) | hypothetical LOC255031 | **0.683** | **161.535** | 58.390 | **110.342** | 51.610 | 2.136E-02 |
| [230139_at](https://www.affymetrix.com/LinkServlet?probeset=230139_at) | [NA](http://www.ncbi.nlm.nih.gov/entrez/query.fcgi?cmd=search&db=gene&term=NA) | NA | **0.684** | **151.477** | 49.379 | **103.605** | 46.911 | 3.244E-02 |
| [230065_at](https://www.affymetrix.com/LinkServlet?probeset=230065_at) | [NA](http://www.ncbi.nlm.nih.gov/entrez/query.fcgi?cmd=search&db=gene&term=NA) | NA | **0.684** | **39.910** | 14.691 | **27.312** | 14.009 | 2.293E-02 |
| [242140_at](https://www.affymetrix.com/LinkServlet?probeset=242140_at) | [NA](http://www.ncbi.nlm.nih.gov/entrez/query.fcgi?cmd=search&db=gene&term=NA) | NA | **0.685** | **133.973** | 52.112 | **91.764** | 51.388 | 1.872E-02 |
| [227569_at](https://www.affymetrix.com/LinkServlet?probeset=227569_at) | [LNX2](http://www.ncbi.nlm.nih.gov/entrez/query.fcgi?cmd=search&db=gene&term=LNX2) | ligand of numb-protein X 2 | **0.685** | **182.181** | 47.014 | **124.799** | 52.426 | 2.094E-02 |
| [220235_s_at](https://www.affymetrix.com/LinkServlet?probeset=220235_s_at) | [C1orf103](http://www.ncbi.nlm.nih.gov/entrez/query.fcgi?cmd=search&db=gene&term=C1orf103) | chromosome 1 open reading frame 103 | **0.686** | **96.845** | 29.908 | **66.389** | 15.879 | 4.694E-02 |
| [205013_s_at](https://www.affymetrix.com/LinkServlet?probeset=205013_s_at) | [NA](http://www.ncbi.nlm.nih.gov/entrez/query.fcgi?cmd=search&db=gene&term=NA) | NA | **0.686** | **565.185** | 151.682 | **387.853** | 114.033 | 4.225E-03 |
| [1568964_x_at](https://www.affymetrix.com/LinkServlet?probeset=1568964_x_at) | [SPN](http://www.ncbi.nlm.nih.gov/entrez/query.fcgi?cmd=search&db=gene&term=SPN) | sialophorin | **0.686** | **145.166** | 45.036 | **99.642** | 45.116 | 1.153E-02 |
| [226041_at](https://www.affymetrix.com/LinkServlet?probeset=226041_at) | [NAPEPLD](http://www.ncbi.nlm.nih.gov/entrez/query.fcgi?cmd=search&db=gene&term=NAPEPLD) | N-acyl phosphatidylethanolamine phospholipase D | **0.687** | **72.623** | 34.156 | **49.911** | 24.216 | 4.371E-02 |
| [239086_at](https://www.affymetrix.com/LinkServlet?probeset=239086_at) | [NA](http://www.ncbi.nlm.nih.gov/entrez/query.fcgi?cmd=search&db=gene&term=NA) | NA | **0.690** | **175.019** | 51.653 | **120.703** | 61.110 | 2.154E-02 |
| [203006_at](https://www.affymetrix.com/LinkServlet?probeset=203006_at) | [INPP5A](http://www.ncbi.nlm.nih.gov/entrez/query.fcgi?cmd=search&db=gene&term=INPP5A) | inositol polyphosphate-5-phosphatase, 40kDa | **0.690** | **111.623** | 55.411 | **77.000** | 31.615 | 1.899E-02 |
| [230707_at](https://www.affymetrix.com/LinkServlet?probeset=230707_at) | [SORL1](http://www.ncbi.nlm.nih.gov/entrez/query.fcgi?cmd=search&db=gene&term=SORL1) | sortilin-related receptor, L(DLR class) A repeats containing | **0.690** | **162.496** | 78.069 | **112.130** | 42.226 | 4.681E-02 |
| [233540_s_at](https://www.affymetrix.com/LinkServlet?probeset=233540_s_at) | [CDK5RAP2](http://www.ncbi.nlm.nih.gov/entrez/query.fcgi?cmd=search&db=gene&term=CDK5RAP2) | CDK5 regulatory subunit associated protein 2 | **0.690** | **419.897** | 176.933 | **289.791** | 130.552 | 3.370E-02 |
| [238618_at](https://www.affymetrix.com/LinkServlet?probeset=238618_at) | [NF2](http://www.ncbi.nlm.nih.gov/entrez/query.fcgi?cmd=search&db=gene&term=NF2) | neurofibromin 2 (merlin) | **0.691** | **9.367** | 4.733 | **6.469** | 1.243 | 3.125E-02 |
| [218445_at](https://www.affymetrix.com/LinkServlet?probeset=218445_at) | [H2AFY2](http://www.ncbi.nlm.nih.gov/entrez/query.fcgi?cmd=search&db=gene&term=H2AFY2) | H2A histone family, member Y2 | **0.691** | **106.496** | 44.858 | **73.581** | 23.900 | 2.725E-02 |
| [217826_s_at](https://www.affymetrix.com/LinkServlet?probeset=217826_s_at) | [UBE2J1](http://www.ncbi.nlm.nih.gov/entrez/query.fcgi?cmd=search&db=gene&term=UBE2J1) | ubiquitin-conjugating enzyme E2, J1 (UBC6 homolog, yeast) | **0.691** | **257.121** | 115.238 | **177.660** | 121.586 | 2.306E-02 |
| [228353_x_at](https://www.affymetrix.com/LinkServlet?probeset=228353_x_at) | [UBASH3B](http://www.ncbi.nlm.nih.gov/entrez/query.fcgi?cmd=search&db=gene&term=UBASH3B) | ubiquitin associated and SH3 domain containing B | **0.692** | **133.008** | 28.815 | **92.008** | 21.242 | 8.542E-04 |
| [236128_at](https://www.affymetrix.com/LinkServlet?probeset=236128_at) | [ZNF91](http://www.ncbi.nlm.nih.gov/entrez/query.fcgi?cmd=search&db=gene&term=ZNF91) | zinc finger protein 91 | **0.692** | **260.721** | 65.542 | **180.411** | 91.244 | 5.247E-03 |
| [227822_at](https://www.affymetrix.com/LinkServlet?probeset=227822_at) | [ZNF605](http://www.ncbi.nlm.nih.gov/entrez/query.fcgi?cmd=search&db=gene&term=ZNF605) | zinc finger protein 605 | **0.692** | **132.359** | 40.375 | **91.636** | 50.524 | 1.626E-02 |
| [219291_at](https://www.affymetrix.com/LinkServlet?probeset=219291_at) | [DTWD1](http://www.ncbi.nlm.nih.gov/entrez/query.fcgi?cmd=search&db=gene&term=DTWD1) | DTW domain containing 1 | **0.693** | **81.996** | 25.748 | **56.807** | 43.026 | 1.309E-02 |
| [232071_at](https://www.affymetrix.com/LinkServlet?probeset=232071_at) | [NA](http://www.ncbi.nlm.nih.gov/entrez/query.fcgi?cmd=search&db=gene&term=NA) | NA | **0.693** | **43.558** | 16.133 | **30.185** | 20.244 | 1.782E-02 |
| [241433_at](https://www.affymetrix.com/LinkServlet?probeset=241433_at) | [RCOR3](http://www.ncbi.nlm.nih.gov/entrez/query.fcgi?cmd=search&db=gene&term=RCOR3) | REST corepressor 3 | **0.693** | **37.186** | 18.486 | **25.774** | 16.564 | 4.465E-02 |
| [225970_at](https://www.affymetrix.com/LinkServlet?probeset=225970_at) | [DDHD1](http://www.ncbi.nlm.nih.gov/entrez/query.fcgi?cmd=search&db=gene&term=DDHD1) | DDHD domain containing 1 | **0.694** | **243.851** | 58.979 | **169.165** | 41.816 | 5.994E-04 |
| [221985_at](https://www.affymetrix.com/LinkServlet?probeset=221985_at) | [KLHL24](http://www.ncbi.nlm.nih.gov/entrez/query.fcgi?cmd=search&db=gene&term=KLHL24) | kelch-like 24 (Drosophila) | **0.694** | **75.391** | 27.297 | **52.314** | 19.117 | 2.728E-02 |
| [232058_at](https://www.affymetrix.com/LinkServlet?probeset=232058_at) | [NA](http://www.ncbi.nlm.nih.gov/entrez/query.fcgi?cmd=search&db=gene&term=NA) | NA | **0.694** | **108.743** | 42.900 | **75.465** | 45.740 | 2.902E-02 |
| [207574_s_at](https://www.affymetrix.com/LinkServlet?probeset=207574_s_at) | [GADD45B](http://www.ncbi.nlm.nih.gov/entrez/query.fcgi?cmd=search&db=gene&term=GADD45B) | growth arrest and DNA-damage-inducible, beta | **0.694** | **320.140** | 149.343 | **222.230** | 62.085 | 3.902E-02 |
| [226391_at](https://www.affymetrix.com/LinkServlet?probeset=226391_at) | [NA](http://www.ncbi.nlm.nih.gov/entrez/query.fcgi?cmd=search&db=gene&term=NA) | NA | **0.694** | **134.707** | 55.039 | **93.538** | 37.560 | 3.055E-02 |
| [223493_at](https://www.affymetrix.com/LinkServlet?probeset=223493_at) | [FBXO4](http://www.ncbi.nlm.nih.gov/entrez/query.fcgi?cmd=search&db=gene&term=FBXO4) | F-box protein 4 | **0.695** | **156.768** | 39.824 | **108.957** | 31.984 | 1.412E-03 |
| [210124_x_at](https://www.affymetrix.com/LinkServlet?probeset=210124_x_at) | [SEMA4F](http://www.ncbi.nlm.nih.gov/entrez/query.fcgi?cmd=search&db=gene&term=SEMA4F) | sema domain, immunoglobulin domain (Ig), transmembrane domain (TM) and short cytoplasmic domain, (semaphorin) 4F | **0.695** | **41.167** | 19.552 | **28.614** | 13.329 | 3.279E-02 |
| [221135_s_at](https://www.affymetrix.com/LinkServlet?probeset=221135_s_at) | [ASTE1](http://www.ncbi.nlm.nih.gov/entrez/query.fcgi?cmd=search&db=gene&term=ASTE1) | asteroid homolog 1 (Drosophila) | **0.695** | **159.130** | 61.908 | **110.614** | 47.197 | 3.645E-02 |
| [203276_at](https://www.affymetrix.com/LinkServlet?probeset=203276_at) | [LMNB1](http://www.ncbi.nlm.nih.gov/entrez/query.fcgi?cmd=search&db=gene&term=LMNB1) | lamin B1 | **0.695** | **370.095** | 85.746 | **257.263** | 68.573 | 6.040E-04 |
| [235282_at](https://www.affymetrix.com/LinkServlet?probeset=235282_at) | [NA](http://www.ncbi.nlm.nih.gov/entrez/query.fcgi?cmd=search&db=gene&term=NA) | NA | **0.696** | **45.966** | 17.994 | **31.973** | 31.692 | 1.471E-02 |
| [223144_s_at](https://www.affymetrix.com/LinkServlet?probeset=223144_s_at) | [AKIRIN2](http://www.ncbi.nlm.nih.gov/entrez/query.fcgi?cmd=search&db=gene&term=AKIRIN2) | akirin 2 | **0.696** | **153.860** | 52.220 | **107.156** | 37.604 | 2.923E-02 |
| [224153_s_at](https://www.affymetrix.com/LinkServlet?probeset=224153_s_at) | [C14orf167](http://www.ncbi.nlm.nih.gov/entrez/query.fcgi?cmd=search&db=gene&term=C14orf167) | chromosome 14 open reading frame 167 | **0.698** | **82.586** | 28.726 | **57.613** | 16.692 | 9.948E-03 |
| [214706_at](https://www.affymetrix.com/LinkServlet?probeset=214706_at) | [ZNF200](http://www.ncbi.nlm.nih.gov/entrez/query.fcgi?cmd=search&db=gene&term=ZNF200) | zinc finger protein 200 | **0.698** | **40.129** | 16.807 | **27.994** | 19.976 | 3.403E-02 |
| [219426_at](https://www.affymetrix.com/LinkServlet?probeset=219426_at) | [EIF2C3](http://www.ncbi.nlm.nih.gov/entrez/query.fcgi?cmd=search&db=gene&term=EIF2C3) | eukaryotic translation initiation factor 2C, 3 | **0.698** | **33.290** | 12.405 | **23.227** | 15.904 | 3.308E-02 |
| [204264_at](https://www.affymetrix.com/LinkServlet?probeset=204264_at) | [CPT2](http://www.ncbi.nlm.nih.gov/entrez/query.fcgi?cmd=search&db=gene&term=CPT2) | carnitine palmitoyltransferase 2 | **0.698** | **85.827** | 28.706 | **59.886** | 19.500 | 2.770E-02 |
| [1556151_at](https://www.affymetrix.com/LinkServlet?probeset=1556151_at) | [NA](http://www.ncbi.nlm.nih.gov/entrez/query.fcgi?cmd=search&db=gene&term=NA) | NA | **0.698** | **497.966** | 179.513 | **347.492** | 159.175 | 2.527E-02 |
| [204412_s_at](https://www.affymetrix.com/LinkServlet?probeset=204412_s_at) | [NEFH](http://www.ncbi.nlm.nih.gov/entrez/query.fcgi?cmd=search&db=gene&term=NEFH) | neurofilament, heavy polypeptide | **0.698** | **15.499** | 6.361 | **10.823** | 6.196 | 4.075E-02 |
| [226655_at](https://www.affymetrix.com/LinkServlet?probeset=226655_at) | [STX17](http://www.ncbi.nlm.nih.gov/entrez/query.fcgi?cmd=search&db=gene&term=STX17) | syntaxin 17 | **0.698** | **15.325** | 5.733 | **10.703** | 6.485 | 3.530E-02 |
| [206800_at](https://www.affymetrix.com/LinkServlet?probeset=206800_at) | [MTHFR](http://www.ncbi.nlm.nih.gov/entrez/query.fcgi?cmd=search&db=gene&term=MTHFR) | methylenetetrahydrofolate reductase (NAD(P)H) | **0.699** | **9.696** | 3.909 | **6.780** | 1.112 | 2.264E-02 |
| [223477_s_at](https://www.affymetrix.com/LinkServlet?probeset=223477_s_at) | [C12orf65](http://www.ncbi.nlm.nih.gov/entrez/query.fcgi?cmd=search&db=gene&term=C12orf65) | chromosome 12 open reading frame 65 | **0.699** | **209.951** | 47.003 | **146.820** | 55.309 | 3.254E-03 |
| [226774_at](https://www.affymetrix.com/LinkServlet?probeset=226774_at) | [FAM120B](http://www.ncbi.nlm.nih.gov/entrez/query.fcgi?cmd=search&db=gene&term=FAM120B) | family with sequence similarity 120B | **0.700** | **69.138** | 28.673 | **48.367** | 17.705 | 2.093E-02 |
| [227338_at](https://www.affymetrix.com/LinkServlet?probeset=227338_at) | [NA](http://www.ncbi.nlm.nih.gov/entrez/query.fcgi?cmd=search&db=gene&term=NA) | NA | **0.700** | **449.925** | 116.684 | **314.760** | 108.155 | 5.145E-03 |
| [226472_at](https://www.affymetrix.com/LinkServlet?probeset=226472_at) | [PPIL4](http://www.ncbi.nlm.nih.gov/entrez/query.fcgi?cmd=search&db=gene&term=PPIL4) | peptidylprolyl isomerase (cyclophilin)-like 4 | **0.700** | **136.610** | 40.837 | **95.572** | 28.633 | 5.113E-03 |
| [1555893_at](https://www.affymetrix.com/LinkServlet?probeset=1555893_at) | [NA](http://www.ncbi.nlm.nih.gov/entrez/query.fcgi?cmd=search&db=gene&term=NA) | NA | **0.700** | **9.024** | 3.281 | **6.314** | 0.812 | 8.464E-03 |
| [222505_at](https://www.affymetrix.com/LinkServlet?probeset=222505_at) | [LMBR1](http://www.ncbi.nlm.nih.gov/entrez/query.fcgi?cmd=search&db=gene&term=LMBR1) | limb region 1 homolog (mouse) | **0.701** | **63.185** | 21.152 | **44.281** | 29.573 | 2.199E-02 |
| [228660_x_at](https://www.affymetrix.com/LinkServlet?probeset=228660_x_at) | [SEMA4F](http://www.ncbi.nlm.nih.gov/entrez/query.fcgi?cmd=search&db=gene&term=SEMA4F) | sema domain, immunoglobulin domain (Ig), transmembrane domain (TM) and short cytoplasmic domain, (semaphorin) 4F | **0.702** | **35.943** | 16.411 | **25.238** | 12.180 | 3.809E-02 |
| [223886_s_at](https://www.affymetrix.com/LinkServlet?probeset=223886_s_at) | [RNF146](http://www.ncbi.nlm.nih.gov/entrez/query.fcgi?cmd=search&db=gene&term=RNF146) | ring finger protein 146 | **0.702** | **486.261** | 147.418 | **341.453** | 78.681 | 7.079E-03 |
| [240549_at](https://www.affymetrix.com/LinkServlet?probeset=240549_at) | [NA](http://www.ncbi.nlm.nih.gov/entrez/query.fcgi?cmd=search&db=gene&term=NA) | NA | **0.703** | **18.619** | 8.107 | **13.081** | 9.889 | 4.704E-02 |
| [201555_at](https://www.affymetrix.com/LinkServlet?probeset=201555_at) | [MCM3](http://www.ncbi.nlm.nih.gov/entrez/query.fcgi?cmd=search&db=gene&term=MCM3) | minichromosome maintenance complex component 3 | **0.703** | **262.452** | 113.537 | **184.440** | 78.272 | 3.341E-02 |
| [204504_s_at](https://www.affymetrix.com/LinkServlet?probeset=204504_s_at) | [HIRIP3](http://www.ncbi.nlm.nih.gov/entrez/query.fcgi?cmd=search&db=gene&term=HIRIP3) | HIRA interacting protein 3 | **0.703** | **67.338** | 26.208 | **47.365** | 21.696 | 4.004E-02 |
| [207113_s_at](https://www.affymetrix.com/LinkServlet?probeset=207113_s_at) | [TNF](http://www.ncbi.nlm.nih.gov/entrez/query.fcgi?cmd=search&db=gene&term=TNF) | tumor necrosis factor | **0.705** | **135.647** | 59.289 | **95.572** | 38.775 | 4.599E-02 |
| [230026_at](https://www.affymetrix.com/LinkServlet?probeset=230026_at) | [MRPL43](http://www.ncbi.nlm.nih.gov/entrez/query.fcgi?cmd=search&db=gene&term=MRPL43) | mitochondrial ribosomal protein L43 | **0.705** | **144.078** | 69.682 | **101.620** | 105.003 | 4.544E-02 |
| [226760_at](https://www.affymetrix.com/LinkServlet?probeset=226760_at) | [MBTPS2](http://www.ncbi.nlm.nih.gov/entrez/query.fcgi?cmd=search&db=gene&term=MBTPS2) | membrane-bound transcription factor peptidase, site 2 | **0.706** | **142.269** | 43.228 | **100.409** | 35.275 | 6.048E-03 |
| [229268_at](https://www.affymetrix.com/LinkServlet?probeset=229268_at) | [FAM105B](http://www.ncbi.nlm.nih.gov/entrez/query.fcgi?cmd=search&db=gene&term=FAM105B) | family with sequence similarity 105, member B | **0.706** | **231.701** | 98.363 | **163.533** | 95.371 | 3.309E-02 |
| [242130_at](https://www.affymetrix.com/LinkServlet?probeset=242130_at) | [NA](http://www.ncbi.nlm.nih.gov/entrez/query.fcgi?cmd=search&db=gene&term=NA) | NA | **0.706** | **9.668** | 3.812 | **6.824** | 1.215 | 2.620E-02 |
| [204876_at](https://www.affymetrix.com/LinkServlet?probeset=204876_at) | [ZNF646](http://www.ncbi.nlm.nih.gov/entrez/query.fcgi?cmd=search&db=gene&term=ZNF646) | zinc finger protein 646 | **0.707** | **26.201** | 8.304 | **18.514** | 14.650 | 1.558E-02 |
| [212478_at](https://www.affymetrix.com/LinkServlet?probeset=212478_at) | [RMND5A](http://www.ncbi.nlm.nih.gov/entrez/query.fcgi?cmd=search&db=gene&term=RMND5A) | required for meiotic nuclear division 5 homolog A (S. cerevisiae) | **0.707** | **21.139** | 8.353 | **14.938** | 8.045 | 4.213E-02 |
| [227228_s_at](https://www.affymetrix.com/LinkServlet?probeset=227228_s_at) | [CCDC88C](http://www.ncbi.nlm.nih.gov/entrez/query.fcgi?cmd=search&db=gene&term=CCDC88C) | coiled-coil domain containing 88C | **0.707** | **168.152** | 62.365 | **118.844** | 37.072 | 2.332E-02 |
| [226819_at](https://www.affymetrix.com/LinkServlet?probeset=226819_at) | [NA](http://www.ncbi.nlm.nih.gov/entrez/query.fcgi?cmd=search&db=gene&term=NA) | NA | **0.707** | **9.325** | 4.027 | **6.593** | 0.878 | 3.873E-02 |
| [228604_at](https://www.affymetrix.com/LinkServlet?probeset=228604_at) | [FAM76A](http://www.ncbi.nlm.nih.gov/entrez/query.fcgi?cmd=search&db=gene&term=FAM76A) | family with sequence similarity 76, member A | **0.708** | **110.554** | 46.191 | **78.314** | 26.795 | 2.729E-02 |
| [217692_at](https://www.affymetrix.com/LinkServlet?probeset=217692_at) | [MAGOH2](http://www.ncbi.nlm.nih.gov/entrez/query.fcgi?cmd=search&db=gene&term=MAGOH2) | mago-nashi homolog 2, proliferation-associated (Drosophila) | **0.709** | **27.288** | 12.608 | **19.344** | 5.292 | 2.202E-02 |
| [233881_s_at](https://www.affymetrix.com/LinkServlet?probeset=233881_s_at) | [TOLLIP](http://www.ncbi.nlm.nih.gov/entrez/query.fcgi?cmd=search&db=gene&term=TOLLIP) | toll interacting protein | **0.711** | **64.656** | 25.388 | **45.941** | 22.083 | 3.841E-02 |
| [1556698_a_at](https://www.affymetrix.com/LinkServlet?probeset=1556698_a_at) | [GPRIN3](http://www.ncbi.nlm.nih.gov/entrez/query.fcgi?cmd=search&db=gene&term=GPRIN3) | GPRIN family member 3 | **0.711** | **183.404** | 45.744 | **130.370** | 62.758 | 8.150E-03 |
| [218900_at](https://www.affymetrix.com/LinkServlet?probeset=218900_at) | [CNNM4](http://www.ncbi.nlm.nih.gov/entrez/query.fcgi?cmd=search&db=gene&term=CNNM4) | cyclin M4 | **0.711** | **55.531** | 31.303 | **39.487** | 14.521 | 4.090E-02 |
| [225957_at](https://www.affymetrix.com/LinkServlet?probeset=225957_at) | [C5orf41](http://www.ncbi.nlm.nih.gov/entrez/query.fcgi?cmd=search&db=gene&term=C5orf41) | chromosome 5 open reading frame 41 | **0.711** | **179.607** | 66.849 | **127.737** | 45.655 | 4.192E-02 |
| [202881_x_at](https://www.affymetrix.com/LinkServlet?probeset=202881_x_at) | [NOL7](http://www.ncbi.nlm.nih.gov/entrez/query.fcgi?cmd=search&db=gene&term=NOL7) | nucleolar protein 7, 27kDa | **0.711** | **9.761** | 3.208 | **6.945** | 1.380 | 7.702E-03 |
| [214820_at](https://www.affymetrix.com/LinkServlet?probeset=214820_at) | [BRWD1](http://www.ncbi.nlm.nih.gov/entrez/query.fcgi?cmd=search&db=gene&term=BRWD1) | bromodomain and WD repeat domain containing 1 | **0.713** | **91.498** | 33.863 | **65.225** | 47.110 | 4.232E-02 |
| [201746_at](https://www.affymetrix.com/LinkServlet?probeset=201746_at) | [TP53](http://www.ncbi.nlm.nih.gov/entrez/query.fcgi?cmd=search&db=gene&term=TP53) | tumor protein p53 | **0.714** | **1068.585** | 294.517 | **762.579** | 177.645 | 4.209E-03 |
| [215113_s_at](https://www.affymetrix.com/LinkServlet?probeset=215113_s_at) | [SENP3](http://www.ncbi.nlm.nih.gov/entrez/query.fcgi?cmd=search&db=gene&term=SENP3) | SUMO1/sentrin/SMT3 specific peptidase 3 | **0.714** | **25.019** | 9.028 | **17.875** | 9.944 | 1.707E-02 |
| [214766_s_at](https://www.affymetrix.com/LinkServlet?probeset=214766_s_at) | [AHCTF1](http://www.ncbi.nlm.nih.gov/entrez/query.fcgi?cmd=search&db=gene&term=AHCTF1) | AT hook containing transcription factor 1 | **0.715** | **192.969** | 70.023 | **137.944** | 44.594 | 1.858E-02 |
| [244551_at](https://www.affymetrix.com/LinkServlet?probeset=244551_at) | [NA](http://www.ncbi.nlm.nih.gov/entrez/query.fcgi?cmd=search&db=gene&term=NA) | NA | **0.715** | **49.968** | 19.001 | **35.730** | 18.589 | 4.205E-02 |
| [215030_at](https://www.affymetrix.com/LinkServlet?probeset=215030_at) | [GRSF1](http://www.ncbi.nlm.nih.gov/entrez/query.fcgi?cmd=search&db=gene&term=GRSF1) | G-rich RNA sequence binding factor 1 | **0.715** | **149.128** | 47.303 | **106.661** | 56.511 | 2.140E-02 |
| [212838_at](https://www.affymetrix.com/LinkServlet?probeset=212838_at) | [DNMBP](http://www.ncbi.nlm.nih.gov/entrez/query.fcgi?cmd=search&db=gene&term=DNMBP) | dynamin binding protein | **0.716** | **200.402** | 60.450 | **143.452** | 54.904 | 1.521E-02 |
| [231764_at](https://www.affymetrix.com/LinkServlet?probeset=231764_at) | [CHRAC1](http://www.ncbi.nlm.nih.gov/entrez/query.fcgi?cmd=search&db=gene&term=CHRAC1) | chromatin accessibility complex 1 | **0.716** | **208.204** | 98.684 | **149.153** | 58.179 | 4.296E-02 |
| [215307_at](https://www.affymetrix.com/LinkServlet?probeset=215307_at) | [ZNF529](http://www.ncbi.nlm.nih.gov/entrez/query.fcgi?cmd=search&db=gene&term=ZNF529) | zinc finger protein 529 | **0.716** | **130.945** | 44.526 | **93.818** | 39.686 | 2.299E-02 |
| [205961_s_at](https://www.affymetrix.com/LinkServlet?probeset=205961_s_at) | [PSIP1](http://www.ncbi.nlm.nih.gov/entrez/query.fcgi?cmd=search&db=gene&term=PSIP1) | PC4 and SFRS1 interacting protein 1 | **0.717** | **515.187** | 123.579 | **369.364** | 101.652 | 2.278E-03 |
| [205006_s_at](https://www.affymetrix.com/LinkServlet?probeset=205006_s_at) | [NMT2](http://www.ncbi.nlm.nih.gov/entrez/query.fcgi?cmd=search&db=gene&term=NMT2) | N-myristoyltransferase 2 | **0.717** | **299.778** | 82.401 | **214.982** | 66.238 | 6.961E-03 |
| [203991_s_at](https://www.affymetrix.com/LinkServlet?probeset=203991_s_at) | [KDM6A](http://www.ncbi.nlm.nih.gov/entrez/query.fcgi?cmd=search&db=gene&term=KDM6A) | lysine (K)-specific demethylase 6A | **0.717** | **165.981** | 45.474 | **119.035** | 53.863 | 1.941E-02 |
| [244834_at](https://www.affymetrix.com/LinkServlet?probeset=244834_at) | [C1orf134](http://www.ncbi.nlm.nih.gov/entrez/query.fcgi?cmd=search&db=gene&term=C1orf134) | chromosome 1 open reading frame 134 | **0.718** | **94.425** | 29.309 | **67.755** | 25.693 | 2.024E-02 |
| [215747_s_at](https://www.affymetrix.com/LinkServlet?probeset=215747_s_at) | [RCC1](http://www.ncbi.nlm.nih.gov/entrez/query.fcgi?cmd=search&db=gene&term=RCC1) | regulator of chromosome condensation 1 | **0.718** | **20.638** | 4.227 | **14.811** | 8.195 | 7.542E-03 |
| [205189_s_at](https://www.affymetrix.com/LinkServlet?probeset=205189_s_at) | [FANCC](http://www.ncbi.nlm.nih.gov/entrez/query.fcgi?cmd=search&db=gene&term=FANCC) | Fanconi anemia, complementation group C | **0.718** | **18.841** | 5.685 | **13.535** | 8.223 | 1.128E-02 |
| [212310_at](https://www.affymetrix.com/LinkServlet?probeset=212310_at) | [MIA3](http://www.ncbi.nlm.nih.gov/entrez/query.fcgi?cmd=search&db=gene&term=MIA3) | melanoma inhibitory activity family, member 3 | **0.718** | **205.339** | 53.102 | **147.512** | 54.247 | 3.633E-03 |
| [238330_s_at](https://www.affymetrix.com/LinkServlet?probeset=238330_s_at) | [MPRIP](http://www.ncbi.nlm.nih.gov/entrez/query.fcgi?cmd=search&db=gene&term=MPRIP) | myosin phosphatase Rho interacting protein | **0.719** | **11.116** | 4.459 | **7.992** | 2.407 | 3.482E-02 |
| [203440_at](https://www.affymetrix.com/LinkServlet?probeset=203440_at) | [CDH2](http://www.ncbi.nlm.nih.gov/entrez/query.fcgi?cmd=search&db=gene&term=CDH2) | cadherin 2, type 1, N-cadherin (neuronal) | **0.719** | **20.476** | 10.061 | **14.726** | 16.916 | 4.338E-02 |
| [206499_s_at](https://www.affymetrix.com/LinkServlet?probeset=206499_s_at) | [RCC1](http://www.ncbi.nlm.nih.gov/entrez/query.fcgi?cmd=search&db=gene&term=RCC1) | regulator of chromosome condensation 1 | **0.719** | **153.331** | 51.339 | **110.290** | 30.918 | 2.028E-02 |
| [65585_at](https://www.affymetrix.com/LinkServlet?probeset=65585_at) | [FAM86B1](http://www.ncbi.nlm.nih.gov/entrez/query.fcgi?cmd=search&db=gene&term=FAM86B1) | family with sequence similarity 86, member B1 | **0.719** | **99.606** | 56.684 | **71.666** | 31.036 | 4.844E-02 |
| [212570_at](https://www.affymetrix.com/LinkServlet?probeset=212570_at) | [ENDOD1](http://www.ncbi.nlm.nih.gov/entrez/query.fcgi?cmd=search&db=gene&term=ENDOD1) | endonuclease domain containing 1 | **0.721** | **47.366** | 17.323 | **34.128** | 12.125 | 2.106E-02 |
| [201013_s_at](https://www.affymetrix.com/LinkServlet?probeset=201013_s_at) | [PAICS](http://www.ncbi.nlm.nih.gov/entrez/query.fcgi?cmd=search&db=gene&term=PAICS) | phosphoribosylaminoimidazole carboxylase, phosphoribosylaminoimidazole succinocarboxamide synthetase | **0.721** | **475.824** | 160.377 | **343.110** | 86.072 | 1.297E-02 |
| [222317_at](https://www.affymetrix.com/LinkServlet?probeset=222317_at) | [PDE3B](http://www.ncbi.nlm.nih.gov/entrez/query.fcgi?cmd=search&db=gene&term=PDE3B) | phosphodiesterase 3B, cGMP-inhibited | **0.722** | **192.879** | 69.617 | **139.224** | 47.790 | 1.957E-02 |
| [214260_at](https://www.affymetrix.com/LinkServlet?probeset=214260_at) | [COPS8](http://www.ncbi.nlm.nih.gov/entrez/query.fcgi?cmd=search&db=gene&term=COPS8) | COP9 constitutive photomorphogenic homolog subunit 8 (Arabidopsis) | **0.722** | **11.005** | 4.597 | **7.944** | 3.698 | 3.736E-02 |
| [218759_at](https://www.affymetrix.com/LinkServlet?probeset=218759_at) | [DVL2](http://www.ncbi.nlm.nih.gov/entrez/query.fcgi?cmd=search&db=gene&term=DVL2) | dishevelled, dsh homolog 2 (Drosophila) | **0.722** | **19.301** | 5.430 | **13.937** | 4.455 | 6.999E-03 |
| [1554569_a_at](https://www.affymetrix.com/LinkServlet?probeset=1554569_a_at) | [CELF2](http://www.ncbi.nlm.nih.gov/entrez/query.fcgi?cmd=search&db=gene&term=CELF2) | CUGBP, Elav-like family member 2 | **0.722** | **97.460** | 45.928 | **70.381** | 35.497 | 4.448E-02 |
| [231912_s_at](https://www.affymetrix.com/LinkServlet?probeset=231912_s_at) | [TECPR1](http://www.ncbi.nlm.nih.gov/entrez/query.fcgi?cmd=search&db=gene&term=TECPR1) | tectonin beta-propeller repeat containing 1 | **0.722** | **160.935** | 64.310 | **116.257** | 71.559 | 4.502E-02 |
| [202073_at](https://www.affymetrix.com/LinkServlet?probeset=202073_at) | [OPTN](http://www.ncbi.nlm.nih.gov/entrez/query.fcgi?cmd=search&db=gene&term=OPTN) | optineurin | **0.722** | **497.427** | 112.805 | **359.355** | 124.374 | 4.573E-03 |
| [216381_x_at](https://www.affymetrix.com/LinkServlet?probeset=216381_x_at) | [AKR7A3](http://www.ncbi.nlm.nih.gov/entrez/query.fcgi?cmd=search&db=gene&term=AKR7A3) | aldo-keto reductase family 7, member A3 (aflatoxin aldehyde reductase) | **0.723** | **40.309** | 14.426 | **29.139** | 10.869 | 1.855E-02 |
| [243074_at](https://www.affymetrix.com/LinkServlet?probeset=243074_at) | [NA](http://www.ncbi.nlm.nih.gov/entrez/query.fcgi?cmd=search&db=gene&term=NA) | NA | **0.723** | **8.594** | 4.406 | **6.215** | 0.519 | 4.790E-02 |
| [234708_at](https://www.affymetrix.com/LinkServlet?probeset=234708_at) | [SMUG1](http://www.ncbi.nlm.nih.gov/entrez/query.fcgi?cmd=search&db=gene&term=SMUG1) | single-strand-selective monofunctional uracil-DNA glycosylase 1 | **0.723** | **26.128** | 7.554 | **18.900** | 11.948 | 1.690E-02 |
| [234021_at](https://www.affymetrix.com/LinkServlet?probeset=234021_at) | [EML2](http://www.ncbi.nlm.nih.gov/entrez/query.fcgi?cmd=search&db=gene&term=EML2) | echinoderm microtubule associated protein like 2 | **0.724** | **8.290** | 3.610 | **6.000** | 0.000 | 2.452E-02 |
| [209413_at](https://www.affymetrix.com/LinkServlet?probeset=209413_at) | [B4GALT2](http://www.ncbi.nlm.nih.gov/entrez/query.fcgi?cmd=search&db=gene&term=B4GALT2) | UDP-Gal:betaGlcNAc beta 1,4- galactosyltransferase, polypeptide 2 | **0.724** | **12.391** | 4.458 | **8.970** | 3.981 | 2.619E-02 |
| [219089_s_at](https://www.affymetrix.com/LinkServlet?probeset=219089_s_at) | [ZNF576](http://www.ncbi.nlm.nih.gov/entrez/query.fcgi?cmd=search&db=gene&term=ZNF576) | zinc finger protein 576 | **0.725** | **25.186** | 9.636 | **18.254** | 7.503 | 3.618E-02 |
| [238866_at](https://www.affymetrix.com/LinkServlet?probeset=238866_at) | [C19orf68](http://www.ncbi.nlm.nih.gov/entrez/query.fcgi?cmd=search&db=gene&term=C19orf68) | chromosome 19 open reading frame 68 | **0.725** | **39.893** | 18.004 | **28.919** | 11.027 | 4.628E-02 |
| [1557945_at](https://www.affymetrix.com/LinkServlet?probeset=1557945_at) | [TCTE3](http://www.ncbi.nlm.nih.gov/entrez/query.fcgi?cmd=search&db=gene&term=TCTE3) | t-complex-associated-testis-expressed 3 | **0.725** | **63.346** | 33.749 | **45.948** | 43.785 | 3.843E-02 |
| [215510_at](https://www.affymetrix.com/LinkServlet?probeset=215510_at) | [ETV2](http://www.ncbi.nlm.nih.gov/entrez/query.fcgi?cmd=search&db=gene&term=ETV2) | ets variant 2 | **0.725** | **17.824** | 7.049 | **12.930** | 10.415 | 3.251E-02 |
| [221603_at](https://www.affymetrix.com/LinkServlet?probeset=221603_at) | [PEX16](http://www.ncbi.nlm.nih.gov/entrez/query.fcgi?cmd=search&db=gene&term=PEX16) | peroxisomal biogenesis factor 16 | **0.725** | **10.522** | 4.284 | **7.634** | 2.721 | 4.379E-02 |
| [225387_at](https://www.affymetrix.com/LinkServlet?probeset=225387_at) | [TSPAN5](http://www.ncbi.nlm.nih.gov/entrez/query.fcgi?cmd=search&db=gene&term=TSPAN5) | tetraspanin 5 | **0.727** | **215.002** | 64.023 | **156.295** | 46.506 | 1.118E-02 |
| [214440_at](https://www.affymetrix.com/LinkServlet?probeset=214440_at) | [NAT1](http://www.ncbi.nlm.nih.gov/entrez/query.fcgi?cmd=search&db=gene&term=NAT1) | N-acetyltransferase 1 (arylamine N-acetyltransferase) | **0.727** | **128.217** | 36.145 | **93.231** | 34.990 | 4.588E-02 |
| [235625_at](https://www.affymetrix.com/LinkServlet?probeset=235625_at) | [VPS41](http://www.ncbi.nlm.nih.gov/entrez/query.fcgi?cmd=search&db=gene&term=VPS41) | vacuolar protein sorting 41 homolog (S. cerevisiae) | **0.727** | **258.822** | 78.628 | **188.286** | 20.964 | 1.265E-02 |
| [241534_at](https://www.affymetrix.com/LinkServlet?probeset=241534_at) | [LOC100505549](http://www.ncbi.nlm.nih.gov/entrez/query.fcgi?cmd=search&db=gene&term=LOC100505549) | hypothetical LOC100505549 | **0.728** | **85.837** | 27.829 | **62.475** | 29.849 | 3.240E-02 |
| [228423_at](https://www.affymetrix.com/LinkServlet?probeset=228423_at) | [MAP9](http://www.ncbi.nlm.nih.gov/entrez/query.fcgi?cmd=search&db=gene&term=MAP9) | microtubule-associated protein 9 | **0.728** | **128.364** | 56.316 | **93.438** | 43.817 | 4.089E-02 |
| [243612_at](https://www.affymetrix.com/LinkServlet?probeset=243612_at) | [NSD1](http://www.ncbi.nlm.nih.gov/entrez/query.fcgi?cmd=search&db=gene&term=NSD1) | nuclear receptor binding SET domain protein 1 | **0.729** | **16.296** | 5.210 | **11.872** | 7.965 | 1.759E-02 |
| [37831_at](https://www.affymetrix.com/LinkServlet?probeset=37831_at) | [SIPA1L3](http://www.ncbi.nlm.nih.gov/entrez/query.fcgi?cmd=search&db=gene&term=SIPA1L3) | signal-induced proliferation-associated 1 like 3 | **0.729** | **78.333** | 21.351 | **57.079** | 21.340 | 1.540E-02 |
| [212017_at](https://www.affymetrix.com/LinkServlet?probeset=212017_at) | [FAM168B](http://www.ncbi.nlm.nih.gov/entrez/query.fcgi?cmd=search&db=gene&term=FAM168B) | family with sequence similarity 168, member B | **0.730** | **211.564** | 66.702 | **154.435** | 57.509 | 1.887E-02 |
| [1556468_at](https://www.affymetrix.com/LinkServlet?probeset=1556468_at) | [NA](http://www.ncbi.nlm.nih.gov/entrez/query.fcgi?cmd=search&db=gene&term=NA) | NA | **0.730** | **8.909** | 3.331 | **6.506** | 1.219 | 2.580E-02 |
| [204902_s_at](https://www.affymetrix.com/LinkServlet?probeset=204902_s_at) | [ATG4B](http://www.ncbi.nlm.nih.gov/entrez/query.fcgi?cmd=search&db=gene&term=ATG4B) | ATG4 autophagy related 4 homolog B (S. cerevisiae) | **0.731** | **68.751** | 25.491 | **50.256** | 24.679 | 4.581E-02 |
| [1554176_a_at](https://www.affymetrix.com/LinkServlet?probeset=1554176_a_at) | [C3orf33](http://www.ncbi.nlm.nih.gov/entrez/query.fcgi?cmd=search&db=gene&term=C3orf33) | chromosome 3 open reading frame 33 | **0.731** | **43.771** | 16.961 | **31.999** | 30.408 | 2.355E-02 |
| [204176_at](https://www.affymetrix.com/LinkServlet?probeset=204176_at) | [KLHL20](http://www.ncbi.nlm.nih.gov/entrez/query.fcgi?cmd=search&db=gene&term=KLHL20) | kelch-like 20 (Drosophila) | **0.731** | **48.358** | 14.145 | **35.372** | 17.482 | 2.001E-02 |
| [219515_at](https://www.affymetrix.com/LinkServlet?probeset=219515_at) | [PRDM10](http://www.ncbi.nlm.nih.gov/entrez/query.fcgi?cmd=search&db=gene&term=PRDM10) | PR domain containing 10 | **0.732** | **182.287** | 65.103 | **133.355** | 42.286 | 2.984E-02 |
| [200999_s_at](https://www.affymetrix.com/LinkServlet?probeset=200999_s_at) | [CKAP4](http://www.ncbi.nlm.nih.gov/entrez/query.fcgi?cmd=search&db=gene&term=CKAP4) | cytoskeleton-associated protein 4 | **0.732** | **577.469** | 216.319 | **422.765** | 104.885 | 3.015E-02 |
| [233827_s_at](https://www.affymetrix.com/LinkServlet?probeset=233827_s_at) | [SUPT16H](http://www.ncbi.nlm.nih.gov/entrez/query.fcgi?cmd=search&db=gene&term=SUPT16H) | suppressor of Ty 16 homolog (S. cerevisiae) | **0.733** | **107.895** | 30.023 | **79.119** | 28.456 | 1.935E-02 |
| [229867_at](https://www.affymetrix.com/LinkServlet?probeset=229867_at) | [BTBD9](http://www.ncbi.nlm.nih.gov/entrez/query.fcgi?cmd=search&db=gene&term=BTBD9) | BTB (POZ) domain containing 9 | **0.734** | **64.394** | 14.474 | **47.246** | 18.093 | 1.529E-02 |
| [219012_s_at](https://www.affymetrix.com/LinkServlet?probeset=219012_s_at) | [C11orf30](http://www.ncbi.nlm.nih.gov/entrez/query.fcgi?cmd=search&db=gene&term=C11orf30) | chromosome 11 open reading frame 30 | **0.734** | **63.741** | 22.031 | **46.780** | 19.195 | 4.610E-02 |
| [33767_at](https://www.affymetrix.com/LinkServlet?probeset=33767_at) | [NEFH](http://www.ncbi.nlm.nih.gov/entrez/query.fcgi?cmd=search&db=gene&term=NEFH) | neurofilament, heavy polypeptide | **0.734** | **22.452** | 7.577 | **16.481** | 11.721 | 4.418E-02 |
| [203068_at](https://www.affymetrix.com/LinkServlet?probeset=203068_at) | [KLHL21](http://www.ncbi.nlm.nih.gov/entrez/query.fcgi?cmd=search&db=gene&term=KLHL21) | kelch-like 21 (Drosophila) | **0.734** | **416.308** | 122.170 | **305.747** | 88.650 | 1.818E-02 |
| [222604_at](https://www.affymetrix.com/LinkServlet?probeset=222604_at) | [GTF3C3](http://www.ncbi.nlm.nih.gov/entrez/query.fcgi?cmd=search&db=gene&term=GTF3C3) | general transcription factor IIIC, polypeptide 3, 102kDa | **0.735** | **309.040** | 98.391 | **227.009** | 63.751 | 1.046E-02 |
| [216304_x_at](https://www.affymetrix.com/LinkServlet?probeset=216304_x_at) | [YME1L1](http://www.ncbi.nlm.nih.gov/entrez/query.fcgi?cmd=search&db=gene&term=YME1L1) | YME1-like 1 (S. cerevisiae) | **0.735** | **247.724** | 100.097 | **181.997** | 34.797 | 1.343E-02 |
| [1555761_x_at](https://www.affymetrix.com/LinkServlet?probeset=1555761_x_at) | [RBM15](http://www.ncbi.nlm.nih.gov/entrez/query.fcgi?cmd=search&db=gene&term=RBM15) | RNA binding motif protein 15 | **0.735** | **11.898** | 2.431 | **8.742** | 2.759 | 1.856E-03 |
| [240229_at](https://www.affymetrix.com/LinkServlet?probeset=240229_at) | [NA](http://www.ncbi.nlm.nih.gov/entrez/query.fcgi?cmd=search&db=gene&term=NA) | NA | **0.735** | **11.719** | 4.144 | **8.617** | 2.751 | 3.877E-02 |
| [221596_s_at](https://www.affymetrix.com/LinkServlet?probeset=221596_s_at) | [C7orf64](http://www.ncbi.nlm.nih.gov/entrez/query.fcgi?cmd=search&db=gene&term=C7orf64) | chromosome 7 open reading frame 64 | **0.736** | **291.406** | 72.298 | **214.503** | 65.858 | 4.878E-03 |
| [223114_at](https://www.affymetrix.com/LinkServlet?probeset=223114_at) | [COQ5](http://www.ncbi.nlm.nih.gov/entrez/query.fcgi?cmd=search&db=gene&term=COQ5) | coenzyme Q5 homolog, methyltransferase (S. cerevisiae) | **0.736** | **203.300** | 51.874 | **149.683** | 29.551 | 3.358E-03 |
| [1555501_s_at](https://www.affymetrix.com/LinkServlet?probeset=1555501_s_at) | [RSRC1](http://www.ncbi.nlm.nih.gov/entrez/query.fcgi?cmd=search&db=gene&term=RSRC1) | arginine/serine-rich coiled-coil 1 | **0.736** | **42.396** | 15.213 | **31.215** | 19.133 | 3.259E-02 |
| [238863_x_at](https://www.affymetrix.com/LinkServlet?probeset=238863_x_at) | [NA](http://www.ncbi.nlm.nih.gov/entrez/query.fcgi?cmd=search&db=gene&term=NA) | NA | **0.736** | **194.258** | 36.470 | **143.031** | 47.746 | 2.926E-03 |
| [229298_at](https://www.affymetrix.com/LinkServlet?probeset=229298_at) | [NA](http://www.ncbi.nlm.nih.gov/entrez/query.fcgi?cmd=search&db=gene&term=NA) | NA | **0.737** | **106.122** | 39.626 | **78.174** | 24.809 | 2.534E-02 |
| [214934_at](https://www.affymetrix.com/LinkServlet?probeset=214934_at) | [ATP9B](http://www.ncbi.nlm.nih.gov/entrez/query.fcgi?cmd=search&db=gene&term=ATP9B) | ATPase, class II, type 9B | **0.737** | **456.097** | 137.223 | **336.166** | 133.076 | 2.054E-02 |
| [244286_at](https://www.affymetrix.com/LinkServlet?probeset=244286_at) | [NA](http://www.ncbi.nlm.nih.gov/entrez/query.fcgi?cmd=search&db=gene&term=NA) | NA | **0.737** | **114.021** | 39.176 | **84.046** | 50.445 | 4.363E-02 |
| [230401_at](https://www.affymetrix.com/LinkServlet?probeset=230401_at) | [NA](http://www.ncbi.nlm.nih.gov/entrez/query.fcgi?cmd=search&db=gene&term=NA) | NA | **0.737** | **62.189** | 18.927 | **45.848** | 35.264 | 3.866E-02 |
| [227127_at](https://www.affymetrix.com/LinkServlet?probeset=227127_at) | [TMEM110](http://www.ncbi.nlm.nih.gov/entrez/query.fcgi?cmd=search&db=gene&term=TMEM110) | transmembrane protein 110 | **0.738** | **13.208** | 4.313 | **9.741** | 4.079 | 2.496E-02 |
| [201248_s_at](https://www.affymetrix.com/LinkServlet?probeset=201248_s_at) | [SREBF2](http://www.ncbi.nlm.nih.gov/entrez/query.fcgi?cmd=search&db=gene&term=SREBF2) | sterol regulatory element binding transcription factor 2 | **0.738** | **65.752** | 21.189 | **48.504** | 16.137 | 1.766E-02 |
| [214315_x_at](https://www.affymetrix.com/LinkServlet?probeset=214315_x_at) | [CALR](http://www.ncbi.nlm.nih.gov/entrez/query.fcgi?cmd=search&db=gene&term=CALR) | calreticulin | **0.738** | **814.771** | 201.011 | **601.095** | 75.635 | 3.266E-03 |
| [233122_at](https://www.affymetrix.com/LinkServlet?probeset=233122_at) | [KRTCAP2](http://www.ncbi.nlm.nih.gov/entrez/query.fcgi?cmd=search&db=gene&term=KRTCAP2) | keratinocyte associated protein 2 | **0.738** | **91.595** | 34.250 | **67.604** | 46.777 | 4.916E-02 |
| [210053_at](https://www.affymetrix.com/LinkServlet?probeset=210053_at) | [TAF5](http://www.ncbi.nlm.nih.gov/entrez/query.fcgi?cmd=search&db=gene&term=TAF5) | TAF5 RNA polymerase II, TATA box binding protein (TBP)-associated factor, 100kDa | **0.738** | **584.926** | 114.040 | **431.894** | 144.018 | 7.417E-03 |
| [242337_at](https://www.affymetrix.com/LinkServlet?probeset=242337_at) | [NA](http://www.ncbi.nlm.nih.gov/entrez/query.fcgi?cmd=search&db=gene&term=NA) | NA | **0.739** | **249.970** | 82.675 | **184.789** | 101.318 | 4.442E-02 |
| [201025_at](https://www.affymetrix.com/LinkServlet?probeset=201025_at) | [EIF5B](http://www.ncbi.nlm.nih.gov/entrez/query.fcgi?cmd=search&db=gene&term=EIF5B) | eukaryotic translation initiation factor 5B | **0.740** | **153.074** | 40.446 | **113.241** | 30.754 | 6.132E-03 |
| [218689_at](https://www.affymetrix.com/LinkServlet?probeset=218689_at) | [FANCF](http://www.ncbi.nlm.nih.gov/entrez/query.fcgi?cmd=search&db=gene&term=FANCF) | Fanconi anemia, complementation group F | **0.740** | **469.123** | 82.057 | **347.296** | 84.117 | 1.378E-03 |
| [235203_at](https://www.affymetrix.com/LinkServlet?probeset=235203_at) | [NA](http://www.ncbi.nlm.nih.gov/entrez/query.fcgi?cmd=search&db=gene&term=NA) | NA | **0.740** | **235.228** | 37.305 | **174.174** | 37.133 | 1.183E-03 |
| [226730_s_at](https://www.affymetrix.com/LinkServlet?probeset=226730_s_at) | [USP37](http://www.ncbi.nlm.nih.gov/entrez/query.fcgi?cmd=search&db=gene&term=USP37) | ubiquitin specific peptidase 37 | **0.740** | **291.760** | 79.292 | **216.035** | 54.245 | 1.328E-02 |
| [225428_s_at](https://www.affymetrix.com/LinkServlet?probeset=225428_s_at) | [DDX54](http://www.ncbi.nlm.nih.gov/entrez/query.fcgi?cmd=search&db=gene&term=DDX54) | DEAD (Asp-Glu-Ala-Asp) box polypeptide 54 | **0.741** | **17.804** | 5.145 | **13.186** | 6.649 | 2.953E-02 |
| [202811_at](https://www.affymetrix.com/LinkServlet?probeset=202811_at) | [STAMBP](http://www.ncbi.nlm.nih.gov/entrez/query.fcgi?cmd=search&db=gene&term=STAMBP) | STAM binding protein | **0.741** | **611.886** | 102.341 | **453.311** | 94.413 | 4.027E-04 |
| [217963_s_at](https://www.affymetrix.com/LinkServlet?probeset=217963_s_at) | [NGFRAP1](http://www.ncbi.nlm.nih.gov/entrez/query.fcgi?cmd=search&db=gene&term=NGFRAP1) | nerve growth factor receptor (TNFRSF16) associated protein 1 | **0.741** | **217.583** | 76.242 | **161.263** | 56.068 | 4.180E-02 |
| [238030_at](https://www.affymetrix.com/LinkServlet?probeset=238030_at) | [ZNF268](http://www.ncbi.nlm.nih.gov/entrez/query.fcgi?cmd=search&db=gene&term=ZNF268) | zinc finger protein 268 | **0.741** | **63.513** | 17.986 | **47.074** | 23.732 | 4.469E-02 |
| [202837_at](https://www.affymetrix.com/LinkServlet?probeset=202837_at) | [TRAFD1](http://www.ncbi.nlm.nih.gov/entrez/query.fcgi?cmd=search&db=gene&term=TRAFD1) | TRAF-type zinc finger domain containing 1 | **0.741** | **153.005** | 49.329 | **113.445** | 39.882 | 2.423E-02 |
| [202502_at](https://www.affymetrix.com/LinkServlet?probeset=202502_at) | [ACADM](http://www.ncbi.nlm.nih.gov/entrez/query.fcgi?cmd=search&db=gene&term=ACADM) | acyl-CoA dehydrogenase, C-4 to C-12 straight chain | **0.742** | **173.322** | 53.309 | **128.615** | 40.061 | 1.516E-02 |
| [227335_at](https://www.affymetrix.com/LinkServlet?probeset=227335_at) | [DIDO1](http://www.ncbi.nlm.nih.gov/entrez/query.fcgi?cmd=search&db=gene&term=DIDO1) | death inducer-obliterator 1 | **0.743** | **221.974** | 76.828 | **164.837** | 55.304 | 2.625E-02 |
| [222192_s_at](https://www.affymetrix.com/LinkServlet?probeset=222192_s_at) | [C2orf43](http://www.ncbi.nlm.nih.gov/entrez/query.fcgi?cmd=search&db=gene&term=C2orf43) | chromosome 2 open reading frame 43 | **0.743** | **60.545** | 20.501 | **45.014** | 24.573 | 4.440E-02 |
| [221187_s_at](https://www.affymetrix.com/LinkServlet?probeset=221187_s_at) | [FUZ](http://www.ncbi.nlm.nih.gov/entrez/query.fcgi?cmd=search&db=gene&term=FUZ) | fuzzy homolog (Drosophila) | **0.744** | **24.326** | 5.504 | **18.088** | 6.858 | 9.431E-03 |
| [231834_at](https://www.affymetrix.com/LinkServlet?probeset=231834_at) | [RBM33](http://www.ncbi.nlm.nih.gov/entrez/query.fcgi?cmd=search&db=gene&term=RBM33) | RNA binding motif protein 33 | **0.744** | **8.881** | 3.970 | **6.610** | 1.049 | 4.692E-02 |
| [238076_at](https://www.affymetrix.com/LinkServlet?probeset=238076_at) | [GATAD2B](http://www.ncbi.nlm.nih.gov/entrez/query.fcgi?cmd=search&db=gene&term=GATAD2B) | GATA zinc finger domain containing 2B | **0.744** | **166.791** | 51.447 | **124.164** | 72.490 | 2.971E-02 |
| [204496_at](https://www.affymetrix.com/LinkServlet?probeset=204496_at) | [STRN3](http://www.ncbi.nlm.nih.gov/entrez/query.fcgi?cmd=search&db=gene&term=STRN3) | striatin, calmodulin binding protein 3 | **0.745** | **102.118** | 35.479 | **76.107** | 24.407 | 3.658E-02 |
| [225939_at](https://www.affymetrix.com/LinkServlet?probeset=225939_at) | [EIF4E3](http://www.ncbi.nlm.nih.gov/entrez/query.fcgi?cmd=search&db=gene&term=EIF4E3) | eukaryotic translation initiation factor 4E family member 3 | **0.746** | **356.507** | 106.509 | **265.776** | 95.630 | 1.820E-02 |
| [217183_at](https://www.affymetrix.com/LinkServlet?probeset=217183_at) | [LDLR](http://www.ncbi.nlm.nih.gov/entrez/query.fcgi?cmd=search&db=gene&term=LDLR) | low density lipoprotein receptor | **0.746** | **15.341** | 5.657 | **11.445** | 5.510 | 4.998E-02 |
| [217344_at](https://www.affymetrix.com/LinkServlet?probeset=217344_at) | [FDPS](http://www.ncbi.nlm.nih.gov/entrez/query.fcgi?cmd=search&db=gene&term=FDPS) | farnesyl diphosphate synthase | **0.747** | **26.153** | 7.954 | **19.528** | 6.092 | 3.359E-02 |
| [223078_s_at](https://www.affymetrix.com/LinkServlet?probeset=223078_s_at) | [TMOD3](http://www.ncbi.nlm.nih.gov/entrez/query.fcgi?cmd=search&db=gene&term=TMOD3) | tropomodulin 3 (ubiquitous) | **0.747** | **29.306** | 10.374 | **21.882** | 18.128 | 2.733E-02 |
| [213689_x_at](https://www.affymetrix.com/LinkServlet?probeset=213689_x_at) | [FAM69A](http://www.ncbi.nlm.nih.gov/entrez/query.fcgi?cmd=search&db=gene&term=FAM69A) | family with sequence similarity 69, member A | **0.747** | **334.980** | 121.712 | **250.175** | 74.004 | 2.367E-02 |
| [225195_at](https://www.affymetrix.com/LinkServlet?probeset=225195_at) | [DPH3](http://www.ncbi.nlm.nih.gov/entrez/query.fcgi?cmd=search&db=gene&term=DPH3) | DPH3, KTI11 homolog (S. cerevisiae) | **0.747** | **186.059** | 59.567 | **138.993** | 45.775 | 2.782E-02 |
| [205555_s_at](https://www.affymetrix.com/LinkServlet?probeset=205555_s_at) | [MSX2](http://www.ncbi.nlm.nih.gov/entrez/query.fcgi?cmd=search&db=gene&term=MSX2) | msh homeobox 2 | **0.747** | **9.511** | 2.838 | **7.106** | 1.560 | 1.049E-02 |
| [219397_at](https://www.affymetrix.com/LinkServlet?probeset=219397_at) | [COQ10B](http://www.ncbi.nlm.nih.gov/entrez/query.fcgi?cmd=search&db=gene&term=COQ10B) | coenzyme Q10 homolog B (S. cerevisiae) | **0.747** | **472.393** | 73.166 | **352.939** | 93.906 | 8.566E-04 |
| [214193_s_at](https://www.affymetrix.com/LinkServlet?probeset=214193_s_at) | [DIEXF](http://www.ncbi.nlm.nih.gov/entrez/query.fcgi?cmd=search&db=gene&term=DIEXF) | digestive organ expansion factor homolog (zebrafish) | **0.748** | **95.244** | 20.735 | **71.220** | 26.314 | 1.124E-02 |
| [227014_at](https://www.affymetrix.com/LinkServlet?probeset=227014_at) | [ASPHD2](http://www.ncbi.nlm.nih.gov/entrez/query.fcgi?cmd=search&db=gene&term=ASPHD2) | aspartate beta-hydroxylase domain containing 2 | **0.749** | **152.642** | 46.439 | **114.277** | 42.088 | 3.141E-02 |
| [227508_at](https://www.affymetrix.com/LinkServlet?probeset=227508_at) | [NA](http://www.ncbi.nlm.nih.gov/entrez/query.fcgi?cmd=search&db=gene&term=NA) | NA | **0.749** | **40.731** | 10.679 | **30.502** | 14.225 | 3.179E-02 |
| [222440_s_at](https://www.affymetrix.com/LinkServlet?probeset=222440_s_at) | [THRAP3](http://www.ncbi.nlm.nih.gov/entrez/query.fcgi?cmd=search&db=gene&term=THRAP3) | thyroid hormone receptor associated protein 3 | **0.750** | **600.670** | 103.860 | **450.234** | 122.596 | 2.479E-03 |
| [203112_s_at](https://www.affymetrix.com/LinkServlet?probeset=203112_s_at) | [WHSC2](http://www.ncbi.nlm.nih.gov/entrez/query.fcgi?cmd=search&db=gene&term=WHSC2) | Wolf-Hirschhorn syndrome candidate 2 | **0.750** | **123.729** | 50.171 | **92.752** | 37.027 | 3.598E-02 |
| [1558233_s_at](https://www.affymetrix.com/LinkServlet?probeset=1558233_s_at) | [ATF1](http://www.ncbi.nlm.nih.gov/entrez/query.fcgi?cmd=search&db=gene&term=ATF1) | activating transcription factor 1 | **0.750** | **355.393** | 100.660 | **266.543** | 105.576 | 2.047E-02 |
| [212881_at](https://www.affymetrix.com/LinkServlet?probeset=212881_at) | [PIAS4](http://www.ncbi.nlm.nih.gov/entrez/query.fcgi?cmd=search&db=gene&term=PIAS4) | protein inhibitor of activated STAT, 4 | **0.750** | **97.693** | 29.572 | **73.271** | 25.474 | 3.755E-02 |
| [205407_at](https://www.affymetrix.com/LinkServlet?probeset=205407_at) | [RECK](http://www.ncbi.nlm.nih.gov/entrez/query.fcgi?cmd=search&db=gene&term=RECK) | reversion-inducing-cysteine-rich protein with kazal motifs | **0.750** | **171.579** | 60.483 | **128.729** | 29.461 | 2.056E-02 |
| [203094_at](https://www.affymetrix.com/LinkServlet?probeset=203094_at) | [MAD2L1BP](http://www.ncbi.nlm.nih.gov/entrez/query.fcgi?cmd=search&db=gene&term=MAD2L1BP) | MAD2L1 binding protein | **0.751** | **147.885** | 45.433 | **111.007** | 32.596 | 2.758E-02 |
| [225388_at](https://www.affymetrix.com/LinkServlet?probeset=225388_at) | [TSPAN5](http://www.ncbi.nlm.nih.gov/entrez/query.fcgi?cmd=search&db=gene&term=TSPAN5) | tetraspanin 5 | **0.751** | **36.246** | 11.128 | **27.208** | 8.868 | 4.902E-02 |
| [222763_s_at](https://www.affymetrix.com/LinkServlet?probeset=222763_s_at) | [WDR33](http://www.ncbi.nlm.nih.gov/entrez/query.fcgi?cmd=search&db=gene&term=WDR33) | WD repeat domain 33 | **0.751** | **360.965** | 61.648 | **271.062** | 61.601 | 1.164E-03 |
| [242887_at](https://www.affymetrix.com/LinkServlet?probeset=242887_at) | [KCMF1](http://www.ncbi.nlm.nih.gov/entrez/query.fcgi?cmd=search&db=gene&term=KCMF1) | potassium channel modulatory factor 1 | **0.751** | **283.919** | 83.428 | **213.311** | 66.111 | 1.253E-02 |
| [223989_s_at](https://www.affymetrix.com/LinkServlet?probeset=223989_s_at) | [REXO2](http://www.ncbi.nlm.nih.gov/entrez/query.fcgi?cmd=search&db=gene&term=REXO2) | REX2, RNA exonuclease 2 homolog (S. cerevisiae) | **0.752** | **93.908** | 28.630 | **70.625** | 31.305 | 4.330E-02 |
| [218514_at](https://www.affymetrix.com/LinkServlet?probeset=218514_at) | [C17orf71](http://www.ncbi.nlm.nih.gov/entrez/query.fcgi?cmd=search&db=gene&term=C17orf71) | chromosome 17 open reading frame 71 | **0.752** | **158.298** | 62.053 | **119.076** | 28.608 | 2.649E-02 |
| [205333_s_at](https://www.affymetrix.com/LinkServlet?probeset=205333_s_at) | [RCE1](http://www.ncbi.nlm.nih.gov/entrez/query.fcgi?cmd=search&db=gene&term=RCE1) | RCE1 homolog, prenyl protein peptidase (S. cerevisiae) | **0.753** | **74.145** | 25.749 | **55.800** | 20.150 | 3.244E-02 |
| [216323_x_at](https://www.affymetrix.com/LinkServlet?probeset=216323_x_at) | [TUBA3D](http://www.ncbi.nlm.nih.gov/entrez/query.fcgi?cmd=search&db=gene&term=TUBA3D) | tubulin, alpha 3d | **0.753** | **65.783** | 16.465 | **49.532** | 18.375 | 1.348E-02 |
| [230197_s_at](https://www.affymetrix.com/LinkServlet?probeset=230197_s_at) | [TPPP](http://www.ncbi.nlm.nih.gov/entrez/query.fcgi?cmd=search&db=gene&term=TPPP) | tubulin polymerization promoting protein | **0.753** | **9.541** | 3.671 | **7.186** | 2.002 | 3.280E-02 |
| [204642_at](https://www.affymetrix.com/LinkServlet?probeset=204642_at) | [S1PR1](http://www.ncbi.nlm.nih.gov/entrez/query.fcgi?cmd=search&db=gene&term=S1PR1) | sphingosine-1-phosphate receptor 1 | **0.754** | **578.928** | 153.382 | **436.737** | 126.659 | 1.504E-02 |
| [235585_at](https://www.affymetrix.com/LinkServlet?probeset=235585_at) | [NA](http://www.ncbi.nlm.nih.gov/entrez/query.fcgi?cmd=search&db=gene&term=NA) | NA | **0.754** | **206.138** | 47.161 | **155.529** | 36.660 | 1.009E-02 |
| [226749_at](https://www.affymetrix.com/LinkServlet?probeset=226749_at) | [MRPS9](http://www.ncbi.nlm.nih.gov/entrez/query.fcgi?cmd=search&db=gene&term=MRPS9) | mitochondrial ribosomal protein S9 | **0.755** | **269.961** | 86.380 | **203.929** | 60.289 | 3.682E-02 |
| [204484_at](https://www.affymetrix.com/LinkServlet?probeset=204484_at) | [PIK3C2B](http://www.ncbi.nlm.nih.gov/entrez/query.fcgi?cmd=search&db=gene&term=PIK3C2B) | phosphoinositide-3-kinase, class 2, beta polypeptide | **0.757** | **375.722** | 108.675 | **284.244** | 108.478 | 4.295E-02 |
| [219171_s_at](https://www.affymetrix.com/LinkServlet?probeset=219171_s_at) | [ZNF236](http://www.ncbi.nlm.nih.gov/entrez/query.fcgi?cmd=search&db=gene&term=ZNF236) | zinc finger protein 236 | **0.757** | **42.271** | 11.541 | **31.996** | 23.553 | 3.329E-02 |
| [202383_at](https://www.affymetrix.com/LinkServlet?probeset=202383_at) | [KDM5C](http://www.ncbi.nlm.nih.gov/entrez/query.fcgi?cmd=search&db=gene&term=KDM5C) | lysine (K)-specific demethylase 5C | **0.757** | **170.489** | 41.884 | **129.054** | 31.336 | 1.306E-02 |
| [203156_at](https://www.affymetrix.com/LinkServlet?probeset=203156_at) | [AKAP11](http://www.ncbi.nlm.nih.gov/entrez/query.fcgi?cmd=search&db=gene&term=AKAP11) | A kinase (PRKA) anchor protein 11 | **0.757** | **248.902** | 79.414 | **188.426** | 37.707 | 1.299E-02 |
| [205412_at](https://www.affymetrix.com/LinkServlet?probeset=205412_at) | [ACAT1](http://www.ncbi.nlm.nih.gov/entrez/query.fcgi?cmd=search&db=gene&term=ACAT1) | acetyl-CoA acetyltransferase 1 | **0.758** | **341.296** | 112.891 | **258.537** | 60.013 | 2.625E-02 |
| [1552789_at](https://www.affymetrix.com/LinkServlet?probeset=1552789_at) | [SEC62](http://www.ncbi.nlm.nih.gov/entrez/query.fcgi?cmd=search&db=gene&term=SEC62) | SEC62 homolog (S. cerevisiae) | **0.758** | **225.656** | 37.771 | **170.973** | 64.687 | 1.196E-02 |
| [221918_at](https://www.affymetrix.com/LinkServlet?probeset=221918_at) | [CDK17](http://www.ncbi.nlm.nih.gov/entrez/query.fcgi?cmd=search&db=gene&term=CDK17) | cyclin-dependent kinase 17 | **0.758** | **401.493** | 81.079 | **304.283** | 50.051 | 8.597E-04 |
| [209247_s_at](https://www.affymetrix.com/LinkServlet?probeset=209247_s_at) | [ABCF2](http://www.ncbi.nlm.nih.gov/entrez/query.fcgi?cmd=search&db=gene&term=ABCF2) | ATP-binding cassette, sub-family F (GCN20), member 2 | **0.759** | **56.886** | 16.212 | **43.189** | 19.825 | 3.923E-02 |
| [242300_at](https://www.affymetrix.com/LinkServlet?probeset=242300_at) | [NA](http://www.ncbi.nlm.nih.gov/entrez/query.fcgi?cmd=search&db=gene&term=NA) | NA | **0.759** | **90.507** | 29.770 | **68.715** | 26.881 | 4.760E-02 |
| [238719_at](https://www.affymetrix.com/LinkServlet?probeset=238719_at) | [PPP2CA](http://www.ncbi.nlm.nih.gov/entrez/query.fcgi?cmd=search&db=gene&term=PPP2CA) | protein phosphatase 2, catalytic subunit, alpha isozyme | **0.759** | **253.086** | 40.217 | **192.160** | 62.250 | 5.262E-03 |
| [224927_at](https://www.affymetrix.com/LinkServlet?probeset=224927_at) | [KIAA1949](http://www.ncbi.nlm.nih.gov/entrez/query.fcgi?cmd=search&db=gene&term=KIAA1949) | KIAA1949 | **0.760** | **407.477** | 129.824 | **309.551** | 86.113 | 3.866E-02 |
| [1561500_at](https://www.affymetrix.com/LinkServlet?probeset=1561500_at) | [CTU2](http://www.ncbi.nlm.nih.gov/entrez/query.fcgi?cmd=search&db=gene&term=CTU2) | cytosolic thiouridylase subunit 2 homolog (S. pombe) | **0.760** | **16.198** | 4.580 | **12.307** | 5.248 | 2.192E-02 |
| [226747_at](https://www.affymetrix.com/LinkServlet?probeset=226747_at) | [TXNDC16](http://www.ncbi.nlm.nih.gov/entrez/query.fcgi?cmd=search&db=gene&term=TXNDC16) | thioredoxin domain containing 16 | **0.760** | **162.373** | 45.104 | **123.412** | 26.025 | 2.309E-02 |
| [205263_at](https://www.affymetrix.com/LinkServlet?probeset=205263_at) | [BCL10](http://www.ncbi.nlm.nih.gov/entrez/query.fcgi?cmd=search&db=gene&term=BCL10) | B-cell CLL/lymphoma 10 | **0.760** | **401.979** | 122.099 | **305.584** | 97.056 | 3.470E-02 |
| [213341_at](https://www.affymetrix.com/LinkServlet?probeset=213341_at) | [FEM1C](http://www.ncbi.nlm.nih.gov/entrez/query.fcgi?cmd=search&db=gene&term=FEM1C) | fem-1 homolog c (C. elegans) | **0.760** | **154.452** | 43.773 | **117.450** | 35.730 | 4.257E-02 |
| [219806_s_at](https://www.affymetrix.com/LinkServlet?probeset=219806_s_at) | [C11orf75](http://www.ncbi.nlm.nih.gov/entrez/query.fcgi?cmd=search&db=gene&term=C11orf75) | chromosome 11 open reading frame 75 | **0.762** | **65.805** | 19.526 | **50.149** | 16.130 | 4.911E-02 |
| [1555281_x_at](https://www.affymetrix.com/LinkServlet?probeset=1555281_x_at) | [ARMC8](http://www.ncbi.nlm.nih.gov/entrez/query.fcgi?cmd=search&db=gene&term=ARMC8) | armadillo repeat containing 8 | **0.762** | **137.896** | 38.384 | **105.102** | 29.982 | 1.291E-02 |
| [200938_s_at](https://www.affymetrix.com/LinkServlet?probeset=200938_s_at) | [RERE](http://www.ncbi.nlm.nih.gov/entrez/query.fcgi?cmd=search&db=gene&term=RERE) | arginine-glutamic acid dipeptide (RE) repeats | **0.762** | **8.519** | 2.486 | **6.494** | 0.587 | 9.883E-03 |
| [201496_x_at](https://www.affymetrix.com/LinkServlet?probeset=201496_x_at) | [MYH11](http://www.ncbi.nlm.nih.gov/entrez/query.fcgi?cmd=search&db=gene&term=MYH11) | myosin, heavy chain 11, smooth muscle | **0.763** | **21.100** | 6.078 | **16.090** | 5.152 | 4.529E-02 |
| [226181_at](https://www.affymetrix.com/LinkServlet?probeset=226181_at) | [TUBE1](http://www.ncbi.nlm.nih.gov/entrez/query.fcgi?cmd=search&db=gene&term=TUBE1) | tubulin, epsilon 1 | **0.763** | **104.097** | 24.107 | **79.407** | 44.884 | 2.424E-02 |
| [244663_at](https://www.affymetrix.com/LinkServlet?probeset=244663_at) | [NA](http://www.ncbi.nlm.nih.gov/entrez/query.fcgi?cmd=search&db=gene&term=NA) | NA | **0.763** | **219.814** | 53.451 | **167.774** | 68.195 | 2.181E-02 |
| [213025_at](https://www.affymetrix.com/LinkServlet?probeset=213025_at) | [THUMPD1](http://www.ncbi.nlm.nih.gov/entrez/query.fcgi?cmd=search&db=gene&term=THUMPD1) | THUMP domain containing 1 | **0.763** | **462.971** | 152.932 | **353.370** | 40.958 | 1.416E-02 |
| [214835_s_at](https://www.affymetrix.com/LinkServlet?probeset=214835_s_at) | [SUCLG2](http://www.ncbi.nlm.nih.gov/entrez/query.fcgi?cmd=search&db=gene&term=SUCLG2) | succinate-CoA ligase, GDP-forming, beta subunit | **0.763** | **206.541** | 64.064 | **157.664** | 60.849 | 3.572E-02 |
| [1561501_s_at](https://www.affymetrix.com/LinkServlet?probeset=1561501_s_at) | [CTU2](http://www.ncbi.nlm.nih.gov/entrez/query.fcgi?cmd=search&db=gene&term=CTU2) | cytosolic thiouridylase subunit 2 homolog (S. pombe) | **0.764** | **8.157** | 2.892 | **6.229** | 0.520 | 3.225E-02 |
| [216060_s_at](https://www.affymetrix.com/LinkServlet?probeset=216060_s_at) | [DAAM1](http://www.ncbi.nlm.nih.gov/entrez/query.fcgi?cmd=search&db=gene&term=DAAM1) | dishevelled associated activator of morphogenesis 1 | **0.764** | **184.803** | 40.007 | **141.178** | 44.546 | 1.180E-02 |
| [205739_x_at](https://www.affymetrix.com/LinkServlet?probeset=205739_x_at) | [ZNF107](http://www.ncbi.nlm.nih.gov/entrez/query.fcgi?cmd=search&db=gene&term=ZNF107) | zinc finger protein 107 | **0.765** | **250.023** | 53.758 | **191.156** | 99.134 | 1.781E-02 |
| [228567_at](https://www.affymetrix.com/LinkServlet?probeset=228567_at) | [NA](http://www.ncbi.nlm.nih.gov/entrez/query.fcgi?cmd=search&db=gene&term=NA) | NA | **0.765** | **178.541** | 50.965 | **136.552** | 40.035 | 3.148E-02 |
| [227839_at](https://www.affymetrix.com/LinkServlet?probeset=227839_at) | [MBD5](http://www.ncbi.nlm.nih.gov/entrez/query.fcgi?cmd=search&db=gene&term=MBD5) | methyl-CpG binding domain protein 5 | **0.765** | **71.150** | 24.904 | **54.421** | 36.602 | 4.881E-02 |
| [224705_s_at](https://www.affymetrix.com/LinkServlet?probeset=224705_s_at) | [TNRC6A](http://www.ncbi.nlm.nih.gov/entrez/query.fcgi?cmd=search&db=gene&term=TNRC6A) | trinucleotide repeat containing 6A | **0.765** | **309.046** | 53.688 | **236.433** | 96.913 | 2.215E-02 |
| [204700_x_at](https://www.affymetrix.com/LinkServlet?probeset=204700_x_at) | [DIEXF](http://www.ncbi.nlm.nih.gov/entrez/query.fcgi?cmd=search&db=gene&term=DIEXF) | digestive organ expansion factor homolog (zebrafish) | **0.765** | **141.820** | 35.946 | **108.529** | 32.626 | 1.713E-02 |
| [204576_s_at](https://www.affymetrix.com/LinkServlet?probeset=204576_s_at) | [CLUAP1](http://www.ncbi.nlm.nih.gov/entrez/query.fcgi?cmd=search&db=gene&term=CLUAP1) | clusterin associated protein 1 | **0.765** | **130.860** | 32.410 | **100.149** | 45.853 | 3.693E-02 |
| [220097_s_at](https://www.affymetrix.com/LinkServlet?probeset=220097_s_at) | [TMEM104](http://www.ncbi.nlm.nih.gov/entrez/query.fcgi?cmd=search&db=gene&term=TMEM104) | transmembrane protein 104 | **0.765** | **79.712** | 19.356 | **61.010** | 21.005 | 1.359E-02 |
| [222499_at](https://www.affymetrix.com/LinkServlet?probeset=222499_at) | [MRPS16](http://www.ncbi.nlm.nih.gov/entrez/query.fcgi?cmd=search&db=gene&term=MRPS16) | mitochondrial ribosomal protein S16 | **0.766** | **90.377** | 20.417 | **69.204** | 24.059 | 9.183E-03 |
| [205763_s_at](https://www.affymetrix.com/LinkServlet?probeset=205763_s_at) | [DDX18](http://www.ncbi.nlm.nih.gov/entrez/query.fcgi?cmd=search&db=gene&term=DDX18) | DEAD (Asp-Glu-Ala-Asp) box polypeptide 18 | **0.766** | **862.104** | 128.769 | **660.157** | 128.180 | 5.188E-04 |
| [203057_s_at](https://www.affymetrix.com/LinkServlet?probeset=203057_s_at) | [PRDM2](http://www.ncbi.nlm.nih.gov/entrez/query.fcgi?cmd=search&db=gene&term=PRDM2) | PR domain containing 2, with ZNF domain | **0.766** | **552.660** | 140.834 | **423.465** | 105.689 | 7.471E-03 |
| [222979_s_at](https://www.affymetrix.com/LinkServlet?probeset=222979_s_at) | [SURF4](http://www.ncbi.nlm.nih.gov/entrez/query.fcgi?cmd=search&db=gene&term=SURF4) | surfeit 4 | **0.766** | **643.789** | 162.514 | **493.387** | 209.481 | 3.109E-02 |
| [35254_at](https://www.affymetrix.com/LinkServlet?probeset=35254_at) | [TRAFD1](http://www.ncbi.nlm.nih.gov/entrez/query.fcgi?cmd=search&db=gene&term=TRAFD1) | TRAF-type zinc finger domain containing 1 | **0.766** | **407.589** | 92.957 | **312.417** | 60.238 | 8.040E-03 |
| [209423_s_at](https://www.affymetrix.com/LinkServlet?probeset=209423_s_at) | [PHF20](http://www.ncbi.nlm.nih.gov/entrez/query.fcgi?cmd=search&db=gene&term=PHF20) | PHD finger protein 20 | **0.767** | **7.825** | 2.555 | **6.000** | **0.000** | 1.349E-02 |
| [217803_at](https://www.affymetrix.com/LinkServlet?probeset=217803_at) | [GOLPH3](http://www.ncbi.nlm.nih.gov/entrez/query.fcgi?cmd=search&db=gene&term=GOLPH3) | golgi phosphoprotein 3 (coat-protein) | **0.767** | **451.683** | 90.266 | **346.411** | 80.419 | 5.452E-03 |
| [242740_at](https://www.affymetrix.com/LinkServlet?probeset=242740_at) | [NA](http://www.ncbi.nlm.nih.gov/entrez/query.fcgi?cmd=search&db=gene&term=NA) | NA | **0.767** | **62.489** | 28.399 | **47.929** | 44.895 | 4.611E-02 |
| [222566_at](https://www.affymetrix.com/LinkServlet?probeset=222566_at) | [SUV420H1](http://www.ncbi.nlm.nih.gov/entrez/query.fcgi?cmd=search&db=gene&term=SUV420H1) | suppressor of variegation 4-20 homolog 1 (Drosophila) | **0.768** | **233.156** | 41.679 | **178.979** | 35.117 | 1.779E-03 |
| [201622_at](https://www.affymetrix.com/LinkServlet?probeset=201622_at) | [SND1](http://www.ncbi.nlm.nih.gov/entrez/query.fcgi?cmd=search&db=gene&term=SND1) | staphylococcal nuclease and tudor domain containing 1 | **0.768** | **284.937** | 93.899 | **218.741** | 47.521 | 3.982E-02 |
| [221597_s_at](https://www.affymetrix.com/LinkServlet?probeset=221597_s_at) | [TMEM208](http://www.ncbi.nlm.nih.gov/entrez/query.fcgi?cmd=search&db=gene&term=TMEM208) | transmembrane protein 208 | **0.768** | **229.417** | 73.087 | **176.224** | 48.759 | 3.957E-02 |
| [210047_at](https://www.affymetrix.com/LinkServlet?probeset=210047_at) | [SLC11A2](http://www.ncbi.nlm.nih.gov/entrez/query.fcgi?cmd=search&db=gene&term=SLC11A2) | solute carrier family 11 (proton-coupled divalent metal ion transporters), member 2 | **0.768** | **255.168** | 80.359 | **196.047** | 125.284 | 4.176E-02 |
| [220147_s_at](https://www.affymetrix.com/LinkServlet?probeset=220147_s_at) | [FAM60A](http://www.ncbi.nlm.nih.gov/entrez/query.fcgi?cmd=search&db=gene&term=FAM60A) | family with sequence similarity 60, member A | **0.768** | **682.400** | 120.081 | **524.370** | 133.657 | 4.300E-03 |
| [226158_at](https://www.affymetrix.com/LinkServlet?probeset=226158_at) | [KLHL24](http://www.ncbi.nlm.nih.gov/entrez/query.fcgi?cmd=search&db=gene&term=KLHL24) | kelch-like 24 (Drosophila) | **0.769** | **361.155** | 95.265 | **277.687** | 64.213 | 1.620E-02 |
| [1552790_a_at](https://www.affymetrix.com/LinkServlet?probeset=1552790_a_at) | [SEC62](http://www.ncbi.nlm.nih.gov/entrez/query.fcgi?cmd=search&db=gene&term=SEC62) | SEC62 homolog (S. cerevisiae) | **0.769** | **252.264** | 61.978 | **194.060** | 78.733 | 4.558E-02 |
| [212341_at](https://www.affymetrix.com/LinkServlet?probeset=212341_at) | [YIPF6](http://www.ncbi.nlm.nih.gov/entrez/query.fcgi?cmd=search&db=gene&term=YIPF6) | Yip1 domain family, member 6 | **0.769** | **89.103** | 23.621 | **68.562** | 28.949 | 2.775E-02 |
| [213561_at](https://www.affymetrix.com/LinkServlet?probeset=213561_at) | [ASF1A](http://www.ncbi.nlm.nih.gov/entrez/query.fcgi?cmd=search&db=gene&term=ASF1A) | ASF1 anti-silencing function 1 homolog A (S. cerevisiae) | **0.770** | **359.469** | 76.418 | **276.616** | 47.403 | 2.456E-03 |
| [233952_s_at](https://www.affymetrix.com/LinkServlet?probeset=233952_s_at) | [ZNF295](http://www.ncbi.nlm.nih.gov/entrez/query.fcgi?cmd=search&db=gene&term=ZNF295) | zinc finger protein 295 | **0.770** | **131.850** | 26.435 | **101.550** | 53.812 | 2.813E-02 |
| [222422_s_at](https://www.affymetrix.com/LinkServlet?probeset=222422_s_at) | [NDFIP1](http://www.ncbi.nlm.nih.gov/entrez/query.fcgi?cmd=search&db=gene&term=NDFIP1) | Nedd4 family interacting protein 1 | **0.771** | **361.041** | 127.058 | **278.237** | 77.379 | 4.639E-02 |
| [1559343_at](https://www.affymetrix.com/LinkServlet?probeset=1559343_at) | [SNRPN](http://www.ncbi.nlm.nih.gov/entrez/query.fcgi?cmd=search&db=gene&term=SNRPN) | small nuclear ribonucleoprotein polypeptide N | **0.771** | **389.579** | 78.906 | **300.428** | 96.455 | 8.078E-03 |
| [201036_s_at](https://www.affymetrix.com/LinkServlet?probeset=201036_s_at) | [HADH](http://www.ncbi.nlm.nih.gov/entrez/query.fcgi?cmd=search&db=gene&term=HADH) | hydroxyacyl-CoA dehydrogenase | **0.771** | **395.039** | 118.149 | **304.676** | 72.768 | 2.154E-02 |
| [229766_at](https://www.affymetrix.com/LinkServlet?probeset=229766_at) | [ZNF445](http://www.ncbi.nlm.nih.gov/entrez/query.fcgi?cmd=search&db=gene&term=ZNF445) | zinc finger protein 445 | **0.771** | **140.082** | 52.885 | **108.067** | 37.276 | 4.810E-02 |
| [237753_at](https://www.affymetrix.com/LinkServlet?probeset=237753_at) | [IL21R](http://www.ncbi.nlm.nih.gov/entrez/query.fcgi?cmd=search&db=gene&term=IL21R) | interleukin 21 receptor | **0.772** | **355.878** | 93.915 | **274.807** | 87.880 | 3.288E-02 |
| [207490_at](https://www.affymetrix.com/LinkServlet?probeset=207490_at) | [TUBA4B](http://www.ncbi.nlm.nih.gov/entrez/query.fcgi?cmd=search&db=gene&term=TUBA4B) | tubulin, alpha 4b (pseudogene) | **0.773** | **16.591** | 4.997 | **12.830** | 5.663 | 4.567E-02 |
| [202716_at](https://www.affymetrix.com/LinkServlet?probeset=202716_at) | [PTPN1](http://www.ncbi.nlm.nih.gov/entrez/query.fcgi?cmd=search&db=gene&term=PTPN1) | protein tyrosine phosphatase, non-receptor type 1 | **0.773** | **219.046** | 67.113 | **169.417** | 57.422 | 2.007E-02 |
| [232283_at](https://www.affymetrix.com/LinkServlet?probeset=232283_at) | [LYSMD1](http://www.ncbi.nlm.nih.gov/entrez/query.fcgi?cmd=search&db=gene&term=LYSMD1) | LysM, putative peptidoglycan-binding, domain containing 1 | **0.774** | **147.906** | 39.402 | **114.422** | 57.453 | 4.348E-02 |
| [230403_at](https://www.affymetrix.com/LinkServlet?probeset=230403_at) | [NA](http://www.ncbi.nlm.nih.gov/entrez/query.fcgi?cmd=search&db=gene&term=NA) | NA | **0.774** | **197.498** | 48.209 | **152.810** | 47.604 | 2.246E-02 |
| [228189_at](https://www.affymetrix.com/LinkServlet?probeset=228189_at) | [BAG4](http://www.ncbi.nlm.nih.gov/entrez/query.fcgi?cmd=search&db=gene&term=BAG4) | BCL2-associated athanogene 4 | **0.774** | **207.265** | 46.584 | **160.438** | 37.014 | 2.180E-02 |
| [230230_at](https://www.affymetrix.com/LinkServlet?probeset=230230_at) | [PTPN4](http://www.ncbi.nlm.nih.gov/entrez/query.fcgi?cmd=search&db=gene&term=PTPN4) | protein tyrosine phosphatase, non-receptor type 4 (megakaryocyte) | **0.775** | **218.069** | 66.185 | **168.947** | 81.185 | 4.769E-02 |
| [205397_x_at](https://www.affymetrix.com/LinkServlet?probeset=205397_x_at) | [SMAD3](http://www.ncbi.nlm.nih.gov/entrez/query.fcgi?cmd=search&db=gene&term=SMAD3) | SMAD family member 3 | **0.775** | **17.500** | 5.227 | **13.563** | 11.629 | 2.176E-02 |
| [224699_s_at](https://www.affymetrix.com/LinkServlet?probeset=224699_s_at) | [ESYT2](http://www.ncbi.nlm.nih.gov/entrez/query.fcgi?cmd=search&db=gene&term=ESYT2) | extended synaptotagmin-like protein 2 | **0.776** | **440.889** | 115.065 | **342.202** | 63.511 | 8.493E-03 |
| [218470_at](https://www.affymetrix.com/LinkServlet?probeset=218470_at) | [YARS2](http://www.ncbi.nlm.nih.gov/entrez/query.fcgi?cmd=search&db=gene&term=YARS2) | tyrosyl-tRNA synthetase 2, mitochondrial | **0.776** | **170.589** | 31.638 | **132.407** | 49.551 | 2.087E-02 |
| [226321_at](https://www.affymetrix.com/LinkServlet?probeset=226321_at) | [LYSMD3](http://www.ncbi.nlm.nih.gov/entrez/query.fcgi?cmd=search&db=gene&term=LYSMD3) | LysM, putative peptidoglycan-binding, domain containing 3 | **0.777** | **321.489** | 78.497 | **249.738** | 55.098 | 7.475E-03 |
| [244704_at](https://www.affymetrix.com/LinkServlet?probeset=244704_at) | [NFYB](http://www.ncbi.nlm.nih.gov/entrez/query.fcgi?cmd=search&db=gene&term=NFYB) | nuclear transcription factor Y, beta | **0.777** | **37.021** | 8.830 | **28.761** | 11.042 | 4.577E-02 |
| [214144_at](https://www.affymetrix.com/LinkServlet?probeset=214144_at) | [POLR2D](http://www.ncbi.nlm.nih.gov/entrez/query.fcgi?cmd=search&db=gene&term=POLR2D) | polymerase (RNA) II (DNA directed) polypeptide D | **0.777** | **229.996** | 65.093 | **178.696** | 84.409 | 4.435E-02 |
| [211348_s_at](https://www.affymetrix.com/LinkServlet?probeset=211348_s_at) | [CDC14B](http://www.ncbi.nlm.nih.gov/entrez/query.fcgi?cmd=search&db=gene&term=CDC14B) | CDC14 cell division cycle 14 homolog B (S. cerevisiae) | **0.777** | **179.337** | 49.016 | **139.410** | 24.326 | 1.890E-02 |
| [229835_s_at](https://www.affymetrix.com/LinkServlet?probeset=229835_s_at) | [SLMO2](http://www.ncbi.nlm.nih.gov/entrez/query.fcgi?cmd=search&db=gene&term=SLMO2) | slowmo homolog 2 (Drosophila) | **0.778** | **496.140** | 129.067 | **385.765** | 45.584 | 8.267E-03 |
| [1555279_at](https://www.affymetrix.com/LinkServlet?probeset=1555279_at) | [ARMC8](http://www.ncbi.nlm.nih.gov/entrez/query.fcgi?cmd=search&db=gene&term=ARMC8) | armadillo repeat containing 8 | **0.778** | **196.561** | 43.358 | **152.966** | 58.404 | 2.830E-02 |
| [219097_x_at](https://www.affymetrix.com/LinkServlet?probeset=219097_x_at) | [C19orf42](http://www.ncbi.nlm.nih.gov/entrez/query.fcgi?cmd=search&db=gene&term=C19orf42) | chromosome 19 open reading frame 42 | **0.778** | **492.789** | 110.050 | **383.529** | 58.277 | 3.073E-03 |
| [224416_s_at](https://www.affymetrix.com/LinkServlet?probeset=224416_s_at) | [MED28](http://www.ncbi.nlm.nih.gov/entrez/query.fcgi?cmd=search&db=gene&term=MED28) | mediator complex subunit 28 | **0.778** | **400.366** | 73.046 | **311.629** | 65.336 | 3.245E-03 |
| [1554397_s_at](https://www.affymetrix.com/LinkServlet?probeset=1554397_s_at) | [UEVLD](http://www.ncbi.nlm.nih.gov/entrez/query.fcgi?cmd=search&db=gene&term=UEVLD) | UEV and lactate/malate dehyrogenase domains | **0.779** | **7.805** | 2.436 | **6.082** | 0.272 | 2.179E-02 |
| [235300_x_at](https://www.affymetrix.com/LinkServlet?probeset=235300_x_at) | [RCHY1](http://www.ncbi.nlm.nih.gov/entrez/query.fcgi?cmd=search&db=gene&term=RCHY1) | ring finger and CHY zinc finger domain containing 1 | **0.780** | **41.357** | 11.988 | **32.255** | 16.769 | 3.517E-02 |
| [218268_at](https://www.affymetrix.com/LinkServlet?probeset=218268_at) | [TBC1D15](http://www.ncbi.nlm.nih.gov/entrez/query.fcgi?cmd=search&db=gene&term=TBC1D15) | TBC1 domain family, member 15 | **0.780** | **350.588** | 83.871 | **273.626** | 38.826 | 1.061E-02 |
| [206075_s_at](https://www.affymetrix.com/LinkServlet?probeset=206075_s_at) | [CSNK2A1](http://www.ncbi.nlm.nih.gov/entrez/query.fcgi?cmd=search&db=gene&term=CSNK2A1) | casein kinase 2, alpha 1 polypeptide | **0.781** | **231.504** | 32.530 | **180.699** | 43.312 | 2.313E-03 |
| [204448_s_at](https://www.affymetrix.com/LinkServlet?probeset=204448_s_at) | [PDCL](http://www.ncbi.nlm.nih.gov/entrez/query.fcgi?cmd=search&db=gene&term=PDCL) | phosducin-like | **0.782** | **160.642** | 31.912 | **125.576** | 37.355 | 1.026E-02 |
| [225435_at](https://www.affymetrix.com/LinkServlet?probeset=225435_at) | [SSR1](http://www.ncbi.nlm.nih.gov/entrez/query.fcgi?cmd=search&db=gene&term=SSR1) | signal sequence receptor, alpha | **0.782** | **460.168** | 129.080 | **359.772** | 84.986 | 4.677E-02 |
| [210256_s_at](https://www.affymetrix.com/LinkServlet?probeset=210256_s_at) | [PIP5K1A](http://www.ncbi.nlm.nih.gov/entrez/query.fcgi?cmd=search&db=gene&term=PIP5K1A) | phosphatidylinositol-4-phosphate 5-kinase, type I, alpha | **0.782** | **8.983** | 2.495 | **7.023** | 1.652 | 3.150E-02 |
| [232057_at](https://www.affymetrix.com/LinkServlet?probeset=232057_at) | [SLC7A6OS](http://www.ncbi.nlm.nih.gov/entrez/query.fcgi?cmd=search&db=gene&term=SLC7A6OS) | solute carrier family 7, member 6 opposite strand | **0.782** | **440.802** | 115.681 | **344.771** | 110.099 | 3.348E-02 |
| [224708_at](https://www.affymetrix.com/LinkServlet?probeset=224708_at) | [KIAA2013](http://www.ncbi.nlm.nih.gov/entrez/query.fcgi?cmd=search&db=gene&term=KIAA2013) | KIAA2013 | **0.783** | **692.279** | 169.978 | **541.744** | 119.756 | 2.965E-02 |
| [223197_s_at](https://www.affymetrix.com/LinkServlet?probeset=223197_s_at) | [SMARCAD1](http://www.ncbi.nlm.nih.gov/entrez/query.fcgi?cmd=search&db=gene&term=SMARCAD1) | SWI/SNF-related, matrix-associated actin-dependent regulator of chromatin, subfamily a, containing DEAD/H box 1 | **0.783** | **152.785** | 37.328 | **119.595** | 36.788 | 2.461E-02 |
| [217991_x_at](https://www.affymetrix.com/LinkServlet?probeset=217991_x_at) | [SSBP3](http://www.ncbi.nlm.nih.gov/entrez/query.fcgi?cmd=search&db=gene&term=SSBP3) | single stranded DNA binding protein 3 | **0.783** | **150.232** | 38.490 | **117.600** | 47.435 | 2.500E-02 |
| [225888_at](https://www.affymetrix.com/LinkServlet?probeset=225888_at) | [NAA25](http://www.ncbi.nlm.nih.gov/entrez/query.fcgi?cmd=search&db=gene&term=NAA25) | N(alpha)-acetyltransferase 25, NatB auxiliary subunit | **0.783** | **110.071** | 27.454 | **86.189** | 28.553 | 3.471E-02 |
| [223177_at](https://www.affymetrix.com/LinkServlet?probeset=223177_at) | [NT5DC1](http://www.ncbi.nlm.nih.gov/entrez/query.fcgi?cmd=search&db=gene&term=NT5DC1) | 5'-nucleotidase domain containing 1 | **0.783** | **316.609** | 84.232 | **247.920** | 67.556 | 2.582E-02 |
| [221963_x_at](https://www.affymetrix.com/LinkServlet?probeset=221963_x_at) | [NA](http://www.ncbi.nlm.nih.gov/entrez/query.fcgi?cmd=search&db=gene&term=NA) | NA | **0.783** | **167.097** | 34.032 | **130.859** | 57.360 | 2.024E-02 |
| [213231_at](https://www.affymetrix.com/LinkServlet?probeset=213231_at) | [DMWD](http://www.ncbi.nlm.nih.gov/entrez/query.fcgi?cmd=search&db=gene&term=DMWD) | dystrophia myotonica, WD repeat containing | **0.783** | **26.505** | 5.944 | **20.760** | 8.434 | 3.327E-02 |
| [226873_at](https://www.affymetrix.com/LinkServlet?probeset=226873_at) | [NA](http://www.ncbi.nlm.nih.gov/entrez/query.fcgi?cmd=search&db=gene&term=NA) | NA | **0.784** | **121.657** | 28.373 | **95.343** | 31.239 | 2.485E-02 |
| [209551_at](https://www.affymetrix.com/LinkServlet?probeset=209551_at) | [YIPF4](http://www.ncbi.nlm.nih.gov/entrez/query.fcgi?cmd=search&db=gene&term=YIPF4) | Yip1 domain family, member 4 | **0.784** | **96.944** | 30.087 | **75.987** | 26.885 | 3.097E-02 |
| [212271_at](https://www.affymetrix.com/LinkServlet?probeset=212271_at) | [MAPK1](http://www.ncbi.nlm.nih.gov/entrez/query.fcgi?cmd=search&db=gene&term=MAPK1) | mitogen-activated protein kinase 1 | **0.784** | **180.899** | 51.112 | **141.818** | 35.862 | 3.395E-02 |
| [212160_at](https://www.affymetrix.com/LinkServlet?probeset=212160_at) | [XPOT](http://www.ncbi.nlm.nih.gov/entrez/query.fcgi?cmd=search&db=gene&term=XPOT) | exportin, tRNA (nuclear export receptor for tRNAs) | **0.785** | **435.963** | 96.442 | **342.161** | 81.973 | 6.804E-03 |
| [225774_at](https://www.affymetrix.com/LinkServlet?probeset=225774_at) | [RSPRY1](http://www.ncbi.nlm.nih.gov/entrez/query.fcgi?cmd=search&db=gene&term=RSPRY1) | ring finger and SPRY domain containing 1 | **0.785** | **140.334** | 30.752 | **110.172** | 20.953 | 1.241E-02 |
| [239231_at](https://www.affymetrix.com/LinkServlet?probeset=239231_at) | [NA](http://www.ncbi.nlm.nih.gov/entrez/query.fcgi?cmd=search&db=gene&term=NA) | NA | **0.785** | **884.098** | 173.950 | **694.257** | 118.094 | 3.479E-03 |
| [218506_x_at](https://www.affymetrix.com/LinkServlet?probeset=218506_x_at) | [GLYR1](http://www.ncbi.nlm.nih.gov/entrez/query.fcgi?cmd=search&db=gene&term=GLYR1) | glyoxylate reductase 1 homolog (Arabidopsis) | **0.785** | **288.710** | 68.281 | **226.766** | 68.887 | 2.862E-02 |
| [238860_at](https://www.affymetrix.com/LinkServlet?probeset=238860_at) | [C6orf130](http://www.ncbi.nlm.nih.gov/entrez/query.fcgi?cmd=search&db=gene&term=C6orf130) | chromosome 6 open reading frame 130 | **0.786** | **385.841** | 80.625 | **303.120** | 78.427 | 1.481E-02 |
| [201928_at](https://www.affymetrix.com/LinkServlet?probeset=201928_at) | [PKP4](http://www.ncbi.nlm.nih.gov/entrez/query.fcgi?cmd=search&db=gene&term=PKP4) | plakophilin 4 | **0.786** | **375.664** | 63.288 | **295.144** | 94.815 | 8.232E-03 |
| [218918_at](https://www.affymetrix.com/LinkServlet?probeset=218918_at) | [MAN1C1](http://www.ncbi.nlm.nih.gov/entrez/query.fcgi?cmd=search&db=gene&term=MAN1C1) | mannosidase, alpha, class 1C, member 1 | **0.786** | **98.973** | 27.946 | **77.776** | 29.859 | 3.790E-02 |
| [225559_at](https://www.affymetrix.com/LinkServlet?probeset=225559_at) | [C3orf19](http://www.ncbi.nlm.nih.gov/entrez/query.fcgi?cmd=search&db=gene&term=C3orf19) | chromosome 3 open reading frame 19 | **0.786** | **195.879** | 49.276 | **153.939** | 34.725 | 2.245E-02 |
| [225091_at](https://www.affymetrix.com/LinkServlet?probeset=225091_at) | [ZCCHC3](http://www.ncbi.nlm.nih.gov/entrez/query.fcgi?cmd=search&db=gene&term=ZCCHC3) | zinc finger, CCHC domain containing 3 | **0.786** | **433.027** | 93.423 | **340.439** | 93.247 | 3.990E-02 |
| [232103_at](https://www.affymetrix.com/LinkServlet?probeset=232103_at) | [BPNT1](http://www.ncbi.nlm.nih.gov/entrez/query.fcgi?cmd=search&db=gene&term=BPNT1) | 3'(2'), 5'-bisphosphate nucleotidase 1 | **0.786** | **82.917** | 21.135 | **65.190** | 21.655 | 4.509E-02 |
| [201932_at](https://www.affymetrix.com/LinkServlet?probeset=201932_at) | [LRRC41](http://www.ncbi.nlm.nih.gov/entrez/query.fcgi?cmd=search&db=gene&term=LRRC41) | leucine rich repeat containing 41 | **0.786** | **126.462** | 26.375 | **99.431** | 31.048 | 1.383E-02 |
| [224669_at](https://www.affymetrix.com/LinkServlet?probeset=224669_at) | [NA](http://www.ncbi.nlm.nih.gov/entrez/query.fcgi?cmd=search&db=gene&term=NA) | NA | **0.787** | **332.852** | 76.898 | **261.839** | 69.633 | 2.442E-02 |
| [212496_s_at](https://www.affymetrix.com/LinkServlet?probeset=212496_s_at) | [KDM4B](http://www.ncbi.nlm.nih.gov/entrez/query.fcgi?cmd=search&db=gene&term=KDM4B) | lysine (K)-specific demethylase 4B | **0.787** | **512.846** | 163.433 | **403.512** | 99.550 | 3.745E-02 |
| [218172_s_at](https://www.affymetrix.com/LinkServlet?probeset=218172_s_at) | [DERL1](http://www.ncbi.nlm.nih.gov/entrez/query.fcgi?cmd=search&db=gene&term=DERL1) | Der1-like domain family, member 1 | **0.787** | **356.684** | 63.335 | **280.674** | 77.818 | 8.846E-03 |
| [219029_at](https://www.affymetrix.com/LinkServlet?probeset=219029_at) | [C5orf28](http://www.ncbi.nlm.nih.gov/entrez/query.fcgi?cmd=search&db=gene&term=C5orf28) | chromosome 5 open reading frame 28 | **0.789** | **278.983** | 57.481 | **219.992** | 101.470 | 3.752E-02 |
| [203882_at](https://www.affymetrix.com/LinkServlet?probeset=203882_at) | [IRF9](http://www.ncbi.nlm.nih.gov/entrez/query.fcgi?cmd=search&db=gene&term=IRF9) | interferon regulatory factor 9 | **0.789** | **545.132** | 144.276 | **429.912** | 119.612 | 2.865E-02 |
| [213473_at](https://www.affymetrix.com/LinkServlet?probeset=213473_at) | [BRAP](http://www.ncbi.nlm.nih.gov/entrez/query.fcgi?cmd=search&db=gene&term=BRAP) | BRCA1 associated protein | **0.789** | **191.319** | 40.121 | **150.962** | 25.237 | 6.466E-03 |
| [209221_s_at](https://www.affymetrix.com/LinkServlet?probeset=209221_s_at) | [OSBPL2](http://www.ncbi.nlm.nih.gov/entrez/query.fcgi?cmd=search&db=gene&term=OSBPL2) | oxysterol binding protein-like 2 | **0.789** | **283.581** | 63.151 | **223.805** | 76.125 | 4.663E-02 |
| [204225_at](https://www.affymetrix.com/LinkServlet?probeset=204225_at) | [HDAC4](http://www.ncbi.nlm.nih.gov/entrez/query.fcgi?cmd=search&db=gene&term=HDAC4) | histone deacetylase 4 | **0.790** | **557.094** | 134.018 | **439.946** | 120.228 | 2.912E-02 |
| [230029_x_at](https://www.affymetrix.com/LinkServlet?probeset=230029_x_at) | [UBR3](http://www.ncbi.nlm.nih.gov/entrez/query.fcgi?cmd=search&db=gene&term=UBR3) | ubiquitin protein ligase E3 component n-recognin 3 (putative) | **0.790** | **391.193** | 78.283 | **309.046** | 81.842 | 8.119E-03 |
| [200945_s_at](https://www.affymetrix.com/LinkServlet?probeset=200945_s_at) | [SEC31A](http://www.ncbi.nlm.nih.gov/entrez/query.fcgi?cmd=search&db=gene&term=SEC31A) | SEC31 homolog A (S. cerevisiae) | **0.791** | **768.649** | 158.568 | **607.694** | 136.220 | 7.303E-03 |
| [206707_x_at](https://www.affymetrix.com/LinkServlet?probeset=206707_x_at) | [FAM65B](http://www.ncbi.nlm.nih.gov/entrez/query.fcgi?cmd=search&db=gene&term=FAM65B) | family with sequence similarity 65, member B | **0.791** | **325.341** | 105.929 | **257.401** | 46.337 | 3.566E-02 |
| [226268_at](https://www.affymetrix.com/LinkServlet?probeset=226268_at) | [RAB21](http://www.ncbi.nlm.nih.gov/entrez/query.fcgi?cmd=search&db=gene&term=RAB21) | RAB21, member RAS oncogene family | **0.793** | **121.109** | 26.498 | **95.982** | 17.245 | 1.189E-02 |
| [224759_s_at](https://www.affymetrix.com/LinkServlet?probeset=224759_s_at) | [C12orf23](http://www.ncbi.nlm.nih.gov/entrez/query.fcgi?cmd=search&db=gene&term=C12orf23) | chromosome 12 open reading frame 23 | **0.793** | **458.219** | 141.796 | **363.344** | 93.422 | 3.790E-02 |
| [230126_s_at](https://www.affymetrix.com/LinkServlet?probeset=230126_s_at) | [KDM4B](http://www.ncbi.nlm.nih.gov/entrez/query.fcgi?cmd=search&db=gene&term=KDM4B) | lysine (K)-specific demethylase 4B | **0.793** | **494.675** | 109.921 | **392.321** | 95.612 | 1.245E-02 |
| [226821_at](https://www.affymetrix.com/LinkServlet?probeset=226821_at) | [NA](http://www.ncbi.nlm.nih.gov/entrez/query.fcgi?cmd=search&db=gene&term=NA) | NA | **0.793** | **101.542** | 22.610 | **80.555** | 14.851 | 4.024E-02 |
| [225648_at](https://www.affymetrix.com/LinkServlet?probeset=225648_at) | [STK35](http://www.ncbi.nlm.nih.gov/entrez/query.fcgi?cmd=search&db=gene&term=STK35) | serine/threonine kinase 35 | **0.794** | **174.606** | 39.649 | **138.584** | 47.085 | 4.582E-02 |
| [223090_x_at](https://www.affymetrix.com/LinkServlet?probeset=223090_x_at) | [VEZT](http://www.ncbi.nlm.nih.gov/entrez/query.fcgi?cmd=search&db=gene&term=VEZT) | vezatin, adherens junctions transmembrane protein | **0.794** | **265.513** | 36.703 | **210.869** | 85.390 | 1.568E-02 |
| [202926_at](https://www.affymetrix.com/LinkServlet?probeset=202926_at) | [NBAS](http://www.ncbi.nlm.nih.gov/entrez/query.fcgi?cmd=search&db=gene&term=NBAS) | neuroblastoma amplified sequence | **0.795** | **184.685** | 50.622 | **146.732** | 33.468 | 3.993E-02 |
| [202513_s_at](https://www.affymetrix.com/LinkServlet?probeset=202513_s_at) | [PPP2R5D](http://www.ncbi.nlm.nih.gov/entrez/query.fcgi?cmd=search&db=gene&term=PPP2R5D) | protein phosphatase 2, regulatory subunit B', delta | **0.797** | **148.957** | 45.154 | **118.650** | 24.275 | 4.564E-02 |
| [230383_x_at](https://www.affymetrix.com/LinkServlet?probeset=230383_x_at) | [NA](http://www.ncbi.nlm.nih.gov/entrez/query.fcgi?cmd=search&db=gene&term=NA) | NA | **0.797** | **633.550** | 133.894 | **504.913** | 136.284 | 1.759E-02 |
| [219540_at](https://www.affymetrix.com/LinkServlet?probeset=219540_at) | [ZNF267](http://www.ncbi.nlm.nih.gov/entrez/query.fcgi?cmd=search&db=gene&term=ZNF267) | zinc finger protein 267 | **0.798** | **382.953** | 87.358 | **305.515** | 97.688 | 1.780E-02 |
| [225984_at](https://www.affymetrix.com/LinkServlet?probeset=225984_at) | [PRKAA1](http://www.ncbi.nlm.nih.gov/entrez/query.fcgi?cmd=search&db=gene&term=PRKAA1) | protein kinase, AMP-activated, alpha 1 catalytic subunit | **0.798** | **320.143** | 94.130 | **255.429** | 72.765 | 4.758E-02 |
| [235241_at](https://www.affymetrix.com/LinkServlet?probeset=235241_at) | [SLC38A9](http://www.ncbi.nlm.nih.gov/entrez/query.fcgi?cmd=search&db=gene&term=SLC38A9) | solute carrier family 38, member 9 | **0.798** | **296.917** | 65.862 | **236.934** | 47.397 | 1.671E-02 |
| [222552_at](https://www.affymetrix.com/LinkServlet?probeset=222552_at) | [GOLT1B](http://www.ncbi.nlm.nih.gov/entrez/query.fcgi?cmd=search&db=gene&term=GOLT1B) | golgi transport 1B | **0.799** | **277.657** | 51.162 | **221.829** | 53.305 | 5.967E-03 |
| [233380_s_at](https://www.affymetrix.com/LinkServlet?probeset=233380_s_at) | [RUFY1](http://www.ncbi.nlm.nih.gov/entrez/query.fcgi?cmd=search&db=gene&term=RUFY1) | RUN and FYVE domain containing 1 | **0.799** | **96.578** | 29.122 | **77.196** | 17.541 | 4.786E-02 |
| [225669_at](https://www.affymetrix.com/LinkServlet?probeset=225669_at) | [IFNAR1](http://www.ncbi.nlm.nih.gov/entrez/query.fcgi?cmd=search&db=gene&term=IFNAR1) | interferon (alpha, beta and omega) receptor 1 | **0.800** | **260.395** | 60.085 | **208.258** | 76.887 | 3.771E-02 |
| [222469_s_at](https://www.affymetrix.com/LinkServlet?probeset=222469_s_at) | [TOLLIP](http://www.ncbi.nlm.nih.gov/entrez/query.fcgi?cmd=search&db=gene&term=TOLLIP) | toll interacting protein | **0.800** | **237.109** | 60.494 | **189.707** | 82.228 | 3.913E-02 |
| [225201_s_at](https://www.affymetrix.com/LinkServlet?probeset=225201_s_at) | [MRPL14](http://www.ncbi.nlm.nih.gov/entrez/query.fcgi?cmd=search&db=gene&term=MRPL14) | mitochondrial ribosomal protein L14 | **0.800** | **200.735** | 55.832 | **160.635** | 53.936 | 4.989E-02 |
| [1552426_a_at](https://www.affymetrix.com/LinkServlet?probeset=1552426_a_at) | [TM2D3](http://www.ncbi.nlm.nih.gov/entrez/query.fcgi?cmd=search&db=gene&term=TM2D3) | TM2 domain containing 3 | **0.800** | **964.087** | 158.713 | **771.735** | 77.667 | 1.047E-03 |
| [201776_s_at](https://www.affymetrix.com/LinkServlet?probeset=201776_s_at) | [KIAA0494](http://www.ncbi.nlm.nih.gov/entrez/query.fcgi?cmd=search&db=gene&term=KIAA0494) | KIAA0494 | **0.801** | **342.867** | 82.714 | **274.500** | 79.228 | 3.507E-02 |
| [224352_s_at](https://www.affymetrix.com/LinkServlet?probeset=224352_s_at) | [CFL2](http://www.ncbi.nlm.nih.gov/entrez/query.fcgi?cmd=search&db=gene&term=CFL2) | cofilin 2 (muscle) | **0.801** | **294.591** | 56.959 | **235.880** | 40.617 | 7.590E-03 |
| [209404_s_at](https://www.affymetrix.com/LinkServlet?probeset=209404_s_at) | [TMED7](http://www.ncbi.nlm.nih.gov/entrez/query.fcgi?cmd=search&db=gene&term=TMED7) | transmembrane emp24 protein transport domain containing 7 | **0.801** | **104.945** | 24.449 | **84.032** | 32.176 | 3.397E-02 |
| [212138_at](https://www.affymetrix.com/LinkServlet?probeset=212138_at) | [PDS5A](http://www.ncbi.nlm.nih.gov/entrez/query.fcgi?cmd=search&db=gene&term=PDS5A) | PDS5, regulator of cohesion maintenance, homolog A (S. cerevisiae) | **0.801** | **562.397** | 80.608 | **450.391** | 63.693 | 4.319E-04 |
| [204590_x_at](https://www.affymetrix.com/LinkServlet?probeset=204590_x_at) | [VPS33A](http://www.ncbi.nlm.nih.gov/entrez/query.fcgi?cmd=search&db=gene&term=VPS33A) | vacuolar protein sorting 33 homolog A (S. cerevisiae) | **0.801** | **289.097** | 67.120 | **231.621** | 35.235 | 1.832E-02 |
| [201970_s_at](https://www.affymetrix.com/LinkServlet?probeset=201970_s_at) | [NASP](http://www.ncbi.nlm.nih.gov/entrez/query.fcgi?cmd=search&db=gene&term=NASP) | nuclear autoantigenic sperm protein (histone-binding) | **0.802** | **209.144** | 41.904 | **167.666** | 67.543 | 3.233E-02 |
| [223624_at](https://www.affymetrix.com/LinkServlet?probeset=223624_at) | [ANUBL1](http://www.ncbi.nlm.nih.gov/entrez/query.fcgi?cmd=search&db=gene&term=ANUBL1) | AN1, ubiquitin-like, homolog (Xenopus laevis) | **0.802** | **103.024** | 26.259 | **82.593** | 28.654 | 4.469E-02 |
| [210296_s_at](https://www.affymetrix.com/LinkServlet?probeset=210296_s_at) | [PEX2](http://www.ncbi.nlm.nih.gov/entrez/query.fcgi?cmd=search&db=gene&term=PEX2) | peroxisomal biogenesis factor 2 | **0.802** | **266.031** | 50.667 | **213.306** | 49.475 | 8.863E-03 |
| [235457_at](https://www.affymetrix.com/LinkServlet?probeset=235457_at) | [MAML2](http://www.ncbi.nlm.nih.gov/entrez/query.fcgi?cmd=search&db=gene&term=MAML2) | mastermind-like 2 (Drosophila) | **0.803** | **260.658** | 64.514 | **209.269** | 49.592 | 3.818E-02 |
| [202907_s_at](https://www.affymetrix.com/LinkServlet?probeset=202907_s_at) | [NBN](http://www.ncbi.nlm.nih.gov/entrez/query.fcgi?cmd=search&db=gene&term=NBN) | nibrin | **0.803** | **569.710** | 114.741 | **457.455** | 88.181 | 1.028E-02 |
| [211953_s_at](https://www.affymetrix.com/LinkServlet?probeset=211953_s_at) | [IPO5](http://www.ncbi.nlm.nih.gov/entrez/query.fcgi?cmd=search&db=gene&term=IPO5) | importin 5 | **0.803** | **416.444** | 82.583 | **334.445** | 105.644 | 2.233E-02 |
| [218361_at](https://www.affymetrix.com/LinkServlet?probeset=218361_at) | [GOLPH3L](http://www.ncbi.nlm.nih.gov/entrez/query.fcgi?cmd=search&db=gene&term=GOLPH3L) | golgi phosphoprotein 3-like | **0.803** | **737.413** | 101.558 | **592.368** | 82.440 | 3.811E-04 |
| [1554379_a_at](https://www.affymetrix.com/LinkServlet?probeset=1554379_a_at) | [TP73](http://www.ncbi.nlm.nih.gov/entrez/query.fcgi?cmd=search&db=gene&term=TP73) | tumor protein p73 | **0.804** | **8.332** | 2.367 | **6.702** | 1.324 | 4.655E-02 |
| [227687_at](https://www.affymetrix.com/LinkServlet?probeset=227687_at) | [HYLS1](http://www.ncbi.nlm.nih.gov/entrez/query.fcgi?cmd=search&db=gene&term=HYLS1) | hydrolethalus syndrome 1 | **0.805** | **94.057** | 24.439 | **75.689** | 24.503 | 4.082E-02 |
| [225374_at](https://www.affymetrix.com/LinkServlet?probeset=225374_at) | [TMEM199](http://www.ncbi.nlm.nih.gov/entrez/query.fcgi?cmd=search&db=gene&term=TMEM199) | transmembrane protein 199 | **0.806** | **138.407** | 36.158 | **111.540** | 30.250 | 4.397E-02 |
| [213795_s_at](https://www.affymetrix.com/LinkServlet?probeset=213795_s_at) | [PTPRA](http://www.ncbi.nlm.nih.gov/entrez/query.fcgi?cmd=search&db=gene&term=PTPRA) | protein tyrosine phosphatase, receptor type, A | **0.806** | **322.273** | 62.456 | **259.726** | 62.398 | 1.427E-02 |
| [212366_at](https://www.affymetrix.com/LinkServlet?probeset=212366_at) | [ZNF292](http://www.ncbi.nlm.nih.gov/entrez/query.fcgi?cmd=search&db=gene&term=ZNF292) | zinc finger protein 292 | **0.806** | **556.865** | 99.187 | **448.834** | 98.778 | 1.083E-02 |
| [208839_s_at](https://www.affymetrix.com/LinkServlet?probeset=208839_s_at) | [CAND1](http://www.ncbi.nlm.nih.gov/entrez/query.fcgi?cmd=search&db=gene&term=CAND1) | cullin-associated and neddylation-dissociated 1 | **0.806** | **287.815** | 58.489 | **232.043** | 64.687 | 2.477E-02 |
| [217393_x_at](https://www.affymetrix.com/LinkServlet?probeset=217393_x_at) | [UBE2NL](http://www.ncbi.nlm.nih.gov/entrez/query.fcgi?cmd=search&db=gene&term=UBE2NL) | ubiquitin-conjugating enzyme E2N-like | **0.806** | **45.704** | 11.347 | **36.850** | 11.903 | 3.294E-02 |
| [212474_at](https://www.affymetrix.com/LinkServlet?probeset=212474_at) | [AVL9](http://www.ncbi.nlm.nih.gov/entrez/query.fcgi?cmd=search&db=gene&term=AVL9) | AVL9 homolog (S. cerevisiase) | **0.807** | **358.461** | 84.978 | **289.201** | 63.386 | 2.930E-02 |
| [209674_at](https://www.affymetrix.com/LinkServlet?probeset=209674_at) | [CRY1](http://www.ncbi.nlm.nih.gov/entrez/query.fcgi?cmd=search&db=gene&term=CRY1) | cryptochrome 1 (photolyase-like) | **0.807** | **196.392** | 45.693 | **158.450** | 75.688 | 3.483E-02 |
| [231921_at](https://www.affymetrix.com/LinkServlet?probeset=231921_at) | [DCAF17](http://www.ncbi.nlm.nih.gov/entrez/query.fcgi?cmd=search&db=gene&term=DCAF17) | DDB1 and CUL4 associated factor 17 | **0.807** | **244.578** | 51.968 | **197.337** | 85.397 | 4.329E-02 |
| [221013_s_at](https://www.affymetrix.com/LinkServlet?probeset=221013_s_at) | [APOL2](http://www.ncbi.nlm.nih.gov/entrez/query.fcgi?cmd=search&db=gene&term=APOL2) | apolipoprotein L, 2 | **0.808** | **54.544** | 11.999 | **44.064** | 17.149 | 4.808E-02 |
| [203360_s_at](https://www.affymetrix.com/LinkServlet?probeset=203360_s_at) | [MYCBP](http://www.ncbi.nlm.nih.gov/entrez/query.fcgi?cmd=search&db=gene&term=MYCBP) | c-myc binding protein | **0.808** | **158.411** | 40.017 | **128.019** | 42.210 | 4.858E-02 |
| [223675_s_at](https://www.affymetrix.com/LinkServlet?probeset=223675_s_at) | [VEZT](http://www.ncbi.nlm.nih.gov/entrez/query.fcgi?cmd=search&db=gene&term=VEZT) | vezatin, adherens junctions transmembrane protein | **0.808** | **183.491** | 23.703 | **148.303** | 56.203 | 1.930E-02 |
| [203531_at](https://www.affymetrix.com/LinkServlet?probeset=203531_at) | [CUL5](http://www.ncbi.nlm.nih.gov/entrez/query.fcgi?cmd=search&db=gene&term=CUL5) | cullin 5 | **0.808** | **412.611** | 86.628 | **333.554** | 61.437 | 2.594E-02 |
| [224629_at](https://www.affymetrix.com/LinkServlet?probeset=224629_at) | [LMAN1](http://www.ncbi.nlm.nih.gov/entrez/query.fcgi?cmd=search&db=gene&term=LMAN1) | lectin, mannose-binding, 1 | **0.809** | **912.688** | 254.438 | **738.758** | 155.617 | 3.674E-02 |
| [223133_at](https://www.affymetrix.com/LinkServlet?probeset=223133_at) | [TMEM14B](http://www.ncbi.nlm.nih.gov/entrez/query.fcgi?cmd=search&db=gene&term=TMEM14B) | transmembrane protein 14B | **0.809** | **659.391** | 147.670 | **533.756** | 159.256 | 2.915E-02 |
| [222416_at](https://www.affymetrix.com/LinkServlet?probeset=222416_at) | [ALDH18A1](http://www.ncbi.nlm.nih.gov/entrez/query.fcgi?cmd=search&db=gene&term=ALDH18A1) | aldehyde dehydrogenase 18 family, member A1 | **0.810** | **241.982** | 62.005 | **195.887** | 60.328 | 4.741E-02 |
| [238318_at](https://www.affymetrix.com/LinkServlet?probeset=238318_at) | [NA](http://www.ncbi.nlm.nih.gov/entrez/query.fcgi?cmd=search&db=gene&term=NA) | NA | **0.810** | **7.406** | 2.585 | **6.000** | 0.000 | 4.976E-02 |
| [242844_at](https://www.affymetrix.com/LinkServlet?probeset=242844_at) | [PGGT1B](http://www.ncbi.nlm.nih.gov/entrez/query.fcgi?cmd=search&db=gene&term=PGGT1B) | protein geranylgeranyltransferase type I, beta subunit | **0.810** | **237.899** | 55.131 | **192.750** | 24.948 | 1.845E-02 |
| [209141_at](https://www.affymetrix.com/LinkServlet?probeset=209141_at) | [UBE2G1](http://www.ncbi.nlm.nih.gov/entrez/query.fcgi?cmd=search&db=gene&term=UBE2G1) | ubiquitin-conjugating enzyme E2G 1 (UBC7 homolog, yeast) | **0.811** | **386.018** | 77.307 | **313.199** | 80.498 | 2.107E-02 |
| [225816_at](https://www.affymetrix.com/LinkServlet?probeset=225816_at) | [PHF17](http://www.ncbi.nlm.nih.gov/entrez/query.fcgi?cmd=search&db=gene&term=PHF17) | PHD finger protein 17 | **0.812** | **246.500** | 38.132 | **200.062** | 60.230 | 1.057E-02 |
| [200854_at](https://www.affymetrix.com/LinkServlet?probeset=200854_at) | [NCOR1](http://www.ncbi.nlm.nih.gov/entrez/query.fcgi?cmd=search&db=gene&term=NCOR1) | nuclear receptor corepressor 1 | **0.812** | **532.309** | 114.898 | **432.169** | 111.734 | 4.790E-02 |
| [1558537_x_at](https://www.affymetrix.com/LinkServlet?probeset=1558537_x_at) | [ZNF844](http://www.ncbi.nlm.nih.gov/entrez/query.fcgi?cmd=search&db=gene&term=ZNF844) | zinc finger protein 844 | **0.812** | **7.389** | 2.295 | **6.000** | 0.000 | 4.935E-02 |
| [212748_at](https://www.affymetrix.com/LinkServlet?probeset=212748_at) | [MKL1](http://www.ncbi.nlm.nih.gov/entrez/query.fcgi?cmd=search&db=gene&term=MKL1) | megakaryoblastic leukemia (translocation) 1 | **0.813** | **170.846** | 36.439 | **138.843** | 28.351 | 1.717E-02 |
| [231944_at](https://www.affymetrix.com/LinkServlet?probeset=231944_at) | [ERO1LB](http://www.ncbi.nlm.nih.gov/entrez/query.fcgi?cmd=search&db=gene&term=ERO1LB) | ERO1-like beta (S. cerevisiae) | **0.814** | **335.000** | 64.813 | **272.712** | 60.542 | 1.034E-02 |
| [209705_at](https://www.affymetrix.com/LinkServlet?probeset=209705_at) | [MTF2](http://www.ncbi.nlm.nih.gov/entrez/query.fcgi?cmd=search&db=gene&term=MTF2) | metal response element binding transcription factor 2 | **0.814** | **317.471** | 38.174 | **258.446** | 67.555 | 8.691E-03 |
| [200733_s_at](https://www.affymetrix.com/LinkServlet?probeset=200733_s_at) | [PTP4A1](http://www.ncbi.nlm.nih.gov/entrez/query.fcgi?cmd=search&db=gene&term=PTP4A1) | protein tyrosine phosphatase type IVA, member 1 | **0.814** | **717.957** | 168.985 | **584.742** | 152.551 | 4.279E-02 |
| [214971_s_at](https://www.affymetrix.com/LinkServlet?probeset=214971_s_at) | [ST6GAL1](http://www.ncbi.nlm.nih.gov/entrez/query.fcgi?cmd=search&db=gene&term=ST6GAL1) | ST6 beta-galactosamide alpha-2,6-sialyltranferase 1 | **0.815** | **7.483** | 2.393 | **6.095** | 0.215 | 4.562E-02 |
| [242943_at](https://www.affymetrix.com/LinkServlet?probeset=242943_at) | [ST8SIA4](http://www.ncbi.nlm.nih.gov/entrez/query.fcgi?cmd=search&db=gene&term=ST8SIA4) | ST8 alpha-N-acetyl-neuraminide alpha-2,8-sialyltransferase 4 | **0.815** | **344.059** | 56.838 | **280.319** | 49.080 | 8.201E-03 |
| [223384_s_at](https://www.affymetrix.com/LinkServlet?probeset=223384_s_at) | [TRIM4](http://www.ncbi.nlm.nih.gov/entrez/query.fcgi?cmd=search&db=gene&term=TRIM4) | tripartite motif containing 4 | **0.815** | **239.484** | 42.697 | **195.219** | 30.610 | 5.104E-03 |
| [212738_at](https://www.affymetrix.com/LinkServlet?probeset=212738_at) | [ARHGAP19](http://www.ncbi.nlm.nih.gov/entrez/query.fcgi?cmd=search&db=gene&term=ARHGAP19) | Rho GTPase activating protein 19 | **0.815** | **282.618** | 57.993 | **230.391** | 39.161 | 1.031E-02 |
| [206688_s_at](https://www.affymetrix.com/LinkServlet?probeset=206688_s_at) | [CPSF4](http://www.ncbi.nlm.nih.gov/entrez/query.fcgi?cmd=search&db=gene&term=CPSF4) | cleavage and polyadenylation specific factor 4, 30kDa | **0.815** | **193.880** | 42.073 | **158.056** | 27.439 | 3.625E-02 |
| [224850_at](https://www.affymetrix.com/LinkServlet?probeset=224850_at) | [ATAD1](http://www.ncbi.nlm.nih.gov/entrez/query.fcgi?cmd=search&db=gene&term=ATAD1) | ATPase family, AAA domain containing 1 | **0.815** | **332.112** | 74.803 | **270.762** | 43.846 | 2.451E-02 |
| [218802_at](https://www.affymetrix.com/LinkServlet?probeset=218802_at) | [CCDC109B](http://www.ncbi.nlm.nih.gov/entrez/query.fcgi?cmd=search&db=gene&term=CCDC109B) | coiled-coil domain containing 109B | **0.815** | **843.357** | 175.713 | **687.621** | 72.115 | 1.105E-02 |
| [214317_x_at](https://www.affymetrix.com/LinkServlet?probeset=214317_x_at) | [RPS9](http://www.ncbi.nlm.nih.gov/entrez/query.fcgi?cmd=search&db=gene&term=RPS9) | ribosomal protein S9 | **0.815** | **1810.441** | 373.950 | **1476.317** | 377.563 | 2.853E-02 |
| [213000_at](https://www.affymetrix.com/LinkServlet?probeset=213000_at) | [MORC3](http://www.ncbi.nlm.nih.gov/entrez/query.fcgi?cmd=search&db=gene&term=MORC3) | MORC family CW-type zinc finger 3 | **0.816** | **562.210** | 111.886 | **458.735** | 138.164 | 4.367E-02 |
| [218379_at](https://www.affymetrix.com/LinkServlet?probeset=218379_at) | [RBM7](http://www.ncbi.nlm.nih.gov/entrez/query.fcgi?cmd=search&db=gene&term=RBM7) | RNA binding motif protein 7 | **0.816** | **246.691** | 49.340 | **201.293** | 54.058 | 2.707E-02 |
| [200970_s_at](https://www.affymetrix.com/LinkServlet?probeset=200970_s_at) | [SERP1](http://www.ncbi.nlm.nih.gov/entrez/query.fcgi?cmd=search&db=gene&term=SERP1) | stress-associated endoplasmic reticulum protein 1 | **0.816** | **344.305** | 85.504 | **280.954** | 77.312 | 3.440E-02 |
| [54037_at](https://www.affymetrix.com/LinkServlet?probeset=54037_at) | [HPS4](http://www.ncbi.nlm.nih.gov/entrez/query.fcgi?cmd=search&db=gene&term=HPS4) | Hermansky-Pudlak syndrome 4 | **0.817** | **197.424** | 37.133 | **161.208** | 40.827 | 1.809E-02 |
| [212911_at](https://www.affymetrix.com/LinkServlet?probeset=212911_at) | [DNAJC16](http://www.ncbi.nlm.nih.gov/entrez/query.fcgi?cmd=search&db=gene&term=DNAJC16) | DnaJ (Hsp40) homolog, subfamily C, member 16 | **0.817** | **311.390** | 70.004 | **254.490** | 84.270 | 4.217E-02 |
| [202842_s_at](https://www.affymetrix.com/LinkServlet?probeset=202842_s_at) | [DNAJB9](http://www.ncbi.nlm.nih.gov/entrez/query.fcgi?cmd=search&db=gene&term=DNAJB9) | DnaJ (Hsp40) homolog, subfamily B, member 9 | **0.819** | **700.495** | 143.397 | **573.583** | 130.053 | 2.092E-02 |
| [224448_s_at](https://www.affymetrix.com/LinkServlet?probeset=224448_s_at) | [C6orf125](http://www.ncbi.nlm.nih.gov/entrez/query.fcgi?cmd=search&db=gene&term=C6orf125) | chromosome 6 open reading frame 125 | **0.819** | **159.519** | 41.250 | **130.636** | 37.218 | 4.283E-02 |
| [233642_s_at](https://www.affymetrix.com/LinkServlet?probeset=233642_s_at) | [HEATR5B](http://www.ncbi.nlm.nih.gov/entrez/query.fcgi?cmd=search&db=gene&term=HEATR5B) | HEAT repeat containing 5B | **0.819** | **987.761** | 103.773 | **809.097** | 163.298 | 1.887E-03 |
| [225621_at](https://www.affymetrix.com/LinkServlet?probeset=225621_at) | [ALG2](http://www.ncbi.nlm.nih.gov/entrez/query.fcgi?cmd=search&db=gene&term=ALG2) | asparagine-linked glycosylation 2, alpha-1,3-mannosyltransferase homolog (S. cerevisiae) | **0.820** | **635.610** | 110.100 | **521.071** | 63.637 | 4.714E-03 |
| [222582_at](https://www.affymetrix.com/LinkServlet?probeset=222582_at) | [PRKAG2](http://www.ncbi.nlm.nih.gov/entrez/query.fcgi?cmd=search&db=gene&term=PRKAG2) | protein kinase, AMP-activated, gamma 2 non-catalytic subunit | **0.820** | **224.587** | 47.193 | **184.126** | 54.770 | 3.348E-02 |
| [225033_at](https://www.affymetrix.com/LinkServlet?probeset=225033_at) | [ST3GAL1](http://www.ncbi.nlm.nih.gov/entrez/query.fcgi?cmd=search&db=gene&term=ST3GAL1) | ST3 beta-galactoside alpha-2,3-sialyltransferase 1 | **0.820** | **455.005** | 60.636 | **373.127** | 93.919 | 8.394E-03 |
| [203614_at](https://www.affymetrix.com/LinkServlet?probeset=203614_at) | [UTP14C](http://www.ncbi.nlm.nih.gov/entrez/query.fcgi?cmd=search&db=gene&term=UTP14C) | UTP14, U3 small nucleolar ribonucleoprotein, homolog C (yeast) | **0.820** | **458.020** | 65.406 | **375.602** | 69.115 | 6.596E-03 |
| [207657_x_at](https://www.affymetrix.com/LinkServlet?probeset=207657_x_at) | [TNPO1](http://www.ncbi.nlm.nih.gov/entrez/query.fcgi?cmd=search&db=gene&term=TNPO1) | transportin 1 | **0.820** | **1402.693** | 355.827 | **1150.786** | 184.762 | 4.572E-02 |
| [203090_at](https://www.affymetrix.com/LinkServlet?probeset=203090_at) | [SDF2](http://www.ncbi.nlm.nih.gov/entrez/query.fcgi?cmd=search&db=gene&term=SDF2) | stromal cell-derived factor 2 | **0.821** | **346.383** | 82.146 | **284.213** | 48.387 | 3.054E-02 |
| [214124_x_at](https://www.affymetrix.com/LinkServlet?probeset=214124_x_at) | [FGFR1OP](http://www.ncbi.nlm.nih.gov/entrez/query.fcgi?cmd=search&db=gene&term=FGFR1OP) | FGFR1 oncogene partner | **0.821** | **388.176** | 96.982 | **318.511** | 74.237 | 4.437E-02 |
| [229594_at](https://www.affymetrix.com/LinkServlet?probeset=229594_at) | [SPTY2D1](http://www.ncbi.nlm.nih.gov/entrez/query.fcgi?cmd=search&db=gene&term=SPTY2D1) | SPT2, Suppressor of Ty, domain containing 1 (S. cerevisiae) | **0.822** | **378.079** | 84.507 | **310.616** | 83.186 | 3.343E-02 |
| [226441_at](https://www.affymetrix.com/LinkServlet?probeset=226441_at) | [MAP3K2](http://www.ncbi.nlm.nih.gov/entrez/query.fcgi?cmd=search&db=gene&term=MAP3K2) | mitogen-activated protein kinase kinase kinase 2 | **0.822** | **306.994** | 59.822 | **252.301** | 54.635 | 1.514E-02 |
| [217941_s_at](https://www.affymetrix.com/LinkServlet?probeset=217941_s_at) | [ERBB2IP](http://www.ncbi.nlm.nih.gov/entrez/query.fcgi?cmd=search&db=gene&term=ERBB2IP) | erbb2 interacting protein | **0.823** | **597.613** | 115.343 | **491.584** | 108.768 | 1.265E-02 |
| [229285_at](https://www.affymetrix.com/LinkServlet?probeset=229285_at) | [RNASEL](http://www.ncbi.nlm.nih.gov/entrez/query.fcgi?cmd=search&db=gene&term=RNASEL) | ribonuclease L (2',5'-oligoisoadenylate synthetase-dependent) | **0.823** | **399.143** | 55.642 | **328.546** | 121.339 | 3.511E-02 |
| [206247_at](https://www.affymetrix.com/LinkServlet?probeset=206247_at) | [MICB](http://www.ncbi.nlm.nih.gov/entrez/query.fcgi?cmd=search&db=gene&term=MICB) | MHC class I polypeptide-related sequence B | **0.823** | **491.354** | 113.061 | **404.523** | 110.926 | 4.618E-02 |
| [212819_at](https://www.affymetrix.com/LinkServlet?probeset=212819_at) | [ASB1](http://www.ncbi.nlm.nih.gov/entrez/query.fcgi?cmd=search&db=gene&term=ASB1) | ankyrin repeat and SOCS box containing 1 | **0.824** | **211.148** | 42.990 | **173.904** | 31.847 | 3.948E-02 |
| [218319_at](https://www.affymetrix.com/LinkServlet?probeset=218319_at) | [PELI1](http://www.ncbi.nlm.nih.gov/entrez/query.fcgi?cmd=search&db=gene&term=PELI1) | pellino homolog 1 (Drosophila) | **0.824** | **325.816** | 73.937 | **268.541** | 54.931 | 4.161E-02 |
| [229317_at](https://www.affymetrix.com/LinkServlet?probeset=229317_at) | [KPNA5](http://www.ncbi.nlm.nih.gov/entrez/query.fcgi?cmd=search&db=gene&term=KPNA5) | karyopherin alpha 5 (importin alpha 6) | **0.825** | **92.680** | 17.828 | **76.432** | 32.695 | 4.268E-02 |
| [221011_s_at](https://www.affymetrix.com/LinkServlet?probeset=221011_s_at) | [LBH](http://www.ncbi.nlm.nih.gov/entrez/query.fcgi?cmd=search&db=gene&term=LBH) | limb bud and heart development homolog (mouse) | **0.825** | **722.376** | 152.854 | **595.814** | 146.376 | 2.841E-02 |
| [226430_at](https://www.affymetrix.com/LinkServlet?probeset=226430_at) | [RELL1](http://www.ncbi.nlm.nih.gov/entrez/query.fcgi?cmd=search&db=gene&term=RELL1) | RELT-like 1 | **0.826** | **509.479** | 93.489 | **420.583** | 103.883 | 2.887E-02 |
| [215270_at](https://www.affymetrix.com/LinkServlet?probeset=215270_at) | [LFNG](http://www.ncbi.nlm.nih.gov/entrez/query.fcgi?cmd=search&db=gene&term=LFNG) | LFNG O-fucosylpeptide 3-beta-N-acetylglucosaminyltransferase | **0.826** | **7.346** | 1.585 | **6.065** | 0.215 | 1.223E-02 |
| [222441_x_at](https://www.affymetrix.com/LinkServlet?probeset=222441_x_at) | [SLMO2](http://www.ncbi.nlm.nih.gov/entrez/query.fcgi?cmd=search&db=gene&term=SLMO2) | slowmo homolog 2 (Drosophila) | **0.827** | **460.754** | 98.343 | **380.938** | 31.998 | 2.397E-02 |
| [202666_s_at](https://www.affymetrix.com/LinkServlet?probeset=202666_s_at) | [ACTL6A](http://www.ncbi.nlm.nih.gov/entrez/query.fcgi?cmd=search&db=gene&term=ACTL6A) | actin-like 6A | **0.827** | **143.173** | 30.879 | **118.376** | 43.310 | 4.579E-02 |
| [225421_at](https://www.affymetrix.com/LinkServlet?probeset=225421_at) | [PM20D2](http://www.ncbi.nlm.nih.gov/entrez/query.fcgi?cmd=search&db=gene&term=PM20D2) | peptidase M20 domain containing 2 | **0.827** | **222.432** | 52.021 | **183.988** | 91.646 | 4.901E-02 |
| [203200_s_at](https://www.affymetrix.com/LinkServlet?probeset=203200_s_at) | [MTRR](http://www.ncbi.nlm.nih.gov/entrez/query.fcgi?cmd=search&db=gene&term=MTRR) | 5-methyltetrahydrofolate-homocysteine methyltransferase reductase | **0.828** | **1104.391** | 226.788 | **913.887** | 186.509 | 2.818E-02 |
| [203269_at](https://www.affymetrix.com/LinkServlet?probeset=203269_at) | [NSMAF](http://www.ncbi.nlm.nih.gov/entrez/query.fcgi?cmd=search&db=gene&term=NSMAF) | neutral sphingomyelinase (N-SMase) activation associated factor | **0.828** | **481.674** | 104.235 | **398.759** | 82.471 | 3.896E-02 |
| [238604_at](https://www.affymetrix.com/LinkServlet?probeset=238604_at) | [NA](http://www.ncbi.nlm.nih.gov/entrez/query.fcgi?cmd=search&db=gene&term=NA) | NA | **0.829** | **660.020** | 97.864 | **546.960** | 173.940 | 4.126E-02 |
| [202540_s_at](https://www.affymetrix.com/LinkServlet?probeset=202540_s_at) | [HMGCR](http://www.ncbi.nlm.nih.gov/entrez/query.fcgi?cmd=search&db=gene&term=HMGCR) | 3-hydroxy-3-methylglutaryl-CoA reductase | **0.829** | **700.438** | 97.343 | **580.544** | 103.583 | 4.189E-03 |
| [219231_at](https://www.affymetrix.com/LinkServlet?probeset=219231_at) | [TGS1](http://www.ncbi.nlm.nih.gov/entrez/query.fcgi?cmd=search&db=gene&term=TGS1) | trimethylguanosine synthase 1 | **0.829** | **561.324** | 112.608 | **465.254** | 114.263 | 3.989E-02 |
| [201833_at](https://www.affymetrix.com/LinkServlet?probeset=201833_at) | [HDAC2](http://www.ncbi.nlm.nih.gov/entrez/query.fcgi?cmd=search&db=gene&term=HDAC2) | histone deacetylase 2 | **0.829** | **860.677** | 201.864 | **713.495** | 91.630 | 2.440E-02 |
| [227973_at](https://www.affymetrix.com/LinkServlet?probeset=227973_at) | [C2orf69](http://www.ncbi.nlm.nih.gov/entrez/query.fcgi?cmd=search&db=gene&term=C2orf69) | chromosome 2 open reading frame 69 | **0.829** | **546.708** | 90.566 | **453.325** | 100.145 | 1.095E-02 |
| [222412_s_at](https://www.affymetrix.com/LinkServlet?probeset=222412_s_at) | [SSR3](http://www.ncbi.nlm.nih.gov/entrez/query.fcgi?cmd=search&db=gene&term=SSR3) | signal sequence receptor, gamma (translocon-associated protein gamma) | **0.830** | **269.240** | 50.048 | **223.390** | 73.642 | 2.630E-02 |
| [205036_at](https://www.affymetrix.com/LinkServlet?probeset=205036_at) | [LSM6](http://www.ncbi.nlm.nih.gov/entrez/query.fcgi?cmd=search&db=gene&term=LSM6) | LSM6 homolog, U6 small nuclear RNA associated (S. cerevisiae) | **0.831** | **314.912** | 56.441 | **261.667** | 70.541 | 2.755E-02 |
| [223076_s_at](https://www.affymetrix.com/LinkServlet?probeset=223076_s_at) | [NSUN2](http://www.ncbi.nlm.nih.gov/entrez/query.fcgi?cmd=search&db=gene&term=NSUN2) | NOP2/Sun domain family, member 2 | **0.831** | **845.440** | 125.867 | **702.787** | 95.862 | 2.978E-03 |
| [213853_at](https://www.affymetrix.com/LinkServlet?probeset=213853_at) | [DNAJC24](http://www.ncbi.nlm.nih.gov/entrez/query.fcgi?cmd=search&db=gene&term=DNAJC24) | DnaJ (Hsp40) homolog, subfamily C, member 24 | **0.831** | **219.312** | 38.477 | **182.339** | 63.269 | 3.637E-02 |
| [217408_at](https://www.affymetrix.com/LinkServlet?probeset=217408_at) | [MRPS18B](http://www.ncbi.nlm.nih.gov/entrez/query.fcgi?cmd=search&db=gene&term=MRPS18B) | mitochondrial ribosomal protein S18B | **0.832** | **483.266** | 114.507 | **401.931** | 68.646 | 4.234E-02 |
| [212857_x_at](https://www.affymetrix.com/LinkServlet?probeset=212857_x_at) | [SUB1](http://www.ncbi.nlm.nih.gov/entrez/query.fcgi?cmd=search&db=gene&term=SUB1) | SUB1 homolog (S. cerevisiae) | **0.832** | **1519.436** | 274.703 | **1264.101** | 190.456 | 1.503E-02 |
| [212665_at](https://www.affymetrix.com/LinkServlet?probeset=212665_at) | [TIPARP](http://www.ncbi.nlm.nih.gov/entrez/query.fcgi?cmd=search&db=gene&term=TIPARP) | TCDD-inducible poly(ADP-ribose) polymerase | **0.833** | **260.513** | 37.158 | **216.970** | 53.891 | 1.400E-02 |
| [224618_at](https://www.affymetrix.com/LinkServlet?probeset=224618_at) | [ROD1](http://www.ncbi.nlm.nih.gov/entrez/query.fcgi?cmd=search&db=gene&term=ROD1) | ROD1 regulator of differentiation 1 (S. pombe) | **0.834** | **291.343** | 61.150 | **243.063** | 60.374 | 3.606E-02 |
| [226383_at](https://www.affymetrix.com/LinkServlet?probeset=226383_at) | [C11orf46](http://www.ncbi.nlm.nih.gov/entrez/query.fcgi?cmd=search&db=gene&term=C11orf46) | chromosome 11 open reading frame 46 | **0.835** | **305.603** | 45.272 | **255.135** | 43.474 | 6.250E-03 |
| [226312_at](https://www.affymetrix.com/LinkServlet?probeset=226312_at) | [RICTOR](http://www.ncbi.nlm.nih.gov/entrez/query.fcgi?cmd=search&db=gene&term=RICTOR) | RPTOR independent companion of MTOR, complex 2 | **0.836** | **851.436** | 117.803 | **711.492** | 167.524 | 1.512E-02 |
| [217832_at](https://www.affymetrix.com/LinkServlet?probeset=217832_at) | [SYNCRIP](http://www.ncbi.nlm.nih.gov/entrez/query.fcgi?cmd=search&db=gene&term=SYNCRIP) | synaptotagmin binding, cytoplasmic RNA interacting protein | **0.837** | **715.257** | 106.351 | **598.783** | 107.541 | 9.768E-03 |
| [225501_at](https://www.affymetrix.com/LinkServlet?probeset=225501_at) | [PHF6](http://www.ncbi.nlm.nih.gov/entrez/query.fcgi?cmd=search&db=gene&term=PHF6) | PHD finger protein 6 | **0.838** | **145.755** | 26.530 | **122.076** | 44.026 | 4.547E-02 |
| [219178_at](https://www.affymetrix.com/LinkServlet?probeset=219178_at) | [QTRTD1](http://www.ncbi.nlm.nih.gov/entrez/query.fcgi?cmd=search&db=gene&term=QTRTD1) | queuine tRNA-ribosyltransferase domain containing 1 | **0.839** | **329.350** | 69.253 | **276.280** | 47.174 | 4.929E-02 |
| [226155_at](https://www.affymetrix.com/LinkServlet?probeset=226155_at) | [FAM160B1](http://www.ncbi.nlm.nih.gov/entrez/query.fcgi?cmd=search&db=gene&term=FAM160B1) | family with sequence similarity 160, member B1 | **0.839** | **1096.917** | 232.759 | **920.449** | 158.469 | 4.629E-02 |
| [211747_s_at](https://www.affymetrix.com/LinkServlet?probeset=211747_s_at) | [LSM5](http://www.ncbi.nlm.nih.gov/entrez/query.fcgi?cmd=search&db=gene&term=LSM5) | LSM5 homolog, U6 small nuclear RNA associated (S. cerevisiae) | **0.840** | **605.542** | 106.371 | **508.550** | 130.308 | 4.856E-02 |
| [227319_at](https://www.affymetrix.com/LinkServlet?probeset=227319_at) | [NA](http://www.ncbi.nlm.nih.gov/entrez/query.fcgi?cmd=search&db=gene&term=NA) | NA | **0.840** | **494.226** | 109.021 | **415.336** | 114.762 | 4.659E-02 |
| [215691_x_at](https://www.affymetrix.com/LinkServlet?probeset=215691_x_at) | [HSPB11](http://www.ncbi.nlm.nih.gov/entrez/query.fcgi?cmd=search&db=gene&term=HSPB11) | heat shock protein family B (small), member 11 | **0.841** | **874.936** | 149.158 | **735.771** | 158.726 | 2.263E-02 |
| [230078_at](https://www.affymetrix.com/LinkServlet?probeset=230078_at) | [RAPGEF6](http://www.ncbi.nlm.nih.gov/entrez/query.fcgi?cmd=search&db=gene&term=RAPGEF6) | Rap guanine nucleotide exchange factor (GEF) 6 | **0.841** | **582.227** | 94.587 | **489.745** | 98.841 | 2.025E-02 |
| [203428_s_at](https://www.affymetrix.com/LinkServlet?probeset=203428_s_at) | [ASF1A](http://www.ncbi.nlm.nih.gov/entrez/query.fcgi?cmd=search&db=gene&term=ASF1A) | ASF1 anti-silencing function 1 homolog A (S. cerevisiae) | **0.841** | **419.122** | 88.554 | **352.645** | 51.440 | 3.463E-02 |
| [209798_at](https://www.affymetrix.com/LinkServlet?probeset=209798_at) | [NPAT](http://www.ncbi.nlm.nih.gov/entrez/query.fcgi?cmd=search&db=gene&term=NPAT) | nuclear protein, ataxia-telangiectasia locus | **0.842** | **595.464** | 86.012 | **501.205** | 95.353 | 1.017E-02 |
| [217774_s_at](https://www.affymetrix.com/LinkServlet?probeset=217774_s_at) | [TRMT112](http://www.ncbi.nlm.nih.gov/entrez/query.fcgi?cmd=search&db=gene&term=TRMT112) | tRNA methyltransferase 11-2 homolog (S. cerevisiae) | **0.842** | **1235.464** | 197.198 | **1039.940** | 289.542 | 3.052E-02 |
| [225216_at](https://www.affymetrix.com/LinkServlet?probeset=225216_at) | [FAM199X](http://www.ncbi.nlm.nih.gov/entrez/query.fcgi?cmd=search&db=gene&term=FAM199X) | family with sequence similarity 199, X-linked | **0.842** | **476.035** | 89.579 | **400.940** | 103.256 | 4.301E-02 |
| [222991_s_at](https://www.affymetrix.com/LinkServlet?probeset=222991_s_at) | [UBQLN1](http://www.ncbi.nlm.nih.gov/entrez/query.fcgi?cmd=search&db=gene&term=UBQLN1) | ubiquilin 1 | **0.843** | **573.318** | 98.760 | **483.329** | 67.224 | 1.621E-02 |
| [238653_at](https://www.affymetrix.com/LinkServlet?probeset=238653_at) | [LRIG2](http://www.ncbi.nlm.nih.gov/entrez/query.fcgi?cmd=search&db=gene&term=LRIG2) | leucine-rich repeats and immunoglobulin-like domains 2 | **0.844** | **667.769** | 103.310 | **563.286** | 145.069 | 4.050E-02 |
| [201653_at](https://www.affymetrix.com/LinkServlet?probeset=201653_at) | [CNIH](http://www.ncbi.nlm.nih.gov/entrez/query.fcgi?cmd=search&db=gene&term=CNIH) | cornichon homolog (Drosophila) | **0.844** | **429.896** | 74.487 | **362.690** | 72.904 | 2.244E-02 |
| [241152_at](https://www.affymetrix.com/LinkServlet?probeset=241152_at) | [NA](http://www.ncbi.nlm.nih.gov/entrez/query.fcgi?cmd=search&db=gene&term=NA) | NA | **0.844** | **7.110** | 1.464 | **6.000** | 0.000 | 1.725E-02 |
| [213039_at](https://www.affymetrix.com/LinkServlet?probeset=213039_at) | [ARHGEF18](http://www.ncbi.nlm.nih.gov/entrez/query.fcgi?cmd=search&db=gene&term=ARHGEF18) | Rho/Rac guanine nucleotide exchange factor (GEF) 18 | **0.844** | **1753.713** | 260.520 | **1480.753** | 228.290 | 8.344E-03 |
| [203583_at](https://www.affymetrix.com/LinkServlet?probeset=203583_at) | [UNC50](http://www.ncbi.nlm.nih.gov/entrez/query.fcgi?cmd=search&db=gene&term=UNC50) | unc-50 homolog (C. elegans) | **0.845** | **568.221** | 81.959 | **480.250** | 108.674 | 1.779E-02 |
| [202531_at](https://www.affymetrix.com/LinkServlet?probeset=202531_at) | [IRF1](http://www.ncbi.nlm.nih.gov/entrez/query.fcgi?cmd=search&db=gene&term=IRF1) | interferon regulatory factor 1 | **0.846** | **468.532** | 85.175 | **396.460** | 72.266 | 2.960E-02 |
| [212684_at](https://www.affymetrix.com/LinkServlet?probeset=212684_at) | [ZNF3](http://www.ncbi.nlm.nih.gov/entrez/query.fcgi?cmd=search&db=gene&term=ZNF3) | zinc finger protein 3 | **0.846** | **155.299** | 32.088 | **131.415** | 38.580 | 4.274E-02 |
| [212760_at](https://www.affymetrix.com/LinkServlet?probeset=212760_at) | [UBR2](http://www.ncbi.nlm.nih.gov/entrez/query.fcgi?cmd=search&db=gene&term=UBR2) | ubiquitin protein ligase E3 component n-recognin 2 | **0.846** | **847.331** | 127.865 | **717.213** | 122.134 | 1.362E-02 |
| [213291_s_at](https://www.affymetrix.com/LinkServlet?probeset=213291_s_at) | [UBE3A](http://www.ncbi.nlm.nih.gov/entrez/query.fcgi?cmd=search&db=gene&term=UBE3A) | ubiquitin protein ligase E3A | **0.847** | **582.712** | 73.624 | **493.392** | 133.191 | 2.097E-02 |
| [211967_at](https://www.affymetrix.com/LinkServlet?probeset=211967_at) | [TMEM123](http://www.ncbi.nlm.nih.gov/entrez/query.fcgi?cmd=search&db=gene&term=TMEM123) | transmembrane protein 123 | **0.847** | **826.125** | 132.671 | **699.709** | 133.602 | 2.041E-02 |
| [209927_s_at](https://www.affymetrix.com/LinkServlet?probeset=209927_s_at) | [C1orf77](http://www.ncbi.nlm.nih.gov/entrez/query.fcgi?cmd=search&db=gene&term=C1orf77) | chromosome 1 open reading frame 77 | **0.847** | **227.874** | 35.395 | **193.027** | 61.548 | 4.354E-02 |
| [226883_at](https://www.affymetrix.com/LinkServlet?probeset=226883_at) | [NA](http://www.ncbi.nlm.nih.gov/entrez/query.fcgi?cmd=search&db=gene&term=NA) | NA | **0.847** | **851.210** | 158.882 | **721.170** | 127.518 | 3.844E-02 |
| [203376_at](https://www.affymetrix.com/LinkServlet?probeset=203376_at) | [CDC40](http://www.ncbi.nlm.nih.gov/entrez/query.fcgi?cmd=search&db=gene&term=CDC40) | cell division cycle 40 homolog (S. cerevisiae) | **0.847** | **423.426** | 72.634 | **358.810** | 58.856 | 1.673E-02 |
| [224833_at](https://www.affymetrix.com/LinkServlet?probeset=224833_at) | [ETS1](http://www.ncbi.nlm.nih.gov/entrez/query.fcgi?cmd=search&db=gene&term=ETS1) | v-ets erythroblastosis virus E26 oncogene homolog 1 (avian) | **0.847** | **1896.138** | 299.334 | **1606.850** | 226.486 | 1.199E-02 |
| [219290_x_at](https://www.affymetrix.com/LinkServlet?probeset=219290_x_at) | [DAPP1](http://www.ncbi.nlm.nih.gov/entrez/query.fcgi?cmd=search&db=gene&term=DAPP1) | dual adaptor of phosphotyrosine and 3-phosphoinositides | **0.848** | **156.947** | 27.642 | **133.063** | 17.057 | 2.216E-02 |
| [225889_at](https://www.affymetrix.com/LinkServlet?probeset=225889_at) | [AEBP2](http://www.ncbi.nlm.nih.gov/entrez/query.fcgi?cmd=search&db=gene&term=AEBP2) | AE binding protein 2 | **0.848** | **334.828** | 47.427 | **284.034** | 66.493 | 1.956E-02 |
| [210093_s_at](https://www.affymetrix.com/LinkServlet?probeset=210093_s_at) | [MAGOH](http://www.ncbi.nlm.nih.gov/entrez/query.fcgi?cmd=search&db=gene&term=MAGOH) | mago-nashi homolog, proliferation-associated (Drosophila) | **0.850** | **510.065** | 81.957 | **433.482** | 92.461 | 2.472E-02 |
| [214626_s_at](https://www.affymetrix.com/LinkServlet?probeset=214626_s_at) | [GANAB](http://www.ncbi.nlm.nih.gov/entrez/query.fcgi?cmd=search&db=gene&term=GANAB) | glucosidase, alpha; neutral AB | **0.851** | **341.581** | 36.608 | **290.569** | 52.057 | 5.864E-03 |
| [218194_at](https://www.affymetrix.com/LinkServlet?probeset=218194_at) | [REXO2](http://www.ncbi.nlm.nih.gov/entrez/query.fcgi?cmd=search&db=gene&term=REXO2) | REX2, RNA exonuclease 2 homolog (S. cerevisiae) | **0.851** | **827.792** | 117.165 | **704.178** | 117.246 | 1.316E-02 |
| [225068_at](https://www.affymetrix.com/LinkServlet?probeset=225068_at) | [KLHL12](http://www.ncbi.nlm.nih.gov/entrez/query.fcgi?cmd=search&db=gene&term=KLHL12) | kelch-like 12 (Drosophila) | **0.851** | **340.332** | 62.065 | **289.542** | 38.771 | 2.535E-02 |
| [225780_at](https://www.affymetrix.com/LinkServlet?probeset=225780_at) | [RSC1A1](http://www.ncbi.nlm.nih.gov/entrez/query.fcgi?cmd=search&db=gene&term=RSC1A1) | regulatory solute carrier protein, family 1, member 1 | **0.851** | **542.629** | 91.049 | **461.794** | 118.692 | 3.627E-02 |
| [227947_at](https://www.affymetrix.com/LinkServlet?probeset=227947_at) | [PHACTR2](http://www.ncbi.nlm.nih.gov/entrez/query.fcgi?cmd=search&db=gene&term=PHACTR2) | phosphatase and actin regulator 2 | **0.851** | **350.345** | 49.869 | **298.169** | 94.797 | 3.878E-02 |
| [204658_at](https://www.affymetrix.com/LinkServlet?probeset=204658_at) | [TRA2A](http://www.ncbi.nlm.nih.gov/entrez/query.fcgi?cmd=search&db=gene&term=TRA2A) | transformer 2 alpha homolog (Drosophila) | **0.851** | **1053.478** | 153.690 | **896.587** | 191.668 | 1.786E-02 |
| [227900_at](https://www.affymetrix.com/LinkServlet?probeset=227900_at) | [CBLB](http://www.ncbi.nlm.nih.gov/entrez/query.fcgi?cmd=search&db=gene&term=CBLB) | Cas-Br-M (murine) ecotropic retroviral transforming sequence b | **0.852** | **429.354** | 80.065 | **365.611** | 70.441 | 3.714E-02 |
| [212244_at](https://www.affymetrix.com/LinkServlet?probeset=212244_at) | [NA](http://www.ncbi.nlm.nih.gov/entrez/query.fcgi?cmd=search&db=gene&term=NA) | NA | **0.853** | **477.930** | 76.397 | **407.656** | 74.226 | 3.125E-02 |
| [216689_x_at](https://www.affymetrix.com/LinkServlet?probeset=216689_x_at) | [ARHGAP1](http://www.ncbi.nlm.nih.gov/entrez/query.fcgi?cmd=search&db=gene&term=ARHGAP1) | Rho GTPase activating protein 1 | **0.853** | **7.070** | 1.509 | **6.033** | 0.108 | 2.561E-02 |
| [222811_at](https://www.affymetrix.com/LinkServlet?probeset=222811_at) | [FTSJD1](http://www.ncbi.nlm.nih.gov/entrez/query.fcgi?cmd=search&db=gene&term=FTSJD1) | FtsJ methyltransferase domain containing 1 | **0.854** | **466.333** | 71.523 | **398.065** | 74.834 | 1.863E-02 |
| [200617_at](https://www.affymetrix.com/LinkServlet?probeset=200617_at) | [MLEC](http://www.ncbi.nlm.nih.gov/entrez/query.fcgi?cmd=search&db=gene&term=MLEC) | malectin | **0.854** | **519.615** | 91.679 | **443.684** | 107.351 | 3.821E-02 |
| [212209_at](https://www.affymetrix.com/LinkServlet?probeset=212209_at) | [MED13L](http://www.ncbi.nlm.nih.gov/entrez/query.fcgi?cmd=search&db=gene&term=MED13L) | mediator complex subunit 13-like | **0.856** | **638.781** | 71.133 | **546.728** | 89.458 | 5.089E-03 |
| [226280_at](https://www.affymetrix.com/LinkServlet?probeset=226280_at) | [NA](http://www.ncbi.nlm.nih.gov/entrez/query.fcgi?cmd=search&db=gene&term=NA) | NA | **0.857** | **675.261** | 94.568 | **578.414** | 94.092 | 1.121E-02 |
| [209111_at](https://www.affymetrix.com/LinkServlet?probeset=209111_at) | [RNF5](http://www.ncbi.nlm.nih.gov/entrez/query.fcgi?cmd=search&db=gene&term=RNF5) | ring finger protein 5 | **0.857** | **600.025** | 112.684 | **514.256** | 57.377 | 4.690E-02 |
| [225644_at](https://www.affymetrix.com/LinkServlet?probeset=225644_at) | [CCDC117](http://www.ncbi.nlm.nih.gov/entrez/query.fcgi?cmd=search&db=gene&term=CCDC117) | coiled-coil domain containing 117 | **0.858** | **696.015** | 138.200 | **597.238** | 87.801 | 4.620E-02 |
| [214683_s_at](https://www.affymetrix.com/LinkServlet?probeset=214683_s_at) | [CLK1](http://www.ncbi.nlm.nih.gov/entrez/query.fcgi?cmd=search&db=gene&term=CLK1) | CDC-like kinase 1 | **0.860** | **1562.587** | 277.990 | **1343.547** | 211.527 | 2.833E-02 |
| [212222_at](https://www.affymetrix.com/LinkServlet?probeset=212222_at) | [PSME4](http://www.ncbi.nlm.nih.gov/entrez/query.fcgi?cmd=search&db=gene&term=PSME4) | proteasome (prosome, macropain) activator subunit 4 | **0.860** | **669.139** | 119.511 | **575.563** | 92.069 | 4.130E-02 |
| [212812_at](https://www.affymetrix.com/LinkServlet?probeset=212812_at) | [SERINC5](http://www.ncbi.nlm.nih.gov/entrez/query.fcgi?cmd=search&db=gene&term=SERINC5) | serine incorporator 5 | **0.861** | **811.602** | 122.887 | **698.421** | 93.760 | 2.113E-02 |
| [201385_at](https://www.affymetrix.com/LinkServlet?probeset=201385_at) | [DHX15](http://www.ncbi.nlm.nih.gov/entrez/query.fcgi?cmd=search&db=gene&term=DHX15) | DEAH (Asp-Glu-Ala-His) box polypeptide 15 | **0.861** | **681.941** | 98.942 | **587.444** | 103.085 | 1.699E-02 |
| [223590_at](https://www.affymetrix.com/LinkServlet?probeset=223590_at) | [ZNF700](http://www.ncbi.nlm.nih.gov/entrez/query.fcgi?cmd=search&db=gene&term=ZNF700) | zinc finger protein 700 | **0.863** | **340.696** | 56.584 | **293.859** | 75.256 | 4.946E-02 |
| [209509_s_at](https://www.affymetrix.com/LinkServlet?probeset=209509_s_at) | [DPAGT1](http://www.ncbi.nlm.nih.gov/entrez/query.fcgi?cmd=search&db=gene&term=DPAGT1) | dolichyl-phosphate (UDP-N-acetylglucosamine) N-acetylglucosaminephosphotransferase 1 (GlcNAc-1-P transferase) | **0.865** | **541.808** | 93.062 | **468.649** | 60.843 | 3.107E-02 |
| [201877_s_at](https://www.affymetrix.com/LinkServlet?probeset=201877_s_at) | [PPP2R5C](http://www.ncbi.nlm.nih.gov/entrez/query.fcgi?cmd=search&db=gene&term=PPP2R5C) | protein phosphatase 2, regulatory subunit B', gamma | **0.866** | **821.816** | 138.160 | **711.883** | 104.934 | 3.474E-02 |
| [203635_at](https://www.affymetrix.com/LinkServlet?probeset=203635_at) | [DSCR3](http://www.ncbi.nlm.nih.gov/entrez/query.fcgi?cmd=search&db=gene&term=DSCR3) | Down syndrome critical region gene 3 | **0.866** | **384.604** | 71.256 | **333.196** | 34.640 | 4.040E-02 |
| [225917_at](https://www.affymetrix.com/LinkServlet?probeset=225917_at) | [NA](http://www.ncbi.nlm.nih.gov/entrez/query.fcgi?cmd=search&db=gene&term=NA) | NA | **0.867** | **971.992** | 137.427 | **842.511** | 141.826 | 2.597E-02 |
| [213074_at](https://www.affymetrix.com/LinkServlet?probeset=213074_at) | [PHIP](http://www.ncbi.nlm.nih.gov/entrez/query.fcgi?cmd=search&db=gene&term=PHIP) | pleckstrin homology domain interacting protein | **0.868** | **1450.787** | 237.562 | **1258.835** | 206.548 | 3.979E-02 |
| [203020_at](https://www.affymetrix.com/LinkServlet?probeset=203020_at) | [RABGAP1L](http://www.ncbi.nlm.nih.gov/entrez/query.fcgi?cmd=search&db=gene&term=RABGAP1L) | RAB GTPase activating protein 1-like | **0.868** | **807.966** | 101.062 | **701.077** | 146.415 | 2.592E-02 |
| [212109_at](https://www.affymetrix.com/LinkServlet?probeset=212109_at) | [HN1L](http://www.ncbi.nlm.nih.gov/entrez/query.fcgi?cmd=search&db=gene&term=HN1L) | hematological and neurological expressed 1-like | **0.868** | **552.132** | 92.014 | **479.157** | 82.707 | 4.463E-02 |
| [232024_at](https://www.affymetrix.com/LinkServlet?probeset=232024_at) | [GIMAP2](http://www.ncbi.nlm.nih.gov/entrez/query.fcgi?cmd=search&db=gene&term=GIMAP2) | GTPase, IMAP family member 2 | **0.868** | **921.853** | 160.319 | **800.603** | 61.731 | 2.043E-02 |
| [225281_at](https://www.affymetrix.com/LinkServlet?probeset=225281_at) | [C3orf17](http://www.ncbi.nlm.nih.gov/entrez/query.fcgi?cmd=search&db=gene&term=C3orf17) | chromosome 3 open reading frame 17 | **0.872** | **865.629** | 98.375 | **755.046** | 110.872 | 1.194E-02 |
| [1563250_at](https://www.affymetrix.com/LinkServlet?probeset=1563250_at) | [NA](http://www.ncbi.nlm.nih.gov/entrez/query.fcgi?cmd=search&db=gene&term=NA) | NA | **0.872** | **6.941** | 1.405 | **6.056** | 0.184 | 4.755E-02 |
| [203739_at](https://www.affymetrix.com/LinkServlet?probeset=203739_at) | [ZNF217](http://www.ncbi.nlm.nih.gov/entrez/query.fcgi?cmd=search&db=gene&term=ZNF217) | zinc finger protein 217 | **0.873** | **969.040** | 92.786 | **846.254** | 130.909 | 6.106E-03 |
| [224701_at](https://www.affymetrix.com/LinkServlet?probeset=224701_at) | [PARP14](http://www.ncbi.nlm.nih.gov/entrez/query.fcgi?cmd=search&db=gene&term=PARP14) | poly (ADP-ribose) polymerase family, member 14 | **0.874** | **749.564** | 71.449 | **654.813** | 102.997 | 7.436E-03 |
| [212846_at](https://www.affymetrix.com/LinkServlet?probeset=212846_at) | [RRP1B](http://www.ncbi.nlm.nih.gov/entrez/query.fcgi?cmd=search&db=gene&term=RRP1B) | ribosomal RNA processing 1 homolog B (S. cerevisiae) | **0.875** | **821.849** | 103.124 | **719.065** | 120.392 | 1.666E-02 |
| [203302_at](https://www.affymetrix.com/LinkServlet?probeset=203302_at) | [DCK](http://www.ncbi.nlm.nih.gov/entrez/query.fcgi?cmd=search&db=gene&term=DCK) | deoxycytidine kinase | **0.876** | **831.741** | 112.950 | **728.653** | 117.053 | 3.563E-02 |
| [215884_s_at](https://www.affymetrix.com/LinkServlet?probeset=215884_s_at) | [UBQLN2](http://www.ncbi.nlm.nih.gov/entrez/query.fcgi?cmd=search&db=gene&term=UBQLN2) | ubiquilin 2 | **0.877** | **1130.784** | 188.965 | **992.183** | 95.626 | 3.480E-02 |
| [202515_at](https://www.affymetrix.com/LinkServlet?probeset=202515_at) | [DLG1](http://www.ncbi.nlm.nih.gov/entrez/query.fcgi?cmd=search&db=gene&term=DLG1) | discs, large homolog 1 (Drosophila) | **0.877** | **599.703** | 85.089 | **526.237** | 101.380 | 3.936E-02 |
| [225563_at](https://www.affymetrix.com/LinkServlet?probeset=225563_at) | [PAN3](http://www.ncbi.nlm.nih.gov/entrez/query.fcgi?cmd=search&db=gene&term=PAN3) | PAN3 poly(A) specific ribonuclease subunit homolog (S. cerevisiae) | **0.878** | **1031.809** | 153.896 | **906.411** | 91.194 | 2.511E-02 |
| [212635_at](https://www.affymetrix.com/LinkServlet?probeset=212635_at) | [TNPO1](http://www.ncbi.nlm.nih.gov/entrez/query.fcgi?cmd=search&db=gene&term=TNPO1) | transportin 1 | **0.878** | **974.343** | 145.043 | **855.947** | 105.776 | 1.963E-02 |
| [202401_s_at](https://www.affymetrix.com/LinkServlet?probeset=202401_s_at) | [SRF](http://www.ncbi.nlm.nih.gov/entrez/query.fcgi?cmd=search&db=gene&term=SRF) | serum response factor (c-fos serum response element-binding transcription factor) | **0.879** | **320.404** | 53.703 | **281.565** | 48.008 | 4.820E-02 |
| [219777_at](https://www.affymetrix.com/LinkServlet?probeset=219777_at) | [GIMAP6](http://www.ncbi.nlm.nih.gov/entrez/query.fcgi?cmd=search&db=gene&term=GIMAP6) | GTPase, IMAP family member 6 | **0.879** | **1107.754** | 155.960 | **973.834** | 148.978 | 2.895E-02 |
| [227452_at](https://www.affymetrix.com/LinkServlet?probeset=227452_at) | [LOC100499467](http://www.ncbi.nlm.nih.gov/entrez/query.fcgi?cmd=search&db=gene&term=LOC100499467) | hypothetical LOC100499467 | **0.879** | **6.824** | 1.262 | **6.000** | 0.000 | 3.807E-02 |
| [211686_s_at](https://www.affymetrix.com/LinkServlet?probeset=211686_s_at) | [MAK16](http://www.ncbi.nlm.nih.gov/entrez/query.fcgi?cmd=search&db=gene&term=MAK16) | MAK16 homolog (S. cerevisiae) | **0.880** | **466.176** | 61.914 | **410.033** | 77.911 | 4.609E-02 |
| [212919_at](https://www.affymetrix.com/LinkServlet?probeset=212919_at) | [DCP2](http://www.ncbi.nlm.nih.gov/entrez/query.fcgi?cmd=search&db=gene&term=DCP2) | DCP2 decapping enzyme homolog (S. cerevisiae) | **0.881** | **851.925** | 118.536 | **750.515** | 108.449 | 2.332E-02 |
| [224844_at](https://www.affymetrix.com/LinkServlet?probeset=224844_at) | [SLAIN2](http://www.ncbi.nlm.nih.gov/entrez/query.fcgi?cmd=search&db=gene&term=SLAIN2) | SLAIN motif family, member 2 | **0.881** | **604.141** | 68.132 | **532.432** | 61.979 | 9.299E-03 |
| [209337_at](https://www.affymetrix.com/LinkServlet?probeset=209337_at) | [PSIP1](http://www.ncbi.nlm.nih.gov/entrez/query.fcgi?cmd=search&db=gene&term=PSIP1) | PC4 and SFRS1 interacting protein 1 | **0.883** | **1061.410** | 146.827 | **937.537** | 102.188 | 1.536E-02 |
| [234736_at](https://www.affymetrix.com/LinkServlet?probeset=234736_at) | [NA](http://www.ncbi.nlm.nih.gov/entrez/query.fcgi?cmd=search&db=gene&term=NA) | NA | **0.883** | **6.805** | 1.156 | **6.012** | 0.039 | 2.854E-02 |
| [211987_at](https://www.affymetrix.com/LinkServlet?probeset=211987_at) | [TOP2B](http://www.ncbi.nlm.nih.gov/entrez/query.fcgi?cmd=search&db=gene&term=TOP2B) | topoisomerase (DNA) II beta 180kDa | **0.883** | **921.568** | 122.176 | **814.195** | 64.478 | 1.428E-02 |
| [202683_s_at](https://www.affymetrix.com/LinkServlet?probeset=202683_s_at) | [RNMT](http://www.ncbi.nlm.nih.gov/entrez/query.fcgi?cmd=search&db=gene&term=RNMT) | RNA (guanine-7-) methyltransferase | **0.885** | **371.002** | 41.108 | **328.395** | 58.248 | 3.180E-02 |
| [1559584_a_at](https://www.affymetrix.com/LinkServlet?probeset=1559584_a_at) | [C16orf54](http://www.ncbi.nlm.nih.gov/entrez/query.fcgi?cmd=search&db=gene&term=C16orf54) | chromosome 16 open reading frame 54 | **0.886** | **1408.066** | 183.586 | **1247.141** | 181.206 | 3.523E-02 |
| [212440_at](https://www.affymetrix.com/LinkServlet?probeset=212440_at) | [SNRNP27](http://www.ncbi.nlm.nih.gov/entrez/query.fcgi?cmd=search&db=gene&term=SNRNP27) | small nuclear ribonucleoprotein 27kDa (U4/U6.U5) | **0.886** | **708.116** | 106.304 | **627.467** | 66.061 | 3.584E-02 |
| [227413_at](https://www.affymetrix.com/LinkServlet?probeset=227413_at) | [UBLCP1](http://www.ncbi.nlm.nih.gov/entrez/query.fcgi?cmd=search&db=gene&term=UBLCP1) | ubiquitin-like domain containing CTD phosphatase 1 | **0.887** | **1032.840** | 129.073 | **916.377** | 103.988 | 1.916E-02 |
| [226326_at](https://www.affymetrix.com/LinkServlet?probeset=226326_at) | [PCGF5](http://www.ncbi.nlm.nih.gov/entrez/query.fcgi?cmd=search&db=gene&term=PCGF5) | polycomb group ring finger 5 | **0.888** | **766.520** | 97.641 | **680.802** | 119.488 | 4.853E-02 |
| [200610_s_at](https://www.affymetrix.com/LinkServlet?probeset=200610_s_at) | [NCL](http://www.ncbi.nlm.nih.gov/entrez/query.fcgi?cmd=search&db=gene&term=NCL) | nucleolin | **0.888** | **839.836** | 88.803 | **745.968** | 79.860 | 9.131E-03 |
| [202951_at](https://www.affymetrix.com/LinkServlet?probeset=202951_at) | [STK38](http://www.ncbi.nlm.nih.gov/entrez/query.fcgi?cmd=search&db=gene&term=STK38) | serine/threonine kinase 38 | **0.890** | **1327.539** | 171.481 | **1181.608** | 121.242 | 1.771E-02 |
| [227075_at](https://www.affymetrix.com/LinkServlet?probeset=227075_at) | [ELP3](http://www.ncbi.nlm.nih.gov/entrez/query.fcgi?cmd=search&db=gene&term=ELP3) | elongation protein 3 homolog (S. cerevisiae) | **0.894** | **1031.699** | 122.305 | **921.946** | 142.311 | 3.989E-02 |
| [38892_at](https://www.affymetrix.com/LinkServlet?probeset=38892_at) | [KIAA0240](http://www.ncbi.nlm.nih.gov/entrez/query.fcgi?cmd=search&db=gene&term=KIAA0240) | KIAA0240 | **0.896** | **506.655** | 40.607 | **453.922** | 90.869 | 3.388E-02 |
| [215070_x_at](https://www.affymetrix.com/LinkServlet?probeset=215070_x_at) | [RABGAP1](http://www.ncbi.nlm.nih.gov/entrez/query.fcgi?cmd=search&db=gene&term=RABGAP1) | RAB GTPase activating protein 1 | **0.897** | **6.688** | 0.990 | **6.000** | 0.000 | 2.822E-02 |
| [208863_s_at](https://www.affymetrix.com/LinkServlet?probeset=208863_s_at) | [SRSF1](http://www.ncbi.nlm.nih.gov/entrez/query.fcgi?cmd=search&db=gene&term=SRSF1) | serine/arginine-rich splicing factor 1 | **0.902** | **1354.633** | 135.055 | **1222.113** | 64.138 | 6.081E-03 |
| [212588_at](https://www.affymetrix.com/LinkServlet?probeset=212588_at) | [PTPRC](http://www.ncbi.nlm.nih.gov/entrez/query.fcgi?cmd=search&db=gene&term=PTPRC) | protein tyrosine phosphatase, receptor type, C | **0.903** | **1107.937** | 120.150 | **1000.283** | 149.738 | 4.098E-02 |
| [225583_at](https://www.affymetrix.com/LinkServlet?probeset=225583_at) | [UXS1](http://www.ncbi.nlm.nih.gov/entrez/query.fcgi?cmd=search&db=gene&term=UXS1) | UDP-glucuronate decarboxylase 1 | **0.908** | **1071.301** | 122.408 | **973.230** | 93.801 | 3.609E-02 |
| [1552854_a_at](https://www.affymetrix.com/LinkServlet?probeset=1552854_a_at) | [VWA5B1](http://www.ncbi.nlm.nih.gov/entrez/query.fcgi?cmd=search&db=gene&term=VWA5B1) | von Willebrand factor A domain containing 5B1 | **0.911** | **6.587** | 0.868 | **6.000** | 0.000 | 3.010E-02 |
| [231590_at](https://www.affymetrix.com/LinkServlet?probeset=231590_at) | [GATM](http://www.ncbi.nlm.nih.gov/entrez/query.fcgi?cmd=search&db=gene&term=GATM) | glycine amidinotransferase (L-arginine:glycine amidinotransferase) | **1.010** | **6.000** | 0.000 | **6.059** | 0.116 | 4.925E-02 |
| [1561710_at](https://www.affymetrix.com/LinkServlet?probeset=1561710_at) | [SNX19](http://www.ncbi.nlm.nih.gov/entrez/query.fcgi?cmd=search&db=gene&term=SNX19) | sorting nexin 19 | **1.030** | **6.000** | 0.000 | **6.182** | 0.356 | 4.724E-02 |
| [234992_x_at](https://www.affymetrix.com/LinkServlet?probeset=234992_x_at) | [ECT2](http://www.ncbi.nlm.nih.gov/entrez/query.fcgi?cmd=search&db=gene&term=ECT2) | epithelial cell transforming sequence 2 oncogene | **1.038** | **6.000** | 0.000 | **6.229** | 0.436 | 4.328E-02 |
| [220676_at](https://www.affymetrix.com/LinkServlet?probeset=220676_at) | [ADAMTS8](http://www.ncbi.nlm.nih.gov/entrez/query.fcgi?cmd=search&db=gene&term=ADAMTS8) | ADAM metallopeptidase with thrombospondin type 1 motif, 8 | **1.039** | **6.000** | 0.000 | **6.233** | 0.444 | 4.164E-02 |
| [237152_at](https://www.affymetrix.com/LinkServlet?probeset=237152_at) | [PCDP1](http://www.ncbi.nlm.nih.gov/entrez/query.fcgi?cmd=search&db=gene&term=PCDP1) | primary ciliary dyskinesia protein 1 | **1.040** | **6.000** | 0.000 | **6.237** | 0.420 | 3.070E-02 |
| [216487_at](https://www.affymetrix.com/LinkServlet?probeset=216487_at) | [NA](http://www.ncbi.nlm.nih.gov/entrez/query.fcgi?cmd=search&db=gene&term=NA) | NA | **1.047** | **6.000** | 0.000 | **6.285** | 0.510 | 3.262E-02 |
| [206131_at](https://www.affymetrix.com/LinkServlet?probeset=206131_at) | [CLPS](http://www.ncbi.nlm.nih.gov/entrez/query.fcgi?cmd=search&db=gene&term=CLPS) | colipase, pancreatic | **1.050** | **6.000** | 0.000 | **6.300** | 0.419 | 7.844E-03 |
| [206905_s_at](https://www.affymetrix.com/LinkServlet?probeset=206905_s_at) | [MATN1](http://www.ncbi.nlm.nih.gov/entrez/query.fcgi?cmd=search&db=gene&term=MATN1) | matrilin 1, cartilage matrix protein | **1.054** | **6.000** | 0.000 | **6.326** | 0.597 | 3.503E-02 |
| [216681_at](https://www.affymetrix.com/LinkServlet?probeset=216681_at) | [ENOX2](http://www.ncbi.nlm.nih.gov/entrez/query.fcgi?cmd=search&db=gene&term=ENOX2) | ecto-NOX disulfide-thiol exchanger 2 | **1.058** | **6.000** | 0.000 | **6.349** | 0.590 | 2.252E-02 |
| [240896_at](https://www.affymetrix.com/LinkServlet?probeset=240896_at) | [NA](http://www.ncbi.nlm.nih.gov/entrez/query.fcgi?cmd=search&db=gene&term=NA) | NA | **1.058** | **6.000** | 0.000 | **6.351** | 0.660 | 3.879E-02 |
| [210744_s_at](https://www.affymetrix.com/LinkServlet?probeset=210744_s_at) | [IL5RA](http://www.ncbi.nlm.nih.gov/entrez/query.fcgi?cmd=search&db=gene&term=IL5RA) | interleukin 5 receptor, alpha | **1.059** | **6.000** | 0.000 | **6.354** | 0.663 | 3.943E-02 |
| [215478_at](https://www.affymetrix.com/LinkServlet?probeset=215478_at) | [RIMS2](http://www.ncbi.nlm.nih.gov/entrez/query.fcgi?cmd=search&db=gene&term=RIMS2) | regulating synaptic membrane exocytosis 2 | **1.062** | **6.006** | 0.025 | **6.378** | 0.669 | 3.365E-02 |
| [1562167_a_at](https://www.affymetrix.com/LinkServlet?probeset=1562167_a_at) | [DEFB122](http://www.ncbi.nlm.nih.gov/entrez/query.fcgi?cmd=search&db=gene&term=DEFB122) | defensin, beta 122 (pseudogene) | **1.062** | **6.000** | 0.000 | **6.375** | 0.553 | 1.076E-02 |
| [213973_at](https://www.affymetrix.com/LinkServlet?probeset=213973_at) | [RRBP1](http://www.ncbi.nlm.nih.gov/entrez/query.fcgi?cmd=search&db=gene&term=RRBP1) | ribosome binding protein 1 homolog 180kDa (dog) | **1.063** | **6.000** | 0.000 | **6.377** | 0.703 | 3.833E-02 |
| [203890_s_at](https://www.affymetrix.com/LinkServlet?probeset=203890_s_at) | [DAPK3](http://www.ncbi.nlm.nih.gov/entrez/query.fcgi?cmd=search&db=gene&term=DAPK3) | death-associated protein kinase 3 | **1.064** | **6.022** | 0.088 | **6.410** | 0.746 | 4.758E-02 |
| [1560300_a_at](https://www.affymetrix.com/LinkServlet?probeset=1560300_a_at) | [NA](http://www.ncbi.nlm.nih.gov/entrez/query.fcgi?cmd=search&db=gene&term=NA) | NA | **1.066** | **6.000** | 0.000 | **6.395** | 0.706 | 3.158E-02 |
| [214159_at](https://www.affymetrix.com/LinkServlet?probeset=214159_at) | [PLCE1](http://www.ncbi.nlm.nih.gov/entrez/query.fcgi?cmd=search&db=gene&term=PLCE1) | phospholipase C, epsilon 1 | **1.067** | **6.000** | 0.000 | **6.401** | 0.798 | 4.891E-02 |
| [205468_s_at](https://www.affymetrix.com/LinkServlet?probeset=205468_s_at) | [IRF5](http://www.ncbi.nlm.nih.gov/entrez/query.fcgi?cmd=search&db=gene&term=IRF5) | interferon regulatory factor 5 | **1.067** | **6.004** | 0.017 | **6.406** | 0.718 | 3.217E-02 |
| [220543_at](https://www.affymetrix.com/LinkServlet?probeset=220543_at) | [C21orf62](http://www.ncbi.nlm.nih.gov/entrez/query.fcgi?cmd=search&db=gene&term=C21orf62) | chromosome 21 open reading frame 62 | **1.069** | **6.000** | 0.000 | **6.415** | 0.726 | 2.883E-02 |
| [230903_s_at](https://www.affymetrix.com/LinkServlet?probeset=230903_s_at) | [C8orf42](http://www.ncbi.nlm.nih.gov/entrez/query.fcgi?cmd=search&db=gene&term=C8orf42) | chromosome 8 open reading frame 42 | **1.073** | **6.000** | 0.000 | **6.436** | 0.753 | 2.760E-02 |
| [224530_s_at](https://www.affymetrix.com/LinkServlet?probeset=224530_s_at) | [KCNIP4](http://www.ncbi.nlm.nih.gov/entrez/query.fcgi?cmd=search&db=gene&term=KCNIP4) | Kv channel interacting protein 4 | **1.073** | **6.006** | 0.025 | **6.446** | 0.674 | 1.327E-02 |
| [1555330_at](https://www.affymetrix.com/LinkServlet?probeset=1555330_at) | [GCLC](http://www.ncbi.nlm.nih.gov/entrez/query.fcgi?cmd=search&db=gene&term=GCLC) | glutamate-cysteine ligase, catalytic subunit | **1.075** | **6.000** | 0.000 | **6.453** | 0.888 | 4.515E-02 |
| [1561614_at](https://www.affymetrix.com/LinkServlet?probeset=1561614_at) | [SLC8A1](http://www.ncbi.nlm.nih.gov/entrez/query.fcgi?cmd=search&db=gene&term=SLC8A1) | solute carrier family 8 (sodium/calcium exchanger), member 1 | **1.076** | **6.002** | 0.008 | **6.459** | 0.837 | 3.474E-02 |
| [229148_at](https://www.affymetrix.com/LinkServlet?probeset=229148_at) | [NA](http://www.ncbi.nlm.nih.gov/entrez/query.fcgi?cmd=search&db=gene&term=NA) | NA | **1.078** | **6.000** | 0.000 | **6.468** | 0.706 | 1.020E-02 |
| [240211_at](https://www.affymetrix.com/LinkServlet?probeset=240211_at) | [LOC100130468](http://www.ncbi.nlm.nih.gov/entrez/query.fcgi?cmd=search&db=gene&term=LOC100130468) | hypothetical protein LOC100130468 | **1.082** | **6.000** | 0.000 | **6.493** | 0.689 | 6.658E-03 |
| [1554699_at](https://www.affymetrix.com/LinkServlet?probeset=1554699_at) | [L3MBTL4](http://www.ncbi.nlm.nih.gov/entrez/query.fcgi?cmd=search&db=gene&term=L3MBTL4) | l(3)mbt-like 4 (Drosophila) | **1.083** | **6.000** | 0.000 | **6.498** | 0.975 | 3.891E-02 |
| [205846_at](https://www.affymetrix.com/LinkServlet?probeset=205846_at) | [PTPRB](http://www.ncbi.nlm.nih.gov/entrez/query.fcgi?cmd=search&db=gene&term=PTPRB) | protein tyrosine phosphatase, receptor type, B | **1.083** | **6.000** | 0.000 | **6.501** | 0.792 | 1.602E-02 |
| [231700_at](https://www.affymetrix.com/LinkServlet?probeset=231700_at) | [GUCA1A](http://www.ncbi.nlm.nih.gov/entrez/query.fcgi?cmd=search&db=gene&term=GUCA1A) | guanylate cyclase activator 1A (retina) | **1.086** | **6.000** | 0.000 | **6.513** | 1.006 | 4.474E-02 |
| [205564_at](https://www.affymetrix.com/LinkServlet?probeset=205564_at) | [PAGE4](http://www.ncbi.nlm.nih.gov/entrez/query.fcgi?cmd=search&db=gene&term=PAGE4) | P antigen family, member 4 (prostate associated) | **1.089** | **6.013** | 0.052 | **6.547** | 0.979 | 3.675E-02 |
| [215661_at](https://www.affymetrix.com/LinkServlet?probeset=215661_at) | [MAST2](http://www.ncbi.nlm.nih.gov/entrez/query.fcgi?cmd=search&db=gene&term=MAST2) | microtubule associated serine/threonine kinase 2 | **1.092** | **6.000** | 0.000 | **6.553** | 0.959 | 2.803E-02 |
| [208946_s_at](https://www.affymetrix.com/LinkServlet?probeset=208946_s_at) | [BECN1](http://www.ncbi.nlm.nih.gov/entrez/query.fcgi?cmd=search&db=gene&term=BECN1) | beclin 1, autophagy related | **1.092** | **616.648** | 55.542 | **673.442** | 69.544 | 2.712E-02 |
| [202986_at](https://www.affymetrix.com/LinkServlet?probeset=202986_at) | [ARNT2](http://www.ncbi.nlm.nih.gov/entrez/query.fcgi?cmd=search&db=gene&term=ARNT2) | aryl-hydrocarbon receptor nuclear translocator 2 | **1.093** | **6.000** | 0.000 | **6.556** | 0.989 | 3.091E-02 |
| [1562910_at](https://www.affymetrix.com/LinkServlet?probeset=1562910_at) | [SH3PXD2B](http://www.ncbi.nlm.nih.gov/entrez/query.fcgi?cmd=search&db=gene&term=SH3PXD2B) | SH3 and PX domains 2B | **1.094** | **6.089** | 0.236 | **6.662** | 0.954 | 2.955E-02 |
| [233256_at](https://www.affymetrix.com/LinkServlet?probeset=233256_at) | [NA](http://www.ncbi.nlm.nih.gov/entrez/query.fcgi?cmd=search&db=gene&term=NA) | NA | **1.095** | **6.000** | 0.000 | **6.567** | 1.063 | 3.787E-02 |
| [227667_at](https://www.affymetrix.com/LinkServlet?probeset=227667_at) | [CUEDC1](http://www.ncbi.nlm.nih.gov/entrez/query.fcgi?cmd=search&db=gene&term=CUEDC1) | CUE domain containing 1 | **1.103** | **6.000** | 0.000 | **6.620** | 1.087 | 2.899E-02 |
| [237127_at](https://www.affymetrix.com/LinkServlet?probeset=237127_at) | [NA](http://www.ncbi.nlm.nih.gov/entrez/query.fcgi?cmd=search&db=gene&term=NA) | NA | **1.103** | **6.000** | 0.000 | **6.621** | 0.956 | 1.304E-02 |
| [208970_s_at](https://www.affymetrix.com/LinkServlet?probeset=208970_s_at) | [UROD](http://www.ncbi.nlm.nih.gov/entrez/query.fcgi?cmd=search&db=gene&term=UROD) | uroporphyrinogen decarboxylase | **1.105** | **400.823** | 40.630 | **442.972** | 56.411 | 3.756E-02 |
| [1554625_at](https://www.affymetrix.com/LinkServlet?probeset=1554625_at) | [BCL6B](http://www.ncbi.nlm.nih.gov/entrez/query.fcgi?cmd=search&db=gene&term=BCL6B) | B-cell CLL/lymphoma 6, member B | **1.106** | **6.000** | 0.000 | **6.637** | 1.161 | 3.413E-02 |
| [232457_at](https://www.affymetrix.com/LinkServlet?probeset=232457_at) | [LIMCH1](http://www.ncbi.nlm.nih.gov/entrez/query.fcgi?cmd=search&db=gene&term=LIMCH1) | LIM and calponin homology domains 1 | **1.107** | **6.000** | 0.000 | **6.644** | 1.037 | 1.674E-02 |
| [1561320_at](https://www.affymetrix.com/LinkServlet?probeset=1561320_at) | [NA](http://www.ncbi.nlm.nih.gov/entrez/query.fcgi?cmd=search&db=gene&term=NA) | NA | **1.108** | **6.000** | 0.000 | **6.647** | 1.032 | 1.590E-02 |
| [206229_x_at](https://www.affymetrix.com/LinkServlet?probeset=206229_x_at) | [PAX2](http://www.ncbi.nlm.nih.gov/entrez/query.fcgi?cmd=search&db=gene&term=PAX2) | paired box 2 | **1.108** | **6.029** | 0.062 | **6.680** | 1.293 | 4.337E-02 |
| [236183_at](https://www.affymetrix.com/LinkServlet?probeset=236183_at) | [DYNC1H1](http://www.ncbi.nlm.nih.gov/entrez/query.fcgi?cmd=search&db=gene&term=DYNC1H1) | dynein, cytoplasmic 1, heavy chain 1 | **1.109** | **6.000** | 0.000 | **6.654** | 1.270 | 4.080E-02 |
| [237228_at](https://www.affymetrix.com/LinkServlet?probeset=237228_at) | [ZDHHC1](http://www.ncbi.nlm.nih.gov/entrez/query.fcgi?cmd=search&db=gene&term=ZDHHC1) | zinc finger, DHHC-type containing 1 | **1.110** | **6.066** | 0.199 | **6.732** | 1.187 | 3.270E-02 |
| [208804_s_at](https://www.affymetrix.com/LinkServlet?probeset=208804_s_at) | [SRSF6](http://www.ncbi.nlm.nih.gov/entrez/query.fcgi?cmd=search&db=gene&term=SRSF6) | serine/arginine-rich splicing factor 6 | **1.111** | **1300.954** | 130.460 | **1444.918** | 152.634 | 1.634E-02 |
| [217751_at](https://www.affymetrix.com/LinkServlet?probeset=217751_at) | [GSTK1](http://www.ncbi.nlm.nih.gov/entrez/query.fcgi?cmd=search&db=gene&term=GSTK1) | glutathione S-transferase kappa 1 | **1.115** | **1670.972** | 156.406 | **1863.164** | 144.346 | 3.402E-03 |
| [207092_at](https://www.affymetrix.com/LinkServlet?probeset=207092_at) | [LEP](http://www.ncbi.nlm.nih.gov/entrez/query.fcgi?cmd=search&db=gene&term=LEP) | leptin | **1.119** | **6.000** | 0.000 | **6.715** | 1.293 | 3.265E-02 |
| [209067_s_at](https://www.affymetrix.com/LinkServlet?probeset=209067_s_at) | [HNRPDL](http://www.ncbi.nlm.nih.gov/entrez/query.fcgi?cmd=search&db=gene&term=HNRPDL) | heterogeneous nuclear ribonucleoprotein D-like | **1.121** | **1538.767** | 249.803 | **1724.237** | 191.110 | 4.676E-02 |
| [1557329_at](https://www.affymetrix.com/LinkServlet?probeset=1557329_at) | [NA](http://www.ncbi.nlm.nih.gov/entrez/query.fcgi?cmd=search&db=gene&term=NA) | NA | **1.121** | **6.000** | 0.000 | **6.725** | 1.389 | 4.166E-02 |
| [220208_at](https://www.affymetrix.com/LinkServlet?probeset=220208_at) | [ADAMTS13](http://www.ncbi.nlm.nih.gov/entrez/query.fcgi?cmd=search&db=gene&term=ADAMTS13) | ADAM metallopeptidase with thrombospondin type 1 motif, 13 | **1.122** | **6.131** | 0.229 | **6.880** | 1.092 | 1.415E-02 |
| [222298_at](https://www.affymetrix.com/LinkServlet?probeset=222298_at) | [NA](http://www.ncbi.nlm.nih.gov/entrez/query.fcgi?cmd=search&db=gene&term=NA) | NA | **1.123** | **6.000** | 0.000 | **6.738** | 1.390 | 3.675E-02 |
| [207835_at](https://www.affymetrix.com/LinkServlet?probeset=207835_at) | [FBLN1](http://www.ncbi.nlm.nih.gov/entrez/query.fcgi?cmd=search&db=gene&term=FBLN1) | fibulin 1 | **1.126** | **6.051** | 0.152 | **6.813** | 1.277 | 2.369E-02 |
| [1558579_at](https://www.affymetrix.com/LinkServlet?probeset=1558579_at) | [FLJ37786](http://www.ncbi.nlm.nih.gov/entrez/query.fcgi?cmd=search&db=gene&term=FLJ37786) | hypothetical LOC642691 | **1.127** | **6.027** | 0.105 | **6.794** | 1.329 | 2.686E-02 |
| [200774_at](https://www.affymetrix.com/LinkServlet?probeset=200774_at) | [FAM120A](http://www.ncbi.nlm.nih.gov/entrez/query.fcgi?cmd=search&db=gene&term=FAM120A) | family with sequence similarity 120A | **1.128** | **1135.882** | 107.592 | **1280.965** | 213.852 | 4.084E-02 |
| [216784_at](https://www.affymetrix.com/LinkServlet?probeset=216784_at) | [NA](http://www.ncbi.nlm.nih.gov/entrez/query.fcgi?cmd=search&db=gene&term=NA) | NA | **1.128** | **6.000** | 0.000 | **6.767** | 1.536 | 4.717E-02 |
| [223509_at](https://www.affymetrix.com/LinkServlet?probeset=223509_at) | [CLDN2](http://www.ncbi.nlm.nih.gov/entrez/query.fcgi?cmd=search&db=gene&term=CLDN2) | claudin 2 | **1.131** | **6.000** | 0.000 | **6.787** | 1.244 | 1.475E-02 |
| [201993_x_at](https://www.affymetrix.com/LinkServlet?probeset=201993_x_at) | [HNRPDL](http://www.ncbi.nlm.nih.gov/entrez/query.fcgi?cmd=search&db=gene&term=HNRPDL) | heterogeneous nuclear ribonucleoprotein D-like | **1.134** | **1322.020** | 189.111 | **1499.462** | 201.780 | 3.044E-02 |
| [1554222_at](https://www.affymetrix.com/LinkServlet?probeset=1554222_at) | [MGC45922](http://www.ncbi.nlm.nih.gov/entrez/query.fcgi?cmd=search&db=gene&term=MGC45922) | hypothetical LOC284365 | **1.138** | **6.051** | 0.203 | **6.884** | 1.516 | 3.186E-02 |
| [224874_at](https://www.affymetrix.com/LinkServlet?probeset=224874_at) | [POLR1D](http://www.ncbi.nlm.nih.gov/entrez/query.fcgi?cmd=search&db=gene&term=POLR1D) | polymerase (RNA) I polypeptide D, 16kDa | **1.140** | **361.162** | 29.037 | **411.853** | 73.065 | 2.615E-02 |
| [201600_at](https://www.affymetrix.com/LinkServlet?probeset=201600_at) | [PHB2](http://www.ncbi.nlm.nih.gov/entrez/query.fcgi?cmd=search&db=gene&term=PHB2) | prohibitin 2 | **1.140** | **889.032** | 83.048 | **1013.860** | 200.150 | 4.674E-02 |
| [225272_at](https://www.affymetrix.com/LinkServlet?probeset=225272_at) | [SAT2](http://www.ncbi.nlm.nih.gov/entrez/query.fcgi?cmd=search&db=gene&term=SAT2) | spermidine/spermine N1-acetyltransferase family member 2 | **1.141** | **463.405** | 59.033 | **528.574** | 81.911 | 2.554E-02 |
| [204846_at](https://www.affymetrix.com/LinkServlet?probeset=204846_at) | [CP](http://www.ncbi.nlm.nih.gov/entrez/query.fcgi?cmd=search&db=gene&term=CP) | ceruloplasmin (ferroxidase) | **1.142** | **6.000** | 0.000 | **6.854** | 1.641 | 3.989E-02 |
| [232501_at](https://www.affymetrix.com/LinkServlet?probeset=232501_at) | [NA](http://www.ncbi.nlm.nih.gov/entrez/query.fcgi?cmd=search&db=gene&term=NA) | NA | **1.143** | **6.025** | 0.072 | **6.889** | 1.621 | 4.001E-02 |
| [241150_at](https://www.affymetrix.com/LinkServlet?probeset=241150_at) | [SPTAN1](http://www.ncbi.nlm.nih.gov/entrez/query.fcgi?cmd=search&db=gene&term=SPTAN1) | spectrin, alpha, non-erythrocytic 1 (alpha-fodrin) | **1.145** | **6.124** | 0.285 | **7.013** | 1.680 | 4.797E-02 |
| [226128_at](https://www.affymetrix.com/LinkServlet?probeset=226128_at) | [NA](http://www.ncbi.nlm.nih.gov/entrez/query.fcgi?cmd=search&db=gene&term=NA) | NA | **1.145** | **711.854** | 101.174 | **815.330** | 139.223 | 4.931E-02 |
| [1552703_s_at](https://www.affymetrix.com/LinkServlet?probeset=1552703_s_at) | [NA](http://www.ncbi.nlm.nih.gov/entrez/query.fcgi?cmd=search&db=gene&term=NA) | NA | **1.146** | **805.540** | 130.365 | **922.802** | 145.065 | 4.708E-02 |
| [240424_s_at](https://www.affymetrix.com/LinkServlet?probeset=240424_s_at) | [LOC441204](http://www.ncbi.nlm.nih.gov/entrez/query.fcgi?cmd=search&db=gene&term=LOC441204) | hypothetical locus LOC441204 | **1.146** | **6.000** | 0.000 | **6.876** | 1.740 | 3.607E-02 |
| [241831_at](https://www.affymetrix.com/LinkServlet?probeset=241831_at) | [NA](http://www.ncbi.nlm.nih.gov/entrez/query.fcgi?cmd=search&db=gene&term=NA) | NA | **1.147** | **6.146** | 0.327 | **7.047** | 1.507 | 2.901E-02 |
| [239000_at](https://www.affymetrix.com/LinkServlet?probeset=239000_at) | [BRD4](http://www.ncbi.nlm.nih.gov/entrez/query.fcgi?cmd=search&db=gene&term=BRD4) | bromodomain containing 4 | **1.147** | **6.000** | 0.000 | **6.880** | 1.531 | 2.811E-02 |
| [218341_at](https://www.affymetrix.com/LinkServlet?probeset=218341_at) | [PPCS](http://www.ncbi.nlm.nih.gov/entrez/query.fcgi?cmd=search&db=gene&term=PPCS) | phosphopantothenoylcysteine synthetase | **1.147** | **937.454** | 139.755 | **1074.981** | 201.328 | 4.540E-02 |
| [241129_at](https://www.affymetrix.com/LinkServlet?probeset=241129_at) | [NA](http://www.ncbi.nlm.nih.gov/entrez/query.fcgi?cmd=search&db=gene&term=NA) | NA | **1.149** | **6.000** | 0.000 | **6.894** | 1.205 | 4.903E-03 |
| [201745_at](https://www.affymetrix.com/LinkServlet?probeset=201745_at) | [TWF1](http://www.ncbi.nlm.nih.gov/entrez/query.fcgi?cmd=search&db=gene&term=TWF1) | twinfilin, actin-binding protein, homolog 1 (Drosophila) | **1.151** | **298.292** | 63.677 | **343.210** | 55.366 | 4.778E-02 |
| [209762_x_at](https://www.affymetrix.com/LinkServlet?probeset=209762_x_at) | [SP110](http://www.ncbi.nlm.nih.gov/entrez/query.fcgi?cmd=search&db=gene&term=SP110) | SP110 nuclear body protein | **1.153** | **541.546** | 117.996 | **624.629** | 76.495 | 3.951E-02 |
| [220561_at](https://www.affymetrix.com/LinkServlet?probeset=220561_at) | [IGF2AS](http://www.ncbi.nlm.nih.gov/entrez/query.fcgi?cmd=search&db=gene&term=IGF2AS) | insulin-like growth factor 2 antisense | **1.155** | **6.118** | 0.467 | **7.067** | 1.524 | 2.851E-02 |
| [217765_at](https://www.affymetrix.com/LinkServlet?probeset=217765_at) | [NRBP1](http://www.ncbi.nlm.nih.gov/entrez/query.fcgi?cmd=search&db=gene&term=NRBP1) | nuclear receptor binding protein 1 | **1.155** | **201.184** | 45.330 | **232.461** | 33.782 | 4.880E-02 |
| [201284_s_at](https://www.affymetrix.com/LinkServlet?probeset=201284_s_at) | [APEH](http://www.ncbi.nlm.nih.gov/entrez/query.fcgi?cmd=search&db=gene&term=APEH) | N-acylaminoacyl-peptide hydrolase | **1.156** | **186.581** | 40.837 | **215.776** | 27.814 | 4.686E-02 |
| [1563405_at](https://www.affymetrix.com/LinkServlet?probeset=1563405_at) | [ATP4B](http://www.ncbi.nlm.nih.gov/entrez/query.fcgi?cmd=search&db=gene&term=ATP4B) | ATPase, H+/K+ exchanging, beta polypeptide | **1.157** | **6.053** | 0.116 | **7.006** | 1.635 | 2.256E-02 |
| [208990_s_at](https://www.affymetrix.com/LinkServlet?probeset=208990_s_at) | [HNRNPH3](http://www.ncbi.nlm.nih.gov/entrez/query.fcgi?cmd=search&db=gene&term=HNRNPH3) | heterogeneous nuclear ribonucleoprotein H3 (2H9) | **1.161** | **849.660** | 83.315 | **986.328** | 140.963 | 3.129E-03 |
| [217983_s_at](https://www.affymetrix.com/LinkServlet?probeset=217983_s_at) | [RNASET2](http://www.ncbi.nlm.nih.gov/entrez/query.fcgi?cmd=search&db=gene&term=RNASET2) | ribonuclease T2 | **1.163** | **1189.185** | 203.719 | **1382.968** | 253.752 | 4.228E-02 |
| [217001_x_at](https://www.affymetrix.com/LinkServlet?probeset=217001_x_at) | [HLA-DOA](http://www.ncbi.nlm.nih.gov/entrez/query.fcgi?cmd=search&db=gene&term=HLA-DOA) | major histocompatibility complex, class II, DO alpha | **1.165** | **6.000** | 0.000 | **6.988** | 1.908 | 4.185E-02 |
| [218323_at](https://www.affymetrix.com/LinkServlet?probeset=218323_at) | [RHOT1](http://www.ncbi.nlm.nih.gov/entrez/query.fcgi?cmd=search&db=gene&term=RHOT1) | ras homolog gene family, member T1 | **1.165** | **411.626** | 76.218 | **479.382** | 88.540 | 4.355E-02 |
| [225757_s_at](https://www.affymetrix.com/LinkServlet?probeset=225757_s_at) | [CLMN](http://www.ncbi.nlm.nih.gov/entrez/query.fcgi?cmd=search&db=gene&term=CLMN) | calmin (calponin-like, transmembrane) | **1.168** | **6.000** | 0.000 | **7.008** | 1.642 | 1.568E-02 |
| [1553359_at](https://www.affymetrix.com/LinkServlet?probeset=1553359_at) | [FBXL18](http://www.ncbi.nlm.nih.gov/entrez/query.fcgi?cmd=search&db=gene&term=FBXL18) | F-box and leucine-rich repeat protein 18 | **1.171** | **6.447** | 0.765 | **7.547** | 1.846 | 4.319E-02 |
| [223032_x_at](https://www.affymetrix.com/LinkServlet?probeset=223032_x_at) | [PRELID1](http://www.ncbi.nlm.nih.gov/entrez/query.fcgi?cmd=search&db=gene&term=PRELID1) | PRELI domain containing 1 | **1.174** | **817.372** | 151.507 | **959.238** | 158.692 | 2.550E-02 |
| [218153_at](https://www.affymetrix.com/LinkServlet?probeset=218153_at) | [CARS2](http://www.ncbi.nlm.nih.gov/entrez/query.fcgi?cmd=search&db=gene&term=CARS2) | cysteinyl-tRNA synthetase 2, mitochondrial (putative) | **1.174** | **233.731** | 49.976 | **274.341** | 41.421 | 3.064E-02 |
| [239112_at](https://www.affymetrix.com/LinkServlet?probeset=239112_at) | [NA](http://www.ncbi.nlm.nih.gov/entrez/query.fcgi?cmd=search&db=gene&term=NA) | NA | **1.177** | **6.000** | 0.000 | **7.063** | 1.401 | 4.279E-03 |
| [237185_at](https://www.affymetrix.com/LinkServlet?probeset=237185_at) | [NA](http://www.ncbi.nlm.nih.gov/entrez/query.fcgi?cmd=search&db=gene&term=NA) | NA | **1.178** | **6.096** | 0.383 | **7.179** | 1.519 | 1.085E-02 |
| [230900_at](https://www.affymetrix.com/LinkServlet?probeset=230900_at) | [CCDC110](http://www.ncbi.nlm.nih.gov/entrez/query.fcgi?cmd=search&db=gene&term=CCDC110) | coiled-coil domain containing 110 | **1.178** | **6.049** | 0.136 | **7.125** | 1.959 | 3.803E-02 |
| [200621_at](https://www.affymetrix.com/LinkServlet?probeset=200621_at) | [CSRP1](http://www.ncbi.nlm.nih.gov/entrez/query.fcgi?cmd=search&db=gene&term=CSRP1) | cysteine and glycine-rich protein 1 | **1.178** | **310.214** | 64.440 | **365.409** | 57.778 | 2.849E-02 |
| [1552889_a_at](https://www.affymetrix.com/LinkServlet?probeset=1552889_a_at) | [EXOC3L2](http://www.ncbi.nlm.nih.gov/entrez/query.fcgi?cmd=search&db=gene&term=EXOC3L2) | exocyst complex component 3-like 2 | **1.178** | **6.106** | 0.424 | **7.193** | 1.810 | 2.209E-02 |
| [233062_at](https://www.affymetrix.com/LinkServlet?probeset=233062_at) | [NA](http://www.ncbi.nlm.nih.gov/entrez/query.fcgi?cmd=search&db=gene&term=NA) | NA | **1.179** | **6.128** | 0.370 | **7.226** | 1.709 | 1.651E-02 |
| [1553775_at](https://www.affymetrix.com/LinkServlet?probeset=1553775_at) | [FLJ31715](http://www.ncbi.nlm.nih.gov/entrez/query.fcgi?cmd=search&db=gene&term=FLJ31715) | hypothetical protein FLJ31715 | **1.179** | **6.076** | 0.207 | **7.166** | 2.038 | 4.322E-02 |
| [222148_s_at](https://www.affymetrix.com/LinkServlet?probeset=222148_s_at) | [RHOT1](http://www.ncbi.nlm.nih.gov/entrez/query.fcgi?cmd=search&db=gene&term=RHOT1) | ras homolog gene family, member T1 | **1.181** | **356.210** | 83.972 | **420.702** | 80.087 | 4.720E-02 |
| [210968_s_at](https://www.affymetrix.com/LinkServlet?probeset=210968_s_at) | [RTN4](http://www.ncbi.nlm.nih.gov/entrez/query.fcgi?cmd=search&db=gene&term=RTN4) | reticulon 4 | **1.183** | **718.386** | 138.679 | **849.737** | 182.751 | 4.495E-02 |
| [236529_at](https://www.affymetrix.com/LinkServlet?probeset=236529_at) | [SRCRB4D](http://www.ncbi.nlm.nih.gov/entrez/query.fcgi?cmd=search&db=gene&term=SRCRB4D) | scavenger receptor cysteine rich domain containing, group B (4 domains) | **1.185** | **6.112** | 0.294 | **7.240** | 2.068 | 3.556E-02 |
| [215096_s_at](https://www.affymetrix.com/LinkServlet?probeset=215096_s_at) | [ESD](http://www.ncbi.nlm.nih.gov/entrez/query.fcgi?cmd=search&db=gene&term=ESD) | esterase D | **1.185** | **1369.471** | 152.993 | **1622.337** | 294.889 | 8.970E-03 |
| [239612_at](https://www.affymetrix.com/LinkServlet?probeset=239612_at) | [LOC100240734](http://www.ncbi.nlm.nih.gov/entrez/query.fcgi?cmd=search&db=gene&term=LOC100240734) | hypothetical LOC100240734 | **1.185** | **6.000** | 0.000 | **7.110** | 1.698 | 1.188E-02 |
| [225602_at](https://www.affymetrix.com/LinkServlet?probeset=225602_at) | [GLIPR2](http://www.ncbi.nlm.nih.gov/entrez/query.fcgi?cmd=search&db=gene&term=GLIPR2) | GLI pathogenesis-related 2 | **1.186** | **337.305** | 48.037 | **400.047** | 84.190 | 3.132E-02 |
| [208549_x_at](https://www.affymetrix.com/LinkServlet?probeset=208549_x_at) | [PTMA](http://www.ncbi.nlm.nih.gov/entrez/query.fcgi?cmd=search&db=gene&term=PTMA) | prothymosin, alpha | **1.189** | **2010.739** | 379.340 | **2390.297** | 476.782 | 3.132E-02 |
| [201534_s_at](https://www.affymetrix.com/LinkServlet?probeset=201534_s_at) | [UBL3](http://www.ncbi.nlm.nih.gov/entrez/query.fcgi?cmd=search&db=gene&term=UBL3) | ubiquitin-like 3 | **1.189** | **505.484** | 63.036 | **600.909** | 141.656 | 4.334E-02 |
| [210094_s_at](https://www.affymetrix.com/LinkServlet?probeset=210094_s_at) | [PARD3](http://www.ncbi.nlm.nih.gov/entrez/query.fcgi?cmd=search&db=gene&term=PARD3) | par-3 partitioning defective 3 homolog (C. elegans) | **1.189** | **6.000** | 0.000 | **7.135** | 1.530 | 4.245E-03 |
| [202211_at](https://www.affymetrix.com/LinkServlet?probeset=202211_at) | [ARFGAP3](http://www.ncbi.nlm.nih.gov/entrez/query.fcgi?cmd=search&db=gene&term=ARFGAP3) | ADP-ribosylation factor GTPase activating protein 3 | **1.189** | **540.929** | 113.314 | **643.322** | 82.186 | 1.877E-02 |
| [223933_at](https://www.affymetrix.com/LinkServlet?probeset=223933_at) | [KIF5A](http://www.ncbi.nlm.nih.gov/entrez/query.fcgi?cmd=search&db=gene&term=KIF5A) | kinesin family member 5A | **1.191** | **6.000** | 0.000 | **7.145** | 1.933 | 2.140E-02 |
| [236592_at](https://www.affymetrix.com/LinkServlet?probeset=236592_at) | [NA](http://www.ncbi.nlm.nih.gov/entrez/query.fcgi?cmd=search&db=gene&term=NA) | NA | **1.191** | **6.001** | 0.004 | **7.149** | 2.192 | 3.862E-02 |
| [244568_at](https://www.affymetrix.com/LinkServlet?probeset=244568_at) | [NA](http://www.ncbi.nlm.nih.gov/entrez/query.fcgi?cmd=search&db=gene&term=NA) | NA | **1.191** | **6.168** | 0.670 | **7.348** | 1.917 | 2.910E-02 |
| [213371_at](https://www.affymetrix.com/LinkServlet?probeset=213371_at) | [LDB3](http://www.ncbi.nlm.nih.gov/entrez/query.fcgi?cmd=search&db=gene&term=LDB3) | LIM domain binding 3 | **1.192** | **6.000** | 0.000 | **7.150** | 2.311 | 4.405E-02 |
| [1554405_a_at](https://www.affymetrix.com/LinkServlet?probeset=1554405_a_at) | [NCRNA00161](http://www.ncbi.nlm.nih.gov/entrez/query.fcgi?cmd=search&db=gene&term=NCRNA00161) | non-protein coding RNA 161 | **1.192** | **6.000** | 0.000 | **7.153** | 2.085 | 3.158E-02 |
| [232066_x_at](https://www.affymetrix.com/LinkServlet?probeset=232066_x_at) | [C4orf42](http://www.ncbi.nlm.nih.gov/entrez/query.fcgi?cmd=search&db=gene&term=C4orf42) | chromosome 4 open reading frame 42 | **1.192** | **397.922** | 51.147 | **474.382** | 105.696 | 2.494E-02 |
| [242929_at](https://www.affymetrix.com/LinkServlet?probeset=242929_at) | [NA](http://www.ncbi.nlm.nih.gov/entrez/query.fcgi?cmd=search&db=gene&term=NA) | NA | **1.193** | **6.000** | 0.000 | **7.156** | 2.045 | 1.610E-02 |
| [206323_x_at](https://www.affymetrix.com/LinkServlet?probeset=206323_x_at) | [OPHN1](http://www.ncbi.nlm.nih.gov/entrez/query.fcgi?cmd=search&db=gene&term=OPHN1) | oligophrenin 1 | **1.193** | **1935.422** | 266.884 | **2309.065** | 580.128 | 3.003E-02 |
| [223480_s_at](https://www.affymetrix.com/LinkServlet?probeset=223480_s_at) | [MRPL47](http://www.ncbi.nlm.nih.gov/entrez/query.fcgi?cmd=search&db=gene&term=MRPL47) | mitochondrial ribosomal protein L47 | **1.198** | **849.026** | 217.108 | **1017.019** | 187.109 | 4.736E-02 |
| [217136_at](https://www.affymetrix.com/LinkServlet?probeset=217136_at) | [PPIAL4A](http://www.ncbi.nlm.nih.gov/entrez/query.fcgi?cmd=search&db=gene&term=PPIAL4A) | peptidylprolyl isomerase A (cyclophilin A)-like 4A | **1.199** | **6.015** | 0.051 | **7.210** | 1.970 | 1.651E-02 |
| [1569963_at](https://www.affymetrix.com/LinkServlet?probeset=1569963_at) | [NA](http://www.ncbi.nlm.nih.gov/entrez/query.fcgi?cmd=search&db=gene&term=NA) | NA | **1.201** | **6.656** | 1.276 | **7.991** | 2.150 | 4.777E-02 |
| [212428_at](https://www.affymetrix.com/LinkServlet?probeset=212428_at) | [KIAA0368](http://www.ncbi.nlm.nih.gov/entrez/query.fcgi?cmd=search&db=gene&term=KIAA0368) | KIAA0368 | **1.201** | **351.290** | 67.891 | **421.957** | 92.503 | 4.863E-02 |
| [244417_at](https://www.affymetrix.com/LinkServlet?probeset=244417_at) | [NA](http://www.ncbi.nlm.nih.gov/entrez/query.fcgi?cmd=search&db=gene&term=NA) | NA | **1.202** | **6.354** | 0.817 | **7.635** | 2.097 | 3.260E-02 |
| [212087_s_at](https://www.affymetrix.com/LinkServlet?probeset=212087_s_at) | [ERAL1](http://www.ncbi.nlm.nih.gov/entrez/query.fcgi?cmd=search&db=gene&term=ERAL1) | Era G-protein-like 1 (E. coli) | **1.202** | **128.068** | 27.998 | **153.943** | 29.207 | 2.234E-02 |
| [202485_s_at](https://www.affymetrix.com/LinkServlet?probeset=202485_s_at) | [MBD2](http://www.ncbi.nlm.nih.gov/entrez/query.fcgi?cmd=search&db=gene&term=MBD2) | methyl-CpG binding domain protein 2 | **1.203** | **6.111** | 0.444 | **7.351** | 1.992 | 2.357E-02 |
| [205806_at](https://www.affymetrix.com/LinkServlet?probeset=205806_at) | [ROM1](http://www.ncbi.nlm.nih.gov/entrez/query.fcgi?cmd=search&db=gene&term=ROM1) | retinal outer segment membrane protein 1 | **1.205** | **6.104** | 0.280 | **7.355** | 1.980 | 1.490E-02 |
| [218709_s_at](https://www.affymetrix.com/LinkServlet?probeset=218709_s_at) | [IFT52](http://www.ncbi.nlm.nih.gov/entrez/query.fcgi?cmd=search&db=gene&term=IFT52) | intraflagellar transport 52 homolog (Chlamydomonas) | **1.205** | **133.732** | 31.098 | **161.157** | 32.224 | 4.654E-02 |
| [238814_at](https://www.affymetrix.com/LinkServlet?probeset=238814_at) | [SLC35C2](http://www.ncbi.nlm.nih.gov/entrez/query.fcgi?cmd=search&db=gene&term=SLC35C2) | solute carrier family 35, member C2 | **1.206** | **6.394** | 0.836 | **7.710** | 2.249 | 4.879E-02 |
| [203538_at](https://www.affymetrix.com/LinkServlet?probeset=203538_at) | [CAMLG](http://www.ncbi.nlm.nih.gov/entrez/query.fcgi?cmd=search&db=gene&term=CAMLG) | calcium modulating ligand | **1.206** | **326.184** | 46.782 | **393.338** | 104.801 | 4.345E-02 |
| [220875_at](https://www.affymetrix.com/LinkServlet?probeset=220875_at) | [NA](http://www.ncbi.nlm.nih.gov/entrez/query.fcgi?cmd=search&db=gene&term=NA) | NA | **1.207** | **6.003** | 0.013 | **7.244** | 2.101 | 1.786E-02 |
| [210418_s_at](https://www.affymetrix.com/LinkServlet?probeset=210418_s_at) | [IDH3B](http://www.ncbi.nlm.nih.gov/entrez/query.fcgi?cmd=search&db=gene&term=IDH3B) | isocitrate dehydrogenase 3 (NAD+) beta | **1.207** | **208.754** | 47.766 | **251.981** | 46.378 | 3.988E-02 |
| [206770_s_at](https://www.affymetrix.com/LinkServlet?probeset=206770_s_at) | [SLC35A3](http://www.ncbi.nlm.nih.gov/entrez/query.fcgi?cmd=search&db=gene&term=SLC35A3) | solute carrier family 35 (UDP-N-acetylglucosamine (UDP-GlcNAc) transporter), member A3 | **1.210** | **151.169** | 40.081 | **182.869** | 44.505 | 4.422E-02 |
| [226851_at](https://www.affymetrix.com/LinkServlet?probeset=226851_at) | [LYPLAL1](http://www.ncbi.nlm.nih.gov/entrez/query.fcgi?cmd=search&db=gene&term=LYPLAL1) | lysophospholipase-like 1 | **1.211** | **379.846** | 122.595 | **459.874** | 103.481 | 4.417E-02 |
| [218903_s_at](https://www.affymetrix.com/LinkServlet?probeset=218903_s_at) | [OBFC2B](http://www.ncbi.nlm.nih.gov/entrez/query.fcgi?cmd=search&db=gene&term=OBFC2B) | oligonucleotide/oligosaccharide-binding fold containing 2B | **1.211** | **56.133** | 17.529 | **67.968** | 15.683 | 4.869E-02 |
| [203748_x_at](https://www.affymetrix.com/LinkServlet?probeset=203748_x_at) | [RBMS1](http://www.ncbi.nlm.nih.gov/entrez/query.fcgi?cmd=search&db=gene&term=RBMS1) | RNA binding motif, single stranded interacting protein 1 | **1.213** | **292.504** | 80.512 | **354.672** | 70.957 | 3.680E-02 |
| [222138_s_at](https://www.affymetrix.com/LinkServlet?probeset=222138_s_at) | [WDR13](http://www.ncbi.nlm.nih.gov/entrez/query.fcgi?cmd=search&db=gene&term=WDR13) | WD repeat domain 13 | **1.214** | **141.125** | 41.350 | **171.389** | 32.817 | 4.725E-02 |
| [1564164_at](https://www.affymetrix.com/LinkServlet?probeset=1564164_at) | [DENND1B](http://www.ncbi.nlm.nih.gov/entrez/query.fcgi?cmd=search&db=gene&term=DENND1B) | DENN/MADD domain containing 1B | **1.215** | **6.141** | 0.565 | **7.461** | 2.233 | 3.134E-02 |
| [215625_at](https://www.affymetrix.com/LinkServlet?probeset=215625_at) | [LOC644450](http://www.ncbi.nlm.nih.gov/entrez/query.fcgi?cmd=search&db=gene&term=LOC644450) | hypothetical LOC644450 | **1.216** | **6.236** | 0.645 | **7.582** | 2.091 | 2.284E-02 |
| [209382_at](https://www.affymetrix.com/LinkServlet?probeset=209382_at) | [POLR3C](http://www.ncbi.nlm.nih.gov/entrez/query.fcgi?cmd=search&db=gene&term=POLR3C) | polymerase (RNA) III (DNA directed) polypeptide C (62kD) | **1.217** | **220.011** | 72.539 | **267.657** | 53.465 | 4.138E-02 |
| [225890_at](https://www.affymetrix.com/LinkServlet?probeset=225890_at) | [C20orf72](http://www.ncbi.nlm.nih.gov/entrez/query.fcgi?cmd=search&db=gene&term=C20orf72) | chromosome 20 open reading frame 72 | **1.218** | **224.692** | 51.267 | **273.746** | 48.846 | 2.070E-02 |
| [239872_at](https://www.affymetrix.com/LinkServlet?probeset=239872_at) | [NA](http://www.ncbi.nlm.nih.gov/entrez/query.fcgi?cmd=search&db=gene&term=NA) | NA | **1.220** | **6.130** | 0.518 | **7.479** | 2.540 | 4.745E-02 |
| [211955_at](https://www.affymetrix.com/LinkServlet?probeset=211955_at) | [IPO5](http://www.ncbi.nlm.nih.gov/entrez/query.fcgi?cmd=search&db=gene&term=IPO5) | importin 5 | **1.221** | **226.896** | 44.781 | **276.956** | 51.091 | 1.333E-02 |
| [226438_at](https://www.affymetrix.com/LinkServlet?probeset=226438_at) | [SNTB1](http://www.ncbi.nlm.nih.gov/entrez/query.fcgi?cmd=search&db=gene&term=SNTB1) | syntrophin, beta 1 (dystrophin-associated protein A1, 59kDa, basic component 1) | **1.221** | **307.550** | 75.694 | **375.633** | 104.381 | 4.673E-02 |
| [228532_at](https://www.affymetrix.com/LinkServlet?probeset=228532_at) | [C1orf162](http://www.ncbi.nlm.nih.gov/entrez/query.fcgi?cmd=search&db=gene&term=C1orf162) | chromosome 1 open reading frame 162 | **1.224** | **1030.170** | 212.518 | **1261.082** | 327.108 | 3.928E-02 |
| [209042_s_at](https://www.affymetrix.com/LinkServlet?probeset=209042_s_at) | [UBE2G2](http://www.ncbi.nlm.nih.gov/entrez/query.fcgi?cmd=search&db=gene&term=UBE2G2) | ubiquitin-conjugating enzyme E2G 2 (UBC7 homolog, yeast) | **1.225** | **696.211** | 160.393 | **852.543** | 158.368 | 2.050E-02 |
| [223563_at](https://www.affymetrix.com/LinkServlet?probeset=223563_at) | [GNB1L](http://www.ncbi.nlm.nih.gov/entrez/query.fcgi?cmd=search&db=gene&term=GNB1L) | guanine nucleotide binding protein (G protein), beta polypeptide 1-like | **1.225** | **6.160** | 0.508 | **7.546** | 2.245 | 2.562E-02 |
| [1556192_x_at](https://www.affymetrix.com/LinkServlet?probeset=1556192_x_at) | [NA](http://www.ncbi.nlm.nih.gov/entrez/query.fcgi?cmd=search&db=gene&term=NA) | NA | **1.227** | **6.003** | 0.011 | **7.369** | 2.530 | 3.503E-02 |
| [228131_at](https://www.affymetrix.com/LinkServlet?probeset=228131_at) | [ERCC1](http://www.ncbi.nlm.nih.gov/entrez/query.fcgi?cmd=search&db=gene&term=ERCC1) | excision repair cross-complementing rodent repair deficiency, complementation group 1 (includes overlapping antisense sequence) | **1.228** | **686.306** | 179.911 | **842.901** | 173.704 | 2.911E-02 |
| [232169_x_at](https://www.affymetrix.com/LinkServlet?probeset=232169_x_at) | [NDUFS8](http://www.ncbi.nlm.nih.gov/entrez/query.fcgi?cmd=search&db=gene&term=NDUFS8) | NADH dehydrogenase (ubiquinone) Fe-S protein 8, 23kDa (NADH-coenzyme Q reductase) | **1.231** | **49.110** | 12.525 | **60.435** | 12.924 | 2.930E-02 |
| [217627_at](https://www.affymetrix.com/LinkServlet?probeset=217627_at) | [ZNF573](http://www.ncbi.nlm.nih.gov/entrez/query.fcgi?cmd=search&db=gene&term=ZNF573) | zinc finger protein 573 | **1.231** | **179.664** | 48.450 | **221.184** | 50.436 | 3.476E-02 |
| [216924_s_at](https://www.affymetrix.com/LinkServlet?probeset=216924_s_at) | [DRD2](http://www.ncbi.nlm.nih.gov/entrez/query.fcgi?cmd=search&db=gene&term=DRD2) | dopamine receptor D2 | **1.231** | **6.114** | 0.453 | **7.527** | 2.098 | 1.230E-02 |
| [207266_x_at](https://www.affymetrix.com/LinkServlet?probeset=207266_x_at) | [RBMS1](http://www.ncbi.nlm.nih.gov/entrez/query.fcgi?cmd=search&db=gene&term=RBMS1) | RNA binding motif, single stranded interacting protein 1 | **1.234** | **284.764** | 79.010 | **351.324** | 78.655 | 3.226E-02 |
| [1557099_at](https://www.affymetrix.com/LinkServlet?probeset=1557099_at) | [NA](http://www.ncbi.nlm.nih.gov/entrez/query.fcgi?cmd=search&db=gene&term=NA) | NA | **1.234** | **6.202** | 0.809 | **7.654** | 2.410 | 3.079E-02 |
| [214557_at](https://www.affymetrix.com/LinkServlet?probeset=214557_at) | [PTTG2](http://www.ncbi.nlm.nih.gov/entrez/query.fcgi?cmd=search&db=gene&term=PTTG2) | pituitary tumor-transforming 2 | **1.235** | **6.379** | 1.252 | **7.876** | 2.455 | 3.737E-02 |
| [208974_x_at](https://www.affymetrix.com/LinkServlet?probeset=208974_x_at) | [KPNB1](http://www.ncbi.nlm.nih.gov/entrez/query.fcgi?cmd=search&db=gene&term=KPNB1) | karyopherin (importin) beta 1 | **1.235** | **862.700** | 221.592 | **1065.344** | 161.483 | 3.415E-02 |
| [218000_s_at](https://www.affymetrix.com/LinkServlet?probeset=218000_s_at) | [PHLDA1](http://www.ncbi.nlm.nih.gov/entrez/query.fcgi?cmd=search&db=gene&term=PHLDA1) | pleckstrin homology-like domain, family A, member 1 | **1.235** | **6.000** | 0.000 | **7.413** | 2.714 | 3.945E-02 |
| [218310_at](https://www.affymetrix.com/LinkServlet?probeset=218310_at) | [RABGEF1](http://www.ncbi.nlm.nih.gov/entrez/query.fcgi?cmd=search&db=gene&term=RABGEF1) | RAB guanine nucleotide exchange factor (GEF) 1 | **1.236** | **214.698** | 57.165 | **265.319** | 36.321 | 1.542E-02 |
| [1554678_s_at](https://www.affymetrix.com/LinkServlet?probeset=1554678_s_at) | [HNRPDL](http://www.ncbi.nlm.nih.gov/entrez/query.fcgi?cmd=search&db=gene&term=HNRPDL) | heterogeneous nuclear ribonucleoprotein D-like | **1.237** | **869.802** | 160.302 | **1075.605** | 195.326 | 5.423E-03 |
| [201723_s_at](https://www.affymetrix.com/LinkServlet?probeset=201723_s_at) | [GALNT1](http://www.ncbi.nlm.nih.gov/entrez/query.fcgi?cmd=search&db=gene&term=GALNT1) | UDP-N-acetyl-alpha-D-galactosamine:polypeptide N-acetylgalactosaminyltransferase 1 (GalNAc-T1) | **1.237** | **216.431** | 50.190 | **267.699** | 37.909 | 1.342E-02 |
| [1557052_at](https://www.affymetrix.com/LinkServlet?probeset=1557052_at) | [NA](http://www.ncbi.nlm.nih.gov/entrez/query.fcgi?cmd=search&db=gene&term=NA) | NA | **1.237** | **6.650** | 1.001 | **8.227** | 2.221 | 1.811E-02 |
| [225430_at](https://www.affymetrix.com/LinkServlet?probeset=225430_at) | [GATC](http://www.ncbi.nlm.nih.gov/entrez/query.fcgi?cmd=search&db=gene&term=GATC) | glutamyl-tRNA(Gln) amidotransferase, subunit C homolog (bacterial) | **1.238** | **235.210** | 37.108 | **291.076** | 56.157 | 5.380E-03 |
| [236056_s_at](https://www.affymetrix.com/LinkServlet?probeset=236056_s_at) | [8-Mar](http://www.ncbi.nlm.nih.gov/entrez/query.fcgi?cmd=search&db=gene&term=MARCH8) | membrane-associated ring finger (C3HC4) 8 | **1.238** | **6.299** | 0.830 | **7.797** | 1.617 | 3.609E-03 |
| [216439_at](https://www.affymetrix.com/LinkServlet?probeset=216439_at) | [TNK2](http://www.ncbi.nlm.nih.gov/entrez/query.fcgi?cmd=search&db=gene&term=TNK2) | tyrosine kinase, non-receptor, 2 | **1.238** | **6.136** | 0.348 | **7.597** | 2.420 | 2.467E-02 |
| [231676_s_at](https://www.affymetrix.com/LinkServlet?probeset=231676_s_at) | [NA](http://www.ncbi.nlm.nih.gov/entrez/query.fcgi?cmd=search&db=gene&term=NA) | NA | **1.238** | **6.163** | 0.386 | **7.632** | 2.525 | 1.974E-02 |
| [222937_s_at](https://www.affymetrix.com/LinkServlet?probeset=222937_s_at) | [MMP28](http://www.ncbi.nlm.nih.gov/entrez/query.fcgi?cmd=search&db=gene&term=MMP28) | matrix metallopeptidase 28 | **1.242** | **6.172** | 0.431 | **7.668** | 2.018 | 7.158E-03 |
| [209697_at](https://www.affymetrix.com/LinkServlet?probeset=209697_at) | [PPP3CC](http://www.ncbi.nlm.nih.gov/entrez/query.fcgi?cmd=search&db=gene&term=PPP3CC) | protein phosphatase 3, catalytic subunit, gamma isozyme | **1.242** | **7.020** | 1.811 | **8.722** | 2.798 | 4.764E-02 |
| [215641_at](https://www.affymetrix.com/LinkServlet?probeset=215641_at) | [SEC24D](http://www.ncbi.nlm.nih.gov/entrez/query.fcgi?cmd=search&db=gene&term=SEC24D) | SEC24 family, member D (S. cerevisiae) | **1.242** | **6.075** | 0.287 | **7.548** | 2.854 | 4.359E-02 |
| [201661_s_at](https://www.affymetrix.com/LinkServlet?probeset=201661_s_at) | [ACSL3](http://www.ncbi.nlm.nih.gov/entrez/query.fcgi?cmd=search&db=gene&term=ACSL3) | acyl-CoA synthetase long-chain family member 3 | **1.243** | **197.142** | 62.226 | **244.979** | 43.712 | 4.944E-02 |
| [214085_x_at](https://www.affymetrix.com/LinkServlet?probeset=214085_x_at) | [NA](http://www.ncbi.nlm.nih.gov/entrez/query.fcgi?cmd=search&db=gene&term=NA) | NA | **1.244** | **924.896** | 187.758 | **1150.845** | 341.004 | 4.728E-02 |
| [205875_s_at](https://www.affymetrix.com/LinkServlet?probeset=205875_s_at) | [TREX1](http://www.ncbi.nlm.nih.gov/entrez/query.fcgi?cmd=search&db=gene&term=TREX1) | three prime repair exonuclease 1 | **1.245** | **159.998** | 51.987 | **199.148** | 40.152 | 3.593E-02 |
| [218290_at](https://www.affymetrix.com/LinkServlet?probeset=218290_at) | [PLEKHJ1](http://www.ncbi.nlm.nih.gov/entrez/query.fcgi?cmd=search&db=gene&term=PLEKHJ1) | pleckstrin homology domain containing, family J member 1 | **1.245** | **342.170** | 76.645 | **426.114** | 96.692 | 2.653E-02 |
| [215482_s_at](https://www.affymetrix.com/LinkServlet?probeset=215482_s_at) | [EIF2B4](http://www.ncbi.nlm.nih.gov/entrez/query.fcgi?cmd=search&db=gene&term=EIF2B4) | eukaryotic translation initiation factor 2B, subunit 4 delta, 67kDa | **1.246** | **251.976** | 59.892 | **313.914** | 71.230 | 1.621E-02 |
| [228183_s_at](https://www.affymetrix.com/LinkServlet?probeset=228183_s_at) | [RPAIN](http://www.ncbi.nlm.nih.gov/entrez/query.fcgi?cmd=search&db=gene&term=RPAIN) | RPA interacting protein | **1.246** | **295.514** | 70.489 | **368.158** | 67.784 | 1.768E-02 |
| [243689_s_at](https://www.affymetrix.com/LinkServlet?probeset=243689_s_at) | [FRG1B](http://www.ncbi.nlm.nih.gov/entrez/query.fcgi?cmd=search&db=gene&term=FRG1B) | FSHD region gene 1 family, member B | **1.246** | **6.288** | 0.827 | **7.835** | 2.186 | 1.830E-02 |
| [1566777_at](https://www.affymetrix.com/LinkServlet?probeset=1566777_at) | [NA](http://www.ncbi.nlm.nih.gov/entrez/query.fcgi?cmd=search&db=gene&term=NA) | NA | **1.246** | **6.000** | 0.000 | **7.477** | 2.951 | 4.099E-02 |
| [212830_at](https://www.affymetrix.com/LinkServlet?probeset=212830_at) | [MEGF9](http://www.ncbi.nlm.nih.gov/entrez/query.fcgi?cmd=search&db=gene&term=MEGF9) | multiple EGF-like-domains 9 | **1.248** | **205.754** | 45.891 | **256.685** | 52.976 | 1.903E-02 |
| [218231_at](https://www.affymetrix.com/LinkServlet?probeset=218231_at) | [NAGK](http://www.ncbi.nlm.nih.gov/entrez/query.fcgi?cmd=search&db=gene&term=NAGK) | N-acetylglucosamine kinase | **1.248** | **452.649** | 113.781 | **564.726** | 122.162 | 1.848E-02 |
| [216210_x_at](https://www.affymetrix.com/LinkServlet?probeset=216210_x_at) | [TRIOBP](http://www.ncbi.nlm.nih.gov/entrez/query.fcgi?cmd=search&db=gene&term=TRIOBP) | TRIO and F-actin binding protein | **1.249** | **162.662** | 48.975 | **203.171** | 40.370 | 3.090E-02 |
| [219299_at](https://www.affymetrix.com/LinkServlet?probeset=219299_at) | [TRMT12](http://www.ncbi.nlm.nih.gov/entrez/query.fcgi?cmd=search&db=gene&term=TRMT12) | tRNA methyltransferase 12 homolog (S. cerevisiae) | **1.250** | **314.007** | 79.361 | **392.650** | 82.441 | 2.242E-02 |
| [203530_s_at](https://www.affymetrix.com/LinkServlet?probeset=203530_s_at) | [STX4](http://www.ncbi.nlm.nih.gov/entrez/query.fcgi?cmd=search&db=gene&term=STX4) | syntaxin 4 | **1.251** | **298.144** | 70.818 | **372.896** | 90.176 | 2.635E-02 |
| [219229_at](https://www.affymetrix.com/LinkServlet?probeset=219229_at) | [SLCO3A1](http://www.ncbi.nlm.nih.gov/entrez/query.fcgi?cmd=search&db=gene&term=SLCO3A1) | solute carrier organic anion transporter family, member 3A1 | **1.251** | **208.119** | 71.872 | **260.348** | 63.749 | 4.786E-02 |
| [202055_at](https://www.affymetrix.com/LinkServlet?probeset=202055_at) | [KPNA1](http://www.ncbi.nlm.nih.gov/entrez/query.fcgi?cmd=search&db=gene&term=KPNA1) | karyopherin alpha 1 (importin alpha 5) | **1.252** | **348.495** | 79.301 | **436.325** | 72.693 | 8.308E-03 |
| [224968_at](https://www.affymetrix.com/LinkServlet?probeset=224968_at) | [CCDC104](http://www.ncbi.nlm.nih.gov/entrez/query.fcgi?cmd=search&db=gene&term=CCDC104) | coiled-coil domain containing 104 | **1.253** | **412.848** | 134.664 | **517.214** | 105.202 | 3.023E-02 |
| [1552701_a_at](https://www.affymetrix.com/LinkServlet?probeset=1552701_a_at) | [CARD16](http://www.ncbi.nlm.nih.gov/entrez/query.fcgi?cmd=search&db=gene&term=CARD16) | caspase recruitment domain family, member 16 | **1.255** | **550.330** | 81.824 | **690.408** | 168.327 | 1.221E-02 |
| [226527_at](https://www.affymetrix.com/LinkServlet?probeset=226527_at) | [RPRD2](http://www.ncbi.nlm.nih.gov/entrez/query.fcgi?cmd=search&db=gene&term=RPRD2) | regulation of nuclear pre-mRNA domain containing 2 | **1.255** | **144.002** | 39.057 | **180.754** | 33.296 | 1.549E-02 |
| [222515_x_at](https://www.affymetrix.com/LinkServlet?probeset=222515_x_at) | [TMEM165](http://www.ncbi.nlm.nih.gov/entrez/query.fcgi?cmd=search&db=gene&term=TMEM165) | transmembrane protein 165 | **1.256** | **8.416** | 2.526 | **10.567** | 2.484 | 4.064E-02 |
| [236880_at](https://www.affymetrix.com/LinkServlet?probeset=236880_at) | [RAD52](http://www.ncbi.nlm.nih.gov/entrez/query.fcgi?cmd=search&db=gene&term=RAD52) | RAD52 homolog (S. cerevisiae) | **1.257** | **6.214** | 0.606 | **7.812** | 2.665 | 2.074E-02 |
| [235444_at](https://www.affymetrix.com/LinkServlet?probeset=235444_at) | [FOXP1](http://www.ncbi.nlm.nih.gov/entrez/query.fcgi?cmd=search&db=gene&term=FOXP1) | forkhead box P1 | **1.257** | **189.995** | 57.091 | **238.897** | 38.092 | 2.419E-02 |
| [216947_at](https://www.affymetrix.com/LinkServlet?probeset=216947_at) | [DES](http://www.ncbi.nlm.nih.gov/entrez/query.fcgi?cmd=search&db=gene&term=DES) | desmin | **1.258** | **6.028** | 0.113 | **7.581** | 2.758 | 3.208E-02 |
| [1558588_at](https://www.affymetrix.com/LinkServlet?probeset=1558588_at) | [LOC728855](http://www.ncbi.nlm.nih.gov/entrez/query.fcgi?cmd=search&db=gene&term=LOC728855) | hypothetical LOC728855 | **1.258** | **6.029** | 0.117 | **7.584** | 2.760 | 2.759E-02 |
| [1560224_at](https://www.affymetrix.com/LinkServlet?probeset=1560224_at) | [AHCTF1](http://www.ncbi.nlm.nih.gov/entrez/query.fcgi?cmd=search&db=gene&term=AHCTF1) | AT hook containing transcription factor 1 | **1.258** | **6.248** | 0.537 | **7.862** | 2.748 | 3.101E-02 |
| [224955_at](https://www.affymetrix.com/LinkServlet?probeset=224955_at) | [TEAD1](http://www.ncbi.nlm.nih.gov/entrez/query.fcgi?cmd=search&db=gene&term=TEAD1) | TEA domain family member 1 (SV40 transcriptional enhancer factor) | **1.259** | **6.041** | 0.162 | **7.606** | 2.742 | 2.216E-02 |
| [208470_s_at](https://www.affymetrix.com/LinkServlet?probeset=208470_s_at) | [NA](http://www.ncbi.nlm.nih.gov/entrez/query.fcgi?cmd=search&db=gene&term=NA) | NA | **1.260** | **6.176** | 0.702 | **7.780** | 3.124 | 4.770E-02 |
| [207677_s_at](https://www.affymetrix.com/LinkServlet?probeset=207677_s_at) | [NCF4](http://www.ncbi.nlm.nih.gov/entrez/query.fcgi?cmd=search&db=gene&term=NCF4) | neutrophil cytosolic factor 4, 40kDa | **1.261** | **345.581** | 84.929 | **435.706** | 107.190 | 2.351E-02 |
| [211370_s_at](https://www.affymetrix.com/LinkServlet?probeset=211370_s_at) | [MAP2K5](http://www.ncbi.nlm.nih.gov/entrez/query.fcgi?cmd=search&db=gene&term=MAP2K5) | mitogen-activated protein kinase kinase 5 | **1.263** | **117.500** | 33.615 | **148.411** | 33.786 | 3.318E-02 |
| [1569473_s_at](https://www.affymetrix.com/LinkServlet?probeset=1569473_s_at) | [LOC155060](http://www.ncbi.nlm.nih.gov/entrez/query.fcgi?cmd=search&db=gene&term=LOC155060) | AI894139 pseudogene | **1.263** | **6.167** | 0.401 | **7.791** | 1.846 | 1.619E-03 |
| [224513_s_at](https://www.affymetrix.com/LinkServlet?probeset=224513_s_at) | [UBQLN4](http://www.ncbi.nlm.nih.gov/entrez/query.fcgi?cmd=search&db=gene&term=UBQLN4) | ubiquilin 4 | **1.264** | **259.920** | 54.403 | **328.603** | 93.922 | 2.839E-02 |
| [232330_at](https://www.affymetrix.com/LinkServlet?probeset=232330_at) | [C7orf44](http://www.ncbi.nlm.nih.gov/entrez/query.fcgi?cmd=search&db=gene&term=C7orf44) | chromosome 7 open reading frame 44 | **1.266** | **102.427** | 40.705 | **129.660** | 18.426 | 3.651E-02 |
| [209868_s_at](https://www.affymetrix.com/LinkServlet?probeset=209868_s_at) | [RBMS1](http://www.ncbi.nlm.nih.gov/entrez/query.fcgi?cmd=search&db=gene&term=RBMS1) | RNA binding motif, single stranded interacting protein 1 | **1.266** | **244.833** | 74.102 | **309.956** | 76.049 | 3.099E-02 |
| [206157_at](https://www.affymetrix.com/LinkServlet?probeset=206157_at) | [PTX3](http://www.ncbi.nlm.nih.gov/entrez/query.fcgi?cmd=search&db=gene&term=PTX3) | pentraxin 3, long | **1.267** | **6.325** | 1.301 | **8.012** | 2.854 | 4.380E-02 |
| [221443_x_at](https://www.affymetrix.com/LinkServlet?probeset=221443_x_at) | [PRLH](http://www.ncbi.nlm.nih.gov/entrez/query.fcgi?cmd=search&db=gene&term=PRLH) | prolactin releasing hormone | **1.267** | **7.555** | 1.920 | **9.576** | 3.095 | 4.111E-02 |
| [215160_x_at](https://www.affymetrix.com/LinkServlet?probeset=215160_x_at) | [LOC642236](http://www.ncbi.nlm.nih.gov/entrez/query.fcgi?cmd=search&db=gene&term=LOC642236) | FSHD region gene 1 pseudogene | **1.271** | **712.595** | 189.092 | **905.525** | 262.170 | 2.324E-02 |
| [228094_at](https://www.affymetrix.com/LinkServlet?probeset=228094_at) | [AMICA1](http://www.ncbi.nlm.nih.gov/entrez/query.fcgi?cmd=search&db=gene&term=AMICA1) | adhesion molecule, interacts with CXADR antigen 1 | **1.271** | **815.999** | 143.718 | **1037.013** | 234.886 | 9.286E-03 |
| [218249_at](https://www.affymetrix.com/LinkServlet?probeset=218249_at) | [ZDHHC6](http://www.ncbi.nlm.nih.gov/entrez/query.fcgi?cmd=search&db=gene&term=ZDHHC6) | zinc finger, DHHC-type containing 6 | **1.273** | **331.938** | 74.394 | **422.459** | 46.463 | 1.598E-03 |
| [218589_at](https://www.affymetrix.com/LinkServlet?probeset=218589_at) | [LPAR6](http://www.ncbi.nlm.nih.gov/entrez/query.fcgi?cmd=search&db=gene&term=LPAR6) | lysophosphatidic acid receptor 6 | **1.273** | **847.626** | 204.171 | **1079.203** | 285.525 | 2.602E-02 |
| [224014_at](https://www.affymetrix.com/LinkServlet?probeset=224014_at) | [ZNF160](http://www.ncbi.nlm.nih.gov/entrez/query.fcgi?cmd=search&db=gene&term=ZNF160) | zinc finger protein 160 | **1.275** | **6.000** | 0.000 | **7.652** | 2.863 | 2.404E-02 |
| [222483_at](https://www.affymetrix.com/LinkServlet?probeset=222483_at) | [EFHD2](http://www.ncbi.nlm.nih.gov/entrez/query.fcgi?cmd=search&db=gene&term=EFHD2) | EF-hand domain family, member D2 | **1.275** | **58.288** | 16.208 | **74.334** | 19.610 | 1.968E-02 |
| [208727_s_at](https://www.affymetrix.com/LinkServlet?probeset=208727_s_at) | [CDC42](http://www.ncbi.nlm.nih.gov/entrez/query.fcgi?cmd=search&db=gene&term=CDC42) | cell division cycle 42 (GTP binding protein, 25kDa) | **1.276** | **352.087** | 98.075 | **449.119** | 153.307 | 4.811E-02 |
| [208971_at](https://www.affymetrix.com/LinkServlet?probeset=208971_at) | [UROD](http://www.ncbi.nlm.nih.gov/entrez/query.fcgi?cmd=search&db=gene&term=UROD) | uroporphyrinogen decarboxylase | **1.276** | **212.254** | 31.076 | **270.941** | 68.481 | 8.774E-03 |
| [204203_at](https://www.affymetrix.com/LinkServlet?probeset=204203_at) | [CEBPG](http://www.ncbi.nlm.nih.gov/entrez/query.fcgi?cmd=search&db=gene&term=CEBPG) | CCAAT/enhancer binding protein (C/EBP), gamma | **1.278** | **347.696** | 89.581 | **444.214** | 94.542 | 1.707E-02 |
| [1558502_s_at](https://www.affymetrix.com/LinkServlet?probeset=1558502_s_at) | [DNM3](http://www.ncbi.nlm.nih.gov/entrez/query.fcgi?cmd=search&db=gene&term=DNM3) | dynamin 3 | **1.278** | **6.328** | 0.773 | **8.086** | 3.138 | 3.697E-02 |
| [205173_x_at](https://www.affymetrix.com/LinkServlet?probeset=205173_x_at) | [CD58](http://www.ncbi.nlm.nih.gov/entrez/query.fcgi?cmd=search&db=gene&term=CD58) | CD58 molecule | **1.278** | **465.651** | 119.692 | **595.006** | 174.581 | 3.953E-02 |
| [221939_at](https://www.affymetrix.com/LinkServlet?probeset=221939_at) | [NA](http://www.ncbi.nlm.nih.gov/entrez/query.fcgi?cmd=search&db=gene&term=NA) | NA | **1.281** | **234.503** | 63.978 | **300.396** | 88.553 | 3.386E-02 |
| [218425_at](https://www.affymetrix.com/LinkServlet?probeset=218425_at) | [RNF216](http://www.ncbi.nlm.nih.gov/entrez/query.fcgi?cmd=search&db=gene&term=RNF216) | ring finger protein 216 | **1.283** | **64.571** | 14.785 | **82.833** | 26.742 | 4.798E-02 |
| [205784_x_at](https://www.affymetrix.com/LinkServlet?probeset=205784_x_at) | [ARVCF](http://www.ncbi.nlm.nih.gov/entrez/query.fcgi?cmd=search&db=gene&term=ARVCF) | armadillo repeat gene deleted in velocardiofacial syndrome | **1.284** | **6.285** | 0.812 | **8.068** | 2.793 | 1.939E-02 |
| [1559065_a_at](https://www.affymetrix.com/LinkServlet?probeset=1559065_a_at) | [CLEC4G](http://www.ncbi.nlm.nih.gov/entrez/query.fcgi?cmd=search&db=gene&term=CLEC4G) | C-type lectin domain family 4, member G | **1.286** | **6.281** | 0.437 | **8.080** | 3.156 | 2.834E-02 |
| [226958_s_at](https://www.affymetrix.com/LinkServlet?probeset=226958_s_at) | [MED11](http://www.ncbi.nlm.nih.gov/entrez/query.fcgi?cmd=search&db=gene&term=MED11) | mediator complex subunit 11 | **1.288** | **411.084** | 80.435 | **529.662** | 134.126 | 1.130E-02 |
| [1553498_at](https://www.affymetrix.com/LinkServlet?probeset=1553498_at) | [NA](http://www.ncbi.nlm.nih.gov/entrez/query.fcgi?cmd=search&db=gene&term=NA) | NA | **1.290** | **6.000** | 0.000 | **7.741** | 2.735 | 1.400E-02 |
| [209009_at](https://www.affymetrix.com/LinkServlet?probeset=209009_at) | [ESD](http://www.ncbi.nlm.nih.gov/entrez/query.fcgi?cmd=search&db=gene&term=ESD) | esterase D | **1.291** | **973.383** | 139.446 | **1256.757** | 322.591 | 6.801E-03 |
| [243802_at](https://www.affymetrix.com/LinkServlet?probeset=243802_at) | [DNAH12](http://www.ncbi.nlm.nih.gov/entrez/query.fcgi?cmd=search&db=gene&term=DNAH12) | dynein, axonemal, heavy chain 12 | **1.291** | **6.069** | 0.276 | **7.837** | 3.724 | 4.459E-02 |
| [1553593_a_at](https://www.affymetrix.com/LinkServlet?probeset=1553593_a_at) | [TAL2](http://www.ncbi.nlm.nih.gov/entrez/query.fcgi?cmd=search&db=gene&term=TAL2) | T-cell acute lymphocytic leukemia 2 | **1.293** | **6.087** | 0.243 | **7.870** | 3.732 | 3.537E-02 |
| [201945_at](https://www.affymetrix.com/LinkServlet?probeset=201945_at) | [FURIN](http://www.ncbi.nlm.nih.gov/entrez/query.fcgi?cmd=search&db=gene&term=FURIN) | furin (paired basic amino acid cleaving enzyme) | **1.294** | **129.742** | 45.192 | **167.832** | 49.951 | 4.703E-02 |
| [224130_s_at](https://www.affymetrix.com/LinkServlet?probeset=224130_s_at) | [SRA1](http://www.ncbi.nlm.nih.gov/entrez/query.fcgi?cmd=search&db=gene&term=SRA1) | steroid receptor RNA activator 1 | **1.294** | **256.944** | 48.500 | **332.499** | 80.644 | 4.425E-03 |
| [207765_s_at](https://www.affymetrix.com/LinkServlet?probeset=207765_s_at) | [KIAA1539](http://www.ncbi.nlm.nih.gov/entrez/query.fcgi?cmd=search&db=gene&term=KIAA1539) | KIAA1539 | **1.295** | **40.902** | 19.682 | **52.949** | 13.896 | 3.513E-02 |
| [203081_at](https://www.affymetrix.com/LinkServlet?probeset=203081_at) | [CTNNBIP1](http://www.ncbi.nlm.nih.gov/entrez/query.fcgi?cmd=search&db=gene&term=CTNNBIP1) | catenin, beta interacting protein 1 | **1.295** | **21.561** | 5.788 | **27.926** | 7.220 | 2.040E-02 |
| [232830_at](https://www.affymetrix.com/LinkServlet?probeset=232830_at) | [RNF32](http://www.ncbi.nlm.nih.gov/entrez/query.fcgi?cmd=search&db=gene&term=RNF32) | ring finger protein 32 | **1.297** | **6.920** | 1.992 | **8.974** | 3.449 | 3.704E-02 |
| [218672_at](https://www.affymetrix.com/LinkServlet?probeset=218672_at) | [SCNM1](http://www.ncbi.nlm.nih.gov/entrez/query.fcgi?cmd=search&db=gene&term=SCNM1) | sodium channel modifier 1 | **1.298** | **712.577** | 104.927 | **924.897** | 303.164 | 1.801E-02 |
| [243861_at](https://www.affymetrix.com/LinkServlet?probeset=243861_at) | [NA](http://www.ncbi.nlm.nih.gov/entrez/query.fcgi?cmd=search&db=gene&term=NA) | NA | **1.298** | **6.142** | 0.553 | **7.973** | 3.308 | 3.256E-02 |
| [204132_s_at](https://www.affymetrix.com/LinkServlet?probeset=204132_s_at) | [NA](http://www.ncbi.nlm.nih.gov/entrez/query.fcgi?cmd=search&db=gene&term=NA) | NA | **1.298** | **78.182** | 19.114 | **101.503** | 30.959 | 3.083E-02 |
| [211743_s_at](https://www.affymetrix.com/LinkServlet?probeset=211743_s_at) | [PRG2](http://www.ncbi.nlm.nih.gov/entrez/query.fcgi?cmd=search&db=gene&term=PRG2) | proteoglycan 2, bone marrow (natural killer cell activator, eosinophil granule major basic protein) | **1.300** | **6.569** | 1.257 | **8.538** | 3.108 | 3.252E-02 |
| [231692_at](https://www.affymetrix.com/LinkServlet?probeset=231692_at) | [PIGG](http://www.ncbi.nlm.nih.gov/entrez/query.fcgi?cmd=search&db=gene&term=PIGG) | phosphatidylinositol glycan anchor biosynthesis, class G | **1.300** | **45.815** | 12.399 | **59.548** | 13.143 | 6.051E-03 |
| [221604_s_at](https://www.affymetrix.com/LinkServlet?probeset=221604_s_at) | [PEX16](http://www.ncbi.nlm.nih.gov/entrez/query.fcgi?cmd=search&db=gene&term=PEX16) | peroxisomal biogenesis factor 16 | **1.301** | **91.801** | 26.026 | **119.465** | 37.584 | 4.482E-02 |
| [221600_s_at](https://www.affymetrix.com/LinkServlet?probeset=221600_s_at) | [C11orf67](http://www.ncbi.nlm.nih.gov/entrez/query.fcgi?cmd=search&db=gene&term=C11orf67) | chromosome 11 open reading frame 67 | **1.302** | **48.116** | 16.288 | **62.635** | 17.455 | 4.436E-02 |
| [240670_at](https://www.affymetrix.com/LinkServlet?probeset=240670_at) | [NA](http://www.ncbi.nlm.nih.gov/entrez/query.fcgi?cmd=search&db=gene&term=NA) | NA | **1.304** | **6.399** | 1.316 | **8.347** | 3.180 | 3.410E-02 |
| [200941_at](https://www.affymetrix.com/LinkServlet?probeset=200941_at) | [HSBP1](http://www.ncbi.nlm.nih.gov/entrez/query.fcgi?cmd=search&db=gene&term=HSBP1) | heat shock factor binding protein 1 | **1.305** | **685.823** | 145.125 | **895.244** | 199.320 | 5.030E-03 |
| [237349_at](https://www.affymetrix.com/LinkServlet?probeset=237349_at) | [TSHR](http://www.ncbi.nlm.nih.gov/entrez/query.fcgi?cmd=search&db=gene&term=TSHR) | thyroid stimulating hormone receptor | **1.306** | **6.000** | 0.000 | **7.834** | 3.374 | 2.615E-02 |
| [203656_at](https://www.affymetrix.com/LinkServlet?probeset=203656_at) | [FIG4](http://www.ncbi.nlm.nih.gov/entrez/query.fcgi?cmd=search&db=gene&term=FIG4) | FIG4 homolog, SAC1 lipid phosphatase domain containing (S. cerevisiae) | **1.306** | **148.103** | 35.785 | **193.394** | 43.566 | 8.811E-03 |
| [207511_s_at](https://www.affymetrix.com/LinkServlet?probeset=207511_s_at) | [C2orf24](http://www.ncbi.nlm.nih.gov/entrez/query.fcgi?cmd=search&db=gene&term=C2orf24) | chromosome 2 open reading frame 24 | **1.306** | **79.187** | 23.188 | **103.444** | 29.114 | 2.822E-02 |
| [223738_s_at](https://www.affymetrix.com/LinkServlet?probeset=223738_s_at) | [PGM2](http://www.ncbi.nlm.nih.gov/entrez/query.fcgi?cmd=search&db=gene&term=PGM2) | phosphoglucomutase 2 | **1.307** | **116.821** | 20.160 | **152.628** | 54.445 | 3.731E-02 |
| [202446_s_at](https://www.affymetrix.com/LinkServlet?probeset=202446_s_at) | [PLSCR1](http://www.ncbi.nlm.nih.gov/entrez/query.fcgi?cmd=search&db=gene&term=PLSCR1) | phospholipid scramblase 1 | **1.307** | **267.425** | 104.827 | **349.400** | 109.047 | 4.882E-02 |
| [241405_at](https://www.affymetrix.com/LinkServlet?probeset=241405_at) | [LOC400604](http://www.ncbi.nlm.nih.gov/entrez/query.fcgi?cmd=search&db=gene&term=LOC400604) | hypothetical LOC400604 | **1.307** | **7.192** | 1.531 | **9.398** | 3.022 | 2.675E-02 |
| [1562263_at](https://www.affymetrix.com/LinkServlet?probeset=1562263_at) | [LOC100507156](http://www.ncbi.nlm.nih.gov/entrez/query.fcgi?cmd=search&db=gene&term=LOC100507156) | hypothetical LOC100507156 | **1.308** | **6.154** | 0.534 | **8.046** | 3.544 | 3.917E-02 |
| [223031_s_at](https://www.affymetrix.com/LinkServlet?probeset=223031_s_at) | [TRAF7](http://www.ncbi.nlm.nih.gov/entrez/query.fcgi?cmd=search&db=gene&term=TRAF7) | TNF receptor-associated factor 7 | **1.308** | **468.823** | 97.501 | **613.392** | 174.904 | 8.359E-03 |
| [224307_x_at](https://www.affymetrix.com/LinkServlet?probeset=224307_x_at) | [MAGI3](http://www.ncbi.nlm.nih.gov/entrez/query.fcgi?cmd=search&db=gene&term=MAGI3) | membrane associated guanylate kinase, WW and PDZ domain containing 3 | **1.309** | **6.737** | 0.833 | **8.818** | 3.242 | 2.758E-02 |
| [1570185_at](https://www.affymetrix.com/LinkServlet?probeset=1570185_at) | [NA](http://www.ncbi.nlm.nih.gov/entrez/query.fcgi?cmd=search&db=gene&term=NA) | NA | **1.309** | **7.069** | 1.619 | **9.256** | 3.190 | 3.399E-02 |
| [205571_at](https://www.affymetrix.com/LinkServlet?probeset=205571_at) | [LIPT1](http://www.ncbi.nlm.nih.gov/entrez/query.fcgi?cmd=search&db=gene&term=LIPT1) | lipoyltransferase 1 | **1.310** | **214.425** | 78.434 | **280.808** | 57.208 | 1.808E-02 |
| [213279_at](https://www.affymetrix.com/LinkServlet?probeset=213279_at) | [DHRS1](http://www.ncbi.nlm.nih.gov/entrez/query.fcgi?cmd=search&db=gene&term=DHRS1) | dehydrogenase/reductase (SDR family) member 1 | **1.312** | **168.807** | 62.276 | **221.509** | 52.011 | 3.533E-02 |
| [209941_at](https://www.affymetrix.com/LinkServlet?probeset=209941_at) | [RIPK1](http://www.ncbi.nlm.nih.gov/entrez/query.fcgi?cmd=search&db=gene&term=RIPK1) | receptor (TNFRSF)-interacting serine-threonine kinase 1 | **1.313** | **56.765** | 22.166 | **74.556** | 16.171 | 2.946E-02 |
| [214430_at](https://www.affymetrix.com/LinkServlet?probeset=214430_at) | [GLA](http://www.ncbi.nlm.nih.gov/entrez/query.fcgi?cmd=search&db=gene&term=GLA) | galactosidase, alpha | **1.314** | **227.901** | 95.582 | **299.392** | 75.719 | 1.480E-02 |
| [210592_s_at](https://www.affymetrix.com/LinkServlet?probeset=210592_s_at) | [SAT1](http://www.ncbi.nlm.nih.gov/entrez/query.fcgi?cmd=search&db=gene&term=SAT1) | spermidine/spermine N1-acetyltransferase 1 | **1.314** | **1497.310** | 428.882 | **1967.036** | 636.805 | 2.892E-02 |
| [203720_s_at](https://www.affymetrix.com/LinkServlet?probeset=203720_s_at) | [ERCC1](http://www.ncbi.nlm.nih.gov/entrez/query.fcgi?cmd=search&db=gene&term=ERCC1) | excision repair cross-complementing rodent repair deficiency, complementation group 1 (includes overlapping antisense sequence) | **1.314** | **225.908** | 41.053 | **296.813** | 103.655 | 3.695E-02 |
| [205603_s_at](https://www.affymetrix.com/LinkServlet?probeset=205603_s_at) | [DIAPH2](http://www.ncbi.nlm.nih.gov/entrez/query.fcgi?cmd=search&db=gene&term=DIAPH2) | diaphanous homolog 2 (Drosophila) | **1.316** | **107.480** | 25.162 | **141.441** | 41.737 | 8.566E-03 |
| [225624_at](https://www.affymetrix.com/LinkServlet?probeset=225624_at) | [SNX29](http://www.ncbi.nlm.nih.gov/entrez/query.fcgi?cmd=search&db=gene&term=SNX29) | sorting nexin 29 | **1.316** | **125.480** | 34.621 | **165.160** | 53.332 | 3.358E-02 |
| [205338_s_at](https://www.affymetrix.com/LinkServlet?probeset=205338_s_at) | [DCT](http://www.ncbi.nlm.nih.gov/entrez/query.fcgi?cmd=search&db=gene&term=DCT) | dopachrome tautomerase (dopachrome delta-isomerase, tyrosine-related protein 2) | **1.317** | **6.000** | 0.000 | **7.903** | 3.311 | 2.760E-02 |
| [209230_s_at](https://www.affymetrix.com/LinkServlet?probeset=209230_s_at) | [NUPR1](http://www.ncbi.nlm.nih.gov/entrez/query.fcgi?cmd=search&db=gene&term=NUPR1) | nuclear protein, transcriptional regulator, 1 | **1.319** | **53.809** | 68.564 | **70.985** | 33.397 | 4.324E-02 |
| [209885_at](https://www.affymetrix.com/LinkServlet?probeset=209885_at) | [RHOD](http://www.ncbi.nlm.nih.gov/entrez/query.fcgi?cmd=search&db=gene&term=RHOD) | ras homolog gene family, member D | **1.319** | **6.230** | 0.918 | **8.219** | 3.508 | 3.183E-02 |
| [239316_at](https://www.affymetrix.com/LinkServlet?probeset=239316_at) | [METTL12](http://www.ncbi.nlm.nih.gov/entrez/query.fcgi?cmd=search&db=gene&term=METTL12) | methyltransferase like 12 | **1.320** | **10.562** | 4.939 | **13.941** | 3.647 | 2.794E-02 |
| [1559289_at](https://www.affymetrix.com/LinkServlet?probeset=1559289_at) | [TADA1](http://www.ncbi.nlm.nih.gov/entrez/query.fcgi?cmd=search&db=gene&term=TADA1) | transcriptional adaptor 1 | **1.321** | **6.000** | 0.000 | **7.927** | 3.573 | 2.625E-02 |
| [205084_at](https://www.affymetrix.com/LinkServlet?probeset=205084_at) | [BCAP29](http://www.ncbi.nlm.nih.gov/entrez/query.fcgi?cmd=search&db=gene&term=BCAP29) | B-cell receptor-associated protein 29 | **1.322** | **141.679** | 38.443 | **187.306** | 62.486 | 3.827E-02 |
| [210482_x_at](https://www.affymetrix.com/LinkServlet?probeset=210482_x_at) | [MAP2K5](http://www.ncbi.nlm.nih.gov/entrez/query.fcgi?cmd=search&db=gene&term=MAP2K5) | mitogen-activated protein kinase kinase 5 | **1.323** | **72.561** | 20.938 | **96.021** | 21.100 | 1.728E-02 |
| [233250_x_at](https://www.affymetrix.com/LinkServlet?probeset=233250_x_at) | [FOXRED2](http://www.ncbi.nlm.nih.gov/entrez/query.fcgi?cmd=search&db=gene&term=FOXRED2) | FAD-dependent oxidoreductase domain containing 2 | **1.324** | **6.431** | 0.748 | **8.514** | 3.656 | 3.349E-02 |
| [227481_at](https://www.affymetrix.com/LinkServlet?probeset=227481_at) | [CNKSR3](http://www.ncbi.nlm.nih.gov/entrez/query.fcgi?cmd=search&db=gene&term=CNKSR3) | CNKSR family member 3 | **1.324** | **6.000** | 0.000 | **7.947** | 3.939 | 4.577E-02 |
| [211744_s_at](https://www.affymetrix.com/LinkServlet?probeset=211744_s_at) | [CD58](http://www.ncbi.nlm.nih.gov/entrez/query.fcgi?cmd=search&db=gene&term=CD58) | CD58 molecule | **1.324** | **185.066** | 51.675 | **245.110** | 74.681 | 2.654E-02 |
| [221478_at](https://www.affymetrix.com/LinkServlet?probeset=221478_at) | [BNIP3L](http://www.ncbi.nlm.nih.gov/entrez/query.fcgi?cmd=search&db=gene&term=BNIP3L) | BCL2/adenovirus E1B 19kDa interacting protein 3-like | **1.326** | **618.348** | 168.982 | **819.705** | 283.783 | 4.206E-02 |
| [224958_at](https://www.affymetrix.com/LinkServlet?probeset=224958_at) | [NUFIP2](http://www.ncbi.nlm.nih.gov/entrez/query.fcgi?cmd=search&db=gene&term=NUFIP2) | nuclear fragile X mental retardation protein interacting protein 2 | **1.328** | **279.634** | 58.806 | **371.403** | 91.602 | 7.918E-03 |
| [213781_at](https://www.affymetrix.com/LinkServlet?probeset=213781_at) | [LRRC68](http://www.ncbi.nlm.nih.gov/entrez/query.fcgi?cmd=search&db=gene&term=LRRC68) | leucine rich repeat containing 68 | **1.330** | **6.101** | 0.283 | **8.112** | 3.807 | 3.270E-02 |
| [228483_s_at](https://www.affymetrix.com/LinkServlet?probeset=228483_s_at) | [TAF9B](http://www.ncbi.nlm.nih.gov/entrez/query.fcgi?cmd=search&db=gene&term=TAF9B) | TAF9B RNA polymerase II, TATA box binding protein (TBP)-associated factor, 31kDa | **1.330** | **102.610** | 33.471 | **136.460** | 43.161 | 4.598E-02 |
| [233506_at](https://www.affymetrix.com/LinkServlet?probeset=233506_at) | [NA](http://www.ncbi.nlm.nih.gov/entrez/query.fcgi?cmd=search&db=gene&term=NA) | NA | **1.330** | **306.509** | 92.386 | **407.710** | 148.659 | 2.786E-02 |
| [201571_s_at](https://www.affymetrix.com/LinkServlet?probeset=201571_s_at) | [DCTD](http://www.ncbi.nlm.nih.gov/entrez/query.fcgi?cmd=search&db=gene&term=DCTD) | dCMP deaminase | **1.333** | **80.076** | 30.051 | **106.766** | 30.964 | 2.825E-02 |
| [220173_at](https://www.affymetrix.com/LinkServlet?probeset=220173_at) | [C14orf45](http://www.ncbi.nlm.nih.gov/entrez/query.fcgi?cmd=search&db=gene&term=C14orf45) | chromosome 14 open reading frame 45 | **1.334** | **10.486** | 4.153 | **13.993** | 4.633 | 4.502E-02 |
| [216949_s_at](https://www.affymetrix.com/LinkServlet?probeset=216949_s_at) | [PKD1](http://www.ncbi.nlm.nih.gov/entrez/query.fcgi?cmd=search&db=gene&term=PKD1) | polycystic kidney disease 1 (autosomal dominant) | **1.335** | **7.189** | 2.014 | **9.595** | 2.045 | 2.897E-03 |
| [216942_s_at](https://www.affymetrix.com/LinkServlet?probeset=216942_s_at) | [CD58](http://www.ncbi.nlm.nih.gov/entrez/query.fcgi?cmd=search&db=gene&term=CD58) | CD58 molecule | **1.336** | **102.659** | 33.722 | **137.186** | 37.439 | 2.597E-02 |
| [227097_at](https://www.affymetrix.com/LinkServlet?probeset=227097_at) | [ZNF800](http://www.ncbi.nlm.nih.gov/entrez/query.fcgi?cmd=search&db=gene&term=ZNF800) | zinc finger protein 800 | **1.336** | **6.112** | 0.337 | **8.168** | 3.765 | 3.296E-02 |
| [205147_x_at](https://www.affymetrix.com/LinkServlet?probeset=205147_x_at) | [NCF4](http://www.ncbi.nlm.nih.gov/entrez/query.fcgi?cmd=search&db=gene&term=NCF4) | neutrophil cytosolic factor 4, 40kDa | **1.337** | **254.022** | 68.199 | **339.591** | 109.878 | 4.073E-02 |
| [202996_at](https://www.affymetrix.com/LinkServlet?probeset=202996_at) | [POLD4](http://www.ncbi.nlm.nih.gov/entrez/query.fcgi?cmd=search&db=gene&term=POLD4) | polymerase (DNA-directed), delta 4 | **1.337** | **158.698** | 35.578 | **212.160** | 77.498 | 2.506E-02 |
| [213335_s_at](https://www.affymetrix.com/LinkServlet?probeset=213335_s_at) | [ST3GAL6](http://www.ncbi.nlm.nih.gov/entrez/query.fcgi?cmd=search&db=gene&term=ST3GAL6) | ST3 beta-galactoside alpha-2,3-sialyltransferase 6 | **1.338** | **6.967** | 2.327 | **9.323** | 2.806 | 1.513E-02 |
| [213581_at](https://www.affymetrix.com/LinkServlet?probeset=213581_at) | [PDCD2](http://www.ncbi.nlm.nih.gov/entrez/query.fcgi?cmd=search&db=gene&term=PDCD2) | programmed cell death 2 | **1.339** | **216.442** | 58.892 | **289.773** | 94.217 | 3.106E-02 |
| [240037_at](https://www.affymetrix.com/LinkServlet?probeset=240037_at) | [ITPRIPL1](http://www.ncbi.nlm.nih.gov/entrez/query.fcgi?cmd=search&db=gene&term=ITPRIPL1) | inositol 1,4,5-triphosphate receptor interacting protein-like 1 | **1.339** | **35.006** | 14.038 | **46.878** | 13.119 | 2.044E-02 |
| [229446_at](https://www.affymetrix.com/LinkServlet?probeset=229446_at) | [NA](http://www.ncbi.nlm.nih.gov/entrez/query.fcgi?cmd=search&db=gene&term=NA) | NA | **1.339** | **6.197** | 0.407 | **8.300** | 3.406 | 1.852E-02 |
| [224896_s_at](https://www.affymetrix.com/LinkServlet?probeset=224896_s_at) | [TTL](http://www.ncbi.nlm.nih.gov/entrez/query.fcgi?cmd=search&db=gene&term=TTL) | tubulin tyrosine ligase | **1.340** | **514.305** | 121.681 | **689.049** | 174.669 | 4.539E-03 |
| [219228_at](https://www.affymetrix.com/LinkServlet?probeset=219228_at) | [ZNF331](http://www.ncbi.nlm.nih.gov/entrez/query.fcgi?cmd=search&db=gene&term=ZNF331) | zinc finger protein 331 | **1.341** | **168.277** | 44.034 | **225.590** | 69.839 | 2.462E-02 |
| [204323_x_at](https://www.affymetrix.com/LinkServlet?probeset=204323_x_at) | [NF1](http://www.ncbi.nlm.nih.gov/entrez/query.fcgi?cmd=search&db=gene&term=NF1) | neurofibromin 1 | **1.341** | **6.112** | 0.305 | **8.196** | 4.246 | 4.559E-02 |
| [238068_at](https://www.affymetrix.com/LinkServlet?probeset=238068_at) | [ARIH2](http://www.ncbi.nlm.nih.gov/entrez/query.fcgi?cmd=search&db=gene&term=ARIH2) | ariadne homolog 2 (Drosophila) | **1.342** | **110.259** | 33.263 | **147.926** | 45.807 | 3.279E-02 |
| [216290_x_at](https://www.affymetrix.com/LinkServlet?probeset=216290_x_at) | [NA](http://www.ncbi.nlm.nih.gov/entrez/query.fcgi?cmd=search&db=gene&term=NA) | NA | **1.342** | **13.965** | 4.841 | **18.737** | 4.531 | 1.720E-02 |
| [1553361_x_at](https://www.affymetrix.com/LinkServlet?probeset=1553361_x_at) | [FBXL18](http://www.ncbi.nlm.nih.gov/entrez/query.fcgi?cmd=search&db=gene&term=FBXL18) | F-box and leucine-rich repeat protein 18 | **1.342** | **6.559** | 1.217 | **8.805** | 2.626 | 4.814E-03 |
| [229494_s_at](https://www.affymetrix.com/LinkServlet?probeset=229494_s_at) | [PHLDA2](http://www.ncbi.nlm.nih.gov/entrez/query.fcgi?cmd=search&db=gene&term=PHLDA2) | pleckstrin homology-like domain, family A, member 2 | **1.345** | **7.074** | 1.630 | **9.512** | 3.905 | 4.811E-02 |
| [217133_x_at](https://www.affymetrix.com/LinkServlet?probeset=217133_x_at) | [CYP2B6](http://www.ncbi.nlm.nih.gov/entrez/query.fcgi?cmd=search&db=gene&term=CYP2B6) | cytochrome P450, family 2, subfamily B, polypeptide 6 | **1.345** | **6.972** | 1.407 | **9.377** | 3.110 | 1.239E-02 |
| [243287_s_at](https://www.affymetrix.com/LinkServlet?probeset=243287_s_at) | [OSTM1](http://www.ncbi.nlm.nih.gov/entrez/query.fcgi?cmd=search&db=gene&term=OSTM1) | osteopetrosis associated transmembrane protein 1 | **1.345** | **38.441** | 13.048 | **51.721** | 18.735 | 4.397E-02 |
| [206120_at](https://www.affymetrix.com/LinkServlet?probeset=206120_at) | [CD33](http://www.ncbi.nlm.nih.gov/entrez/query.fcgi?cmd=search&db=gene&term=CD33) | CD33 molecule | **1.346** | **282.296** | 146.424 | **380.094** | 104.478 | 3.709E-02 |
| [214805_at](https://www.affymetrix.com/LinkServlet?probeset=214805_at) | [EIF4A1](http://www.ncbi.nlm.nih.gov/entrez/query.fcgi?cmd=search&db=gene&term=EIF4A1) | eukaryotic translation initiation factor 4A1 | **1.352** | **97.065** | 27.208 | **131.263** | 35.850 | 2.214E-02 |
| [244691_at](https://www.affymetrix.com/LinkServlet?probeset=244691_at) | [SETD5](http://www.ncbi.nlm.nih.gov/entrez/query.fcgi?cmd=search&db=gene&term=SETD5) | SET domain containing 5 | **1.353** | **6.076** | 0.213 | **8.220** | 3.868 | 2.226E-02 |
| [209473_at](https://www.affymetrix.com/LinkServlet?probeset=209473_at) | [ENTPD1](http://www.ncbi.nlm.nih.gov/entrez/query.fcgi?cmd=search&db=gene&term=ENTPD1) | ectonucleoside triphosphate diphosphohydrolase 1 | **1.353** | **543.966** | 163.071 | **736.063** | 262.022 | 3.370E-02 |
| [224898_at](https://www.affymetrix.com/LinkServlet?probeset=224898_at) | [WDR26](http://www.ncbi.nlm.nih.gov/entrez/query.fcgi?cmd=search&db=gene&term=WDR26) | WD repeat domain 26 | **1.355** | **98.869** | 44.962 | **133.963** | 51.005 | 4.072E-02 |
| [205068_s_at](https://www.affymetrix.com/LinkServlet?probeset=205068_s_at) | [ARHGAP26](http://www.ncbi.nlm.nih.gov/entrez/query.fcgi?cmd=search&db=gene&term=ARHGAP26) | Rho GTPase activating protein 26 | **1.355** | **200.285** | 36.781 | **271.433** | 83.851 | 5.757E-03 |
| [212625_at](https://www.affymetrix.com/LinkServlet?probeset=212625_at) | [STX10](http://www.ncbi.nlm.nih.gov/entrez/query.fcgi?cmd=search&db=gene&term=STX10) | syntaxin 10 | **1.357** | **112.681** | 25.690 | **152.901** | 37.915 | 3.944E-03 |
| [204306_s_at](https://www.affymetrix.com/LinkServlet?probeset=204306_s_at) | [CD151](http://www.ncbi.nlm.nih.gov/entrez/query.fcgi?cmd=search&db=gene&term=CD151) | CD151 molecule (Raph blood group) | **1.358** | **106.695** | 57.981 | **144.856** | 55.728 | 3.738E-02 |
| [204933_s_at](https://www.affymetrix.com/LinkServlet?probeset=204933_s_at) | [TNFRSF11B](http://www.ncbi.nlm.nih.gov/entrez/query.fcgi?cmd=search&db=gene&term=TNFRSF11B) | tumor necrosis factor receptor superfamily, member 11b | **1.358** | **6.296** | 0.717 | **8.549** | 4.584 | 4.864E-02 |
| [201179_s_at](https://www.affymetrix.com/LinkServlet?probeset=201179_s_at) | [GNAI3](http://www.ncbi.nlm.nih.gov/entrez/query.fcgi?cmd=search&db=gene&term=GNAI3) | guanine nucleotide binding protein (G protein), alpha inhibiting activity polypeptide 3 | **1.358** | **168.412** | 30.422 | **228.746** | 83.931 | 1.039E-02 |
| [213229_at](https://www.affymetrix.com/LinkServlet?probeset=213229_at) | [DICER1](http://www.ncbi.nlm.nih.gov/entrez/query.fcgi?cmd=search&db=gene&term=DICER1) | dicer 1, ribonuclease type III | **1.359** | **251.829** | 125.398 | **342.277** | 106.003 | 4.158E-02 |
| [234994_at](https://www.affymetrix.com/LinkServlet?probeset=234994_at) | [TMEM200A](http://www.ncbi.nlm.nih.gov/entrez/query.fcgi?cmd=search&db=gene&term=TMEM200A) | transmembrane protein 200A | **1.359** | **165.157** | 55.307 | **224.499** | 74.992 | 4.134E-02 |
| [201881_s_at](https://www.affymetrix.com/LinkServlet?probeset=201881_s_at) | [ARIH1](http://www.ncbi.nlm.nih.gov/entrez/query.fcgi?cmd=search&db=gene&term=ARIH1) | ariadne homolog, ubiquitin-conjugating enzyme E2 binding protein, 1 (Drosophila) | **1.360** | **83.242** | 26.242 | **113.174** | 33.284 | 2.325E-02 |
| [225926_at](https://www.affymetrix.com/LinkServlet?probeset=225926_at) | [VTI1B](http://www.ncbi.nlm.nih.gov/entrez/query.fcgi?cmd=search&db=gene&term=VTI1B) | vesicle transport through interaction with t-SNAREs homolog 1B (yeast) | **1.362** | **229.918** | 61.316 | **313.035** | 131.866 | 4.998E-02 |
| [213244_at](https://www.affymetrix.com/LinkServlet?probeset=213244_at) | [SCAMP4](http://www.ncbi.nlm.nih.gov/entrez/query.fcgi?cmd=search&db=gene&term=SCAMP4) | secretory carrier membrane protein 4 | **1.362** | **257.095** | 83.118 | **350.098** | 95.961 | 1.744E-02 |
| [1560770_at](https://www.affymetrix.com/LinkServlet?probeset=1560770_at) | [PABPC1](http://www.ncbi.nlm.nih.gov/entrez/query.fcgi?cmd=search&db=gene&term=PABPC1) | poly(A) binding protein, cytoplasmic 1 | **1.363** | **6.986** | 1.820 | **9.520** | 3.824 | 3.279E-02 |
| [1566266_at](https://www.affymetrix.com/LinkServlet?probeset=1566266_at) | [NA](http://www.ncbi.nlm.nih.gov/entrez/query.fcgi?cmd=search&db=gene&term=NA) | NA | **1.363** | **6.440** | 1.633 | **8.779** | 4.316 | 4.200E-02 |
| [201948_at](https://www.affymetrix.com/LinkServlet?probeset=201948_at) | [GNL2](http://www.ncbi.nlm.nih.gov/entrez/query.fcgi?cmd=search&db=gene&term=GNL2) | guanine nucleotide binding protein-like 2 (nucleolar) | **1.366** | **382.661** | 88.485 | **522.729** | 202.644 | 1.822E-02 |
| [238434_at](https://www.affymetrix.com/LinkServlet?probeset=238434_at) | [SMCR8](http://www.ncbi.nlm.nih.gov/entrez/query.fcgi?cmd=search&db=gene&term=SMCR8) | Smith-Magenis syndrome chromosome region, candidate 8 | **1.367** | **25.759** | 8.487 | **35.225** | 10.882 | 2.504E-02 |
| [1554443_s_at](https://www.affymetrix.com/LinkServlet?probeset=1554443_s_at) | [BEST1](http://www.ncbi.nlm.nih.gov/entrez/query.fcgi?cmd=search&db=gene&term=BEST1) | bestrophin 1 | **1.368** | **15.441** | 18.085 | **21.124** | 9.362 | 2.891E-02 |
| [202862_at](https://www.affymetrix.com/LinkServlet?probeset=202862_at) | [FAH](http://www.ncbi.nlm.nih.gov/entrez/query.fcgi?cmd=search&db=gene&term=FAH) | fumarylacetoacetate hydrolase (fumarylacetoacetase) | **1.370** | **92.820** | 31.750 | **127.187** | 36.156 | 1.172E-02 |
| [1552675_at](https://www.affymetrix.com/LinkServlet?probeset=1552675_at) | [DNAJB7](http://www.ncbi.nlm.nih.gov/entrez/query.fcgi?cmd=search&db=gene&term=DNAJB7) | DnaJ (Hsp40) homolog, subfamily B, member 7 | **1.371** | **6.000** | 0.000 | **8.225** | 4.484 | 4.563E-02 |
| [200724_at](https://www.affymetrix.com/LinkServlet?probeset=200724_at) | [RPL10](http://www.ncbi.nlm.nih.gov/entrez/query.fcgi?cmd=search&db=gene&term=RPL10) | ribosomal protein L10 | **1.372** | **286.336** | 120.256 | **392.767** | 135.407 | 2.530E-02 |
| [211998_at](https://www.affymetrix.com/LinkServlet?probeset=211998_at) | [H3F3B](http://www.ncbi.nlm.nih.gov/entrez/query.fcgi?cmd=search&db=gene&term=H3F3B) | H3 histone, family 3B (H3.3B) | **1.373** | **407.484** | 119.340 | **559.344** | 128.581 | 5.687E-03 |
| [239605_x_at](https://www.affymetrix.com/LinkServlet?probeset=239605_x_at) | [NA](http://www.ncbi.nlm.nih.gov/entrez/query.fcgi?cmd=search&db=gene&term=NA) | NA | **1.374** | **34.041** | 15.976 | **46.768** | 16.973 | 3.766E-02 |
| [204284_at](https://www.affymetrix.com/LinkServlet?probeset=204284_at) | [PPP1R3C](http://www.ncbi.nlm.nih.gov/entrez/query.fcgi?cmd=search&db=gene&term=PPP1R3C) | protein phosphatase 1, regulatory (inhibitor) subunit 3C | **1.374** | **6.013** | 0.050 | **8.265** | 3.361 | 7.722E-03 |
| [220358_at](https://www.affymetrix.com/LinkServlet?probeset=220358_at) | [BATF3](http://www.ncbi.nlm.nih.gov/entrez/query.fcgi?cmd=search&db=gene&term=BATF3) | basic leucine zipper transcription factor, ATF-like 3 | **1.375** | **56.968** | 27.023 | **78.353** | 20.440 | 1.848E-02 |
| [1554800_at](https://www.affymetrix.com/LinkServlet?probeset=1554800_at) | [RAB39](http://www.ncbi.nlm.nih.gov/entrez/query.fcgi?cmd=search&db=gene&term=RAB39) | RAB39, member RAS oncogene family | **1.375** | **16.448** | 10.035 | **22.623** | 10.522 | 4.925E-02 |
| [241259_at](https://www.affymetrix.com/LinkServlet?probeset=241259_at) | [GAB3](http://www.ncbi.nlm.nih.gov/entrez/query.fcgi?cmd=search&db=gene&term=GAB3) | GRB2-associated binding protein 3 | **1.379** | **6.000** | 0.000 | **8.274** | 4.538 | 4.234E-02 |
| [1562664_at](https://www.affymetrix.com/LinkServlet?probeset=1562664_at) | [LOC286009](http://www.ncbi.nlm.nih.gov/entrez/query.fcgi?cmd=search&db=gene&term=LOC286009) | hypothetical protein LOC286009 | **1.384** | **6.237** | 0.869 | **8.633** | 4.200 | 1.400E-02 |
| [237204_at](https://www.affymetrix.com/LinkServlet?probeset=237204_at) | [NA](http://www.ncbi.nlm.nih.gov/entrez/query.fcgi?cmd=search&db=gene&term=NA) | NA | **1.384** | **6.685** | 1.231 | **9.255** | 4.496 | 4.448E-02 |
| [207061_at](https://www.affymetrix.com/LinkServlet?probeset=207061_at) | [ERN1](http://www.ncbi.nlm.nih.gov/entrez/query.fcgi?cmd=search&db=gene&term=ERN1) | endoplasmic reticulum to nucleus signaling 1 | **1.385** | **7.005** | 1.394 | **9.702** | 3.472 | 1.337E-02 |
| [230115_at](https://www.affymetrix.com/LinkServlet?probeset=230115_at) | [DKFZp779M0652](http://www.ncbi.nlm.nih.gov/entrez/query.fcgi?cmd=search&db=gene&term=DKFZp779M0652) | hypothetical DKFZp779M0652 | **1.387** | **6.517** | 1.463 | **9.037** | 2.746 | 3.389E-03 |
| [236841_at](https://www.affymetrix.com/LinkServlet?probeset=236841_at) | [NA](http://www.ncbi.nlm.nih.gov/entrez/query.fcgi?cmd=search&db=gene&term=NA) | NA | **1.387** | **45.772** | 22.779 | **63.480** | 24.046 | 3.879E-02 |
| [231150_at](https://www.affymetrix.com/LinkServlet?probeset=231150_at) | [NA](http://www.ncbi.nlm.nih.gov/entrez/query.fcgi?cmd=search&db=gene&term=NA) | NA | **1.387** | **6.222** | 0.781 | **8.629** | 4.326 | 3.557E-02 |
| [215185_at](https://www.affymetrix.com/LinkServlet?probeset=215185_at) | [LOC100506190](http://www.ncbi.nlm.nih.gov/entrez/query.fcgi?cmd=search&db=gene&term=LOC100506190) | hypothetical LOC100506190 | **1.387** | **61.815** | 28.070 | **85.754** | 33.612 | 4.167E-02 |
| [214013_s_at](https://www.affymetrix.com/LinkServlet?probeset=214013_s_at) | [TBC1D1](http://www.ncbi.nlm.nih.gov/entrez/query.fcgi?cmd=search&db=gene&term=TBC1D1) | TBC1 (tre-2/USP6, BUB2, cdc16) domain family, member 1 | **1.387** | **35.924** | 16.410 | **49.840** | 12.433 | 3.142E-02 |
| [202988_s_at](https://www.affymetrix.com/LinkServlet?probeset=202988_s_at) | [RGS1](http://www.ncbi.nlm.nih.gov/entrez/query.fcgi?cmd=search&db=gene&term=RGS1) | regulator of G-protein signaling 1 | **1.389** | **713.263** | 252.735 | **990.422** | 293.513 | 8.883E-03 |
| [202604_x_at](https://www.affymetrix.com/LinkServlet?probeset=202604_x_at) | [ADAM10](http://www.ncbi.nlm.nih.gov/entrez/query.fcgi?cmd=search&db=gene&term=ADAM10) | ADAM metallopeptidase domain 10 | **1.390** | **81.876** | 28.712 | **113.799** | 53.949 | 2.389E-02 |
| [206289_at](https://www.affymetrix.com/LinkServlet?probeset=206289_at) | [HOXA4](http://www.ncbi.nlm.nih.gov/entrez/query.fcgi?cmd=search&db=gene&term=HOXA4) | homeobox A4 | **1.391** | **6.381** | 1.034 | **8.878** | 5.235 | 4.777E-02 |
| [232034_at](https://www.affymetrix.com/LinkServlet?probeset=232034_at) | [LOC203274](http://www.ncbi.nlm.nih.gov/entrez/query.fcgi?cmd=search&db=gene&term=LOC203274) | hypothetical protein LOC203274 | **1.392** | **60.070** | 34.405 | **83.633** | 28.818 | 3.117E-02 |
| [239466_at](https://www.affymetrix.com/LinkServlet?probeset=239466_at) | [LOC344595](http://www.ncbi.nlm.nih.gov/entrez/query.fcgi?cmd=search&db=gene&term=LOC344595) | hypothetical LOC344595 | **1.397** | **6.051** | 0.171 | **8.453** | 3.584 | 1.148E-02 |
| [200722_s_at](https://www.affymetrix.com/LinkServlet?probeset=200722_s_at) | [CAPRIN1](http://www.ncbi.nlm.nih.gov/entrez/query.fcgi?cmd=search&db=gene&term=CAPRIN1) | cell cycle associated protein 1 | **1.397** | **47.398** | 11.879 | **66.219** | 19.403 | 1.957E-02 |
| [227296_at](https://www.affymetrix.com/LinkServlet?probeset=227296_at) | [MFSD3](http://www.ncbi.nlm.nih.gov/entrez/query.fcgi?cmd=search&db=gene&term=MFSD3) | major facilitator superfamily domain containing 3 | **1.398** | **20.459** | 7.866 | **28.603** | 11.391 | 4.870E-02 |
| [243887_at](https://www.affymetrix.com/LinkServlet?probeset=243887_at) | [NA](http://www.ncbi.nlm.nih.gov/entrez/query.fcgi?cmd=search&db=gene&term=NA) | NA | **1.398** | **109.785** | 34.094 | **153.513** | 67.853 | 3.965E-02 |
| [235197_s_at](https://www.affymetrix.com/LinkServlet?probeset=235197_s_at) | [OSTM1](http://www.ncbi.nlm.nih.gov/entrez/query.fcgi?cmd=search&db=gene&term=OSTM1) | osteopetrosis associated transmembrane protein 1 | **1.400** | **69.720** | 32.263 | **97.598** | 38.475 | 4.231E-02 |
| [1552735_at](https://www.affymetrix.com/LinkServlet?probeset=1552735_at) | [NA](http://www.ncbi.nlm.nih.gov/entrez/query.fcgi?cmd=search&db=gene&term=NA) | NA | **1.401** | **6.000** | 0.000 | **8.403** | 4.121 | 1.682E-02 |
| [235308_at](https://www.affymetrix.com/LinkServlet?probeset=235308_at) | [ZBTB20](http://www.ncbi.nlm.nih.gov/entrez/query.fcgi?cmd=search&db=gene&term=ZBTB20) | zinc finger and BTB domain containing 20 | **1.402** | **48.979** | 27.660 | **68.670** | 22.712 | 4.779E-02 |
| [241454_at](https://www.affymetrix.com/LinkServlet?probeset=241454_at) | [NA](http://www.ncbi.nlm.nih.gov/entrez/query.fcgi?cmd=search&db=gene&term=NA) | NA | **1.403** | **6.115** | 0.357 | **8.582** | 4.528 | 3.465E-02 |
| [215592_at](https://www.affymetrix.com/LinkServlet?probeset=215592_at) | [NA](http://www.ncbi.nlm.nih.gov/entrez/query.fcgi?cmd=search&db=gene&term=NA) | NA | **1.404** | **14.707** | 8.158 | **20.650** | 7.529 | 3.601E-02 |
| [219170_at](https://www.affymetrix.com/LinkServlet?probeset=219170_at) | [FSD1](http://www.ncbi.nlm.nih.gov/entrez/query.fcgi?cmd=search&db=gene&term=FSD1) | fibronectin type III and SPRY domain containing 1 | **1.404** | **6.059** | 0.208 | **8.507** | 3.596 | 5.958E-03 |
| [233056_x_at](https://www.affymetrix.com/LinkServlet?probeset=233056_x_at) | [DLGAP4](http://www.ncbi.nlm.nih.gov/entrez/query.fcgi?cmd=search&db=gene&term=DLGAP4) | discs, large (Drosophila) homolog-associated protein 4 | **1.405** | **46.814** | 11.707 | **65.782** | 32.702 | 3.821E-02 |
| [222720_x_at](https://www.affymetrix.com/LinkServlet?probeset=222720_x_at) | [C1orf27](http://www.ncbi.nlm.nih.gov/entrez/query.fcgi?cmd=search&db=gene&term=C1orf27) | chromosome 1 open reading frame 27 | **1.406** | **125.730** | 43.273 | **176.764** | 53.329 | 2.440E-02 |
| [221165_s_at](https://www.affymetrix.com/LinkServlet?probeset=221165_s_at) | [IL22](http://www.ncbi.nlm.nih.gov/entrez/query.fcgi?cmd=search&db=gene&term=IL22) | interleukin 22 | **1.407** | **6.117** | 0.466 | **8.609** | 4.402 | 2.000E-02 |
| [224374_s_at](https://www.affymetrix.com/LinkServlet?probeset=224374_s_at) | [EMILIN2](http://www.ncbi.nlm.nih.gov/entrez/query.fcgi?cmd=search&db=gene&term=EMILIN2) | elastin microfibril interfacer 2 | **1.408** | **403.773** | 210.673 | **568.390** | 167.495 | 2.543E-02 |
| [206467_x_at](https://www.affymetrix.com/LinkServlet?probeset=206467_x_at) | [NA](http://www.ncbi.nlm.nih.gov/entrez/query.fcgi?cmd=search&db=gene&term=NA) | NA | **1.409** | **8.386** | 3.057 | **11.817** | 4.846 | 3.850E-02 |
| [243355_at](https://www.affymetrix.com/LinkServlet?probeset=243355_at) | [NA](http://www.ncbi.nlm.nih.gov/entrez/query.fcgi?cmd=search&db=gene&term=NA) | NA | **1.411** | **6.152** | 0.440 | **8.679** | 3.953 | 8.221E-03 |
| [210513_s_at](https://www.affymetrix.com/LinkServlet?probeset=210513_s_at) | [VEGFA](http://www.ncbi.nlm.nih.gov/entrez/query.fcgi?cmd=search&db=gene&term=VEGFA) | vascular endothelial growth factor A | **1.411** | **7.331** | 1.639 | **10.343** | 4.518 | 1.947E-02 |
| [228664_at](https://www.affymetrix.com/LinkServlet?probeset=228664_at) | [NA](http://www.ncbi.nlm.nih.gov/entrez/query.fcgi?cmd=search&db=gene&term=NA) | NA | **1.412** | **45.190** | 15.505 | **63.829** | 16.409 | 3.244E-02 |
| [217738_at](https://www.affymetrix.com/LinkServlet?probeset=217738_at) | [NAMPT](http://www.ncbi.nlm.nih.gov/entrez/query.fcgi?cmd=search&db=gene&term=NAMPT) | nicotinamide phosphoribosyltransferase | **1.414** | **213.417** | 122.697 | **301.774** | 130.054 | 3.515E-02 |
| [235198_at](https://www.affymetrix.com/LinkServlet?probeset=235198_at) | [OSTM1](http://www.ncbi.nlm.nih.gov/entrez/query.fcgi?cmd=search&db=gene&term=OSTM1) | osteopetrosis associated transmembrane protein 1 | **1.415** | **71.937** | 38.166 | **101.756** | 32.489 | 1.327E-02 |
| [206785_s_at](https://www.affymetrix.com/LinkServlet?probeset=206785_s_at) | [NA](http://www.ncbi.nlm.nih.gov/entrez/query.fcgi?cmd=search&db=gene&term=NA) | NA | **1.415** | **301.917** | 207.804 | **427.228** | 91.630 | 1.784E-02 |
| [222403_at](https://www.affymetrix.com/LinkServlet?probeset=222403_at) | [MTCH2](http://www.ncbi.nlm.nih.gov/entrez/query.fcgi?cmd=search&db=gene&term=MTCH2) | mitochondrial carrier homolog 2 (C. elegans) | **1.416** | **303.721** | 97.383 | **429.954** | 176.969 | 2.192E-02 |
| [235553_at](https://www.affymetrix.com/LinkServlet?probeset=235553_at) | [GAPVD1](http://www.ncbi.nlm.nih.gov/entrez/query.fcgi?cmd=search&db=gene&term=GAPVD1) | GTPase activating protein and VPS9 domains 1 | **1.417** | **123.120** | 58.138 | **174.466** | 39.661 | 3.224E-02 |
| [215837_x_at](https://www.affymetrix.com/LinkServlet?probeset=215837_x_at) | [NA](http://www.ncbi.nlm.nih.gov/entrez/query.fcgi?cmd=search&db=gene&term=NA) | NA | **1.418** | **6.660** | 1.438 | **9.442** | 5.122 | 4.498E-02 |
| [1556480_a_at](https://www.affymetrix.com/LinkServlet?probeset=1556480_a_at) | [NA](http://www.ncbi.nlm.nih.gov/entrez/query.fcgi?cmd=search&db=gene&term=NA) | NA | **1.420** | **6.394** | 0.749 | **9.079** | 5.443 | 3.622E-02 |
| [1570051_at](https://www.affymetrix.com/LinkServlet?probeset=1570051_at) | [RNF144A](http://www.ncbi.nlm.nih.gov/entrez/query.fcgi?cmd=search&db=gene&term=RNF144A) | ring finger protein 144A | **1.421** | **6.082** | 0.329 | **8.644** | 5.143 | 4.600E-02 |
| [210753_s_at](https://www.affymetrix.com/LinkServlet?probeset=210753_s_at) | [EPHB1](http://www.ncbi.nlm.nih.gov/entrez/query.fcgi?cmd=search&db=gene&term=EPHB1) | EPH receptor B1 | **1.423** | **6.816** | 1.403 | **9.697** | 4.156 | 1.674E-02 |
| [212906_at](https://www.affymetrix.com/LinkServlet?probeset=212906_at) | [GRAMD1B](http://www.ncbi.nlm.nih.gov/entrez/query.fcgi?cmd=search&db=gene&term=GRAMD1B) | GRAM domain containing 1B | **1.423** | **43.060** | 15.556 | **61.265** | 12.535 | 6.537E-03 |
| [232143_at](https://www.affymetrix.com/LinkServlet?probeset=232143_at) | [DNM1P41](http://www.ncbi.nlm.nih.gov/entrez/query.fcgi?cmd=search&db=gene&term=DNM1P41) | DNM1 pseudogene 41 | **1.423** | **6.458** | 0.966 | **9.190** | 2.706 | 7.002E-04 |
| [202878_s_at](https://www.affymetrix.com/LinkServlet?probeset=202878_s_at) | [CD93](http://www.ncbi.nlm.nih.gov/entrez/query.fcgi?cmd=search&db=gene&term=CD93) | CD93 molecule | **1.425** | **495.107** | 211.316 | **705.493** | 243.725 | 3.102E-02 |
| [237622_at](https://www.affymetrix.com/LinkServlet?probeset=237622_at) | [NA](http://www.ncbi.nlm.nih.gov/entrez/query.fcgi?cmd=search&db=gene&term=NA) | NA | **1.426** | **6.000** | 0.000 | **8.559** | 5.279 | 4.567E-02 |
| [244504_x_at](https://www.affymetrix.com/LinkServlet?probeset=244504_x_at) | [ARF1](http://www.ncbi.nlm.nih.gov/entrez/query.fcgi?cmd=search&db=gene&term=ARF1) | ADP-ribosylation factor 1 | **1.426** | **7.062** | 2.213 | **10.073** | 3.842 | 1.709E-02 |
| [238613_at](https://www.affymetrix.com/LinkServlet?probeset=238613_at) | [ZAK](http://www.ncbi.nlm.nih.gov/entrez/query.fcgi?cmd=search&db=gene&term=ZAK) | sterile alpha motif and leucine zipper containing kinase AZK | **1.427** | **21.567** | 10.756 | **30.771** | 10.787 | 3.946E-02 |
| [226728_at](https://www.affymetrix.com/LinkServlet?probeset=226728_at) | [SLC27A1](http://www.ncbi.nlm.nih.gov/entrez/query.fcgi?cmd=search&db=gene&term=SLC27A1) | solute carrier family 27 (fatty acid transporter), member 1 | **1.427** | **97.687** | 53.454 | **139.427** | 38.780 | 2.748E-02 |
| [206747_at](https://www.affymetrix.com/LinkServlet?probeset=206747_at) | [GPRIN2](http://www.ncbi.nlm.nih.gov/entrez/query.fcgi?cmd=search&db=gene&term=GPRIN2) | G protein regulated inducer of neurite outgrowth 2 | **1.428** | **6.660** | 1.124 | **9.511** | 4.767 | 2.303E-02 |
| [239472_at](https://www.affymetrix.com/LinkServlet?probeset=239472_at) | [NA](http://www.ncbi.nlm.nih.gov/entrez/query.fcgi?cmd=search&db=gene&term=NA) | NA | **1.428** | **8.337** | 2.630 | **11.907** | 6.584 | 4.922E-02 |
| [203912_s_at](https://www.affymetrix.com/LinkServlet?probeset=203912_s_at) | [DNASE1L1](http://www.ncbi.nlm.nih.gov/entrez/query.fcgi?cmd=search&db=gene&term=DNASE1L1) | deoxyribonuclease I-like 1 | **1.432** | **367.699** | 118.534 | **526.429** | 206.298 | 1.249E-02 |
| [235967_at](https://www.affymetrix.com/LinkServlet?probeset=235967_at) | [LOC100127891](http://www.ncbi.nlm.nih.gov/entrez/query.fcgi?cmd=search&db=gene&term=LOC100127891) | hypothetical LOC100127891 | **1.433** | **7.811** | 2.155 | **11.196** | 5.221 | 3.922E-02 |
| [224549_x_at](https://www.affymetrix.com/LinkServlet?probeset=224549_x_at) | [NA](http://www.ncbi.nlm.nih.gov/entrez/query.fcgi?cmd=search&db=gene&term=NA) | NA | **1.434** | **655.052** | 219.840 | **939.579** | 409.534 | 1.507E-02 |
| [1561539_at](https://www.affymetrix.com/LinkServlet?probeset=1561539_at) | [LOC100506368](http://www.ncbi.nlm.nih.gov/entrez/query.fcgi?cmd=search&db=gene&term=LOC100506368) | hypothetical LOC100506368 | **1.434** | **6.136** | 0.543 | **8.802** | 5.059 | 2.127E-02 |
| [209311_at](https://www.affymetrix.com/LinkServlet?probeset=209311_at) | [BCL2L2](http://www.ncbi.nlm.nih.gov/entrez/query.fcgi?cmd=search&db=gene&term=BCL2L2) | BCL2-like 2 | **1.435** | **56.305** | 32.013 | **80.778** | 30.834 | 3.349E-02 |
| [243931_at](https://www.affymetrix.com/LinkServlet?probeset=243931_at) | [NA](http://www.ncbi.nlm.nih.gov/entrez/query.fcgi?cmd=search&db=gene&term=NA) | NA | **1.435** | **178.284** | 56.692 | **255.829** | 49.843 | 1.029E-02 |
| [226195_at](https://www.affymetrix.com/LinkServlet?probeset=226195_at) | [C14orf179](http://www.ncbi.nlm.nih.gov/entrez/query.fcgi?cmd=search&db=gene&term=C14orf179) | chromosome 14 open reading frame 179 | **1.435** | **105.779** | 31.279 | **151.839** | 67.069 | 1.858E-02 |
| [228607_at](https://www.affymetrix.com/LinkServlet?probeset=228607_at) | [OAS2](http://www.ncbi.nlm.nih.gov/entrez/query.fcgi?cmd=search&db=gene&term=OAS2) | 2'-5'-oligoadenylate synthetase 2, 69/71kDa | **1.436** | **123.481** | 49.658 | **177.354** | 49.233 | 1.022E-02 |
| [244466_at](https://www.affymetrix.com/LinkServlet?probeset=244466_at) | [ZNF544](http://www.ncbi.nlm.nih.gov/entrez/query.fcgi?cmd=search&db=gene&term=ZNF544) | zinc finger protein 544 | **1.437** | **6.236** | 0.442 | **8.962** | 5.157 | 3.922E-02 |
| [227138_at](https://www.affymetrix.com/LinkServlet?probeset=227138_at) | [CRTAP](http://www.ncbi.nlm.nih.gov/entrez/query.fcgi?cmd=search&db=gene&term=CRTAP) | cartilage associated protein | **1.440** | **94.563** | 37.633 | **136.134** | 56.656 | 3.298E-02 |
| [1557465_at](https://www.affymetrix.com/LinkServlet?probeset=1557465_at) | [NCRNA00282](http://www.ncbi.nlm.nih.gov/entrez/query.fcgi?cmd=search&db=gene&term=NCRNA00282) | non-protein coding RNA 282 | **1.441** | **8.279** | 4.775 | **11.934** | 5.238 | 3.664E-02 |
| [211474_s_at](https://www.affymetrix.com/LinkServlet?probeset=211474_s_at) | [SERPINB6](http://www.ncbi.nlm.nih.gov/entrez/query.fcgi?cmd=search&db=gene&term=SERPINB6) | serpin peptidase inhibitor, clade B (ovalbumin), member 6 | **1.442** | **421.986** | 141.092 | **608.644** | 257.380 | 4.321E-02 |
| [1566784_at](https://www.affymetrix.com/LinkServlet?probeset=1566784_at) | [NSF](http://www.ncbi.nlm.nih.gov/entrez/query.fcgi?cmd=search&db=gene&term=NSF) | N-ethylmaleimide-sensitive factor | **1.444** | **6.000** | 0.000 | **8.662** | 4.378 | 1.188E-02 |
| [243615_at](https://www.affymetrix.com/LinkServlet?probeset=243615_at) | [NA](http://www.ncbi.nlm.nih.gov/entrez/query.fcgi?cmd=search&db=gene&term=NA) | NA | **1.444** | **15.698** | 7.179 | **22.666** | 8.758 | 2.910E-02 |
| [1554393_a_at](https://www.affymetrix.com/LinkServlet?probeset=1554393_a_at) | [ADC](http://www.ncbi.nlm.nih.gov/entrez/query.fcgi?cmd=search&db=gene&term=ADC) | arginine decarboxylase | **1.446** | **6.732** | 1.565 | **9.732** | 4.719 | 2.114E-02 |
| [208392_x_at](https://www.affymetrix.com/LinkServlet?probeset=208392_x_at) | [SP110](http://www.ncbi.nlm.nih.gov/entrez/query.fcgi?cmd=search&db=gene&term=SP110) | SP110 nuclear body protein | **1.447** | **66.022** | 31.265 | **95.539** | 31.293 | 2.598E-02 |
| [224254_x_at](https://www.affymetrix.com/LinkServlet?probeset=224254_x_at) | [NA](http://www.ncbi.nlm.nih.gov/entrez/query.fcgi?cmd=search&db=gene&term=NA) | NA | **1.448** | **57.501** | 31.000 | **83.242** | 34.461 | 2.736E-02 |
| [235579_at](https://www.affymetrix.com/LinkServlet?probeset=235579_at) | [SCAF11](http://www.ncbi.nlm.nih.gov/entrez/query.fcgi?cmd=search&db=gene&term=SCAF11) | SR-related CTD-associated factor 11 | **1.448** | **25.396** | 10.460 | **36.768** | 15.320 | 4.158E-02 |
| [212605_s_at](https://www.affymetrix.com/LinkServlet?probeset=212605_s_at) | [NA](http://www.ncbi.nlm.nih.gov/entrez/query.fcgi?cmd=search&db=gene&term=NA) | NA | **1.448** | **60.021** | 26.540 | **86.939** | 34.217 | 4.731E-02 |
| [209078_s_at](https://www.affymetrix.com/LinkServlet?probeset=209078_s_at) | [TXN2](http://www.ncbi.nlm.nih.gov/entrez/query.fcgi?cmd=search&db=gene&term=TXN2) | thioredoxin 2 | **1.451** | **179.663** | 60.131 | **260.630** | 108.142 | 3.226E-02 |
| [238757_at](https://www.affymetrix.com/LinkServlet?probeset=238757_at) | [DBF4B](http://www.ncbi.nlm.nih.gov/entrez/query.fcgi?cmd=search&db=gene&term=DBF4B) | DBF4 homolog B (S. cerevisiae) | **1.452** | **7.475** | 2.558 | **10.856** | 4.350 | 1.963E-02 |
| [227056_at](https://www.affymetrix.com/LinkServlet?probeset=227056_at) | [KIAA0141](http://www.ncbi.nlm.nih.gov/entrez/query.fcgi?cmd=search&db=gene&term=KIAA0141) | KIAA0141 | **1.454** | **114.149** | 30.386 | **165.970** | 75.861 | 1.831E-02 |
| [241724_x_at](https://www.affymetrix.com/LinkServlet?probeset=241724_x_at) | [NA](http://www.ncbi.nlm.nih.gov/entrez/query.fcgi?cmd=search&db=gene&term=NA) | NA | **1.454** | **14.357** | 5.201 | **20.881** | 6.992 | 1.173E-02 |
| [202388_at](https://www.affymetrix.com/LinkServlet?probeset=202388_at) | [RGS2](http://www.ncbi.nlm.nih.gov/entrez/query.fcgi?cmd=search&db=gene&term=RGS2) | regulator of G-protein signaling 2, 24kDa | **1.456** | **1276.461** | 532.507 | **1858.390** | 626.625 | 1.431E-02 |
| [207069_s_at](https://www.affymetrix.com/LinkServlet?probeset=207069_s_at) | [SMAD6](http://www.ncbi.nlm.nih.gov/entrez/query.fcgi?cmd=search&db=gene&term=SMAD6) | SMAD family member 6 | **1.457** | **7.640** | 5.140 | **11.134** | 6.355 | 4.295E-02 |
| [236803_at](https://www.affymetrix.com/LinkServlet?probeset=236803_at) | [NA](http://www.ncbi.nlm.nih.gov/entrez/query.fcgi?cmd=search&db=gene&term=NA) | NA | **1.461** | **6.111** | 0.445 | **8.929** | 5.183 | 3.510E-02 |
| [207316_at](https://www.affymetrix.com/LinkServlet?probeset=207316_at) | [HAS1](http://www.ncbi.nlm.nih.gov/entrez/query.fcgi?cmd=search&db=gene&term=HAS1) | hyaluronan synthase 1 | **1.463** | **6.928** | 2.161 | **10.134** | 4.130 | 1.392E-02 |
| [1553088_a_at](https://www.affymetrix.com/LinkServlet?probeset=1553088_a_at) | [BCL2L11](http://www.ncbi.nlm.nih.gov/entrez/query.fcgi?cmd=search&db=gene&term=BCL2L11) | BCL2-like 11 (apoptosis facilitator) | **1.464** | **6.087** | 0.188 | **8.911** | 5.210 | 2.896E-02 |
| [209356_x_at](https://www.affymetrix.com/LinkServlet?probeset=209356_x_at) | [EFEMP2](http://www.ncbi.nlm.nih.gov/entrez/query.fcgi?cmd=search&db=gene&term=EFEMP2) | EGF containing fibulin-like extracellular matrix protein 2 | **1.464** | **15.059** | 10.961 | **22.050** | 8.264 | 2.620E-02 |
| [223279_s_at](https://www.affymetrix.com/LinkServlet?probeset=223279_s_at) | [UACA](http://www.ncbi.nlm.nih.gov/entrez/query.fcgi?cmd=search&db=gene&term=UACA) | uveal autoantigen with coiled-coil domains and ankyrin repeats | **1.465** | **21.740** | 21.555 | **31.842** | 10.000 | 1.431E-02 |
| [1552618_at](https://www.affymetrix.com/LinkServlet?probeset=1552618_at) | [STX6](http://www.ncbi.nlm.nih.gov/entrez/query.fcgi?cmd=search&db=gene&term=STX6) | syntaxin 6 | **1.466** | **34.237** | 11.368 | **50.181** | 19.641 | 2.000E-02 |
| [205878_at](https://www.affymetrix.com/LinkServlet?probeset=205878_at) | [POU6F1](http://www.ncbi.nlm.nih.gov/entrez/query.fcgi?cmd=search&db=gene&term=POU6F1) | POU class 6 homeobox 1 | **1.467** | **92.850** | 37.847 | **136.206** | 53.736 | 4.282E-02 |
| [218394_at](https://www.affymetrix.com/LinkServlet?probeset=218394_at) | [ROGDI](http://www.ncbi.nlm.nih.gov/entrez/query.fcgi?cmd=search&db=gene&term=ROGDI) | rogdi homolog (Drosophila) | **1.468** | **74.592** | 38.502 | **109.504** | 32.829 | 3.370E-02 |
| [216135_at](https://www.affymetrix.com/LinkServlet?probeset=216135_at) | [IQCK](http://www.ncbi.nlm.nih.gov/entrez/query.fcgi?cmd=search&db=gene&term=IQCK) | IQ motif containing K | **1.469** | **6.128** | 0.372 | **9.004** | 4.548 | 1.409E-02 |
| [232011_s_at](https://www.affymetrix.com/LinkServlet?probeset=232011_s_at) | [MAP1LC3A](http://www.ncbi.nlm.nih.gov/entrez/query.fcgi?cmd=search&db=gene&term=MAP1LC3A) | microtubule-associated protein 1 light chain 3 alpha | **1.470** | **7.741** | 1.943 | **11.383** | 4.344 | 6.206E-03 |
| [242196_at](https://www.affymetrix.com/LinkServlet?probeset=242196_at) | [ARHGAP32](http://www.ncbi.nlm.nih.gov/entrez/query.fcgi?cmd=search&db=gene&term=ARHGAP32) | Rho GTPase activating protein 32 | **1.472** | **6.092** | 0.366 | **8.965** | 5.283 | 2.048E-02 |
| [225080_at](https://www.affymetrix.com/LinkServlet?probeset=225080_at) | [MYO1C](http://www.ncbi.nlm.nih.gov/entrez/query.fcgi?cmd=search&db=gene&term=MYO1C) | myosin IC | **1.472** | **70.738** | 20.643 | **104.134** | 34.722 | 4.853E-03 |
| [238410_x_at](https://www.affymetrix.com/LinkServlet?probeset=238410_x_at) | [NA](http://www.ncbi.nlm.nih.gov/entrez/query.fcgi?cmd=search&db=gene&term=NA) | NA | **1.474** | **6.000** | 0.000 | **8.843** | 4.795 | 9.901E-03 |
| [208868_s_at](https://www.affymetrix.com/LinkServlet?probeset=208868_s_at) | [GABARAPL1](http://www.ncbi.nlm.nih.gov/entrez/query.fcgi?cmd=search&db=gene&term=GABARAPL1) | GABA(A) receptor-associated protein like 1 | **1.479** | **82.684** | 37.357 | **122.250** | 37.854 | 1.506E-02 |
| [230096_at](https://www.affymetrix.com/LinkServlet?probeset=230096_at) | [NA](http://www.ncbi.nlm.nih.gov/entrez/query.fcgi?cmd=search&db=gene&term=NA) | NA | **1.479** | **54.802** | 32.225 | **81.073** | 39.252 | 4.472E-02 |
| [240137_at](https://www.affymetrix.com/LinkServlet?probeset=240137_at) | [NA](http://www.ncbi.nlm.nih.gov/entrez/query.fcgi?cmd=search&db=gene&term=NA) | NA | **1.479** | **6.621** | 2.048 | **9.796** | 4.356 | 1.068E-02 |
| [1554292_a_at](https://www.affymetrix.com/LinkServlet?probeset=1554292_a_at) | [UHRF1BP1L](http://www.ncbi.nlm.nih.gov/entrez/query.fcgi?cmd=search&db=gene&term=UHRF1BP1L) | UHRF1 binding protein 1-like | **1.480** | **6.247** | 0.642 | **9.244** | 4.700 | 8.663E-03 |
| [226282_at](https://www.affymetrix.com/LinkServlet?probeset=226282_at) | [PTPN14](http://www.ncbi.nlm.nih.gov/entrez/query.fcgi?cmd=search&db=gene&term=PTPN14) | protein tyrosine phosphatase, non-receptor type 14 | **1.480** | **6.405** | 0.787 | **9.480** | 5.629 | 2.377E-02 |
| [1555691_a_at](https://www.affymetrix.com/LinkServlet?probeset=1555691_a_at) | [NA](http://www.ncbi.nlm.nih.gov/entrez/query.fcgi?cmd=search&db=gene&term=NA) | NA | **1.481** | **330.461** | 132.692 | **489.357** | 244.014 | 2.976E-02 |
| [216230_x_at](https://www.affymetrix.com/LinkServlet?probeset=216230_x_at) | [SMPD1](http://www.ncbi.nlm.nih.gov/entrez/query.fcgi?cmd=search&db=gene&term=SMPD1) | sphingomyelin phosphodiesterase 1, acid lysosomal | **1.483** | **17.750** | 6.678 | **26.316** | 4.326 | 1.154E-03 |
| [223702_x_at](https://www.affymetrix.com/LinkServlet?probeset=223702_x_at) | [FTCD](http://www.ncbi.nlm.nih.gov/entrez/query.fcgi?cmd=search&db=gene&term=FTCD) | formiminotransferase cyclodeaminase | **1.483** | **6.438** | 0.999 | **9.547** | 5.787 | 2.777E-02 |
| [1556224_a_at](https://www.affymetrix.com/LinkServlet?probeset=1556224_a_at) | [LOC155060](http://www.ncbi.nlm.nih.gov/entrez/query.fcgi?cmd=search&db=gene&term=LOC155060) | AI894139 pseudogene | **1.483** | **7.912** | 2.976 | **11.732** | 5.658 | 4.369E-02 |
| [204959_at](https://www.affymetrix.com/LinkServlet?probeset=204959_at) | [MNDA](http://www.ncbi.nlm.nih.gov/entrez/query.fcgi?cmd=search&db=gene&term=MNDA) | myeloid cell nuclear differentiation antigen | **1.483** | **430.903** | 210.399 | **639.030** | 241.722 | 4.011E-02 |
| [230207_s_at](https://www.affymetrix.com/LinkServlet?probeset=230207_s_at) | [DOCK5](http://www.ncbi.nlm.nih.gov/entrez/query.fcgi?cmd=search&db=gene&term=DOCK5) | dedicator of cytokinesis 5 | **1.483** | **298.293** | 185.513 | **442.500** | 188.561 | 4.690E-02 |
| [243483_at](https://www.affymetrix.com/LinkServlet?probeset=243483_at) | [TRPM8](http://www.ncbi.nlm.nih.gov/entrez/query.fcgi?cmd=search&db=gene&term=TRPM8) | transient receptor potential cation channel, subfamily M, member 8 | **1.485** | **6.037** | 0.101 | **8.964** | 5.629 | 3.871E-02 |
| [230150_at](https://www.affymetrix.com/LinkServlet?probeset=230150_at) | [BCAP29](http://www.ncbi.nlm.nih.gov/entrez/query.fcgi?cmd=search&db=gene&term=BCAP29) | B-cell receptor-associated protein 29 | **1.486** | **76.961** | 31.869 | **114.339** | 51.427 | 4.016E-02 |
| [223452_s_at](https://www.affymetrix.com/LinkServlet?probeset=223452_s_at) | [ATL3](http://www.ncbi.nlm.nih.gov/entrez/query.fcgi?cmd=search&db=gene&term=ATL3) | atlastin GTPase 3 | **1.486** | **42.652** | 21.267 | **63.391** | 29.211 | 1.952E-02 |
| [242236_at](https://www.affymetrix.com/LinkServlet?probeset=242236_at) | [NA](http://www.ncbi.nlm.nih.gov/entrez/query.fcgi?cmd=search&db=gene&term=NA) | NA | **1.486** | **6.073** | 0.292 | **9.027** | 4.478 | 1.285E-02 |
| [1556650_at](https://www.affymetrix.com/LinkServlet?probeset=1556650_at) | [NA](http://www.ncbi.nlm.nih.gov/entrez/query.fcgi?cmd=search&db=gene&term=NA) | NA | **1.487** | **6.046** | 0.184 | **8.992** | 5.128 | 2.055E-02 |
| [201728_s_at](https://www.affymetrix.com/LinkServlet?probeset=201728_s_at) | [KIAA0100](http://www.ncbi.nlm.nih.gov/entrez/query.fcgi?cmd=search&db=gene&term=KIAA0100) | KIAA0100 | **1.489** | **110.322** | 45.068 | **164.257** | 58.084 | 1.262E-02 |
| [231109_at](https://www.affymetrix.com/LinkServlet?probeset=231109_at) | [NA](http://www.ncbi.nlm.nih.gov/entrez/query.fcgi?cmd=search&db=gene&term=NA) | NA | **1.490** | **81.481** | 56.056 | **121.416** | 50.388 | 4.650E-02 |
| [1555724_s_at](https://www.affymetrix.com/LinkServlet?probeset=1555724_s_at) | [TAGLN](http://www.ncbi.nlm.nih.gov/entrez/query.fcgi?cmd=search&db=gene&term=TAGLN) | transgelin | **1.492** | **20.498** | 6.678 | **30.578** | 11.494 | 1.498E-02 |
| [204148_s_at](https://www.affymetrix.com/LinkServlet?probeset=204148_s_at) | [NA](http://www.ncbi.nlm.nih.gov/entrez/query.fcgi?cmd=search&db=gene&term=NA) | NA | **1.496** | **127.647** | 91.459 | **190.901** | 67.764 | 3.044E-02 |
| [223717_s_at](https://www.affymetrix.com/LinkServlet?probeset=223717_s_at) | [ACRBP](http://www.ncbi.nlm.nih.gov/entrez/query.fcgi?cmd=search&db=gene&term=ACRBP) | acrosin binding protein | **1.497** | **36.506** | 20.014 | **54.632** | 24.037 | 4.763E-02 |
| [228369_at](https://www.affymetrix.com/LinkServlet?probeset=228369_at) | [CNPY3](http://www.ncbi.nlm.nih.gov/entrez/query.fcgi?cmd=search&db=gene&term=CNPY3) | canopy 3 homolog (zebrafish) | **1.498** | **85.007** | 25.895 | **127.310** | 45.558 | 6.250E-03 |
| [235207_at](https://www.affymetrix.com/LinkServlet?probeset=235207_at) | [NA](http://www.ncbi.nlm.nih.gov/entrez/query.fcgi?cmd=search&db=gene&term=NA) | NA | **1.500** | **95.988** | 35.946 | **143.950** | 50.142 | 2.148E-02 |
| [1552761_at](https://www.affymetrix.com/LinkServlet?probeset=1552761_at) | [SLC16A11](http://www.ncbi.nlm.nih.gov/entrez/query.fcgi?cmd=search&db=gene&term=SLC16A11) | solute carrier family 16, member 11 (monocarboxylic acid transporter 11) | **1.501** | **6.170** | 0.523 | **9.263** | 5.084 | 7.952E-03 |
| [228922_at](https://www.affymetrix.com/LinkServlet?probeset=228922_at) | [SHF](http://www.ncbi.nlm.nih.gov/entrez/query.fcgi?cmd=search&db=gene&term=SHF) | Src homology 2 domain containing F | **1.502** | **9.114** | 3.926 | **13.694** | 4.600 | 8.149E-03 |
| [226556_at](https://www.affymetrix.com/LinkServlet?probeset=226556_at) | [NA](http://www.ncbi.nlm.nih.gov/entrez/query.fcgi?cmd=search&db=gene&term=NA) | NA | **1.503** | **98.900** | 30.423 | **148.619** | 51.563 | 2.978E-03 |
| [205657_at](https://www.affymetrix.com/LinkServlet?probeset=205657_at) | [HAAO](http://www.ncbi.nlm.nih.gov/entrez/query.fcgi?cmd=search&db=gene&term=HAAO) | 3-hydroxyanthranilate 3,4-dioxygenase | **1.503** | **13.384** | 7.756 | **20.122** | 8.544 | 3.664E-02 |
| [211447_s_at](https://www.affymetrix.com/LinkServlet?probeset=211447_s_at) | [PDE4A](http://www.ncbi.nlm.nih.gov/entrez/query.fcgi?cmd=search&db=gene&term=PDE4A) | phosphodiesterase 4A, cAMP-specific | **1.505** | **10.432** | 3.438 | **15.695** | 7.997 | 3.099E-02 |
| [210835_s_at](https://www.affymetrix.com/LinkServlet?probeset=210835_s_at) | [CTBP2](http://www.ncbi.nlm.nih.gov/entrez/query.fcgi?cmd=search&db=gene&term=CTBP2) | C-terminal binding protein 2 | **1.505** | **184.599** | 83.151 | **277.756** | 87.123 | 1.613E-02 |
| [1565723_at](https://www.affymetrix.com/LinkServlet?probeset=1565723_at) | [LOC100128281](http://www.ncbi.nlm.nih.gov/entrez/query.fcgi?cmd=search&db=gene&term=LOC100128281) | hypothetical protein LOC100128281 | **1.506** | **7.416** | 2.419 | **11.170** | 6.507 | 4.290E-02 |
| [205334_at](https://www.affymetrix.com/LinkServlet?probeset=205334_at) | [S100A1](http://www.ncbi.nlm.nih.gov/entrez/query.fcgi?cmd=search&db=gene&term=S100A1) | S100 calcium binding protein A1 | **1.507** | **9.263** | 4.737 | **13.962** | 7.469 | 3.884E-02 |
| [232673_at](https://www.affymetrix.com/LinkServlet?probeset=232673_at) | [LRRFIP2](http://www.ncbi.nlm.nih.gov/entrez/query.fcgi?cmd=search&db=gene&term=LRRFIP2) | leucine rich repeat (in FLII) interacting protein 2 | **1.510** | **7.574** | 2.796 | **11.435** | 7.216 | 4.829E-02 |
| [216129_at](https://www.affymetrix.com/LinkServlet?probeset=216129_at) | [ATP9A](http://www.ncbi.nlm.nih.gov/entrez/query.fcgi?cmd=search&db=gene&term=ATP9A) | ATPase, class II, type 9A | **1.511** | **7.639** | 2.421 | **11.544** | 5.675 | 2.883E-02 |
| [211840_s_at](https://www.affymetrix.com/LinkServlet?probeset=211840_s_at) | [PDE4D](http://www.ncbi.nlm.nih.gov/entrez/query.fcgi?cmd=search&db=gene&term=PDE4D) | phosphodiesterase 4D, cAMP-specific | **1.512** | **6.210** | 0.758 | **9.388** | 6.477 | 3.133E-02 |
| [239251_at](https://www.affymetrix.com/LinkServlet?probeset=239251_at) | [NA](http://www.ncbi.nlm.nih.gov/entrez/query.fcgi?cmd=search&db=gene&term=NA) | NA | **1.513** | **19.363** | 10.331 | **29.289** | 14.078 | 4.887E-02 |
| [238766_at](https://www.affymetrix.com/LinkServlet?probeset=238766_at) | [NA](http://www.ncbi.nlm.nih.gov/entrez/query.fcgi?cmd=search&db=gene&term=NA) | NA | **1.513** | **7.461** | 2.459 | **11.287** | 6.532 | 3.795E-02 |
| [232096_x_at](https://www.affymetrix.com/LinkServlet?probeset=232096_x_at) | [NA](http://www.ncbi.nlm.nih.gov/entrez/query.fcgi?cmd=search&db=gene&term=NA) | NA | **1.514** | **37.243** | 20.303 | **56.390** | 21.455 | 4.333E-02 |
| [1568658_at](https://www.affymetrix.com/LinkServlet?probeset=1568658_at) | [C2orf74](http://www.ncbi.nlm.nih.gov/entrez/query.fcgi?cmd=search&db=gene&term=C2orf74) | chromosome 2 open reading frame 74 | **1.515** | **291.420** | 109.410 | **441.378** | 141.757 | 9.773E-03 |
| [236114_at](https://www.affymetrix.com/LinkServlet?probeset=236114_at) | [NA](http://www.ncbi.nlm.nih.gov/entrez/query.fcgi?cmd=search&db=gene&term=NA) | NA | **1.516** | **26.640** | 14.627 | **40.377** | 17.981 | 3.202E-02 |
| [217202_s_at](https://www.affymetrix.com/LinkServlet?probeset=217202_s_at) | [GLUL](http://www.ncbi.nlm.nih.gov/entrez/query.fcgi?cmd=search&db=gene&term=GLUL) | glutamate-ammonia ligase | **1.517** | **41.400** | 14.928 | **62.803** | 29.338 | 2.809E-02 |
| [236043_at](https://www.affymetrix.com/LinkServlet?probeset=236043_at) | [NA](http://www.ncbi.nlm.nih.gov/entrez/query.fcgi?cmd=search&db=gene&term=NA) | NA | **1.519** | **21.071** | 11.927 | **32.005** | 16.130 | 3.900E-02 |
| [233545_at](https://www.affymetrix.com/LinkServlet?probeset=233545_at) | [INPP5D](http://www.ncbi.nlm.nih.gov/entrez/query.fcgi?cmd=search&db=gene&term=INPP5D) | inositol polyphosphate-5-phosphatase, 145kDa | **1.520** | **10.022** | 4.314 | **15.234** | 7.333 | 3.500E-02 |
| [228123_s_at](https://www.affymetrix.com/LinkServlet?probeset=228123_s_at) | [ABHD12](http://www.ncbi.nlm.nih.gov/entrez/query.fcgi?cmd=search&db=gene&term=ABHD12) | abhydrolase domain containing 12 | **1.520** | **52.999** | 19.620 | **80.582** | 31.413 | 2.626E-02 |
| [224064_s_at](https://www.affymetrix.com/LinkServlet?probeset=224064_s_at) | [DHDDS](http://www.ncbi.nlm.nih.gov/entrez/query.fcgi?cmd=search&db=gene&term=DHDDS) | dehydrodolichyl diphosphate synthase | **1.529** | **27.255** | 9.669 | **41.667** | 17.586 | 3.324E-02 |
| [211217_s_at](https://www.affymetrix.com/LinkServlet?probeset=211217_s_at) | [KCNQ1](http://www.ncbi.nlm.nih.gov/entrez/query.fcgi?cmd=search&db=gene&term=KCNQ1) | potassium voltage-gated channel, KQT-like subfamily, member 1 | **1.530** | **9.297** | 4.707 | **14.220** | 6.707 | 4.059E-02 |
| [215002_at](https://www.affymetrix.com/LinkServlet?probeset=215002_at) | [NA](http://www.ncbi.nlm.nih.gov/entrez/query.fcgi?cmd=search&db=gene&term=NA) | NA | **1.530** | **9.380** | 5.164 | **14.351** | 6.973 | 4.180E-02 |
| [214984_at](https://www.affymetrix.com/LinkServlet?probeset=214984_at) | [NA](http://www.ncbi.nlm.nih.gov/entrez/query.fcgi?cmd=search&db=gene&term=NA) | NA | **1.530** | **6.661** | 1.318 | **10.194** | 5.656 | 2.577E-02 |
| [201417_at](https://www.affymetrix.com/LinkServlet?probeset=201417_at) | [SOX4](http://www.ncbi.nlm.nih.gov/entrez/query.fcgi?cmd=search&db=gene&term=SOX4) | SRY (sex determining region Y)-box 4 | **1.532** | **47.221** | 23.451 | **72.354** | 34.500 | 4.957E-02 |
| [206960_at](https://www.affymetrix.com/LinkServlet?probeset=206960_at) | [LPAR4](http://www.ncbi.nlm.nih.gov/entrez/query.fcgi?cmd=search&db=gene&term=LPAR4) | lysophosphatidic acid receptor 4 | **1.533** | **6.155** | 0.443 | **9.435** | 5.881 | 2.991E-02 |
| [216202_s_at](https://www.affymetrix.com/LinkServlet?probeset=216202_s_at) | [SPTLC2](http://www.ncbi.nlm.nih.gov/entrez/query.fcgi?cmd=search&db=gene&term=SPTLC2) | serine palmitoyltransferase, long chain base subunit 2 | **1.533** | **36.817** | 14.152 | **56.446** | 27.704 | 1.027E-02 |
| [1560615_a_at](https://www.affymetrix.com/LinkServlet?probeset=1560615_a_at) | [NA](http://www.ncbi.nlm.nih.gov/entrez/query.fcgi?cmd=search&db=gene&term=NA) | NA | **1.536** | **40.587** | 23.508 | **62.334** | 26.392 | 1.587E-02 |
| [210491_at](https://www.affymetrix.com/LinkServlet?probeset=210491_at) | [NA](http://www.ncbi.nlm.nih.gov/entrez/query.fcgi?cmd=search&db=gene&term=NA) | NA | **1.537** | **6.000** | 0.000 | **9.223** | 5.710 | 1.965E-02 |
| [239296_at](https://www.affymetrix.com/LinkServlet?probeset=239296_at) | [NA](http://www.ncbi.nlm.nih.gov/entrez/query.fcgi?cmd=search&db=gene&term=NA) | NA | **1.538** | **7.601** | 2.935 | **11.690** | 7.354 | 4.125E-02 |
| [229373_at](https://www.affymetrix.com/LinkServlet?probeset=229373_at) | [NA](http://www.ncbi.nlm.nih.gov/entrez/query.fcgi?cmd=search&db=gene&term=NA) | NA | **1.540** | **92.180** | 26.190 | **141.997** | 58.724 | 3.712E-03 |
| [242733_at](https://www.affymetrix.com/LinkServlet?probeset=242733_at) | [NA](http://www.ncbi.nlm.nih.gov/entrez/query.fcgi?cmd=search&db=gene&term=NA) | NA | **1.541** | **6.225** | 0.900 | **9.590** | 6.856 | 4.339E-02 |
| [218837_s_at](https://www.affymetrix.com/LinkServlet?probeset=218837_s_at) | [UBE2D4](http://www.ncbi.nlm.nih.gov/entrez/query.fcgi?cmd=search&db=gene&term=UBE2D4) | ubiquitin-conjugating enzyme E2D 4 (putative) | **1.541** | **44.757** | 18.813 | **68.973** | 36.551 | 3.909E-02 |
| [201466_s_at](https://www.affymetrix.com/LinkServlet?probeset=201466_s_at) | [JUN](http://www.ncbi.nlm.nih.gov/entrez/query.fcgi?cmd=search&db=gene&term=JUN) | jun proto-oncogene | **1.542** | **192.438** | 119.213 | **296.659** | 144.598 | 2.654E-02 |
| [243804_at](https://www.affymetrix.com/LinkServlet?probeset=243804_at) | [MTMR7](http://www.ncbi.nlm.nih.gov/entrez/query.fcgi?cmd=search&db=gene&term=MTMR7) | myotubularin related protein 7 | **1.543** | **9.718** | 4.775 | **14.991** | 5.296 | 7.619E-03 |
| [217419_x_at](https://www.affymetrix.com/LinkServlet?probeset=217419_x_at) | [AGRN](http://www.ncbi.nlm.nih.gov/entrez/query.fcgi?cmd=search&db=gene&term=AGRN) | agrin | **1.544** | **38.099** | 25.899 | **58.806** | 26.884 | 3.560E-02 |
| [231618_s_at](https://www.affymetrix.com/LinkServlet?probeset=231618_s_at) | [SUN3](http://www.ncbi.nlm.nih.gov/entrez/query.fcgi?cmd=search&db=gene&term=SUN3) | Sad1 and UNC84 domain containing 3 | **1.545** | **7.251** | 1.550 | **11.202** | 6.142 | 3.133E-02 |
| [230206_at](https://www.affymetrix.com/LinkServlet?probeset=230206_at) | [DOCK5](http://www.ncbi.nlm.nih.gov/entrez/query.fcgi?cmd=search&db=gene&term=DOCK5) | dedicator of cytokinesis 5 | **1.545** | **225.300** | 153.564 | **348.088** | 191.175 | 4.703E-02 |
| [230074_s_at](https://www.affymetrix.com/LinkServlet?probeset=230074_s_at) | [NA](http://www.ncbi.nlm.nih.gov/entrez/query.fcgi?cmd=search&db=gene&term=NA) | NA | **1.547** | **6.078** | 0.311 | **9.401** | 5.625 | 1.576E-02 |
| [211424_x_at](https://www.affymetrix.com/LinkServlet?probeset=211424_x_at) | [METTL7A](http://www.ncbi.nlm.nih.gov/entrez/query.fcgi?cmd=search&db=gene&term=METTL7A) | methyltransferase like 7A | **1.547** | **15.895** | 5.902 | **24.595** | 10.886 | 2.420E-02 |
| [236495_at](https://www.affymetrix.com/LinkServlet?probeset=236495_at) | [NA](http://www.ncbi.nlm.nih.gov/entrez/query.fcgi?cmd=search&db=gene&term=NA) | NA | **1.548** | **9.954** | 6.618 | **15.408** | 8.097 | 2.324E-02 |
| [222387_s_at](https://www.affymetrix.com/LinkServlet?probeset=222387_s_at) | [VPS35](http://www.ncbi.nlm.nih.gov/entrez/query.fcgi?cmd=search&db=gene&term=VPS35) | vacuolar protein sorting 35 homolog (S. cerevisiae) | **1.548** | **25.011** | 12.034 | **38.726** | 13.050 | 1.547E-02 |
| [216176_at](https://www.affymetrix.com/LinkServlet?probeset=216176_at) | [HCRP1](http://www.ncbi.nlm.nih.gov/entrez/query.fcgi?cmd=search&db=gene&term=HCRP1) | hepatocellular carcinoma-related HCRP1 | **1.549** | **59.801** | 63.958 | **92.608** | 51.074 | 2.939E-02 |
| [1554929_at](https://www.affymetrix.com/LinkServlet?probeset=1554929_at) | [SIK3](http://www.ncbi.nlm.nih.gov/entrez/query.fcgi?cmd=search&db=gene&term=SIK3) | SIK family kinase 3 | **1.550** | **6.410** | 1.292 | **9.934** | 6.373 | 3.103E-02 |
| [1563573_at](https://www.affymetrix.com/LinkServlet?probeset=1563573_at) | [NA](http://www.ncbi.nlm.nih.gov/entrez/query.fcgi?cmd=search&db=gene&term=NA) | NA | **1.551** | **47.443** | 22.291 | **73.601** | 32.275 | 3.586E-02 |
| [207895_at](https://www.affymetrix.com/LinkServlet?probeset=207895_at) | [NAALADL1](http://www.ncbi.nlm.nih.gov/entrez/query.fcgi?cmd=search&db=gene&term=NAALADL1) | N-acetylated alpha-linked acidic dipeptidase-like 1 | **1.552** | **30.277** | 13.831 | **46.984** | 16.495 | 2.375E-02 |
| [213902_at](https://www.affymetrix.com/LinkServlet?probeset=213902_at) | [ASAH1](http://www.ncbi.nlm.nih.gov/entrez/query.fcgi?cmd=search&db=gene&term=ASAH1) | N-acylsphingosine amidohydrolase (acid ceramidase) 1 | **1.552** | **293.824** | 141.994 | **456.154** | 222.201 | 3.526E-02 |
| [215618_at](https://www.affymetrix.com/LinkServlet?probeset=215618_at) | [RSU1](http://www.ncbi.nlm.nih.gov/entrez/query.fcgi?cmd=search&db=gene&term=RSU1) | Ras suppressor protein 1 | **1.553** | **15.910** | 6.943 | **24.707** | 12.166 | 4.958E-02 |
| [243808_at](https://www.affymetrix.com/LinkServlet?probeset=243808_at) | [NA](http://www.ncbi.nlm.nih.gov/entrez/query.fcgi?cmd=search&db=gene&term=NA) | NA | **1.555** | **26.750** | 17.255 | **41.589** | 19.127 | 2.389E-02 |
| [226485_at](https://www.affymetrix.com/LinkServlet?probeset=226485_at) | [VSIG10](http://www.ncbi.nlm.nih.gov/entrez/query.fcgi?cmd=search&db=gene&term=VSIG10) | V-set and immunoglobulin domain containing 10 | **1.555** | **20.032** | 14.765 | **31.146** | 10.998 | 2.008E-02 |
| [201465_s_at](https://www.affymetrix.com/LinkServlet?probeset=201465_s_at) | [JUN](http://www.ncbi.nlm.nih.gov/entrez/query.fcgi?cmd=search&db=gene&term=JUN) | jun proto-oncogene | **1.557** | **127.669** | 34.451 | **198.728** | 133.855 | 1.773E-02 |
| [201718_s_at](https://www.affymetrix.com/LinkServlet?probeset=201718_s_at) | [EPB41L2](http://www.ncbi.nlm.nih.gov/entrez/query.fcgi?cmd=search&db=gene&term=EPB41L2) | erythrocyte membrane protein band 4.1-like 2 | **1.558** | **50.572** | 45.151 | **78.779** | 33.114 | 2.410E-02 |
| [229112_at](https://www.affymetrix.com/LinkServlet?probeset=229112_at) | [SIRT5](http://www.ncbi.nlm.nih.gov/entrez/query.fcgi?cmd=search&db=gene&term=SIRT5) | sirtuin 5 | **1.558** | **108.452** | 30.137 | **169.011** | 97.190 | 3.202E-02 |
| [226401_at](https://www.affymetrix.com/LinkServlet?probeset=226401_at) | [PARP10](http://www.ncbi.nlm.nih.gov/entrez/query.fcgi?cmd=search&db=gene&term=PARP10) | poly (ADP-ribose) polymerase family, member 10 | **1.559** | **6.756** | 1.232 | **10.531** | 3.826 | 8.799E-04 |
| [243480_at](https://www.affymetrix.com/LinkServlet?probeset=243480_at) | [NA](http://www.ncbi.nlm.nih.gov/entrez/query.fcgi?cmd=search&db=gene&term=NA) | NA | **1.559** | **25.996** | 22.185 | **40.537** | 19.546 | 4.295E-02 |
| [1553108_at](https://www.affymetrix.com/LinkServlet?probeset=1553108_at) | [C5orf24](http://www.ncbi.nlm.nih.gov/entrez/query.fcgi?cmd=search&db=gene&term=C5orf24) | chromosome 5 open reading frame 24 | **1.560** | **8.883** | 3.039 | **13.860** | 8.368 | 4.427E-02 |
| [232363_at](https://www.affymetrix.com/LinkServlet?probeset=232363_at) | [NA](http://www.ncbi.nlm.nih.gov/entrez/query.fcgi?cmd=search&db=gene&term=NA) | NA | **1.561** | **25.795** | 11.943 | **40.255** | 17.070 | 4.204E-02 |
| [238360_s_at](https://www.affymetrix.com/LinkServlet?probeset=238360_s_at) | [LOC100505576](http://www.ncbi.nlm.nih.gov/entrez/query.fcgi?cmd=search&db=gene&term=LOC100505576) | hypothetical LOC100505576 | **1.563** | **17.371** | 10.655 | **27.149** | 9.485 | 1.290E-02 |
| [1556176_at](https://www.affymetrix.com/LinkServlet?probeset=1556176_at) | [TAF8](http://www.ncbi.nlm.nih.gov/entrez/query.fcgi?cmd=search&db=gene&term=TAF8) | TAF8 RNA polymerase II, TATA box binding protein (TBP)-associated factor, 43kDa | **1.563** | **85.688** | 39.775 | **133.925** | 72.492 | 4.947E-02 |
| [200648_s_at](https://www.affymetrix.com/LinkServlet?probeset=200648_s_at) | [GLUL](http://www.ncbi.nlm.nih.gov/entrez/query.fcgi?cmd=search&db=gene&term=GLUL) | glutamate-ammonia ligase | **1.564** | **98.828** | 42.767 | **154.532** | 84.334 | 4.869E-02 |
| [207891_s_at](https://www.affymetrix.com/LinkServlet?probeset=207891_s_at) | [NA](http://www.ncbi.nlm.nih.gov/entrez/query.fcgi?cmd=search&db=gene&term=NA) | NA | **1.566** | **17.111** | 6.548 | **26.793** | 10.595 | 6.519E-03 |
| [228044_at](https://www.affymetrix.com/LinkServlet?probeset=228044_at) | [SERP2](http://www.ncbi.nlm.nih.gov/entrez/query.fcgi?cmd=search&db=gene&term=SERP2) | stress-associated endoplasmic reticulum protein family member 2 | **1.566** | **7.708** | 2.698 | **12.073** | 7.377 | 4.938E-02 |
| [238759_at](https://www.affymetrix.com/LinkServlet?probeset=238759_at) | [CCDC88A](http://www.ncbi.nlm.nih.gov/entrez/query.fcgi?cmd=search&db=gene&term=CCDC88A) | coiled-coil domain containing 88A | **1.567** | **6.818** | 2.246 | **10.684** | 7.141 | 3.696E-02 |
| [203946_s_at](https://www.affymetrix.com/LinkServlet?probeset=203946_s_at) | [ARG2](http://www.ncbi.nlm.nih.gov/entrez/query.fcgi?cmd=search&db=gene&term=ARG2) | arginase, type II | **1.567** | **72.515** | 19.714 | **113.654** | 68.422 | 3.769E-02 |
| [206636_at](https://www.affymetrix.com/LinkServlet?probeset=206636_at) | [RASA2](http://www.ncbi.nlm.nih.gov/entrez/query.fcgi?cmd=search&db=gene&term=RASA2) | RAS p21 protein activator 2 | **1.570** | **22.787** | 10.805 | **35.783** | 16.691 | 2.563E-02 |
| [210910_s_at](https://www.affymetrix.com/LinkServlet?probeset=210910_s_at) | [POMZP3](http://www.ncbi.nlm.nih.gov/entrez/query.fcgi?cmd=search&db=gene&term=POMZP3) | POM121 and ZP3 fusion | **1.574** | **80.308** | 52.041 | **126.374** | 52.204 | 2.255E-02 |
| [212225_at](https://www.affymetrix.com/LinkServlet?probeset=212225_at) | [EIF1](http://www.ncbi.nlm.nih.gov/entrez/query.fcgi?cmd=search&db=gene&term=EIF1) | eukaryotic translation initiation factor 1 | **1.574** | **159.549** | 73.484 | **251.208** | 105.918 | 2.171E-02 |
| [218704_at](https://www.affymetrix.com/LinkServlet?probeset=218704_at) | [RNF43](http://www.ncbi.nlm.nih.gov/entrez/query.fcgi?cmd=search&db=gene&term=RNF43) | ring finger protein 43 | **1.575** | **7.042** | 2.408 | **11.090** | 5.898 | 1.789E-02 |
| [230380_at](https://www.affymetrix.com/LinkServlet?probeset=230380_at) | [THAP2](http://www.ncbi.nlm.nih.gov/entrez/query.fcgi?cmd=search&db=gene&term=THAP2) | THAP domain containing, apoptosis associated protein 2 | **1.575** | **33.580** | 24.451 | **52.886** | 21.329 | 3.053E-02 |
| [227044_at](https://www.affymetrix.com/LinkServlet?probeset=227044_at) | [NA](http://www.ncbi.nlm.nih.gov/entrez/query.fcgi?cmd=search&db=gene&term=NA) | NA | **1.575** | **71.245** | 23.470 | **112.214** | 43.460 | 1.395E-02 |
| [213334_x_at](https://www.affymetrix.com/LinkServlet?probeset=213334_x_at) | [HAUS7](http://www.ncbi.nlm.nih.gov/entrez/query.fcgi?cmd=search&db=gene&term=HAUS7) | HAUS augmin-like complex, subunit 7 | **1.575** | **99.681** | 26.098 | **157.014** | 56.995 | 1.236E-03 |
| [243832_at](https://www.affymetrix.com/LinkServlet?probeset=243832_at) | [WDR33](http://www.ncbi.nlm.nih.gov/entrez/query.fcgi?cmd=search&db=gene&term=WDR33) | WD repeat domain 33 | **1.576** | **6.375** | 1.498 | **10.046** | 6.168 | 3.057E-02 |
| [215528_at](https://www.affymetrix.com/LinkServlet?probeset=215528_at) | [NA](http://www.ncbi.nlm.nih.gov/entrez/query.fcgi?cmd=search&db=gene&term=NA) | NA | **1.576** | **16.155** | 14.331 | **25.461** | 12.248 | 2.995E-02 |
| [222451_s_at](https://www.affymetrix.com/LinkServlet?probeset=222451_s_at) | [ZDHHC9](http://www.ncbi.nlm.nih.gov/entrez/query.fcgi?cmd=search&db=gene&term=ZDHHC9) | zinc finger, DHHC-type containing 9 | **1.578** | **20.818** | 9.099 | **32.840** | 11.874 | 1.968E-02 |
| [241600_at](https://www.affymetrix.com/LinkServlet?probeset=241600_at) | [NA](http://www.ncbi.nlm.nih.gov/entrez/query.fcgi?cmd=search&db=gene&term=NA) | NA | **1.578** | **11.646** | 7.488 | **18.376** | 8.399 | 1.991E-02 |
| [1554237_at](https://www.affymetrix.com/LinkServlet?probeset=1554237_at) | [SDCCAG8](http://www.ncbi.nlm.nih.gov/entrez/query.fcgi?cmd=search&db=gene&term=SDCCAG8) | serologically defined colon cancer antigen 8 | **1.579** | **86.513** | 30.732 | **136.597** | 49.577 | 6.825E-03 |
| [204257_at](https://www.affymetrix.com/LinkServlet?probeset=204257_at) | [FADS3](http://www.ncbi.nlm.nih.gov/entrez/query.fcgi?cmd=search&db=gene&term=FADS3) | fatty acid desaturase 3 | **1.583** | **44.760** | 17.107 | **70.835** | 44.479 | 4.308E-02 |
| [232614_at](https://www.affymetrix.com/LinkServlet?probeset=232614_at) | [NA](http://www.ncbi.nlm.nih.gov/entrez/query.fcgi?cmd=search&db=gene&term=NA) | NA | **1.584** | **199.132** | 156.398 | **315.350** | 149.461 | 1.512E-02 |
| [238957_at](https://www.affymetrix.com/LinkServlet?probeset=238957_at) | [NA](http://www.ncbi.nlm.nih.gov/entrez/query.fcgi?cmd=search&db=gene&term=NA) | NA | **1.585** | **7.120** | 2.300 | **11.286** | 4.978 | 8.038E-03 |
| [227777_at](https://www.affymetrix.com/LinkServlet?probeset=227777_at) | [C10orf18](http://www.ncbi.nlm.nih.gov/entrez/query.fcgi?cmd=search&db=gene&term=C10orf18) | chromosome 10 open reading frame 18 | **1.587** | **79.763** | 32.155 | **126.579** | 28.059 | 3.098E-03 |
| [220232_at](https://www.affymetrix.com/LinkServlet?probeset=220232_at) | [SCD5](http://www.ncbi.nlm.nih.gov/entrez/query.fcgi?cmd=search&db=gene&term=SCD5) | stearoyl-CoA desaturase 5 | **1.589** | **9.967** | 4.266 | **15.833** | 8.823 | 1.645E-02 |
| [204800_s_at](https://www.affymetrix.com/LinkServlet?probeset=204800_s_at) | [DHRS12](http://www.ncbi.nlm.nih.gov/entrez/query.fcgi?cmd=search&db=gene&term=DHRS12) | dehydrogenase/reductase (SDR family) member 12 | **1.589** | **112.321** | 52.274 | **178.507** | 91.758 | 4.817E-02 |
| [211751_at](https://www.affymetrix.com/LinkServlet?probeset=211751_at) | [PDE4DIP](http://www.ncbi.nlm.nih.gov/entrez/query.fcgi?cmd=search&db=gene&term=PDE4DIP) | phosphodiesterase 4D interacting protein | **1.591** | **30.617** | 21.528 | **48.701** | 27.800 | 3.646E-02 |
| [232257_s_at](https://www.affymetrix.com/LinkServlet?probeset=232257_s_at) | [NA](http://www.ncbi.nlm.nih.gov/entrez/query.fcgi?cmd=search&db=gene&term=NA) | NA | **1.591** | **48.033** | 28.769 | **76.437** | 41.333 | 4.385E-02 |
| [219427_at](https://www.affymetrix.com/LinkServlet?probeset=219427_at) | [FAT4](http://www.ncbi.nlm.nih.gov/entrez/query.fcgi?cmd=search&db=gene&term=FAT4) | FAT tumor suppressor homolog 4 (Drosophila) | **1.591** | **6.013** | 0.052 | **9.569** | 7.666 | 4.547E-02 |
| [236875_at](https://www.affymetrix.com/LinkServlet?probeset=236875_at) | [LOC100130705](http://www.ncbi.nlm.nih.gov/entrez/query.fcgi?cmd=search&db=gene&term=LOC100130705) | hypothetical protein LOC100130705 | **1.593** | **6.000** | 0.000 | **9.557** | 3.848 | 3.052E-04 |
| [237119_at](https://www.affymetrix.com/LinkServlet?probeset=237119_at) | [NA](http://www.ncbi.nlm.nih.gov/entrez/query.fcgi?cmd=search&db=gene&term=NA) | NA | **1.594** | **7.911** | 3.251 | **12.612** | 6.901 | 3.496E-02 |
| [223641_at](https://www.affymetrix.com/LinkServlet?probeset=223641_at) | [NA](http://www.ncbi.nlm.nih.gov/entrez/query.fcgi?cmd=search&db=gene&term=NA) | NA | **1.594** | **34.375** | 14.327 | **54.807** | 24.332 | 2.931E-02 |
| [1558034_s_at](https://www.affymetrix.com/LinkServlet?probeset=1558034_s_at) | [CP](http://www.ncbi.nlm.nih.gov/entrez/query.fcgi?cmd=search&db=gene&term=CP) | ceruloplasmin (ferroxidase) | **1.595** | **6.000** | 0.000 | **9.571** | 5.232 | 8.914E-03 |
| [223696_at](https://www.affymetrix.com/LinkServlet?probeset=223696_at) | [ARSD](http://www.ncbi.nlm.nih.gov/entrez/query.fcgi?cmd=search&db=gene&term=ARSD) | arylsulfatase D | **1.596** | **32.023** | 14.559 | **51.119** | 18.699 | 1.382E-02 |
| [242341_x_at](https://www.affymetrix.com/LinkServlet?probeset=242341_x_at) | [GLYCTK](http://www.ncbi.nlm.nih.gov/entrez/query.fcgi?cmd=search&db=gene&term=GLYCTK) | glycerate kinase | **1.598** | **16.545** | 6.276 | **26.442** | 11.736 | 1.745E-02 |
| [227043_at](https://www.affymetrix.com/LinkServlet?probeset=227043_at) | [CCDC159](http://www.ncbi.nlm.nih.gov/entrez/query.fcgi?cmd=search&db=gene&term=CCDC159) | coiled-coil domain containing 159 | **1.599** | **36.305** | 14.165 | **58.034** | 33.817 | 1.873E-02 |
| [1559204_x_at](https://www.affymetrix.com/LinkServlet?probeset=1559204_x_at) | [KRAS](http://www.ncbi.nlm.nih.gov/entrez/query.fcgi?cmd=search&db=gene&term=KRAS) | v-Ki-ras2 Kirsten rat sarcoma viral oncogene homolog | **1.603** | **6.970** | 1.994 | **11.175** | 6.396 | 2.192E-02 |
| [215468_at](https://www.affymetrix.com/LinkServlet?probeset=215468_at) | [LOC647070](http://www.ncbi.nlm.nih.gov/entrez/query.fcgi?cmd=search&db=gene&term=LOC647070) | hypothetical LOC647070 | **1.612** | **10.411** | 6.136 | **16.778** | 9.976 | 4.124E-02 |
| [220596_at](https://www.affymetrix.com/LinkServlet?probeset=220596_at) | [GPATCH4](http://www.ncbi.nlm.nih.gov/entrez/query.fcgi?cmd=search&db=gene&term=GPATCH4) | G patch domain containing 4 | **1.614** | **6.730** | 1.454 | **10.862** | 5.347 | 7.230E-03 |
| [223564_s_at](https://www.affymetrix.com/LinkServlet?probeset=223564_s_at) | [GNB1L](http://www.ncbi.nlm.nih.gov/entrez/query.fcgi?cmd=search&db=gene&term=GNB1L) | guanine nucleotide binding protein (G protein), beta polypeptide 1-like | **1.615** | **12.430** | 4.904 | **20.075** | 11.612 | 4.845E-02 |
| [223628_at](https://www.affymetrix.com/LinkServlet?probeset=223628_at) | [TMEM191A](http://www.ncbi.nlm.nih.gov/entrez/query.fcgi?cmd=search&db=gene&term=TMEM191A) | transmembrane protein 191A | **1.615** | **16.942** | 5.801 | **27.366** | 14.378 | 4.941E-02 |
| [231833_at](https://www.affymetrix.com/LinkServlet?probeset=231833_at) | [RBM33](http://www.ncbi.nlm.nih.gov/entrez/query.fcgi?cmd=search&db=gene&term=RBM33) | RNA binding motif protein 33 | **1.615** | **6.778** | 2.232 | **10.949** | 5.661 | 9.788E-03 |
| [219093_at](https://www.affymetrix.com/LinkServlet?probeset=219093_at) | [PID1](http://www.ncbi.nlm.nih.gov/entrez/query.fcgi?cmd=search&db=gene&term=PID1) | phosphotyrosine interaction domain containing 1 | **1.617** | **281.034** | 151.351 | **454.294** | 230.172 | 4.740E-02 |
| [227832_at](https://www.affymetrix.com/LinkServlet?probeset=227832_at) | [MBD6](http://www.ncbi.nlm.nih.gov/entrez/query.fcgi?cmd=search&db=gene&term=MBD6) | methyl-CpG binding domain protein 6 | **1.618** | **11.215** | 5.094 | **18.149** | 10.539 | 4.830E-02 |
| [242361_at](https://www.affymetrix.com/LinkServlet?probeset=242361_at) | [IMMT](http://www.ncbi.nlm.nih.gov/entrez/query.fcgi?cmd=search&db=gene&term=IMMT) | inner membrane protein, mitochondrial | **1.619** | **7.532** | 3.640 | **12.194** | 8.330 | 4.919E-02 |
| [219717_at](https://www.affymetrix.com/LinkServlet?probeset=219717_at) | [DCAF16](http://www.ncbi.nlm.nih.gov/entrez/query.fcgi?cmd=search&db=gene&term=DCAF16) | DDB1 and CUL4 associated factor 16 | **1.619** | **74.060** | 47.991 | **119.923** | 48.205 | 2.457E-02 |
| [226578_s_at](https://www.affymetrix.com/LinkServlet?probeset=226578_s_at) | [DUSP1](http://www.ncbi.nlm.nih.gov/entrez/query.fcgi?cmd=search&db=gene&term=DUSP1) | dual specificity phosphatase 1 | **1.620** | **17.248** | 7.979 | **27.941** | 11.391 | 2.309E-02 |
| [232245_at](https://www.affymetrix.com/LinkServlet?probeset=232245_at) | [SLC25A34](http://www.ncbi.nlm.nih.gov/entrez/query.fcgi?cmd=search&db=gene&term=SLC25A34) | solute carrier family 25, member 34 | **1.621** | **35.797** | 18.764 | **58.035** | 26.232 | 1.992E-02 |
| [237156_at](https://www.affymetrix.com/LinkServlet?probeset=237156_at) | [NA](http://www.ncbi.nlm.nih.gov/entrez/query.fcgi?cmd=search&db=gene&term=NA) | NA | **1.623** | **6.583** | 1.344 | **10.685** | 6.395 | 1.235E-02 |
| [1556479_at](https://www.affymetrix.com/LinkServlet?probeset=1556479_at) | [NA](http://www.ncbi.nlm.nih.gov/entrez/query.fcgi?cmd=search&db=gene&term=NA) | NA | **1.623** | **6.167** | 0.627 | **10.011** | 8.659 | 4.210E-02 |
| [207872_s_at](https://www.affymetrix.com/LinkServlet?probeset=207872_s_at) | [LILRA1](http://www.ncbi.nlm.nih.gov/entrez/query.fcgi?cmd=search&db=gene&term=LILRA1) | leukocyte immunoglobulin-like receptor, subfamily A (with TM domain), member 1 | **1.624** | **15.645** | 12.818 | **25.401** | 13.575 | 4.288E-02 |
| [236101_at](https://www.affymetrix.com/LinkServlet?probeset=236101_at) | [NA](http://www.ncbi.nlm.nih.gov/entrez/query.fcgi?cmd=search&db=gene&term=NA) | NA | **1.624** | **29.787** | 30.445 | **48.369** | 29.179 | 4.832E-02 |
| [236778_at](https://www.affymetrix.com/LinkServlet?probeset=236778_at) | [NA](http://www.ncbi.nlm.nih.gov/entrez/query.fcgi?cmd=search&db=gene&term=NA) | NA | **1.624** | **6.538** | 1.505 | **10.619** | 7.598 | 2.817E-02 |
| [238065_at](https://www.affymetrix.com/LinkServlet?probeset=238065_at) | [TPM3](http://www.ncbi.nlm.nih.gov/entrez/query.fcgi?cmd=search&db=gene&term=TPM3) | tropomyosin 3 | **1.625** | **72.494** | 23.864 | **117.795** | 72.512 | 2.084E-02 |
| [243768_at](https://www.affymetrix.com/LinkServlet?probeset=243768_at) | [NA](http://www.ncbi.nlm.nih.gov/entrez/query.fcgi?cmd=search&db=gene&term=NA) | NA | **1.625** | **124.589** | 90.075 | **202.462** | 121.006 | 4.313E-02 |
| [237778_at](https://www.affymetrix.com/LinkServlet?probeset=237778_at) | [NA](http://www.ncbi.nlm.nih.gov/entrez/query.fcgi?cmd=search&db=gene&term=NA) | NA | **1.629** | **20.263** | 13.381 | **33.009** | 12.794 | 2.597E-02 |
| [216685_s_at](https://www.affymetrix.com/LinkServlet?probeset=216685_s_at) | [MTAP](http://www.ncbi.nlm.nih.gov/entrez/query.fcgi?cmd=search&db=gene&term=MTAP) | methylthioadenosine phosphorylase | **1.631** | **17.560** | 6.307 | **28.635** | 9.505 | 1.619E-03 |
| [234929_s_at](https://www.affymetrix.com/LinkServlet?probeset=234929_s_at) | [SPATA7](http://www.ncbi.nlm.nih.gov/entrez/query.fcgi?cmd=search&db=gene&term=SPATA7) | spermatogenesis associated 7 | **1.631** | **34.902** | 18.677 | **56.922** | 36.730 | 3.669E-02 |
| [211094_s_at](https://www.affymetrix.com/LinkServlet?probeset=211094_s_at) | [NF1](http://www.ncbi.nlm.nih.gov/entrez/query.fcgi?cmd=search&db=gene&term=NF1) | neurofibromin 1 | **1.632** | **8.601** | 4.141 | **14.035** | 9.731 | 4.235E-02 |
| [204915_s_at](https://www.affymetrix.com/LinkServlet?probeset=204915_s_at) | [SOX11](http://www.ncbi.nlm.nih.gov/entrez/query.fcgi?cmd=search&db=gene&term=SOX11) | SRY (sex determining region Y)-box 11 | **1.633** | **6.171** | 0.682 | **10.075** | 7.482 | 3.228E-02 |
| [206301_at](https://www.affymetrix.com/LinkServlet?probeset=206301_at) | [TEC](http://www.ncbi.nlm.nih.gov/entrez/query.fcgi?cmd=search&db=gene&term=TEC) | tec protein tyrosine kinase | **1.633** | **7.080** | 2.093 | **11.562** | 7.288 | 4.179E-02 |
| [215861_at](https://www.affymetrix.com/LinkServlet?probeset=215861_at) | [NA](http://www.ncbi.nlm.nih.gov/entrez/query.fcgi?cmd=search&db=gene&term=NA) | NA | **1.638** | **15.674** | 12.028 | **25.680** | 16.045 | 4.290E-02 |
| [218062_x_at](https://www.affymetrix.com/LinkServlet?probeset=218062_x_at) | [CDC42EP4](http://www.ncbi.nlm.nih.gov/entrez/query.fcgi?cmd=search&db=gene&term=CDC42EP4) | CDC42 effector protein (Rho GTPase binding) 4 | **1.642** | **51.919** | 33.923 | **85.275** | 41.997 | 2.138E-02 |
| [201835_s_at](https://www.affymetrix.com/LinkServlet?probeset=201835_s_at) | [PRKAB1](http://www.ncbi.nlm.nih.gov/entrez/query.fcgi?cmd=search&db=gene&term=PRKAB1) | protein kinase, AMP-activated, beta 1 non-catalytic subunit | **1.643** | **11.592** | 4.270 | **19.051** | 9.877 | 2.474E-02 |
| [221270_s_at](https://www.affymetrix.com/LinkServlet?probeset=221270_s_at) | [QTRT1](http://www.ncbi.nlm.nih.gov/entrez/query.fcgi?cmd=search&db=gene&term=QTRT1) | queuine tRNA-ribosyltransferase 1 | **1.646** | **21.767** | 8.584 | **35.818** | 14.712 | 2.753E-03 |
| [215378_at](https://www.affymetrix.com/LinkServlet?probeset=215378_at) | [NA](http://www.ncbi.nlm.nih.gov/entrez/query.fcgi?cmd=search&db=gene&term=NA) | NA | **1.646** | **71.805** | 45.195 | **118.212** | 57.131 | 2.655E-02 |
| [218850_s_at](https://www.affymetrix.com/LinkServlet?probeset=218850_s_at) | [LIMD1](http://www.ncbi.nlm.nih.gov/entrez/query.fcgi?cmd=search&db=gene&term=LIMD1) | LIM domains containing 1 | **1.647** | **17.556** | 9.144 | **28.916** | 17.318 | 3.077E-02 |
| [226573_at](https://www.affymetrix.com/LinkServlet?probeset=226573_at) | [DIRAS1](http://www.ncbi.nlm.nih.gov/entrez/query.fcgi?cmd=search&db=gene&term=DIRAS1) | DIRAS family, GTP-binding RAS-like 1 | **1.648** | **6.964** | 1.753 | **11.478** | 8.564 | 4.910E-02 |
| [202598_at](https://www.affymetrix.com/LinkServlet?probeset=202598_at) | [S100A13](http://www.ncbi.nlm.nih.gov/entrez/query.fcgi?cmd=search&db=gene&term=S100A13) | S100 calcium binding protein A13 | **1.649** | **70.033** | 43.981 | **115.455** | 71.207 | 4.554E-02 |
| [217208_s_at](https://www.affymetrix.com/LinkServlet?probeset=217208_s_at) | [DLG1](http://www.ncbi.nlm.nih.gov/entrez/query.fcgi?cmd=search&db=gene&term=DLG1) | discs, large homolog 1 (Drosophila) | **1.649** | **7.577** | 1.916 | **12.493** | 7.269 | 1.390E-02 |
| [1554143_a_at](https://www.affymetrix.com/LinkServlet?probeset=1554143_a_at) | [SUGT1P3](http://www.ncbi.nlm.nih.gov/entrez/query.fcgi?cmd=search&db=gene&term=SUGT1P3) | suppressor of G2 allele of SKP1 (S. cerevisiae) pseudogene 3 | **1.650** | **8.565** | 3.582 | **14.128** | 6.621 | 3.580E-03 |
| [239555_at](https://www.affymetrix.com/LinkServlet?probeset=239555_at) | [NA](http://www.ncbi.nlm.nih.gov/entrez/query.fcgi?cmd=search&db=gene&term=NA) | NA | **1.650** | **25.152** | 14.398 | **41.505** | 24.371 | 3.544E-02 |
| [235632_at](https://www.affymetrix.com/LinkServlet?probeset=235632_at) | [NA](http://www.ncbi.nlm.nih.gov/entrez/query.fcgi?cmd=search&db=gene&term=NA) | NA | **1.654** | **14.262** | 8.976 | **23.594** | 11.733 | 1.348E-02 |
| [1564547_x_at](https://www.affymetrix.com/LinkServlet?probeset=1564547_x_at) | [NA](http://www.ncbi.nlm.nih.gov/entrez/query.fcgi?cmd=search&db=gene&term=NA) | NA | **1.658** | **14.732** | 7.335 | **24.422** | 14.083 | 2.925E-02 |
| [243253_at](https://www.affymetrix.com/LinkServlet?probeset=243253_at) | [NA](http://www.ncbi.nlm.nih.gov/entrez/query.fcgi?cmd=search&db=gene&term=NA) | NA | **1.660** | **6.714** | 1.773 | **11.148** | 7.953 | 3.324E-02 |
| [241159_x_at](https://www.affymetrix.com/LinkServlet?probeset=241159_x_at) | [NA](http://www.ncbi.nlm.nih.gov/entrez/query.fcgi?cmd=search&db=gene&term=NA) | NA | **1.662** | **16.586** | 8.466 | **27.566** | 14.932 | 1.616E-02 |
| [235811_at](https://www.affymetrix.com/LinkServlet?probeset=235811_at) | [NA](http://www.ncbi.nlm.nih.gov/entrez/query.fcgi?cmd=search&db=gene&term=NA) | NA | **1.667** | **13.855** | 9.911 | **23.091** | 12.767 | 3.388E-02 |
| [240798_at](https://www.affymetrix.com/LinkServlet?probeset=240798_at) | [NA](http://www.ncbi.nlm.nih.gov/entrez/query.fcgi?cmd=search&db=gene&term=NA) | NA | **1.668** | **27.285** | 16.913 | **45.508** | 24.404 | 3.950E-02 |
| [203661_s_at](https://www.affymetrix.com/LinkServlet?probeset=203661_s_at) | [TMOD1](http://www.ncbi.nlm.nih.gov/entrez/query.fcgi?cmd=search&db=gene&term=TMOD1) | tropomodulin 1 | **1.668** | **16.165** | 9.917 | **26.968** | 18.044 | 4.365E-02 |
| [1553982_a_at](https://www.affymetrix.com/LinkServlet?probeset=1553982_a_at) | [RAB7B](http://www.ncbi.nlm.nih.gov/entrez/query.fcgi?cmd=search&db=gene&term=RAB7B) | RAB7B, member RAS oncogene family | **1.672** | **26.843** | 31.666 | **44.881** | 25.537 | 2.759E-02 |
| [213936_x_at](https://www.affymetrix.com/LinkServlet?probeset=213936_x_at) | [SFTPB](http://www.ncbi.nlm.nih.gov/entrez/query.fcgi?cmd=search&db=gene&term=SFTPB) | surfactant protein B | **1.674** | **6.107** | 0.398 | **10.225** | 6.810 | 1.133E-02 |
| [225584_at](https://www.affymetrix.com/LinkServlet?probeset=225584_at) | [HCG18](http://www.ncbi.nlm.nih.gov/entrez/query.fcgi?cmd=search&db=gene&term=HCG18) | HLA complex group 18 | **1.676** | **36.982** | 17.746 | **61.986** | 28.997 | 1.430E-02 |
| [1564413_at](https://www.affymetrix.com/LinkServlet?probeset=1564413_at) | [FLJ36116](http://www.ncbi.nlm.nih.gov/entrez/query.fcgi?cmd=search&db=gene&term=FLJ36116) | hypothetical locus LOC388666 | **1.678** | **29.611** | 17.111 | **49.690** | 26.003 | 3.649E-02 |
| [206318_at](https://www.affymetrix.com/LinkServlet?probeset=206318_at) | [SPINLW1](http://www.ncbi.nlm.nih.gov/entrez/query.fcgi?cmd=search&db=gene&term=SPINLW1) | serine peptidase inhibitor-like, with Kunitz and WAP domains 1 (eppin) | **1.679** | **6.875** | 1.814 | **11.541** | 7.920 | 2.272E-02 |
| [236219_at](https://www.affymetrix.com/LinkServlet?probeset=236219_at) | [TMEM20](http://www.ncbi.nlm.nih.gov/entrez/query.fcgi?cmd=search&db=gene&term=TMEM20) | transmembrane protein 20 | **1.679** | **6.437** | 1.485 | **10.810** | 5.808 | 3.358E-03 |
| [1559975_at](https://www.affymetrix.com/LinkServlet?probeset=1559975_at) | [BTG1](http://www.ncbi.nlm.nih.gov/entrez/query.fcgi?cmd=search&db=gene&term=BTG1) | B-cell translocation gene 1, anti-proliferative | **1.684** | **39.693** | 18.803 | **66.842** | 34.445 | 3.661E-02 |
| [214947_at](https://www.affymetrix.com/LinkServlet?probeset=214947_at) | [FAM105A](http://www.ncbi.nlm.nih.gov/entrez/query.fcgi?cmd=search&db=gene&term=FAM105A) | family with sequence similarity 105, member A | **1.685** | **25.549** | 21.900 | **43.038** | 23.462 | 2.677E-02 |
| [236603_at](https://www.affymetrix.com/LinkServlet?probeset=236603_at) | [NA](http://www.ncbi.nlm.nih.gov/entrez/query.fcgi?cmd=search&db=gene&term=NA) | NA | **1.687** | **12.505** | 5.364 | **21.094** | 11.332 | 3.170E-02 |
| [236336_at](https://www.affymetrix.com/LinkServlet?probeset=236336_at) | [NA](http://www.ncbi.nlm.nih.gov/entrez/query.fcgi?cmd=search&db=gene&term=NA) | NA | **1.687** | **7.147** | 1.771 | **12.057** | 8.168 | 2.292E-02 |
| [208530_s_at](https://www.affymetrix.com/LinkServlet?probeset=208530_s_at) | [RARB](http://www.ncbi.nlm.nih.gov/entrez/query.fcgi?cmd=search&db=gene&term=RARB) | retinoic acid receptor, beta | **1.688** | **21.871** | 9.839 | **36.909** | 19.103 | 1.408E-02 |
| [210873_x_at](https://www.affymetrix.com/LinkServlet?probeset=210873_x_at) | [APOBEC3A](http://www.ncbi.nlm.nih.gov/entrez/query.fcgi?cmd=search&db=gene&term=APOBEC3A) | apolipoprotein B mRNA editing enzyme, catalytic polypeptide-like 3A | **1.689** | **47.178** | 23.640 | **79.693** | 30.521 | 4.566E-03 |
| [219479_at](https://www.affymetrix.com/LinkServlet?probeset=219479_at) | [KDELC1](http://www.ncbi.nlm.nih.gov/entrez/query.fcgi?cmd=search&db=gene&term=KDELC1) | KDEL (Lys-Asp-Glu-Leu) containing 1 | **1.691** | **17.110** | 9.020 | **28.939** | 17.590 | 2.786E-02 |
| [238411_x_at](https://www.affymetrix.com/LinkServlet?probeset=238411_x_at) | [C5orf13](http://www.ncbi.nlm.nih.gov/entrez/query.fcgi?cmd=search&db=gene&term=C5orf13) | chromosome 5 open reading frame 13 | **1.692** | **6.021** | 0.084 | **10.188** | 7.225 | 8.474E-03 |
| [243049_at](https://www.affymetrix.com/LinkServlet?probeset=243049_at) | [NA](http://www.ncbi.nlm.nih.gov/entrez/query.fcgi?cmd=search&db=gene&term=NA) | NA | **1.695** | **14.121** | 8.368 | **23.937** | 14.438 | 3.400E-02 |
| [225020_at](https://www.affymetrix.com/LinkServlet?probeset=225020_at) | [DAB2IP](http://www.ncbi.nlm.nih.gov/entrez/query.fcgi?cmd=search&db=gene&term=DAB2IP) | DAB2 interacting protein | **1.697** | **29.363** | 21.279 | **49.822** | 28.972 | 3.793E-02 |
| [203103_s_at](https://www.affymetrix.com/LinkServlet?probeset=203103_s_at) | [PRPF19](http://www.ncbi.nlm.nih.gov/entrez/query.fcgi?cmd=search&db=gene&term=PRPF19) | PRP19/PSO4 pre-mRNA processing factor 19 homolog (S. cerevisiae) | **1.698** | **90.716** | 33.002 | **153.998** | 102.348 | 4.945E-02 |
| [229537_at](https://www.affymetrix.com/LinkServlet?probeset=229537_at) | [LMO4](http://www.ncbi.nlm.nih.gov/entrez/query.fcgi?cmd=search&db=gene&term=LMO4) | LIM domain only 4 | **1.699** | **21.357** | 9.868 | **36.277** | 30.290 | 4.356E-02 |
| [241279_at](https://www.affymetrix.com/LinkServlet?probeset=241279_at) | [NA](http://www.ncbi.nlm.nih.gov/entrez/query.fcgi?cmd=search&db=gene&term=NA) | NA | **1.699** | **12.766** | 8.330 | **21.689** | 12.306 | 4.371E-02 |
| [244677_at](https://www.affymetrix.com/LinkServlet?probeset=244677_at) | [NA](http://www.ncbi.nlm.nih.gov/entrez/query.fcgi?cmd=search&db=gene&term=NA) | NA | **1.699** | **65.195** | 35.649 | **110.761** | 61.201 | 2.206E-02 |
| [221406_s_at](https://www.affymetrix.com/LinkServlet?probeset=221406_s_at) | [NA](http://www.ncbi.nlm.nih.gov/entrez/query.fcgi?cmd=search&db=gene&term=NA) | NA | **1.705** | **14.325** | 5.834 | **24.418** | 7.200 | 1.195E-03 |
| [203300_x_at](https://www.affymetrix.com/LinkServlet?probeset=203300_x_at) | [AP1S2](http://www.ncbi.nlm.nih.gov/entrez/query.fcgi?cmd=search&db=gene&term=AP1S2) | adaptor-related protein complex 1, sigma 2 subunit | **1.705** | **148.449** | 108.213 | **253.177** | 109.047 | 2.388E-02 |
| [215278_at](https://www.affymetrix.com/LinkServlet?probeset=215278_at) | [NA](http://www.ncbi.nlm.nih.gov/entrez/query.fcgi?cmd=search&db=gene&term=NA) | NA | **1.706** | **7.006** | 2.632 | **11.949** | 10.220 | 4.899E-02 |
| [1553133_at](https://www.affymetrix.com/LinkServlet?probeset=1553133_at) | [C9orf72](http://www.ncbi.nlm.nih.gov/entrez/query.fcgi?cmd=search&db=gene&term=C9orf72) | chromosome 9 open reading frame 72 | **1.711** | **30.760** | 23.009 | **52.644** | 29.261 | 3.245E-02 |
| [232138_at](https://www.affymetrix.com/LinkServlet?probeset=232138_at) | [MBNL2](http://www.ncbi.nlm.nih.gov/entrez/query.fcgi?cmd=search&db=gene&term=MBNL2) | muscleblind-like 2 (Drosophila) | **1.713** | **50.896** | 26.684 | **87.175** | 51.140 | 2.401E-02 |
| [243560_at](https://www.affymetrix.com/LinkServlet?probeset=243560_at) | [NA](http://www.ncbi.nlm.nih.gov/entrez/query.fcgi?cmd=search&db=gene&term=NA) | NA | **1.714** | **8.218** | 4.856 | **14.085** | 10.549 | 4.294E-02 |
| [1559119_at](https://www.affymetrix.com/LinkServlet?probeset=1559119_at) | [NA](http://www.ncbi.nlm.nih.gov/entrez/query.fcgi?cmd=search&db=gene&term=NA) | NA | **1.716** | **67.731** | 47.714 | **116.231** | 44.209 | 1.716E-02 |
| [236917_at](https://www.affymetrix.com/LinkServlet?probeset=236917_at) | [LRRC34](http://www.ncbi.nlm.nih.gov/entrez/query.fcgi?cmd=search&db=gene&term=LRRC34) | leucine rich repeat containing 34 | **1.716** | **7.411** | 2.788 | **12.720** | 8.700 | 2.553E-02 |
| [205368_at](https://www.affymetrix.com/LinkServlet?probeset=205368_at) | [FAM131B](http://www.ncbi.nlm.nih.gov/entrez/query.fcgi?cmd=search&db=gene&term=FAM131B) | family with sequence similarity 131, member B | **1.718** | **40.365** | 28.004 | **69.329** | 49.040 | 2.279E-02 |
| [230939_at](https://www.affymetrix.com/LinkServlet?probeset=230939_at) | [NA](http://www.ncbi.nlm.nih.gov/entrez/query.fcgi?cmd=search&db=gene&term=NA) | NA | **1.719** | **8.695** | 5.434 | **14.948** | 9.535 | 2.183E-02 |
| [209959_at](https://www.affymetrix.com/LinkServlet?probeset=209959_at) | [NR4A3](http://www.ncbi.nlm.nih.gov/entrez/query.fcgi?cmd=search&db=gene&term=NR4A3) | nuclear receptor subfamily 4, group A, member 3 | **1.721** | **421.849** | 293.462 | **725.993** | 412.715 | 4.247E-02 |
| [1558697_a_at](https://www.affymetrix.com/LinkServlet?probeset=1558697_a_at) | [KIAA0430](http://www.ncbi.nlm.nih.gov/entrez/query.fcgi?cmd=search&db=gene&term=KIAA0430) | KIAA0430 | **1.722** | **8.192** | 2.966 | **14.104** | 7.321 | 9.007E-03 |
| [215828_at](https://www.affymetrix.com/LinkServlet?probeset=215828_at) | [NA](http://www.ncbi.nlm.nih.gov/entrez/query.fcgi?cmd=search&db=gene&term=NA) | NA | **1.722** | **22.954** | 18.275 | **39.528** | 20.693 | 2.006E-02 |
| [1553186_x_at](https://www.affymetrix.com/LinkServlet?probeset=1553186_x_at) | [RASEF](http://www.ncbi.nlm.nih.gov/entrez/query.fcgi?cmd=search&db=gene&term=RASEF) | RAS and EF-hand domain containing | **1.722** | **289.847** | 162.201 | **499.248** | 339.697 | 3.665E-02 |
| [1569013_s_at](https://www.affymetrix.com/LinkServlet?probeset=1569013_s_at) | [LOC96610](http://www.ncbi.nlm.nih.gov/entrez/query.fcgi?cmd=search&db=gene&term=LOC96610) | BMS1 homolog, ribosome assembly protein (yeast) pseudogene | **1.725** | **19.028** | 18.539 | **32.813** | 19.074 | 3.350E-02 |
| [1554406_a_at](https://www.affymetrix.com/LinkServlet?probeset=1554406_a_at) | [CLEC7A](http://www.ncbi.nlm.nih.gov/entrez/query.fcgi?cmd=search&db=gene&term=CLEC7A) | C-type lectin domain family 7, member A | **1.731** | **253.651** | 146.853 | **439.090** | 229.211 | 3.452E-02 |
| [233103_at](https://www.affymetrix.com/LinkServlet?probeset=233103_at) | [NA](http://www.ncbi.nlm.nih.gov/entrez/query.fcgi?cmd=search&db=gene&term=NA) | NA | **1.736** | **7.972** | 2.888 | **13.842** | 8.313 | 1.190E-02 |
| [242674_at](https://www.affymetrix.com/LinkServlet?probeset=242674_at) | [NA](http://www.ncbi.nlm.nih.gov/entrez/query.fcgi?cmd=search&db=gene&term=NA) | NA | **1.738** | **17.841** | 10.730 | **31.002** | 15.207 | 2.159E-02 |
| [233294_at](https://www.affymetrix.com/LinkServlet?probeset=233294_at) | [DENND2C](http://www.ncbi.nlm.nih.gov/entrez/query.fcgi?cmd=search&db=gene&term=DENND2C) | DENN/MADD domain containing 2C | **1.739** | **6.055** | 0.221 | **10.531** | 8.392 | 2.386E-02 |
| [239519_at](https://www.affymetrix.com/LinkServlet?probeset=239519_at) | [NA](http://www.ncbi.nlm.nih.gov/entrez/query.fcgi?cmd=search&db=gene&term=NA) | NA | **1.742** | **10.324** | 5.484 | **17.983** | 11.467 | 4.389E-02 |
| [1552717_s_at](https://www.affymetrix.com/LinkServlet?probeset=1552717_s_at) | [NA](http://www.ncbi.nlm.nih.gov/entrez/query.fcgi?cmd=search&db=gene&term=NA) | NA | **1.743** | **6.000** | 0.000 | **10.460** | 10.148 | 2.645E-02 |
| [1553134_s_at](https://www.affymetrix.com/LinkServlet?probeset=1553134_s_at) | [C9orf72](http://www.ncbi.nlm.nih.gov/entrez/query.fcgi?cmd=search&db=gene&term=C9orf72) | chromosome 9 open reading frame 72 | **1.745** | **9.890** | 8.107 | **17.263** | 8.519 | 1.315E-02 |
| [234607_at](https://www.affymetrix.com/LinkServlet?probeset=234607_at) | [ARRDC1](http://www.ncbi.nlm.nih.gov/entrez/query.fcgi?cmd=search&db=gene&term=ARRDC1) | arrestin domain containing 1 | **1.746** | **9.100** | 3.313 | **15.884** | 9.686 | 4.163E-02 |
| [240544_at](https://www.affymetrix.com/LinkServlet?probeset=240544_at) | [NA](http://www.ncbi.nlm.nih.gov/entrez/query.fcgi?cmd=search&db=gene&term=NA) | NA | **1.747** | **16.559** | 13.853 | **28.934** | 16.199 | 1.257E-02 |
| [236216_at](https://www.affymetrix.com/LinkServlet?probeset=236216_at) | [NA](http://www.ncbi.nlm.nih.gov/entrez/query.fcgi?cmd=search&db=gene&term=NA) | NA | **1.747** | **8.119** | 5.698 | **14.187** | 8.059 | 1.475E-02 |
| [1569136_at](https://www.affymetrix.com/LinkServlet?probeset=1569136_at) | [MGAT4A](http://www.ncbi.nlm.nih.gov/entrez/query.fcgi?cmd=search&db=gene&term=MGAT4A) | mannosyl (alpha-1,3-)-glycoprotein beta-1,4-N-acetylglucosaminyltransferase, isozyme A | **1.749** | **146.372** | 68.107 | **256.032** | 151.187 | 2.697E-02 |
| [232891_at](https://www.affymetrix.com/LinkServlet?probeset=232891_at) | [SIRPD](http://www.ncbi.nlm.nih.gov/entrez/query.fcgi?cmd=search&db=gene&term=SIRPD) | signal-regulatory protein delta | **1.754** | **6.425** | 1.031 | **11.269** | 9.547 | 2.906E-02 |
| [224822_at](https://www.affymetrix.com/LinkServlet?probeset=224822_at) | [DLC1](http://www.ncbi.nlm.nih.gov/entrez/query.fcgi?cmd=search&db=gene&term=DLC1) | deleted in liver cancer 1 | **1.758** | **15.473** | 22.981 | **27.205** | 25.087 | 3.351E-02 |
| [242414_at](https://www.affymetrix.com/LinkServlet?probeset=242414_at) | [QPRT](http://www.ncbi.nlm.nih.gov/entrez/query.fcgi?cmd=search&db=gene&term=QPRT) | quinolinate phosphoribosyltransferase | **1.758** | **40.838** | 22.925 | **71.804** | 48.569 | 3.345E-02 |
| [202952_s_at](https://www.affymetrix.com/LinkServlet?probeset=202952_s_at) | [ADAM12](http://www.ncbi.nlm.nih.gov/entrez/query.fcgi?cmd=search&db=gene&term=ADAM12) | ADAM metallopeptidase domain 12 | **1.761** | **7.450** | 3.303 | **13.118** | 9.279 | 4.669E-02 |
| [240326_at](https://www.affymetrix.com/LinkServlet?probeset=240326_at) | [NA](http://www.ncbi.nlm.nih.gov/entrez/query.fcgi?cmd=search&db=gene&term=NA) | NA | **1.763** | **18.354** | 12.678 | **32.357** | 21.546 | 2.173E-02 |
| [1565149_at](https://www.affymetrix.com/LinkServlet?probeset=1565149_at) | [DYNC2H1](http://www.ncbi.nlm.nih.gov/entrez/query.fcgi?cmd=search&db=gene&term=DYNC2H1) | dynein, cytoplasmic 2, heavy chain 1 | **1.764** | **6.236** | 0.936 | **10.999** | 6.070 | 2.724E-03 |
| [209883_at](https://www.affymetrix.com/LinkServlet?probeset=209883_at) | [GLT25D2](http://www.ncbi.nlm.nih.gov/entrez/query.fcgi?cmd=search&db=gene&term=GLT25D2) | glycosyltransferase 25 domain containing 2 | **1.764** | **54.692** | 31.531 | **96.484** | 41.168 | 5.431E-03 |
| [1553608_a_at](https://www.affymetrix.com/LinkServlet?probeset=1553608_a_at) | [NCRNA00189](http://www.ncbi.nlm.nih.gov/entrez/query.fcgi?cmd=search&db=gene&term=NCRNA00189) | non-protein coding RNA 189 | **1.769** | **6.442** | 1.208 | **11.396** | 9.724 | 3.714E-02 |
| [208790_s_at](https://www.affymetrix.com/LinkServlet?probeset=208790_s_at) | [PTRF](http://www.ncbi.nlm.nih.gov/entrez/query.fcgi?cmd=search&db=gene&term=PTRF) | polymerase I and transcript release factor | **1.775** | **7.491** | 4.236 | **13.296** | 10.996 | 2.332E-02 |
| [1570061_at](https://www.affymetrix.com/LinkServlet?probeset=1570061_at) | [NA](http://www.ncbi.nlm.nih.gov/entrez/query.fcgi?cmd=search&db=gene&term=NA) | NA | **1.778** | **12.712** | 7.538 | **22.602** | 14.666 | 4.040E-02 |
| [1559977_a_at](https://www.affymetrix.com/LinkServlet?probeset=1559977_a_at) | [SLC25A34](http://www.ncbi.nlm.nih.gov/entrez/query.fcgi?cmd=search&db=gene&term=SLC25A34) | solute carrier family 25, member 34 | **1.785** | **42.758** | 23.455 | **76.308** | 40.171 | 1.311E-02 |
| [240023_at](https://www.affymetrix.com/LinkServlet?probeset=240023_at) | [NA](http://www.ncbi.nlm.nih.gov/entrez/query.fcgi?cmd=search&db=gene&term=NA) | NA | **1.794** | **37.687** | 18.432 | **67.611** | 38.653 | 2.009E-02 |
| [226192_at](https://www.affymetrix.com/LinkServlet?probeset=226192_at) | [AR](http://www.ncbi.nlm.nih.gov/entrez/query.fcgi?cmd=search&db=gene&term=AR) | androgen receptor | **1.794** | **9.047** | 5.836 | **16.231** | 12.671 | 1.356E-02 |
| [233683_at](https://www.affymetrix.com/LinkServlet?probeset=233683_at) | [NA](http://www.ncbi.nlm.nih.gov/entrez/query.fcgi?cmd=search&db=gene&term=NA) | NA | **1.795** | **6.000** | 0.000 | **10.770** | 9.855 | 1.585E-02 |
| [237342_at](https://www.affymetrix.com/LinkServlet?probeset=237342_at) | [TOLLIP](http://www.ncbi.nlm.nih.gov/entrez/query.fcgi?cmd=search&db=gene&term=TOLLIP) | toll interacting protein | **1.796** | **7.729** | 2.798 | **13.884** | 10.338 | 4.486E-02 |
| [233939_at](https://www.affymetrix.com/LinkServlet?probeset=233939_at) | [REXO1](http://www.ncbi.nlm.nih.gov/entrez/query.fcgi?cmd=search&db=gene&term=REXO1) | REX1, RNA exonuclease 1 homolog (S. cerevisiae) | **1.799** | **6.449** | 1.091 | **11.603** | 10.710 | 4.235E-02 |
| [205130_at](https://www.affymetrix.com/LinkServlet?probeset=205130_at) | [RAGE](http://www.ncbi.nlm.nih.gov/entrez/query.fcgi?cmd=search&db=gene&term=RAGE) | renal tumor antigen | **1.800** | **11.928** | 6.465 | **21.469** | 10.579 | 8.262E-03 |
| [205896_at](https://www.affymetrix.com/LinkServlet?probeset=205896_at) | [SLC22A4](http://www.ncbi.nlm.nih.gov/entrez/query.fcgi?cmd=search&db=gene&term=SLC22A4) | solute carrier family 22 (organic cation/ergothioneine transporter), member 4 | **1.805** | **7.404** | 3.652 | **13.363** | 10.197 | 4.776E-02 |
| [205119_s_at](https://www.affymetrix.com/LinkServlet?probeset=205119_s_at) | [FPR1](http://www.ncbi.nlm.nih.gov/entrez/query.fcgi?cmd=search&db=gene&term=FPR1) | formyl peptide receptor 1 | **1.808** | **402.120** | 299.727 | **726.869** | 362.004 | 2.820E-02 |
| [232152_at](https://www.affymetrix.com/LinkServlet?probeset=232152_at) | [CEP57L1](http://www.ncbi.nlm.nih.gov/entrez/query.fcgi?cmd=search&db=gene&term=CEP57L1) | centrosomal protein 57kDa-like 1 | **1.809** | **6.709** | 2.287 | **12.135** | 10.584 | 4.411E-02 |
| [239104_at](https://www.affymetrix.com/LinkServlet?probeset=239104_at) | [NA](http://www.ncbi.nlm.nih.gov/entrez/query.fcgi?cmd=search&db=gene&term=NA) | NA | **1.811** | **16.674** | 9.728 | **30.197** | 17.368 | 4.114E-02 |
| [207005_s_at](https://www.affymetrix.com/LinkServlet?probeset=207005_s_at) | [BCL2](http://www.ncbi.nlm.nih.gov/entrez/query.fcgi?cmd=search&db=gene&term=BCL2) | B-cell CLL/lymphoma 2 | **1.815** | **7.091** | 1.809 | **12.871** | 13.402 | 4.913E-02 |
| [219660_s_at](https://www.affymetrix.com/LinkServlet?probeset=219660_s_at) | [ATP8A2](http://www.ncbi.nlm.nih.gov/entrez/query.fcgi?cmd=search&db=gene&term=ATP8A2) | ATPase, aminophospholipid transporter, class I, type 8A, member 2 | **1.817** | **6.266** | 0.760 | **11.386** | 9.689 | 9.740E-03 |
| [1556178_x_at](https://www.affymetrix.com/LinkServlet?probeset=1556178_x_at) | [TAF8](http://www.ncbi.nlm.nih.gov/entrez/query.fcgi?cmd=search&db=gene&term=TAF8) | TAF8 RNA polymerase II, TATA box binding protein (TBP)-associated factor, 43kDa | **1.817** | **67.503** | 27.625 | **122.665** | 73.564 | 1.749E-02 |
| [216249_at](https://www.affymetrix.com/LinkServlet?probeset=216249_at) | [PVT1](http://www.ncbi.nlm.nih.gov/entrez/query.fcgi?cmd=search&db=gene&term=PVT1) | Pvt1 oncogene (non-protein coding) | **1.818** | **19.856** | 16.645 | **36.098** | 20.896 | 1.740E-02 |
| [241401_at](https://www.affymetrix.com/LinkServlet?probeset=241401_at) | [NCRNA00247](http://www.ncbi.nlm.nih.gov/entrez/query.fcgi?cmd=search&db=gene&term=NCRNA00247) | non-protein coding RNA 247 | **1.821** | **6.000** | 0.000 | **10.928** | 10.892 | 2.519E-02 |
| [211102_s_at](https://www.affymetrix.com/LinkServlet?probeset=211102_s_at) | [LILRA2](http://www.ncbi.nlm.nih.gov/entrez/query.fcgi?cmd=search&db=gene&term=LILRA2) | leukocyte immunoglobulin-like receptor, subfamily A (with TM domain), member 2 | **1.822** | **15.125** | 8.812 | **27.554** | 18.750 | 3.678E-02 |
| [208920_at](https://www.affymetrix.com/LinkServlet?probeset=208920_at) | [SRI](http://www.ncbi.nlm.nih.gov/entrez/query.fcgi?cmd=search&db=gene&term=SRI) | sorcin | **1.823** | **133.282** | 42.898 | **243.035** | 123.592 | 9.444E-04 |
| [1569231_x_at](https://www.affymetrix.com/LinkServlet?probeset=1569231_x_at) | [NA](http://www.ncbi.nlm.nih.gov/entrez/query.fcgi?cmd=search&db=gene&term=NA) | NA | **1.833** | **6.166** | 0.501 | **11.303** | 10.544 | 3.661E-02 |
| [213131_at](https://www.affymetrix.com/LinkServlet?probeset=213131_at) | [OLFM1](http://www.ncbi.nlm.nih.gov/entrez/query.fcgi?cmd=search&db=gene&term=OLFM1) | olfactomedin 1 | **1.833** | **9.867** | 10.108 | **18.086** | 14.416 | 2.868E-02 |
| [238281_at](https://www.affymetrix.com/LinkServlet?probeset=238281_at) | [NA](http://www.ncbi.nlm.nih.gov/entrez/query.fcgi?cmd=search&db=gene&term=NA) | NA | **1.834** | **7.678** | 3.145 | **14.085** | 11.080 | 3.128E-02 |
| [233300_at](https://www.affymetrix.com/LinkServlet?probeset=233300_at) | [NA](http://www.ncbi.nlm.nih.gov/entrez/query.fcgi?cmd=search&db=gene&term=NA) | NA | **1.835** | **35.062** | 14.590 | **64.336** | 47.996 | 2.867E-02 |
| [224943_at](https://www.affymetrix.com/LinkServlet?probeset=224943_at) | [BTBD7](http://www.ncbi.nlm.nih.gov/entrez/query.fcgi?cmd=search&db=gene&term=BTBD7) | BTB (POZ) domain containing 7 | **1.837** | **19.664** | 12.287 | **36.118** | 20.119 | 3.542E-02 |
| [240444_x_at](https://www.affymetrix.com/LinkServlet?probeset=240444_x_at) | [CLIP1](http://www.ncbi.nlm.nih.gov/entrez/query.fcgi?cmd=search&db=gene&term=CLIP1) | CAP-GLY domain containing linker protein 1 | **1.838** | **7.342** | 2.980 | **13.498** | 8.098 | 5.455E-03 |
| [1554873_at](https://www.affymetrix.com/LinkServlet?probeset=1554873_at) | [CSPP1](http://www.ncbi.nlm.nih.gov/entrez/query.fcgi?cmd=search&db=gene&term=CSPP1) | centrosome and spindle pole associated protein 1 | **1.843** | **7.048** | 1.935 | **12.987** | 14.054 | 4.419E-02 |
| [1553185_at](https://www.affymetrix.com/LinkServlet?probeset=1553185_at) | [RASEF](http://www.ncbi.nlm.nih.gov/entrez/query.fcgi?cmd=search&db=gene&term=RASEF) | RAS and EF-hand domain containing | **1.844** | **461.087** | 247.860 | **850.040** | 541.240 | 2.944E-02 |
| [205715_at](https://www.affymetrix.com/LinkServlet?probeset=205715_at) | [BST1](http://www.ncbi.nlm.nih.gov/entrez/query.fcgi?cmd=search&db=gene&term=BST1) | bone marrow stromal cell antigen 1 | **1.848** | **88.676** | 54.726 | **163.907** | 98.757 | 3.268E-02 |
| [1556817_a_at](https://www.affymetrix.com/LinkServlet?probeset=1556817_a_at) | [NA](http://www.ncbi.nlm.nih.gov/entrez/query.fcgi?cmd=search&db=gene&term=NA) | NA | **1.853** | **6.135** | 0.541 | **11.367** | 10.102 | 4.310E-02 |
| [226492_at](https://www.affymetrix.com/LinkServlet?probeset=226492_at) | [SEMA6D](http://www.ncbi.nlm.nih.gov/entrez/query.fcgi?cmd=search&db=gene&term=SEMA6D) | sema domain, transmembrane domain (TM), and cytoplasmic domain, (semaphorin) 6D | **1.854** | **6.462** | 1.341 | **11.978** | 8.351 | 9.517E-03 |
| [217999_s_at](https://www.affymetrix.com/LinkServlet?probeset=217999_s_at) | [PHLDA1](http://www.ncbi.nlm.nih.gov/entrez/query.fcgi?cmd=search&db=gene&term=PHLDA1) | pleckstrin homology-like domain, family A, member 1 | **1.857** | **18.532** | 10.274 | **34.419** | 33.166 | 3.090E-02 |
| [240975_x_at](https://www.affymetrix.com/LinkServlet?probeset=240975_x_at) | [RBFOX3](http://www.ncbi.nlm.nih.gov/entrez/query.fcgi?cmd=search&db=gene&term=RBFOX3) | RNA binding protein, fox-1 homolog (C. elegans) 3 | **1.857** | **9.579** | 5.370 | **17.792** | 10.313 | 5.634E-03 |
| [243797_at](https://www.affymetrix.com/LinkServlet?probeset=243797_at) | [STK17B](http://www.ncbi.nlm.nih.gov/entrez/query.fcgi?cmd=search&db=gene&term=STK17B) | serine/threonine kinase 17b | **1.858** | **36.651** | 21.023 | **68.096** | 33.998 | 2.205E-02 |
| [235120_at](https://www.affymetrix.com/LinkServlet?probeset=235120_at) | [SEC22C](http://www.ncbi.nlm.nih.gov/entrez/query.fcgi?cmd=search&db=gene&term=SEC22C) | SEC22 vesicle trafficking protein homolog C (S. cerevisiae) | **1.859** | **13.569** | 8.886 | **25.222** | 7.880 | 1.015E-03 |
| [210519_s_at](https://www.affymetrix.com/LinkServlet?probeset=210519_s_at) | [NQO1](http://www.ncbi.nlm.nih.gov/entrez/query.fcgi?cmd=search&db=gene&term=NQO1) | NAD(P)H dehydrogenase, quinone 1 | **1.860** | **15.414** | 8.941 | **28.663** | 15.368 | 4.484E-03 |
| [211673_s_at](https://www.affymetrix.com/LinkServlet?probeset=211673_s_at) | [MOCS1](http://www.ncbi.nlm.nih.gov/entrez/query.fcgi?cmd=search&db=gene&term=MOCS1) | molybdenum cofactor synthesis 1 | **1.863** | **6.711** | 1.315 | **12.504** | 9.009 | 1.301E-02 |
| [209189_at](https://www.affymetrix.com/LinkServlet?probeset=209189_at) | [FOS](http://www.ncbi.nlm.nih.gov/entrez/query.fcgi?cmd=search&db=gene&term=FOS) | FBJ murine osteosarcoma viral oncogene homolog | **1.868** | **141.296** | 136.588 | **263.985** | 147.604 | 1.378E-02 |
| [242561_at](https://www.affymetrix.com/LinkServlet?probeset=242561_at) | [IPO9](http://www.ncbi.nlm.nih.gov/entrez/query.fcgi?cmd=search&db=gene&term=IPO9) | importin 9 | **1.875** | **21.255** | 11.375 | **39.848** | 17.720 | 1.859E-03 |
| [239184_at](https://www.affymetrix.com/LinkServlet?probeset=239184_at) | [NA](http://www.ncbi.nlm.nih.gov/entrez/query.fcgi?cmd=search&db=gene&term=NA) | NA | **1.878** | **47.886** | 26.519 | **89.912** | 56.828 | 1.359E-02 |
| [216125_s_at](https://www.affymetrix.com/LinkServlet?probeset=216125_s_at) | [RANBP9](http://www.ncbi.nlm.nih.gov/entrez/query.fcgi?cmd=search&db=gene&term=RANBP9) | RAN binding protein 9 | **1.883** | **29.255** | 14.969 | **55.094** | 44.102 | 1.857E-02 |
| [239016_at](https://www.affymetrix.com/LinkServlet?probeset=239016_at) | [NA](http://www.ncbi.nlm.nih.gov/entrez/query.fcgi?cmd=search&db=gene&term=NA) | NA | **1.887** | **18.788** | 8.951 | **35.447** | 18.655 | 2.604E-03 |
| [230581_at](https://www.affymetrix.com/LinkServlet?probeset=230581_at) | [NA](http://www.ncbi.nlm.nih.gov/entrez/query.fcgi?cmd=search&db=gene&term=NA) | NA | **1.889** | **13.312** | 7.298 | **25.143** | 17.975 | 3.271E-02 |
| [228110_x_at](https://www.affymetrix.com/LinkServlet?probeset=228110_x_at) | [RABGEF1](http://www.ncbi.nlm.nih.gov/entrez/query.fcgi?cmd=search&db=gene&term=RABGEF1) | RAB guanine nucleotide exchange factor (GEF) 1 | **1.893** | **18.878** | 10.937 | **35.738** | 14.493 | 1.833E-03 |
| [216288_at](https://www.affymetrix.com/LinkServlet?probeset=216288_at) | [CYSLTR1](http://www.ncbi.nlm.nih.gov/entrez/query.fcgi?cmd=search&db=gene&term=CYSLTR1) | cysteinyl leukotriene receptor 1 | **1.898** | **7.927** | 2.477 | **15.048** | 9.918 | 1.768E-02 |
| [225308_s_at](https://www.affymetrix.com/LinkServlet?probeset=225308_s_at) | [TANC1](http://www.ncbi.nlm.nih.gov/entrez/query.fcgi?cmd=search&db=gene&term=TANC1) | tetratricopeptide repeat, ankyrin repeat and coiled-coil containing 1 | **1.900** | **9.178** | 6.761 | **17.438** | 16.708 | 4.945E-02 |
| [237904_at](https://www.affymetrix.com/LinkServlet?probeset=237904_at) | [NA](http://www.ncbi.nlm.nih.gov/entrez/query.fcgi?cmd=search&db=gene&term=NA) | NA | **1.908** | **6.550** | 1.560 | **12.496** | 9.537 | 1.814E-02 |
| [216222_s_at](https://www.affymetrix.com/LinkServlet?probeset=216222_s_at) | [MYO10](http://www.ncbi.nlm.nih.gov/entrez/query.fcgi?cmd=search&db=gene&term=MYO10) | myosin X | **1.908** | **7.298** | 1.990 | **13.925** | 12.791 | 3.863E-02 |
| [239042_at](https://www.affymetrix.com/LinkServlet?probeset=239042_at) | [TSR1](http://www.ncbi.nlm.nih.gov/entrez/query.fcgi?cmd=search&db=gene&term=TSR1) | TSR1, 20S rRNA accumulation, homolog (S. cerevisiae) | **1.913** | **10.153** | 5.140 | **19.424** | 13.052 | 4.358E-02 |
| [220874_at](https://www.affymetrix.com/LinkServlet?probeset=220874_at) | [NA](http://www.ncbi.nlm.nih.gov/entrez/query.fcgi?cmd=search&db=gene&term=NA) | NA | **1.916** | **8.752** | 3.090 | **16.767** | 12.598 | 3.217E-02 |
| [39402_at](https://www.affymetrix.com/LinkServlet?probeset=39402_at) | [IL1B](http://www.ncbi.nlm.nih.gov/entrez/query.fcgi?cmd=search&db=gene&term=IL1B) | interleukin 1, beta | **1.922** | **113.205** | 84.981 | **217.601** | 108.413 | 1.175E-02 |
| [242303_at](https://www.affymetrix.com/LinkServlet?probeset=242303_at) | [NRG2](http://www.ncbi.nlm.nih.gov/entrez/query.fcgi?cmd=search&db=gene&term=NRG2) | neuregulin 2 | **1.927** | **6.285** | 0.633 | **12.113** | 9.986 | 1.249E-02 |
| [1553655_at](https://www.affymetrix.com/LinkServlet?probeset=1553655_at) | [CDC20B](http://www.ncbi.nlm.nih.gov/entrez/query.fcgi?cmd=search&db=gene&term=CDC20B) | cell division cycle 20 homolog B (S. cerevisiae) | **1.929** | **6.049** | 0.195 | **11.669** | 10.286 | 3.267E-02 |
| [223977_s_at](https://www.affymetrix.com/LinkServlet?probeset=223977_s_at) | [C18orf2](http://www.ncbi.nlm.nih.gov/entrez/query.fcgi?cmd=search&db=gene&term=C18orf2) | chromosome 18 open reading frame 2 | **1.943** | **6.000** | 0.000 | **11.660** | 10.540 | 2.087E-02 |
| [227654_at](https://www.affymetrix.com/LinkServlet?probeset=227654_at) | [FAM65C](http://www.ncbi.nlm.nih.gov/entrez/query.fcgi?cmd=search&db=gene&term=FAM65C) | family with sequence similarity 65, member C | **1.946** | **6.656** | 1.497 | **12.949** | 16.364 | 4.699E-02 |
| [1556850_at](https://www.affymetrix.com/LinkServlet?probeset=1556850_at) | [CEP290](http://www.ncbi.nlm.nih.gov/entrez/query.fcgi?cmd=search&db=gene&term=CEP290) | centrosomal protein 290kDa | **1.951** | **13.646** | 6.476 | **26.626** | 15.728 | 1.393E-02 |
| [1559867_at](https://www.affymetrix.com/LinkServlet?probeset=1559867_at) | [NA](http://www.ncbi.nlm.nih.gov/entrez/query.fcgi?cmd=search&db=gene&term=NA) | NA | **1.952** | **11.955** | 4.390 | **23.341** | 14.296 | 5.529E-03 |
| [226676_at](https://www.affymetrix.com/LinkServlet?probeset=226676_at) | [ZNF521](http://www.ncbi.nlm.nih.gov/entrez/query.fcgi?cmd=search&db=gene&term=ZNF521) | zinc finger protein 521 | **1.956** | **7.281** | 3.079 | **14.242** | 11.043 | 1.491E-02 |
| [205230_at](https://www.affymetrix.com/LinkServlet?probeset=205230_at) | [RPH3A](http://www.ncbi.nlm.nih.gov/entrez/query.fcgi?cmd=search&db=gene&term=RPH3A) | rabphilin 3A homolog (mouse) | **1.960** | **6.769** | 1.544 | **13.271** | 11.903 | 2.054E-02 |
| [243568_at](https://www.affymetrix.com/LinkServlet?probeset=243568_at) | [NA](http://www.ncbi.nlm.nih.gov/entrez/query.fcgi?cmd=search&db=gene&term=NA) | NA | **1.961** | **7.640** | 2.842 | **14.981** | 11.047 | 3.674E-03 |
| [235930_at](https://www.affymetrix.com/LinkServlet?probeset=235930_at) | [NA](http://www.ncbi.nlm.nih.gov/entrez/query.fcgi?cmd=search&db=gene&term=NA) | NA | **1.961** | **10.291** | 4.245 | **20.181** | 22.948 | 4.911E-02 |
| [202718_at](https://www.affymetrix.com/LinkServlet?probeset=202718_at) | [IGFBP2](http://www.ncbi.nlm.nih.gov/entrez/query.fcgi?cmd=search&db=gene&term=IGFBP2) | insulin-like growth factor binding protein 2, 36kDa | **1.963** | **6.344** | 0.990 | **12.451** | 14.473 | 3.392E-02 |
| [240013_at](https://www.affymetrix.com/LinkServlet?probeset=240013_at) | [NA](http://www.ncbi.nlm.nih.gov/entrez/query.fcgi?cmd=search&db=gene&term=NA) | NA | **1.968** | **48.925** | 25.814 | **96.280** | 71.132 | 3.932E-02 |
| [231148_at](https://www.affymetrix.com/LinkServlet?probeset=231148_at) | [IGFL2](http://www.ncbi.nlm.nih.gov/entrez/query.fcgi?cmd=search&db=gene&term=IGFL2) | IGF-like family member 2 | **1.976** | **7.573** | 3.595 | **14.963** | 10.944 | 1.134E-02 |
| [230608_at](https://www.affymetrix.com/LinkServlet?probeset=230608_at) | [C1orf182](http://www.ncbi.nlm.nih.gov/entrez/query.fcgi?cmd=search&db=gene&term=C1orf182) | chromosome 1 open reading frame 182 | **1.986** | **6.112** | 0.449 | **12.139** | 12.983 | 4.257E-02 |
| [1557688_at](https://www.affymetrix.com/LinkServlet?probeset=1557688_at) | [NA](http://www.ncbi.nlm.nih.gov/entrez/query.fcgi?cmd=search&db=gene&term=NA) | NA | **1.991** | **43.709** | 26.190 | **87.039** | 36.447 | 2.956E-03 |
| [1552772_at](https://www.affymetrix.com/LinkServlet?probeset=1552772_at) | [CLEC4D](http://www.ncbi.nlm.nih.gov/entrez/query.fcgi?cmd=search&db=gene&term=CLEC4D) | C-type lectin domain family 4, member D | **1.993** | **19.292** | 14.218 | **38.455** | 32.520 | 1.606E-02 |
| [233099_at](https://www.affymetrix.com/LinkServlet?probeset=233099_at) | [NA](http://www.ncbi.nlm.nih.gov/entrez/query.fcgi?cmd=search&db=gene&term=NA) | NA | **1.996** | **20.878** | 17.759 | **41.667** | 32.041 | 4.372E-02 |
| [235576_at](https://www.affymetrix.com/LinkServlet?probeset=235576_at) | [WDR27](http://www.ncbi.nlm.nih.gov/entrez/query.fcgi?cmd=search&db=gene&term=WDR27) | WD repeat domain 27 | **1.996** | **34.155** | 20.783 | **68.180** | 30.054 | 9.294E-03 |
| [239543_s_at](https://www.affymetrix.com/LinkServlet?probeset=239543_s_at) | [NA](http://www.ncbi.nlm.nih.gov/entrez/query.fcgi?cmd=search&db=gene&term=NA) | NA | **1.998** | **13.943** | 5.404 | **27.856** | 16.889 | 1.133E-02 |
| [229581_at](https://www.affymetrix.com/LinkServlet?probeset=229581_at) | [ELFN1](http://www.ncbi.nlm.nih.gov/entrez/query.fcgi?cmd=search&db=gene&term=ELFN1) | extracellular leucine-rich repeat and fibronectin type III domain containing 1 | **2.000** | **7.443** | 3.640 | **14.887** | 13.615 | 2.491E-02 |
| [203634_s_at](https://www.affymetrix.com/LinkServlet?probeset=203634_s_at) | [CPT1A](http://www.ncbi.nlm.nih.gov/entrez/query.fcgi?cmd=search&db=gene&term=CPT1A) | carnitine palmitoyltransferase 1A (liver) | **2.000** | **15.464** | 9.988 | **30.932** | 19.656 | 2.738E-02 |
| [1558444_at](https://www.affymetrix.com/LinkServlet?probeset=1558444_at) | [NA](http://www.ncbi.nlm.nih.gov/entrez/query.fcgi?cmd=search&db=gene&term=NA) | NA | **2.015** | **18.463** | 11.216 | **37.207** | 23.752 | 4.382E-02 |
| [1554333_at](https://www.affymetrix.com/LinkServlet?probeset=1554333_at) | [DNAJA4](http://www.ncbi.nlm.nih.gov/entrez/query.fcgi?cmd=search&db=gene&term=DNAJA4) | DnaJ (Hsp40) homolog, subfamily A, member 4 | **2.020** | **8.372** | 3.838 | **16.912** | 14.511 | 2.537E-02 |
| [210305_at](https://www.affymetrix.com/LinkServlet?probeset=210305_at) | [PDE4DIP](http://www.ncbi.nlm.nih.gov/entrez/query.fcgi?cmd=search&db=gene&term=PDE4DIP) | phosphodiesterase 4D interacting protein | **2.028** | **12.247** | 8.771 | **24.842** | 18.870 | 2.286E-02 |
| [1569276_at](https://www.affymetrix.com/LinkServlet?probeset=1569276_at) | [NA](http://www.ncbi.nlm.nih.gov/entrez/query.fcgi?cmd=search&db=gene&term=NA) | NA | **2.028** | **11.113** | 5.106 | **22.542** | 19.893 | 4.006E-02 |
| [206237_s_at](https://www.affymetrix.com/LinkServlet?probeset=206237_s_at) | [NRG1](http://www.ncbi.nlm.nih.gov/entrez/query.fcgi?cmd=search&db=gene&term=NRG1) | neuregulin 1 | **2.029** | **6.476** | 1.370 | **13.136** | 15.275 | 3.845E-02 |
| [1557038_s_at](https://www.affymetrix.com/LinkServlet?probeset=1557038_s_at) | [LOC100289373](http://www.ncbi.nlm.nih.gov/entrez/query.fcgi?cmd=search&db=gene&term=LOC100289373) | hypothetical LOC100289373 | **2.040** | **8.910** | 3.748 | **18.179** | 14.244 | 2.122E-02 |
| [242908_x_at](https://www.affymetrix.com/LinkServlet?probeset=242908_x_at) | [NA](http://www.ncbi.nlm.nih.gov/entrez/query.fcgi?cmd=search&db=gene&term=NA) | NA | **2.040** | **7.261** | 2.399 | **14.814** | 9.867 | 5.656E-03 |
| [229770_at](https://www.affymetrix.com/LinkServlet?probeset=229770_at) | [GLT1D1](http://www.ncbi.nlm.nih.gov/entrez/query.fcgi?cmd=search&db=gene&term=GLT1D1) | glycosyltransferase 1 domain containing 1 | **2.041** | **29.908** | 28.361 | **61.031** | 56.072 | 4.807E-02 |
| [215467_x_at](https://www.affymetrix.com/LinkServlet?probeset=215467_x_at) | [LOC647070](http://www.ncbi.nlm.nih.gov/entrez/query.fcgi?cmd=search&db=gene&term=LOC647070) | hypothetical LOC647070 | **2.044** | **18.345** | 9.402 | **37.492** | 26.026 | 8.427E-03 |
| [1560349_at](https://www.affymetrix.com/LinkServlet?probeset=1560349_at) | [NA](http://www.ncbi.nlm.nih.gov/entrez/query.fcgi?cmd=search&db=gene&term=NA) | NA | **2.045** | **8.330** | 3.875 | **17.037** | 16.292 | 3.606E-02 |
| [240293_at](https://www.affymetrix.com/LinkServlet?probeset=240293_at) | [CCDC153](http://www.ncbi.nlm.nih.gov/entrez/query.fcgi?cmd=search&db=gene&term=CCDC153) | coiled-coil domain containing 153 | **2.047** | **16.632** | 8.013 | **34.044** | 28.062 | 4.382E-02 |
| [213369_at](https://www.affymetrix.com/LinkServlet?probeset=213369_at) | [CDHR1](http://www.ncbi.nlm.nih.gov/entrez/query.fcgi?cmd=search&db=gene&term=CDHR1) | cadherin-related family member 1 | **2.048** | **28.476** | 19.014 | **58.317** | 25.398 | 3.141E-03 |
| [226918_at](https://www.affymetrix.com/LinkServlet?probeset=226918_at) | [JPH4](http://www.ncbi.nlm.nih.gov/entrez/query.fcgi?cmd=search&db=gene&term=JPH4) | junctophilin 4 | **2.048** | **14.846** | 10.740 | **30.406** | 24.647 | 2.687E-02 |
| [1562265_at](https://www.affymetrix.com/LinkServlet?probeset=1562265_at) | [NA](http://www.ncbi.nlm.nih.gov/entrez/query.fcgi?cmd=search&db=gene&term=NA) | NA | **2.052** | **13.045** | 8.388 | **26.769** | 28.918 | 2.460E-02 |
| [206393_at](https://www.affymetrix.com/LinkServlet?probeset=206393_at) | [TNNI2](http://www.ncbi.nlm.nih.gov/entrez/query.fcgi?cmd=search&db=gene&term=TNNI2) | troponin I type 2 (skeletal, fast) | **2.062** | **25.846** | 12.217 | **53.301** | 32.268 | 1.219E-02 |
| [234276_at](https://www.affymetrix.com/LinkServlet?probeset=234276_at) | [NA](http://www.ncbi.nlm.nih.gov/entrez/query.fcgi?cmd=search&db=gene&term=NA) | NA | **2.069** | **6.942** | 1.368 | **14.360** | 13.771 | 2.040E-02 |
| [213551_x_at](https://www.affymetrix.com/LinkServlet?probeset=213551_x_at) | [NA](http://www.ncbi.nlm.nih.gov/entrez/query.fcgi?cmd=search&db=gene&term=NA) | NA | **2.079** | **36.686** | 24.177 | **76.265** | 80.985 | 2.613E-02 |
| [1569230_at](https://www.affymetrix.com/LinkServlet?probeset=1569230_at) | [NA](http://www.ncbi.nlm.nih.gov/entrez/query.fcgi?cmd=search&db=gene&term=NA) | NA | **2.104** | **6.401** | 1.063 | **13.470** | 14.030 | 2.390E-02 |
| [227666_at](https://www.affymetrix.com/LinkServlet?probeset=227666_at) | [DCLK2](http://www.ncbi.nlm.nih.gov/entrez/query.fcgi?cmd=search&db=gene&term=DCLK2) | doublecortin-like kinase 2 | **2.108** | **9.834** | 5.334 | **20.730** | 15.663 | 1.269E-02 |
| [242397_at](https://www.affymetrix.com/LinkServlet?probeset=242397_at) | [NA](http://www.ncbi.nlm.nih.gov/entrez/query.fcgi?cmd=search&db=gene&term=NA) | NA | **2.111** | **65.683** | 60.618 | **138.658** | 154.406 | 2.657E-02 |
| [243299_at](https://www.affymetrix.com/LinkServlet?probeset=243299_at) | [VRK2](http://www.ncbi.nlm.nih.gov/entrez/query.fcgi?cmd=search&db=gene&term=VRK2) | vaccinia related kinase 2 | **2.114** | **12.130** | 8.800 | **25.645** | 22.171 | 3.090E-02 |
| [243296_at](https://www.affymetrix.com/LinkServlet?probeset=243296_at) | [NAMPT](http://www.ncbi.nlm.nih.gov/entrez/query.fcgi?cmd=search&db=gene&term=NAMPT) | nicotinamide phosphoribosyltransferase | **2.116** | **78.078** | 50.644 | **165.226** | 118.699 | 7.235E-03 |
| [233079_at](https://www.affymetrix.com/LinkServlet?probeset=233079_at) | [NA](http://www.ncbi.nlm.nih.gov/entrez/query.fcgi?cmd=search&db=gene&term=NA) | NA | **2.118** | **7.567** | 3.174 | **16.027** | 11.588 | 3.567E-03 |
| [243294_at](https://www.affymetrix.com/LinkServlet?probeset=243294_at) | [ZNF780B](http://www.ncbi.nlm.nih.gov/entrez/query.fcgi?cmd=search&db=gene&term=ZNF780B) | zinc finger protein 780B | **2.118** | **10.887** | 5.543 | **23.064** | 17.715 | 6.647E-03 |
| [205067_at](https://www.affymetrix.com/LinkServlet?probeset=205067_at) | [IL1B](http://www.ncbi.nlm.nih.gov/entrez/query.fcgi?cmd=search&db=gene&term=IL1B) | interleukin 1, beta | **2.120** | **90.826** | 69.885 | **192.538** | 98.929 | 6.877E-03 |
| [236963_at](https://www.affymetrix.com/LinkServlet?probeset=236963_at) | [NA](http://www.ncbi.nlm.nih.gov/entrez/query.fcgi?cmd=search&db=gene&term=NA) | NA | **2.132** | **7.762** | 3.013 | **16.546** | 10.646 | 1.108E-03 |
| [239063_at](https://www.affymetrix.com/LinkServlet?probeset=239063_at) | [NA](http://www.ncbi.nlm.nih.gov/entrez/query.fcgi?cmd=search&db=gene&term=NA) | NA | **2.133** | **17.295** | 8.790 | **36.886** | 27.002 | 1.329E-02 |
| [1562189_at](https://www.affymetrix.com/LinkServlet?probeset=1562189_at) | [NA](http://www.ncbi.nlm.nih.gov/entrez/query.fcgi?cmd=search&db=gene&term=NA) | NA | **2.139** | **6.727** | 2.908 | **14.388** | 14.542 | 3.652E-02 |
| [1558920_at](https://www.affymetrix.com/LinkServlet?probeset=1558920_at) | [LOC100128590](http://www.ncbi.nlm.nih.gov/entrez/query.fcgi?cmd=search&db=gene&term=LOC100128590) | hypothetical LOC100128590 | **2.154** | **8.289** | 4.359 | **17.850** | 14.955 | 8.796E-03 |
| [235828_at](https://www.affymetrix.com/LinkServlet?probeset=235828_at) | [PRELID2](http://www.ncbi.nlm.nih.gov/entrez/query.fcgi?cmd=search&db=gene&term=PRELID2) | PRELI domain containing 2 | **2.154** | **12.976** | 6.184 | **27.950** | 21.147 | 2.114E-02 |
| [210074_at](https://www.affymetrix.com/LinkServlet?probeset=210074_at) | [CTSL2](http://www.ncbi.nlm.nih.gov/entrez/query.fcgi?cmd=search&db=gene&term=CTSL2) | cathepsin L2 | **2.160** | **9.967** | 3.521 | **21.527** | 12.387 | 3.855E-03 |
| [202436_s_at](https://www.affymetrix.com/LinkServlet?probeset=202436_s_at) | [CYP1B1](http://www.ncbi.nlm.nih.gov/entrez/query.fcgi?cmd=search&db=gene&term=CYP1B1) | cytochrome P450, family 1, subfamily B, polypeptide 1 | **2.164** | **21.599** | 18.053 | **46.749** | 29.454 | 6.517E-03 |
| [1570078_a_at](https://www.affymetrix.com/LinkServlet?probeset=1570078_a_at) | [DOCK5](http://www.ncbi.nlm.nih.gov/entrez/query.fcgi?cmd=search&db=gene&term=DOCK5) | dedicator of cytokinesis 5 | **2.167** | **9.803** | 4.398 | **21.239** | 26.492 | 4.239E-02 |
| [AFFX-r2-Bs-phe-3_at](https://www.affymetrix.com/LinkServlet?probeset=AFFX-r2-Bs-phe-3_at) | [NA](http://www.ncbi.nlm.nih.gov/entrez/query.fcgi?cmd=search&db=gene&term=NA) | NA | **2.174** | **543.280** | 453.479 | **1181.236** | 814.555 | 4.817E-02 |
| [228610_at](https://www.affymetrix.com/LinkServlet?probeset=228610_at) | [TM9SF3](http://www.ncbi.nlm.nih.gov/entrez/query.fcgi?cmd=search&db=gene&term=TM9SF3) | transmembrane 9 superfamily member 3 | **2.177** | **7.484** | 2.703 | **16.296** | 13.316 | 1.966E-02 |
[truncated: 20,037 more chars]
